# En Route to a Molecular Terminal Tin Oxide

**DOI:** 10.1021/acs.inorgchem.4c00598

**Published:** 2024-04-10

**Authors:** Leon Kreßner, Daniel Duvinage, Pim Puylaert, Nico Graw, Regine Herbst-Irmer, Dietmar Stalke, Oliver P. E. Townrow, Malte Fischer

**Affiliations:** † Institut für Anorganische Chemie, Georg-August-Universität Göttingen, Tammannstraße 4, D-37077 Göttingen, Germany; ‡ Institut für Anorganische Chemie und Kristallographie, Universität Bremen, Leobener Str. 7, D-28359 Bremen, Germany; § Inorganic and Organometallic Chemistry, Friedrich-Alexander-Universität Erlangen-Nürnberg, Egerlandstraße 1, D-91058 Erlangen, Germany

## Abstract

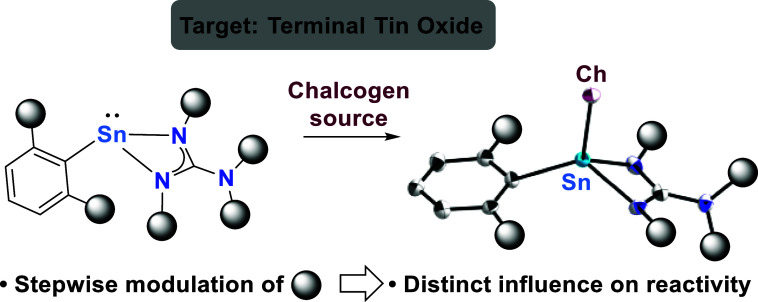

In the pursuit of terminal tin chalcogenides, heteroleptic stannylenes bearing terphenyl- and hexamethyldisilazide ligands were reacted with carbodiimides to yield the respective guanidinato complexes. Further supported by quantum chemical calculations, this revealed that the *iso*-propyl-substituted derivative provides the maximum steric protection achievable. Oxidation with elemental selenium produced monomeric terminal tin selenides with four-coordinate tin centers. In reactions with N_2_O as oxygen transfer reagent, silyl migration toward putative terminal tin oxide intermediates gave rise to tin complexes with terminal —OSiMe_3_ functionality. To prevent silyl migration, the silyl groups were substituted with cyclohexyl moieties. This analogue exhibited distinctively different reactivities toward selenium and N_2_O, yielding a 1,2,3,4,5-tetraselenastannolane and chalcogenide-bridged dimeric compounds, respectively.

## Abstract 2

The reactions of heteroleptic terphenyl-/guanidinato stannylenes with chalcogenide sources are being investigated. These investigations reveal a crucial dependency of the substitution pattern of the supporting guanidinato ligands on the observed reactivity. The studies include, among other things, silyl group migration to a putative terminal tin oxide intermediate.

## Introduction

Compounds featuring the carbonyl functionality (R_2_C=O) such as aldehydes, ketones, and amides are fundamental components in organic chemistry. Despite being thermodynamically robust, these functional groups are straightforward to functionalize, given the polarity of the C=O motif. Due to electronegativity differences, their heavier tetrel analogues R_2_E(14)=O (E(14) = silicon, germanium, tin, and lead) exhibit even greater charge separation, which increases down Group 14.^1^ Furthermore, significantly weaker π overlap between oxygen and the heavier Group 14 elements results in terminal E(14)=O double bond fragments which are thermodynamically unstable, and often adopt a polarized/ylidic form (E(14)^+^–O^–^). The inherent charge disparity in heavier Group 14 carbonyl compounds cannot be quenched effectively by π bond formation, resulting in high reactivity. This is frequently manifested in self-quenching through di-, oligo-, and polymerization reactions; as well as inter and intramolecular C–H activation processes.^2^ Consequently, heavier Group 14 carbonyl compounds have been elusive species in the past, leaving ample room for the further development of their chemistry.

**Figure 1:**
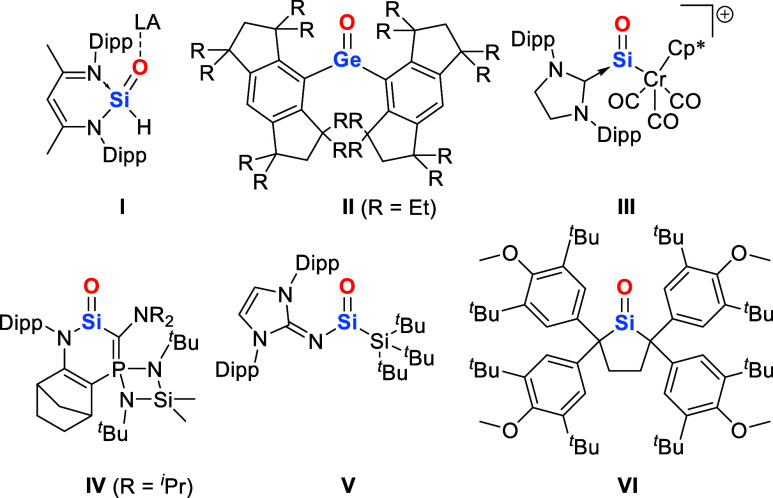
Selected landmark examples of heavier Group 14 carbonyl analogues **I**–**VI** (Dipp = 2,6-^*i*^Pr–C_6_H_3_; LA = Lewis acid).

Over a century ago, Kipping aimed to synthesize the lightest heavier carbonyls, known as silanones (R_2_Si=O). However, the material produced was later identified to be a polysiloxane, a now omnipresent class of polymers and illustrative of one of the typical self-quenching reactivities, *vide supra*.^3^ Despite being detected in low-temperature matrices in the 1980s,^4^ it was not until 2007 that the first stable silacarbonyl compounds were reported, utilizing external Lewis acid and/or Lewis base stabilization (Figure 1, I).^5^ This strategy paved the way for the isolation of main group carbonyl species across the p-block elements.

Interestingly, the first heavier Group 14 analogue of a ketone, devoid of any acid–base stabilization, was reported for germanium instead of silicon. Tamao, Matsuo, and co-workers achieved this milestone in 2012 with the terminal monomeric germanone,^6a^ having paved the way for further examples featuring the terminal Ge=O moiety (Figure 1, II).^6^ Within the next seven years, stable compounds featuring the “free” Si=O functionality were reported by Filippou,^7^ Kato,^8^ and Inoue^9^ (Figure 1, III–V). Iwamoto and co-workers were finally able to tame a cyclic dialkylsilanone, bearing a three-coordinate silicon center with an unperturbed Si=O double bond, utilizing a kinetic stabilization strategy just four years ago (Figure 1, VI).^10^

Such achievements have shifted the perception of these compounds from “laboratory curiosities” to versatile tools for exploring classical carbonyl chemistry with the heavier Group 14 elements and uncovering novel reactivity patterns and applications in oxide ion transfer chemistry.^11^

One intriguing question that remains is whether “true” stannanones/terminal tin oxides are synthetically accessible.^11^ The most closely related isolable complexes in literature involve formal “SnO” and “PbO” units trapped by multiple Lewis acid and Lewis base sites.^12^

Herein, we report on our current progress in isolating terminal tin chalcogenides en route to the isolation of a terminal tin oxide.

## Results and Discussion

**Scheme 1:**
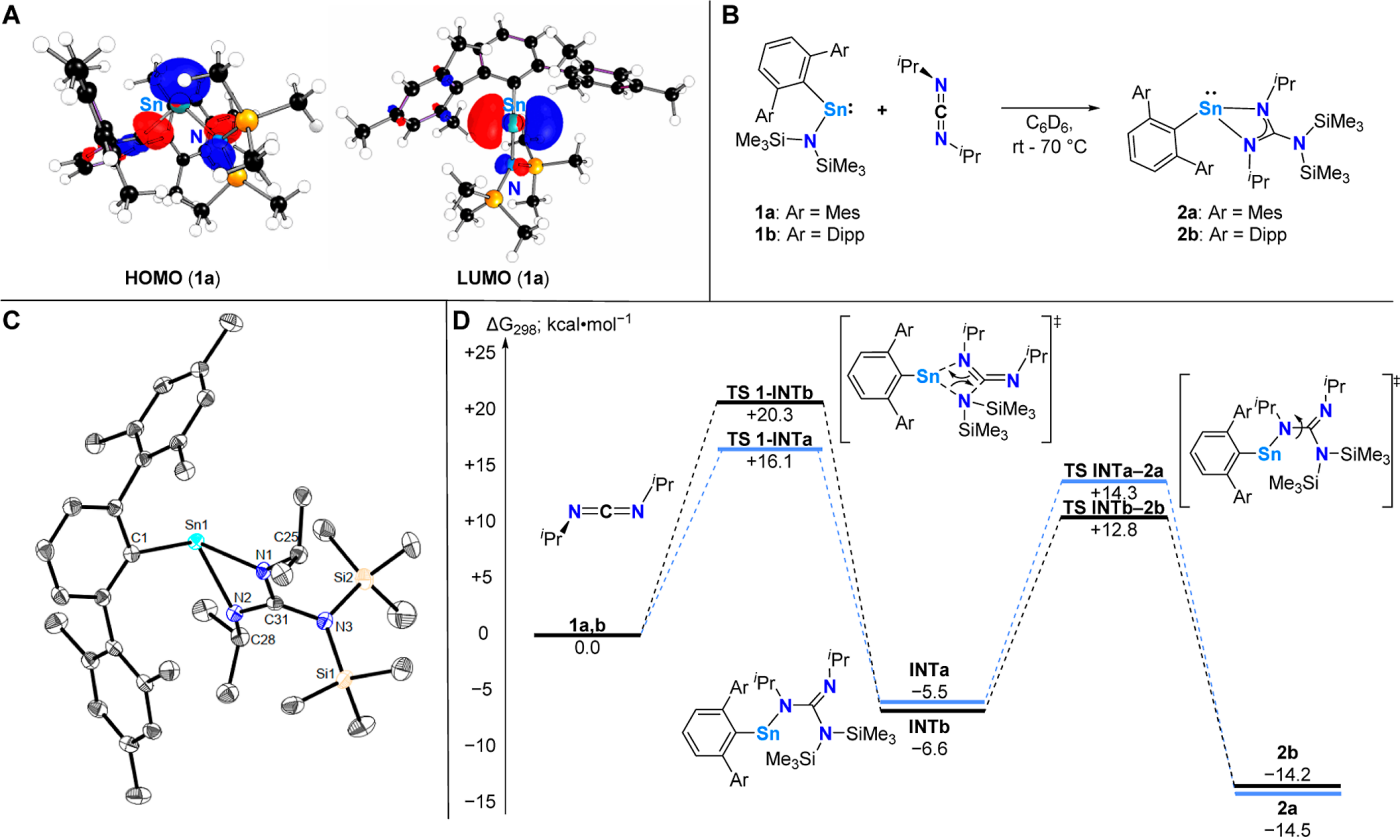
(A): Kohn–Sham Molecular Orbitals of **1a,b** (BP86/Def2-TZVP); (B) Reactivity of **1a**,**b** towards ^*i*^PrN=C=N^*i*^Pr to Give the Heteroleptic Terphenyl-/Guanidinato-Stannylenes **2a,b**; (C) Molecular Structure of ^Mes^TerSn{N(^*i*^Pr)C(N(SiMe_3_)_2_)N(^*i*^Pr)} (**2a**) in the Crystal Anisotropic displacement parameters are drawn at the 50% probability level (hydrogen atoms have been omitted for clarity). Selected bond lengths (Å) and angles (deg): Sn1–N1 2.2017(17), Sn1–N2 2.2678(17), Sn1–C1 2.258(2), Sn1···C31 2.638(2), N1–C31 1.331(3), N2–C31 1.329(3), N3–C31 1.421(3), N1–Sn1–C1 104.57(7), N2–Sn1–C1 114.73(7), N1–Sn1–N2 59.19(6), N1–C31–N2 112.20(18); (D) computed mechanism for the formation of **2** from **1** and *N*,*N*-diisopropylcarbodiimide [BP86-D3BJ/Def2-TZVP/Benzene(PCM)].

Heteroleptic stannylenes, comprising one terphenyl and one hexamethyldisilazido ligand of the general type ^Ar^TerSn{N(SiMe_3_)_2_} [**1a**: Aryl(Ar) = Mes (2,4,6-Me_3_C_6_H_2_), **1b**: Ar = Dipp (2,6-^*i*^Pr_2_C_6_H_3_)], have recently been found to facilitate the isolation of rare instances of terminal stannaphosphenes and stannaimines.^13^ However, when **1a,b** are subjected to typical oxygen transfer reagents, e.g., N_2_O or Me_3_NO, they yield complex reaction mixtures or undergo decomposition. We postulated that a modified ligand set, featuring a three-coordinate tin atom supported by an intramolecular Lewis base, might provide the necessary electronic and steric characteristics to enable the formation of a heteroleptic stannylene capable of generating terminal tin chalcogenides. In this context, upon inspection of the Frontier Kohn–Sham molecular orbitals of **1**, it becomes evident that **1** can act as an ambiphile, capable to react nucleophilically either at the tin or nitrogen lone pair observed in the HOMO, and electrophilically at the tin p-orbital observed in the LUMO, which is the major contributor (77%) to the molecular orbital (Scheme 1, A). This consideration, in conjunction with the well-known behavior of carbodiimides, which tend to formally insert into tetrel–amido bonds due to their propensity to act as nucleophiles at nitrogen and electrophiles at carbon,^14^ further lays the foundation of our rationale.

Accordingly, the reactivity profiles of **1a**,**b** toward a range of carbodiimides RN=C=NR (R = Dipp, Me_3_Si, ^*t*^Bu, ^*i*^Pr) were investigated. The Dipp-, Me_3_Si-, and ^*t*^Bu-substituted derivatives do not show any reactivity with **1a** even after prolonged periods of heating (c.f. Figures S1–S3),^15^^*i*^PrN=C=N^*i*^Pr however readily reacts with both **1a** and **1b** — already starting at room temperature — to solely give the targeted heteroleptic terphenyl-/guanidinato-stannylenes ^Ar^TerSn{N(^*i*^Pr)C(N(SiMe_3_)_2_)N(^*i*^Pr)} (**2a**: Ar = Mes, **2b**: Ar = Dipp) in isolated yields of up to 89% (Scheme 1, **B**).

Given the selectivity for the formation of **2a**,**b** on the nature of the carbodiimide substitution pattern, the overall Gibbs free energy of reactions between **1** and carbodiimides of varying R (R = ^*i*^Pr, ^*t*^Bu, SiMe_3_) were explored computationally by density functional theory at the BP86-D3BJ/def2-TZVP/Benzene(PCM) level of theory (Figure S60, Table S12). This found unanimously that only reactions of R = ^*i*^Pr were exergonic (Δ*G*_298_: ***a***, −14.5 kcal mol^–1^; ***b***, −14.2 kcal mol^–1^), presumably due to the increased steric hindrance producing thermodynamically unfavorable products with strained conformations. The endergonic energies for reactions with carbodiimides featuring R = ^*t*^Bu, SiMe_3_ also correlate well to the experimental findings, which showed no conversion to the respective tin guanidinates.^15^

**2a** and **2b** were characterized by multinuclear nuclear magnetic resonance (NMR) spectroscopy, bulk purity verified by elemental microanalysis, and, in the case of **2a**, single crystal X-ray diffraction (Scheme 1, **C**).

The molecular structure of **2a** shows the three-coordinate tin atom whose coordination environment is distorted trigonal pyramidal [largest bond angle, 114.73(7)° (C1–Sn1–N2)]. The Sn1–N1 and Sn1–N2 bond lengths of 2.2017(17) and 2.2678(17) Å, respectively, differ significantly and are both above respective single bond covalent radii (2.11 Å).^16^ Sn1–N2 is elongated compared to other structurally characterized tin guanidinato complexes (c.f. 2.138(3) and 2.185(3) Å in [Sn(Cl){N(*p*-tolyl)C(N(SiMe_3_)_2_)N(*p*-tolyl)}]_2_^17^). The central quaternary carbon atom of the guanidinato ligand is sp^2^-hybridized (Σ∠ = 359.8°). The characteristic shortening of the carbon–nitrogen bonds of the coordinating κ^2^-*N*,*C*,*N* moiety of guanidinato ligands compared to the exocyclic carbon–nitrogen bond is clearly pronounced [N1–C31 1.331(3) Å, N2–C31 1.329(3) Å, N3–C31 1.421(3) Å].

The solution NMR data of **2a**,**b** exhibit two signals for the SiMe_3_ groups in the respective ^1^H [δ = 0.03 and 0.18 ppm (**2a**)] and ^29^Si{^1^H} [δ = 3.9 and 7.8 ppm (**2a**)] NMR spectra, indicating hindered rotation of the N(SiMe_3_)_2_ moiety. Characteristic of guanidinato ligands is the ^13^C{^1^H} NMR chemical shift of the central quaternary carbon atom which for **2a**,**b** are observed at δ^13^C{^1^H} = 159.5 (**2a**) and 161.4 (**2b**) ppm, respectively. This is in good accordance with previously reported tin complexes bearing this ligand class.^14,17^ The ^119^Sn{^1^H} NMR chemical shifts of **2a**,**b** are located at δ^119^Sn{^1^H} = 90.5 (**2a**) and 95.2 (**2b**) ppm, being significantly shifted to lower field when compared to literature guanidinato-tin complexes featuring additional amido ligands (δ^119^Sn{^1^H} < −110 ppm^17^), thus demonstrating the influence of the strongly σ-donating terphenyl ligand on the tin atom.

To the best of our knowledge, no studies have been reported to elucidate a mechanism for this transformation. Based on the convenient orbital overlap between the HOMO and LUMO of **1** and the carbodiimide, a metathesis-type process was found to proceed first via a *Sn*,*N*,*C*,*N* heterocyclic transition state (ΔG_298_^⧧^: Ar = Mes, + 16.1 kcal mol^–1^; Ar = Dipp, +20.3 kcal mol^–1^) to produce intermediate **A**, featuring a *Sn*,*N*,*C*,*N* chain (Δ*G*_298_: Ar = Mes, −5.5 kcal mol^–1^; Ar = Dipp, −6.6 kcal mol^–1^) (Scheme 1, **D**). Intermediate **A** then undergoes a *ca*. 90° torsion around the *Sn*,*N*,*C*,*N* dihedral (Δ*G*_298_^⧧^: Ar = Mes, + 14.3 kcal mol^–1^; Ar = Dipp, + 12.8 kcal mol^–1^) to form **2** (Overall Δ*G*_298_: Ar = Mes, −14.5 kcal mol^–1^; Ar = Dipp, −14.2 kcal mol^–1^). Although intermediate **A** is lower in energy than the starting materials, the similarity in energies between **TS1** and **TS2** would preclude its observation even at low temperatures.

**Scheme 2:**
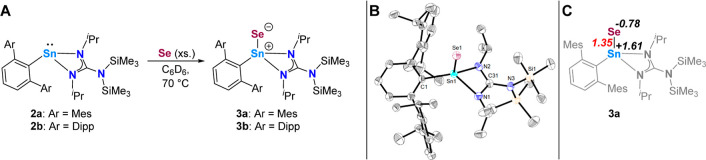
(A) Syntheses of the Terminal Tin Selenides **3a**,**b**; (B) Molecular Structure of ^Dipp^TerSn(Se){N(^*i*^Pr)C(N(SiMe_3_)_2_)N(^*i*^Pr)} (**3b**) in the Crystal Anisotropic displacement parameters are drawn at the 50% probability level (hydrogen atoms have been omitted for clarity). Selected bond lengths (Å) and angles (deg): Sn1–Se1 2.3818(6), Sn1–N1 2.170(4), Sn1–N2 2.151(4), Sn1–C1 2.184(4), Sn1···C31 2.594(3), N1–C31 1.329(6), N2–C31 1.344(6), N3–C31 1.399(6), N1–Sn1–C1 123.30(16), N2–Sn1–C1 122.82(16), N1–Sn1–N2 61.49(15); (C) tin–Selenium Wiberg bond index (shown in red) and selected natural atomic charges (shown in black) of **3a**.

In order to assess the suitability of the selected ligand framework for stabilizing terminal chalcogenides in a broader context, **2a**,**b** were reacted with stoichiometric amounts of elemental selenium (Scheme 2, A). Although no reactions could be observed at room temperature, heating of the reaction mixtures to 70 °C for several hours results in consumption of both starting materials, color changes to a more intense yellow, and main formation of single products according to ^1^H NMR spectroscopy. It is worth noting that **2a** does not react with elemental tellurium in benzene or tetrahydrofuran neither at room temperature nor elevated temperatures of up to 100 °C.

Crystals of **3b** suitable for single crystal X-ray diffraction analysis were obtained from a saturated *n*-hexane solution at −30 °C, confirming the formation of a terminal tin selenide with a four-coordinate tin center, whose coordination environment is best described as distorted tetrahedral (τ_4_ = 0.79^18^) (Scheme 2, B). The terminal tin–selenium bond length of 2.3818(6) Å is in good agreement with the double bond covalent radii of the respective elements (Σ_cov_Sn–Se 2.56 Å, Σ_cov_Sn = Se 2.37 Å) and is on the shorter end of the tin–selenium bonds reported to date (c.f. Figure S57 and Tbt(Ditp)SnSe 2.373(3) Å;^19d^ Tbt = 2,4,6-tris[bis(trimethylsilyl)methyl]phenyl, Ditp = 2,2′-diisopropyl-*m*-terphenyl-2′-yl). Generally, **3a**,**b** account for the first monomeric and neutral terminal tin selenides with four-coordinate tin centers, with most literature-known derivatives bearing five-coordinate tin centers.^14c,19,20^ The aforementioned example with tin in a trigonal planar coordination environment is not obtained directly from the reaction of the respective stannylene precursor with selenium due to the initial formation of a 1,2,3,4,5-tetraselenastannolane and has to be further reacted with three equivalents of triphenylphosphine.^19d^ Although the structural parameters of **3b** are indicative of pronounced double bond character of the Sn–Se bond and are usually the preferred way to describe these complexes in the literature,^14c,19^ the bonding of **3a** was investigated by computational methods. A Wiberg bond index of 1.35 and natural charges of +1.61 (Sn) and −0.78 (Se) were found, indicating a polarized interaction with a formal order between a single and double bond (Scheme 2, C). Furthermore, natural bond orbital (NBO) analysis was employed and found only two NBOs to describe the Sn–Se interaction with a total of 2.05 electrons, both of which polarized toward Se (60.8%), indicating a zwitterionic single bond. This is consistent with the Sn–N interaction in our previously reported stannaimine systems, taking into account the difference in electronegativity between N and Se.^13b^ There are also three NBOs describing lone pairs at Se, accounting for six electrons, one of which is delocalized (86.7% localization on Se). Natural localized molecular orbital analysis shows that a significant amount (12.3%) of the delocalization tail resides in a p-orbital overlap with Sn, explaining the increased Sn–Se bond order above what would be expected for a single bond.

The description of a Sn^δ+^–Se^δ−^ single bond with partial charges is in agreement with a weak π-acceptor character of the tin atom and is usually reflected by an upfield shift in the ^77^Se NMR spectrum (shielded selenium).^19k^ The observed ^77^Se and ^119^Sn NMR chemical shifts of **3a**,**b** are observed at δ^77^Se = −134.6 (**3a**) and −100.6 (**3b**) ppm and δ^119^Sn = −165.3 (**3a**) and −173.1 (**3b**) ppm,^21^ respectively, thus being in the same range as reported for cationic tristannaselone imido clusters, with four-coordinate tin atoms (c.f. δ^77^Se = −172 ppm and δ^119^Sn = −133 ppm).^19e^

Having shown that the chosen supporting ligand set at tin is capable of stabilizing terminal tin selenides, terminal tin oxide complexes were targeted next. By pressurizing a C_6_D_6_ solution of **2a**,**b** with 1 bar of nitrous oxide at room temperature, and following the reaction by ^1^H NMR spectroscopy, clean formations of single species over the course of approximately 5 h are observed (Figures S26 and S30).^15^ Subsequent workup led to the isolation of colorless solids and liquid injection field desorption ionization mass spectrometry (LIFDI-MS) of the ^Mes^Ter-substituted derivative is in agreement with the envisioned net oxygen transfer to precursors **2a**,**b**.^15^

The ^119^Sn NMR chemical shifts of the obtained compounds are upfield shifted [δ^119^Sn = −209.9 (**4a**) and −203.7 (**4b**) ppm] when compared to the starting material [δ^119^Sn = 90.5 (**2a**) and 95.2 (**2b**) ppm] and are in the same range as observed for terminal selenides **3a**,**b** (*vide supra*). Although the solution NMR and LIFDI-MS data generally support the formation of terminal tin oxides, the ^29^Si{^1^H} NMR data indicate different product formation. For the starting material **2a**,**b**, as well as the terminal tin selenides **3a**,**b**, the ^29^Si{^1^H} NMR spectra each exhibit two signals in close proximity, as expected when both trimethylsilyl groups are located at nitrogen [δ^29^Si{^1^H} = 3.9 and 7.8 ppm (**2a**), 4.7 and 7.8 ppm (**2b**), 4.7 and 10.1 ppm (**3a**), 5.5 and 10.9 (**3b**) ppm]. In contrast, the ^29^Si{^1^H} NMR of the newly obtained compounds **4a**,**b** show one signal which is significantly upfield shifted, indicative of different chemical environments of the two silyl moieties [δ^29^Si{^1^H} = −23.5 and 10.9 ppm (**4a**), – 23.5 and 10.1 ppm (**4b**)].

**Scheme 3:**
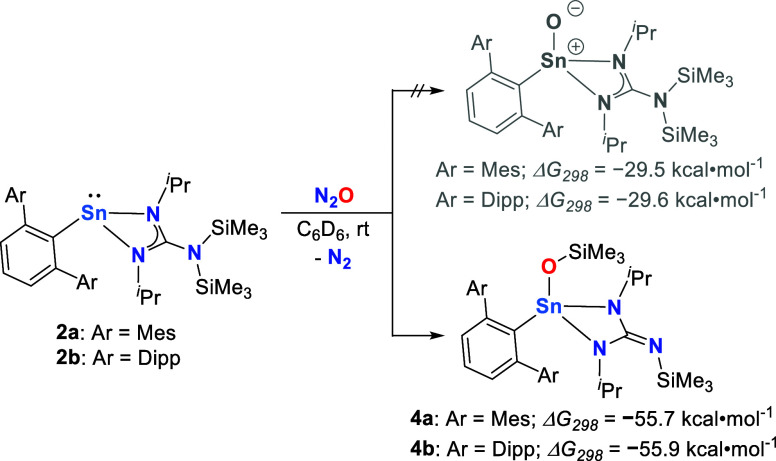
Reaction of **2a**,**b** with N_2_O to Give **4a**,**b** and Overall Reaction Free Energy from **2a**,**b**

**Figure 2:**
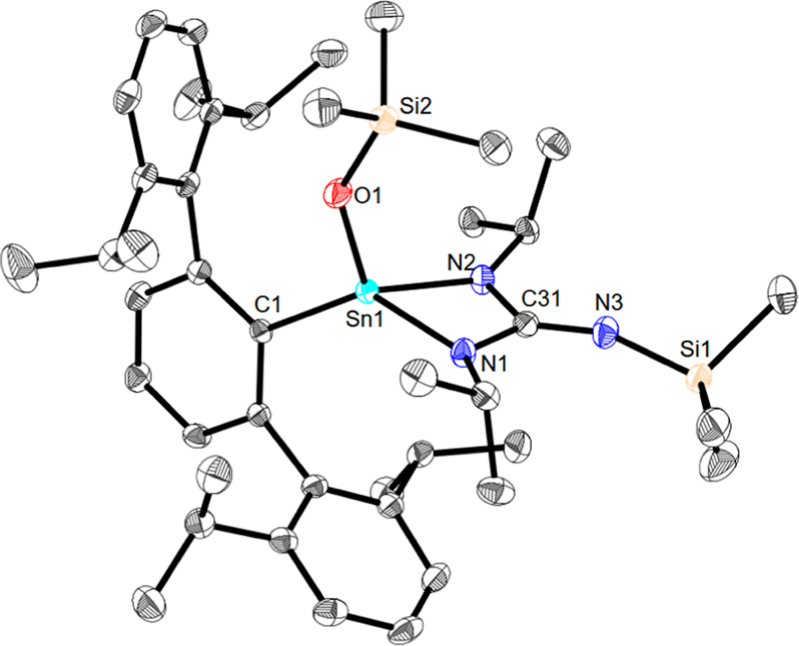
Molecular structure of ^Dipp^TerSn(OSiMe_3_){N(^*i*^Pr)C(=NSiMe_3_)N(^*i*^Pr)} (**4b**) in the crystal. Anisotropic displacement parameters are drawn at the 50% probability level (hydrogen atoms have been omitted for the sake of clarity). Selected bond lengths (Å) and angles (deg): Sn1–O1 1.9427(13), Sn1–N1 2.0466(15), Sn1–N2 2.0389(15), Sn1–C1 2.1373(17), Sn1···C31 2.5587(18), N1–C31 1.396(2), N2–C31 1.400(2), N3–C31 1.263(2), N3–Si1 1.6762(16), N1–Sn1–C1 134.23(6), N2–Sn1–C1 118.46(6), N1–Sn1–N2 65.94(6), and N1–C31–N2 105.33(14).

This is confirmed by the results of single crystal X-ray diffraction of compound **4b**, clearly demonstrating the formation of compounds with Sn–O–SiMe_3_ functionalities, and due to silyl migration from the ligand nitrogen to oxygen, the monoanionic guanidinato ligands in **2a**,**b** are dianionic ligands in **4a**,**b** (Scheme 3 and Figure 2). Computational investigation found that the observed products are significantly thermodynamically favored over the targeted terminal oxides by Δ*G*_298_: ***a***, −26.2 kcal mol^–1^ and ***b***, −26.3 kcal mol^–1^.

The tin–nitrogen bond lengths of 2.0466(15) Å (Sn1–N1) and 2.0389(15) Å (Sn1–N2) are approximately 10% shorter than in the starting material, and the exocyclic nitrogen–carbon bond length of 1.263(2) Å (N3–C31) is typical of the formed double bond. The bond length of the newly formed tin–oxygen moiety [1.9427(13) Å] is 10% shorter than the sum of the related single bond covalent radii (Σ_cov_Sn–O 2.03 Å).

The formal 1,4-silyl migration observed in this study is suggested to occur through a putative terminal tin oxide intermediate, a hypothesis supported by comparable silyl migrations observed in the context of terminal silanones and in our recently reported stannaimine study.^9a,13b,22^

**Scheme 4:**
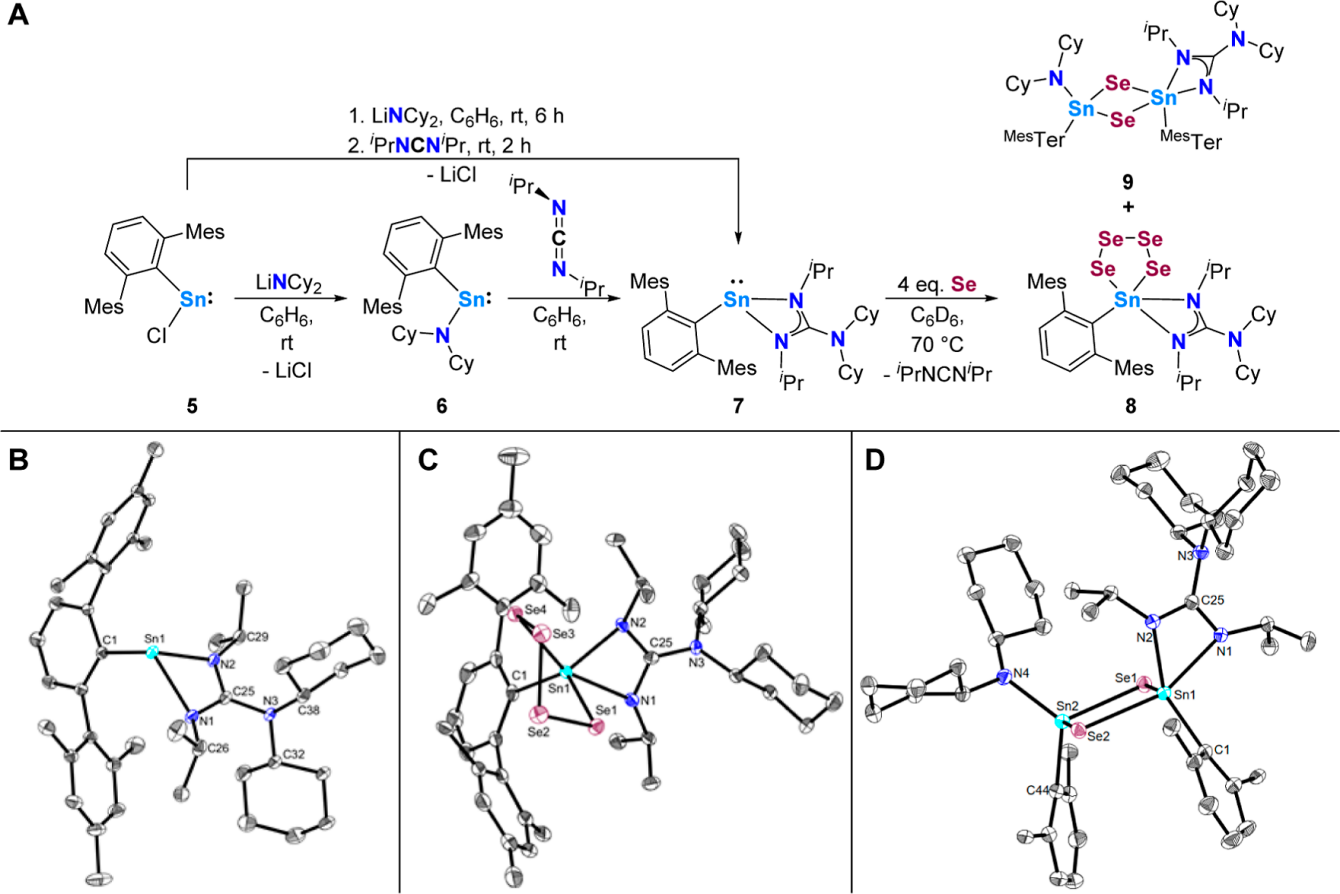
(A) Two-step and One-Pot Synthesis of the Heteroleptic Stannylene **7** and Its Reactivity with Elemental Selenium to Give **8**; (B–D) Molecular Structures of ^Mes^TerSn{N(^*i*^Pr)N(Cy_2_)C(^*i*^Pr)} (**7**), ^Mes^TerSn(Se_4_){N(^*i*^Pr)C(NCy_2_)N^*i*^Pr} (**8**), and ^Mes^TerSn(NCy_2_)(μ-Se_2_)Sn{N(^*i*^Pr)C(NCy_2_)N(^*i*^Pr)}^Mes^Ter (**9**) in the Crystal Anisotropic displacement parameters are drawn at the 50% probability level (hydrogen atoms, mesityl functionalities, second molecule (compound **7**) and lattice solvent (compound **7** and **9**) have been omitted for clarity) selected bond lengths (Å) and angles (deg): (B) Sn1–N1 2.2654(12), Sn1–N2 2.1871(13), Sn1···C25 2.6478(15), N1–C25 1.3377(19), N2–C25 1.3250(18), N3–C25 1.4179(18), N1–Sn1–C1 111.83(5), N2–Sn1–C1 99.20(5), N1–Sn1–N2 59.56(5), N1–C25–N2 112.39(12); (C) Sn1–Se1 2.5654(7), Sn1–Se4 2.6520(6), Sn1–N1 2.315(3), Sn1–N2 2.152(4), Sn1–C1 2.183(4), Se1–Se2 2.3276(7), Se2–Se3 2.3244(7), Se3–Se4 2.3400(8), N1–C25 1.324(5), N2–C25 1.354(5), N3–C25 1.391(5), Se1–Sn1–Se4 99.89(2), N1–Sn1–C1 98.69(14), N2–Sn1–C1 120.96(16), N1–Sn1–N2 59.64(13), N1–C25–N2 112.4(4); (D) Sn1–Se1 2.5582(5), Sn1–Se2 2.6315(4), Sn1–N1 2.2877(16), Sn1–N2 2.1543(16), Sn1–C1 2.2132(19), Sn2–Se1 2.5542(4), Sn2–Se2 2.5224(5), Sn2–N4 2.0452(17), Sn2–C44 2.1975(19), C25–N1 1.314(2), C25–N2 1.347(2), C25–N3 1.418(2).

Given this reaction behavior, our focus shifted to a heteroleptic terphenyl-/guanidinato-tin system devoid of silyl groups. Initially, we synthesized the heteroleptic terphenyl-/dicyclohexylamido- stannylene **6** through a salt metathesis reaction between ^Mes^TerSnCl (**5**)^23^ and freshly prepared LiNCy_2_ (Scheme 4, A).^15^ Characterization of **6** was carried out in solution using NMR spectroscopy and in the solid state by single crystal X-ray diffraction.^15^

Additionally, **6** was found to react with *N*,*N*′-diisopropylcarbodiimide, yielding the corresponding guanidinato complex **7**. Notably, this compound can be conveniently synthesized in a one-pot procedure starting from compound **5** (Scheme 4, A). The analytical data for **7** show only marginal differences from those of **2a**,**b**, so that a detailed discussion is omitted at this stage (cf. Scheme 4, B for the structural data).^17^

Interestingly, when **7** was reacted with equimolar amounts of elemental selenium at elevated temperatures (reaction did not initiate at room temperature), most of the compound remained unreacted as confirmed by ^1^H NMR spectroscopy. Simultaneously, the elemental selenium was entirely consumed, as demonstrated by the absence of any remaining gray precipitate in the reaction mixture. Accordingly, **7** was reacted with an excess of elemental selenium until **7** was completely consumed (Figure S47). From the respective crude ^1^H NMR spectrum, it was already evident that small amounts of ^*i*^PrN=C=N^*i*^Pr were liberated. After multiple crystallization attempts, we eventually succeeded in growing both yellow and orange crystals suitable for single crystal X-ray diffraction. The orange crystalline material revealed the formation of 1,2,3,4,5-tetraselenastannolane **8** (Scheme 4, A,C).

The structural data within the SnSe_4_ linkage is in good agreement with the also structurally characterized 1,2,3,4,5-tetraselenastannolane Tbf(Mes)SnSe_4_ (Sn–Se_av_ 2.58 Å, Se–Se_av_ 2.31 Å).^19d,24^ The coordination environment at tin is best described as square pyramidal, according to the structural parameter τ_5_ (0.04).^25^ The identity of the yellow crystalline material explains why free ^*i*^PrN=C=N^*i*^Pr was detected in the crude NMR spectra of the reaction and is linked to the formation of the selenium-bridged dimer (1,3,2,4-diselenadistannetane) ^Mes^TerSn(NCy_2_)(μ-Se_2_)Sn{N(^*i*^Pr)C(NCy_2_)N(^*i*^Pr)}^Mes^Ter (**9**) with the terphenyl substituents in a *cis* configuration (Scheme 4, A,D). To the best of our knowledge, the release of carbodiimides from guanidinato ligands upon addition of another substrate has not been observed so far. Although **8** and **9** invariably cocrystallized in our hands, small amounts of **8** could be separated and further analyzed by elemental microanalysis, ^1^H and ^119^Sn{^1^H} NMR spectroscopy (δ119Sn{^1^H} = −252.9 ppm) (Figures S46, S48 and S49).^15^

The reactivity of **2a**,**b** and **7** toward elemental selenium differs significantly despite comparatively small differences in backbone substitution patterns.

In this context, we finally investigated the reactivity of **7** toward N_2_O.

**Scheme 5:**
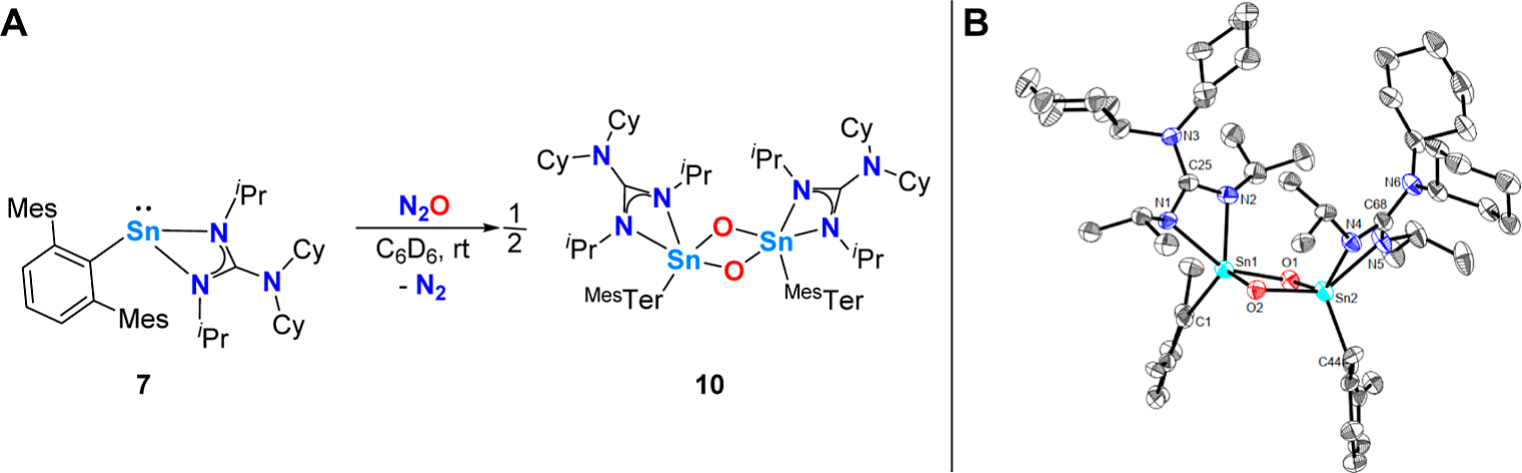
(A) Reaction of **7** with N_2_O to Give **10**; (B) Molecular Structure of ^Mes^TerSn{N(^*i*^Pr)C(NCy_2_)N^*i*^Pr}(μ-O_2_)Sn{N(^*i*^Pr)C(NCy_2_)N^*i*^Pr}^Mes^Ter (**10**) in the Crystal Anisotropic displacement parameters are drawn at the 50% probability level (hydrogen atoms, mesityl functionalities, and lattice solvent have been omitted for clarity). Selected bond lengths (Å) and angles (deg): Sn1–O1 2.039(3), Sn1–O2 1.984(3), Sn1–N1 2.269(3), Sn1–N2 2.140(3), Sn1–C1 2.185(4), Sn2–O1 1.988(3), Sn2–O2 2.052(3), Sn2–N4 2.160(3), Sn2–N5 2.236(4), Sn2–C44 2.193(5), N1–C25 1.338(5), N2–C25 1.350(5), N3–C25 1.393(5), N4–C68 1.358(5), N5–C68 1.329(5), N6–C68 1.395(5), O1–Sn1–O2 82.10(11), O1–Sn2–O2 81.67(11), Sn1–O1–Sn2 97.73(11), Sn1–O2–Sn2 97.39(11).

The reaction is overall clean and results in the formation of a single product according to ^1^H NMR spectroscopy (Figure S52). Removal of all volatile components and recrystallization from *n*-hexane yields a colorless microcrystalline solid which was first analyzed by LIFDI mass spectrometry and is in agreement with the formation of the oxygen bridged dimer ^Mes^TerSn{N(^*i*^Pr)N(Cy_2_)C(^*i*^Pr)}(μ-O_2_)Sn{N(^*i*^Pr)-N(Cy_2_)C(^*i*^Pr)}^Mes^Ter (1,3,2,4-dioxadistannetane) (**10**) (Scheme 5, A).^15^ The *cis* configuration of both, the terphenyl and guanidinato ligands, could further be verified by single crystal X-ray diffraction with crystals obtained from a saturated *n*-pentane solution of **10** stored at −30 °C (Scheme 5, B).

Computational investigation found that the dimerization of the proposed terminal oxide intermediate to dimer 10 is exergonic by Δ*G*_298_ = −26.2 kcal mol^–1^. The observed *cis* configuration is, albeit only slightly, thermodynamically favored over its trans configuration by Δ*G*_298_ = −1.4 kcal mol^–1^.

## Conclusions

We present the reactions of heteroleptic terphenyl-/amido-substituted stannylenes **1a**,**b** with carbodiimides. Investigated through combined experimental and computational studies, **1a**,**b** react with the *iso*-propyl-substituted derivative, yielding the corresponding guanidinato complexes **2a**,**b**. Sterically more demanding carbodiimides are unable to undergo a comparable metathesis-type reaction. Consequently, **2a**,**b** offers maximum steric protection, which should ultimately facilitate the targeted synthesis of terminal tin chalcogenides.

Compounds **2a**,**b** react cleanly with elemental selenium to give respective terminal tin selenides **3a**,**b**. By contrast, in reactions with N_2_O as an oxygen transfer reagent, instead of yielding a terminal tin oxide, silyl migration of the guanidinato ligand to the putative tin–oxygen moiety occurs, yielding the corresponding tin complexes **4a**,**b**, bearing the Sn–OSiMe_3_ functionality.

To prevent silyl migration, tin compound **7** with an aliphatic cyclohexyl substitution pattern instead of SiMe_3_ groups was successfully synthesized. Reacting **7** with elemental selenium does not lead to the formation of a terminal tin selenide and gives rise to both the 1,2,3,4,5-tetraselenostannolane **8** and 1,3,2,4-diselenadistannetane **9**, the formation of which is accompanied by the release of ^*i*^PrN=C=N^*i*^Pr.

The reaction of **7** with N_2_O also deviates significantly from those of **2a**,**b**, leading to the clean formation of the 1,3,2,4-dioxadistannetane **10**, showing that comparatively small changes in substitution have a significant influence on the reaction outcome and further emphasize the difficulties in stabilizing a compound with a terminal tin–oxygen bond.

The obtained compounds have been comprehensively characterized in solution and in the solid state, including single crystal X-ray diffraction of one compound of each accessed class. The bonding situation in the first examples of four-coordinate terminal tin selenide **4a**,**b** was further analyzed by quantum chemical calculations.

## Supporting Information Available

Experimental, crystallographic, and computational details (PDF)

## Notes

Additional references are cited in the Supporting Information.^26−41^

## Notes

Note on safety precautions: compounds containing tin and selenium may pose risks to health and the environment if mishandled or improperly disposed of, and exposure to these compounds could result in adverse health effects. Therefore, manipulations and reactions should be conducted under inert conditions in monitored fume hoods or in an inert-atmosphere dry box while wearing appropriate protective clothing.
