# Supplementary material for: En Route to a Molecular Terminal Tin Oxide
Source: Inorg Chem. 2024 Apr 10;63(16):7455–63. doi: 10.1021/acs.inorgchem.4c00598 (PMC11040714; doi:10.1021/acs.inorgchem.4c00598)
Supplement: Supplementary file 1 — ic4c00598_si_001.pdf [file ic4c00598_si_001.pdf]

Supporting Information

En Route to a Molecular Terminal Tin Oxide

Leon Kreßner,[a] Daniel Duvinage,[b] Pim Puylaert,[b] Nico Graw,[a] Regine Herbst-Irmer,[a] Dietmar Stalke,[a] Oliver P. E. Townrow,*[c] Malte Fischer*[a]

[a] Institut für Anorganische Chemie, Georg-August-Universität Göttingen, Tammannstraße 4, D-37077 Göttingen (Germany)
email: malte.fischer@uni-goettingen.de
[b] Institut für Anorganische Chemie und Kristallographie, Universität Bremen, Leobener Str. 7, D-28359 Bremen (Germany)
[c] Inorganic and Organometallic Chemistry, Friedrich-Alexander-Universität Erlangen-Nürnberg, Egerlandstraße 1, D-91058 Erlangen (Germany)
email: oliver.townrow@fau.de

Table of Contents

General Considerations **S2**

Synthesis and Characterization of Compounds **S3**

Crystallographic Details **S70**

Computational Details **S74**

References **S156**

General Considerations

Materials and Synthetic Methods

All manipulations of air- and moisture-sensitive materials were carried out using standard Schlenk-line and glovebox techniques under an inert atmosphere of argon or dinitrogen. Solvents were purified by a Solvent Purification System, degassed by sparging with argon and stored over 3 Å molecular sieves. The carbodiimides were freeze-pump-thaw degassed three times prior to use and stored over 3 Å molecular sieves in the glovebox. $^{Mes}TerSn\{N(SiMe_3)_2\}$ (**1a**), $^{Dipp}TerSn\{N(SiMe_3)_2\}$ (**1b**) and $^{Mes}TerSnCl$ (**5**) were synthesized according to literature procedures.[S1,S2] All reactions were operated under an argon atmosphere in an MBraun glovebox with oxygen and water concentrations below 0.1 ppm as monitored by an O_2/H_2O Combi-Analyzer.
Note on safety precautions: Compounds containing tin and selenium may pose risks to health and the environment if mishandled or improperly disposed of, and exposure to these compounds could result in adverse health effects. Therefore, manipulations and reactions should be conducted under inert conditions in monitored fume hoods or in an inert-atmosphere dry box while wearing appropriate protective clothing.

Analytical Methods

NMR spectra were measured in benzene-d_6 (C_6D_6) or toluene-d_8 (C_7D_8) (dried over CaH_2, distilled by trap-to-trap transfer in vacuo, degassed by three freeze-pump-thaw cycles and transferred to the glovebox). NMR samples were prepared under argon in NMR tubes with J. Young Teflon valves. NMR spectra were measured on a Bruker Avance 400 MHz, 500 MHz, and 600 MHz spectrometers. ^{1}H and ^{13}C NMR spectra were referenced internally to residual protio-solvent (^{1}H) or solvent (^{13}C) resonances (C_6D_6: δ_H = 7.16 ppm; δ_C = 128.06 ppm; C_7D_8: 2.08 ppm; δ_C = 20.43 ppm). ^{119}Sn NMR spectra were referenced with respect to $SnMe_4$. ^{77}Se NMR spectra were referenced with respect to Me_2Se. ^{7}Li NMR spectra were referenced with respect to lithium chloride (9.7 M in D_2O). ^{29}Si NMR chemical shifts were obtained from $^{1}H/^{29}Si$ HMBC experiments. LIFDI-MS (JEOL AccuTOF JMS-T100GCV; inert conditions) were measured by the Zentrale Massenabteilung (Fakultät für Chemie, Georg-August-Universität Göttingen). Elemental analyses were obtained from the Analytische Labor (Georg-August-Universität Göttingen) using an Elementar Vario EL 3 analyzer.

Synthesis and Characterization of Compounds

Attempted Reactions of ^{Mes}TerSnN(SiMe$_3$)$_2$ (1a) with RN=C=NR (R = Dipp, Me$_3$Si, ^{t}Bu)

^{Mes}TerSn{N(SiMe$_3$)$_2$} (**1a**) (0.030 g, 0.051 mmol) was dissolved in 0.3 mL of C_6D_6 followed by addition of one equivalent of the respective carbodiimide in 0.3 mL of C_6D_6. The reaction progress was monitored by ^{1}H NMR spectroscopy with the obtained spectra being shown below.

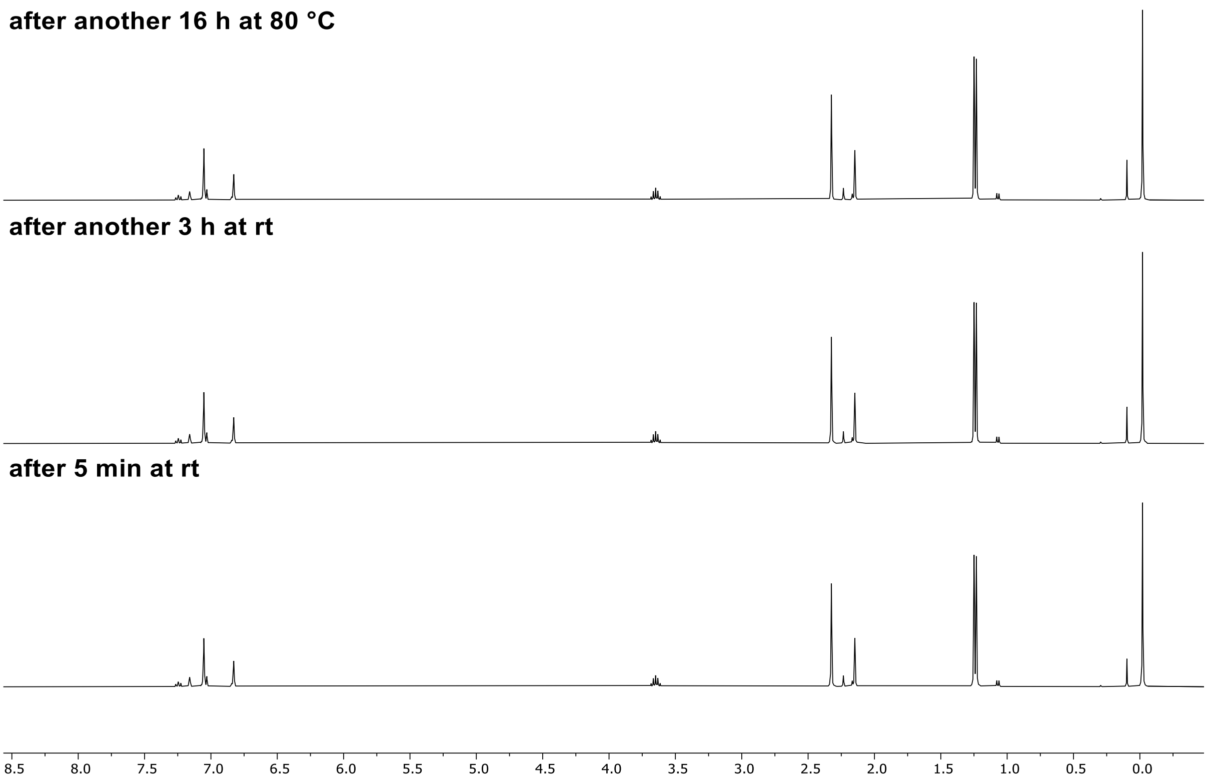

Figure S1. Attempted reaction of ^{Mes}TerSnN(SiMe$_3$)$_2$ (**1a**) with DippN=C=NDipp (400 MHz, C_6D_6, 298 K).

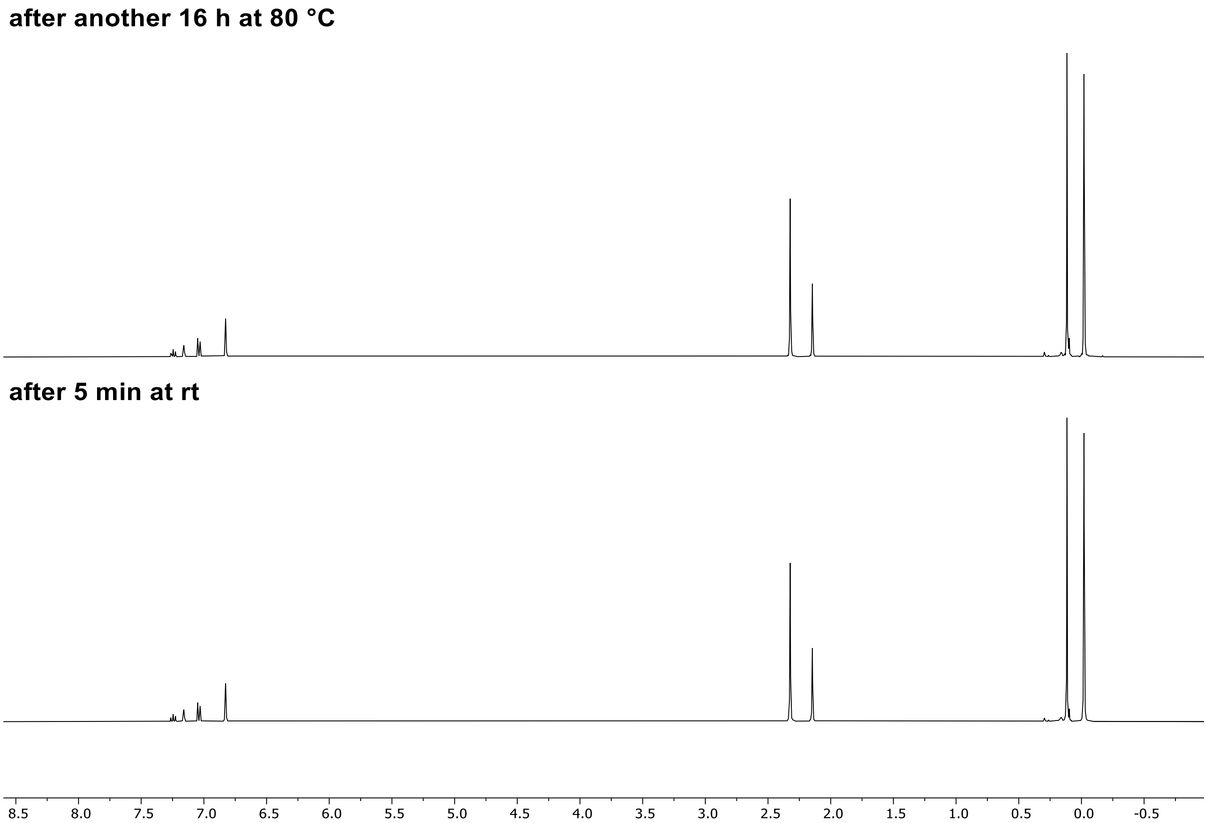

Figure S2. Attempted reaction of ^{Mes}TerSnN(SiMe$_3$)$_2$ (**1a**) with Me$_3$SiN=C=NSiMe$_3$ (400 MHz, C$_6$D$_6$, 298 K).

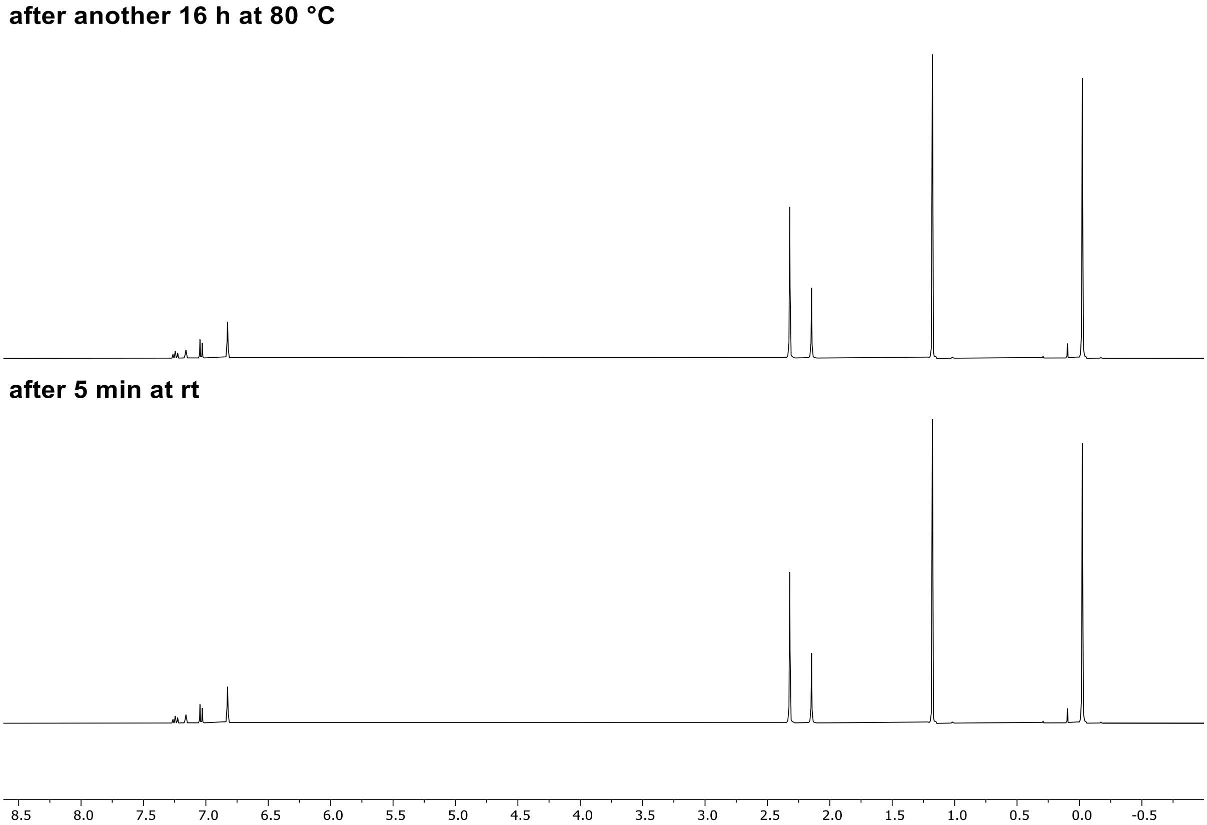

Figure S3. Attempted reaction of ^{Mes}TerSnN(SiMe$_3$)$_2$ (**1a**) with ^{t}BuN=C=N^{t}Bu (400 MHz, C$_6$D$_6$, 298 K).

Reaction of ^{Mes}TerSn{N(SiMe$_3$)$_2$} (1a) and ^{i}PrN=C=N^{i}Pr – Synthesis of ^{Mes}TerSn{N(^{i}Pr)C(N(SiMe$_3$)$_2$)N(^{i}Pr)} (2a)

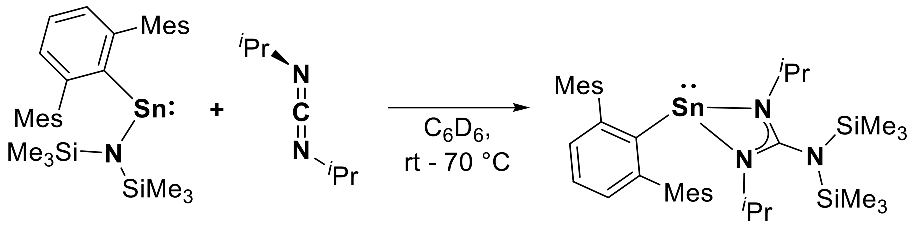

In a typical experiment, ^{Mes}TerSn{N(SiMe$_3$)$_2$} (**1a**) (0.030 g, 0.051 mmol) was dissolved in 0.3 mL of C_6D_6 followed by addition of ^{i}PrN=C=N^{i}Pr (6.4 mg, 0.051 mmol) in 0.3 mL of C_6D_6. The reaction progress was monitored by ^{1}H NMR spectroscopy and after heating of the reaction mixture to 70 °C for one hour, the reaction is finished and results in clean formation of ^{Mes}TerSn{N(^{i}Pr)C(N(SiMe$_3$)$_2$)N(^{i}Pr)} (**2a**) (Figure S4). The end of the reaction is clearly visible by a colour change from clear orange/red to clear yellow. All volatile components were removed under vacuum, the remaining solid was suspended in 0.6 mL of *n*-hexane, filtered and stored at -30 °C to give **2a** as a clear yellow crystalline material. Crystals obtained this way were suitable for single crystal X-ray diffraction.

Note: For all herein reported reactions with ^{i}PrN=C=N^{i}Pr there was no change in reaction outcomes observed when excess amounts were used.

Yield: 0.024 g (0.033 mmol; 65%).
^{1}H NMR (400 MHz, C_6D_6, 298 K): δ = 0.03 (s, 9H, Si(CH$_3$)$_3$), 0.18 (s, 9H, Si(CH$_3$)$_3$), 0.85 (d, $^{3}J_{H,H}$ = 6.3 Hz, 6H, CH(C*H*$_3$)$_2$), 0.93 (d, $^{3}J_{H,H}$ = 6.5 Hz, 6H, CH(C*H*$_3$)$_2$), 2.23 (s, 6H, CH$_3$), 2.35 (s, 12H, CH$_3$), 3.75 (hept, $^{3}J_{H,H}$ = 6.4 Hz, 2H, C*H*(CH$_3$)$_2$), 6.90 (s, 4H, CH$_{Aryl}$), 6.95-6.97 (m, 2H, CH$_{Aryl}$), 7.23-7.27 (m, 1H, CH$_{Aryl}$) ppm.
^{13}C{^{1}H} NMR (101 MHz, C_6D_6, 298 K): δ = 2.5 (Si(CH$_3$)$_3$), 2.6 (Si(CH$_3$)$_3$), 21.2 (CH$_3$), 22.4 (CH$_3$), 24.6 (CH($\underline{C}$H$_3$)$_2$), 27.1 (CH($\underline{C}$H$_3$)$_2$), 45.9 ($\underline{C}$H(CH$_3$)$_2$), 128.1 (CH$_{Aryl}$)*, 129.20 (CH$_{Aryl}$), 129.22 (CH$_{Aryl}$), 136.3 (C$_{q,Aryl}$), 136.4 (C$_{q,Aryl}$), 142.4 (C$_{q,Aryl}$), 148.7 (C$_{q,Aryl}$), 159.5 (C$_q$(N^{i}Pr)$_2$N(SiMe$_3$)$_2$), 172.7 (C$_q$Sn) ppm.
* = overlap with C_6D_6 signal (assigned by ^{1}H/^{13}C HSCQC)
^{29}Si{^{1}H} NMR (80 MHz, C_6D_6, 298 K): δ = 3.9, 7.8 ppm. (assigned by ^{1}H/^{29}Si HMBC)
^{119}Sn{^{1}H} NMR (149 MHz, C_6D_6, 298 K): δ = 90.5 ppm.
MS (LIFDI): m/z calcd. for $C_{37}H_{57}N_3Si_2Sn$: 719.3113; found: 719.2.
EA: Anal. calcd. for $C_{37}H_{57}N_3Si_2Sn$: C, 61.83; H, 7.99; N, 5.85; Found: C, 61.85; H, 8.07; N, 5.83.

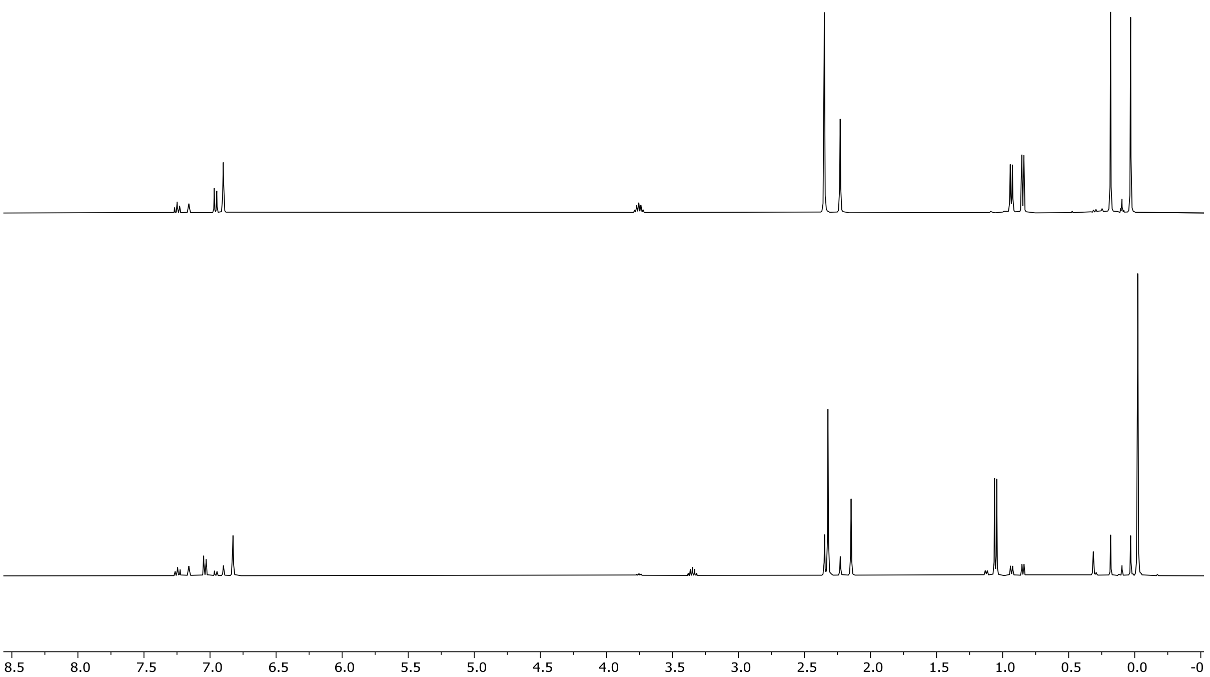

Figure S4. Bottom: ^{1}H NMR spectrum of the reaction of ^{Mes}TerSn{N(SiMe$_3$)$_2$} (**1a**) and ^{i}PrN=C=N^{i}Pr after 15 minutes at room temperature; Top: ^{1}H NMR spectrum after an additional hour at 70 °C (400 MHz, C_6D_6, 298 K).

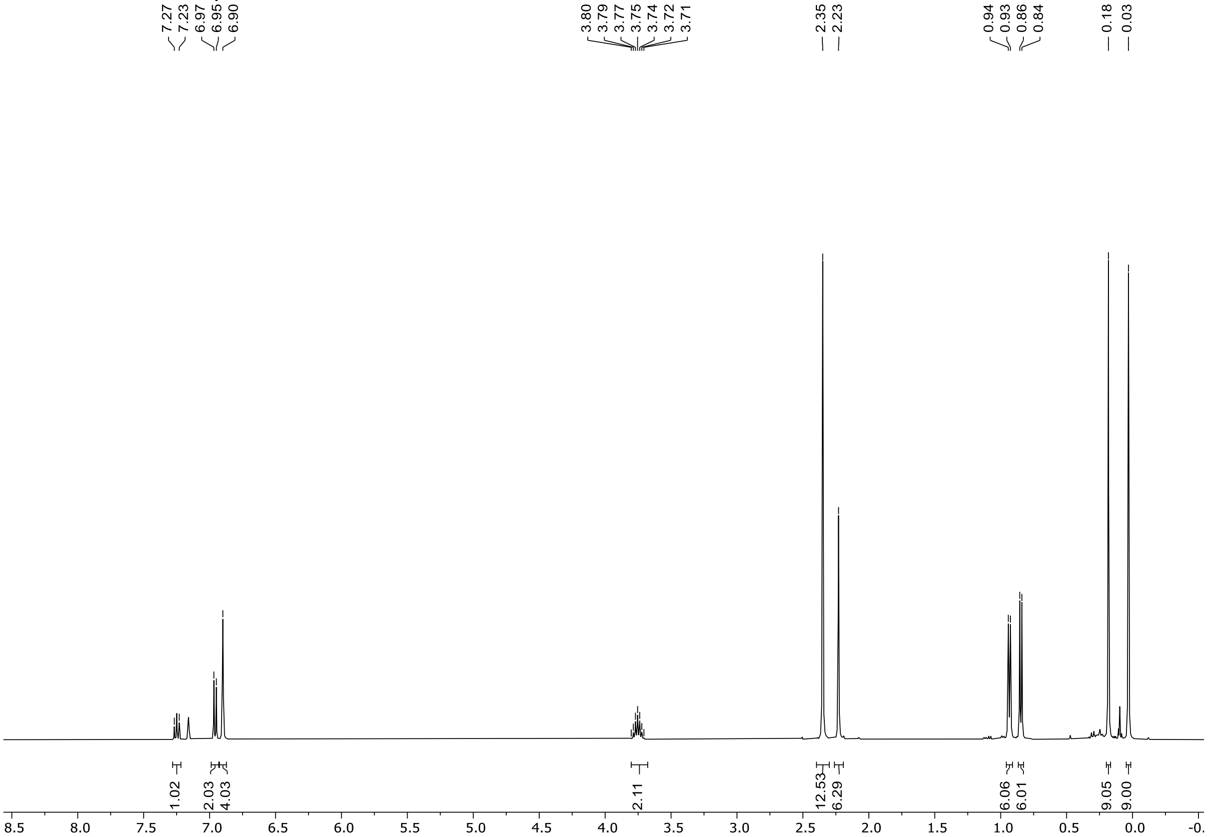

Figure S5. ^{1}H NMR spectrum of ^{Mes}TerSn{N(^{i}Pr)C(N(SiMe$_3$)$_2$)N(^{i}Pr)} (**2a**) (400 MHz, C_6D_6, 298 K).

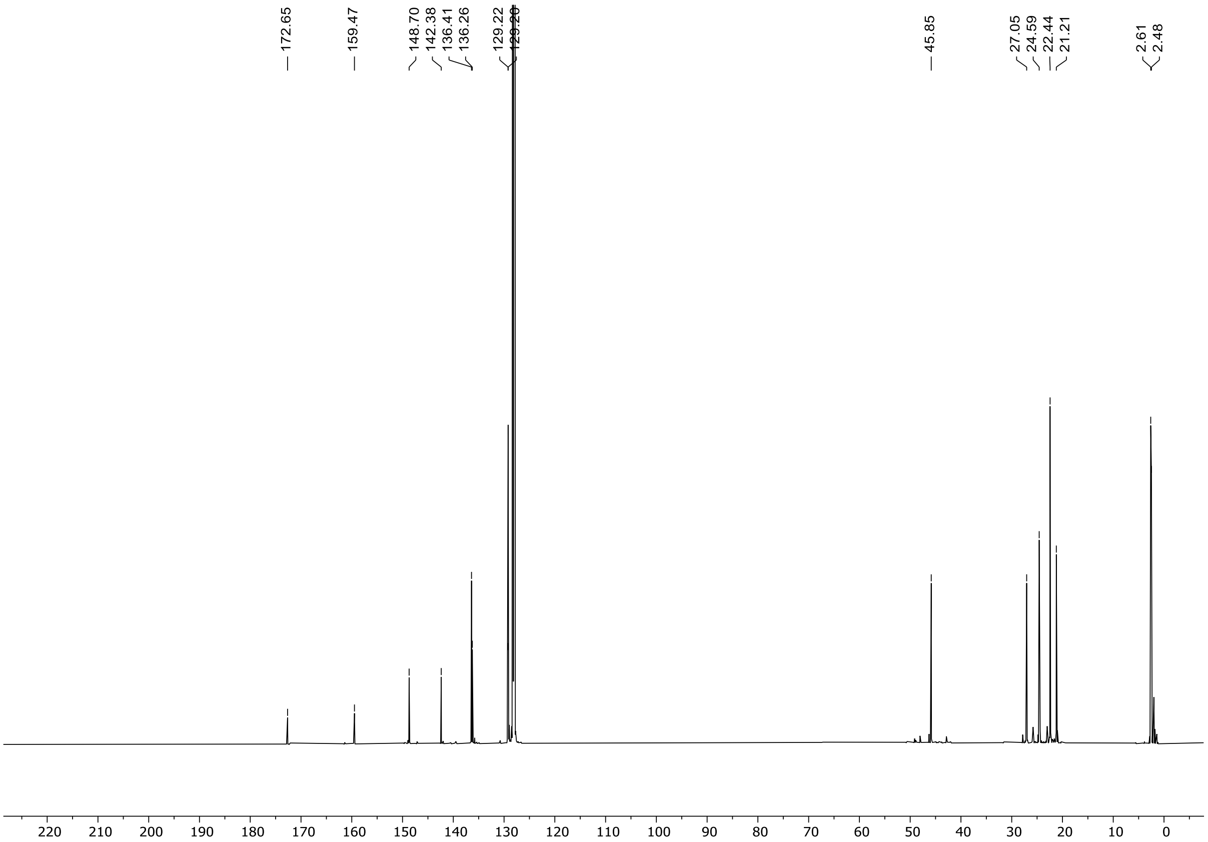

Figure S6. $^{13}C\{^{1}H\}$ NMR spectrum of ^{Mes}TerSn{N(^{i}Pr)C(N(SiMe$_3$)$_2$)N(^{i}Pr)} (**2a**) (126 MHz, C_6D_6, 298 K).

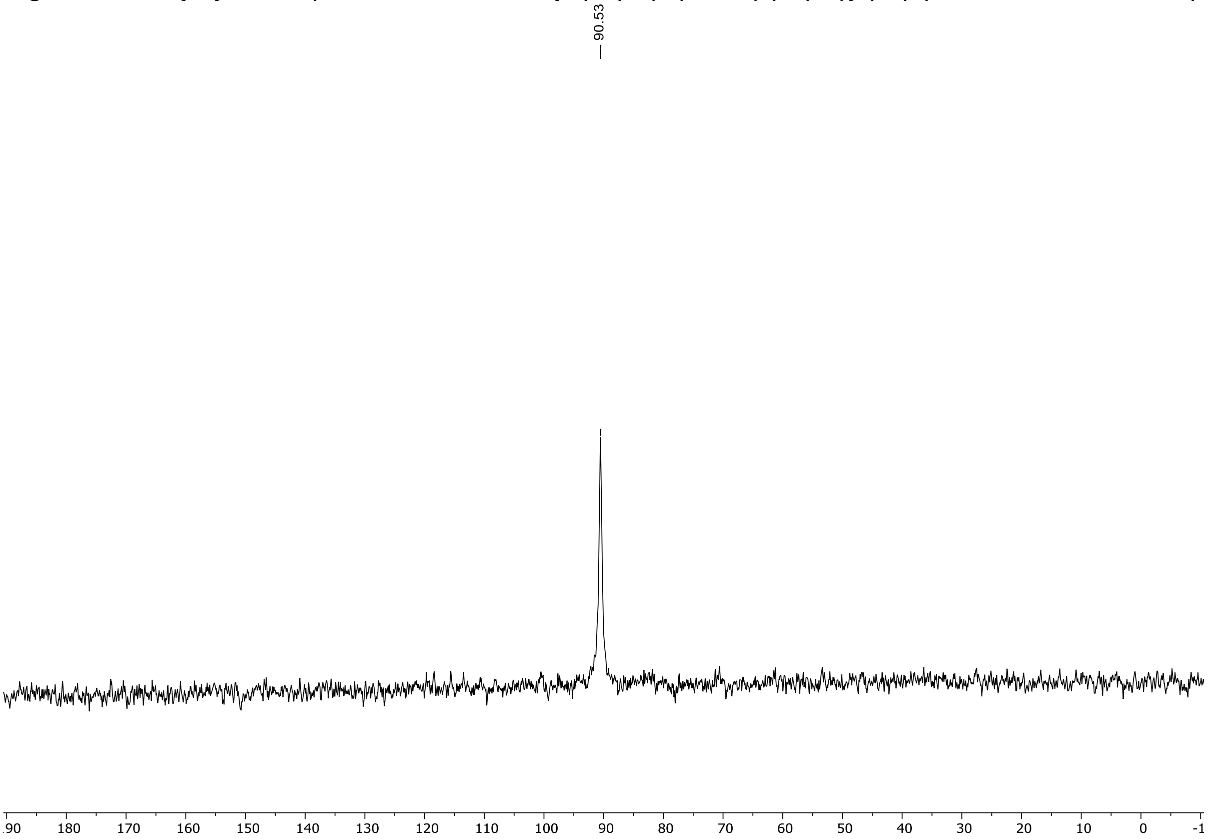

Figure S7. $^{119}Sn\{^{1}H\}$ NMR spectrum of ^{Mes}TerSn{N(^{i}Pr)C(N(SiMe$_3$)$_2$)N(^{i}Pr)} (**2a**) (149 MHz, C_6D_6, 298 K).

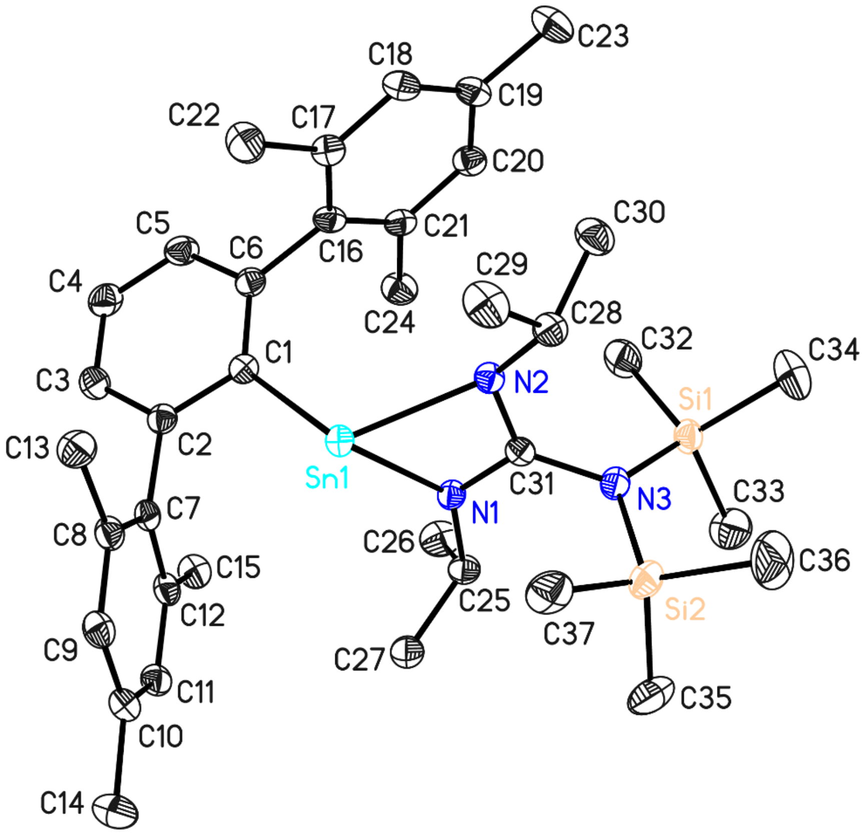

Figure S8. Molecular structure of ^{Mes}TerSn{N(^{i}Pr)C(N(SiMe$_3$)$_2$)N(^{i}Pr)} (**2a**) in the crystal. Thermal ellipsoids are drawn at the 50% probability level (hydrogen atoms have been omitted for clarity).

Table S1. Bond lengths [Å] and angles [°] for **2a**.

Sn(1)-N(1)	2.2017(17)	C(1)-C(6)	1.405(3)
Sn(1)-C(1)	2.258(2)	C(1)-C(2)	1.411(3)
Sn(1)-N(2)	2.2678(17)	C(2)-C(3)	1.395(3)
Sn(1)-C(31)	2.638(2)	C(2)-C(7)	1.503(3)
Si(1)-N(3)	1.7589(19)	C(3)-C(4)	1.384(3)
Si(1)-C(32)	1.845(3)	C(4)-C(5)	1.384(3)
Si(1)-C(33)	1.860(3)	C(5)-C(6)	1.401(3)
Si(1)-C(34)	1.867(3)	C(6)-C(16)	1.498(3)
Si(2)-N(3)	1.764(2)	C(7)-C(12)	1.406(3)
Si(2)-C(36)	1.858(3)	C(7)-C(8)	1.408(3)
Si(2)-C(37)	1.862(3)	C(8)-C(9)	1.388(3)
Si(2)-C(35)	1.873(3)	C(8)-C(13)	1.510(3)
N(1)-C(31)	1.331(3)	C(9)-C(10)	1.395(3)
N(1)-C(25)	1.460(3)	C(10)-C(11)	1.390(3)
N(2)-C(31)	1.329(3)	C(10)-C(14)	1.506(3)
N(2)-C(28)	1.471(3)	C(11)-C(12)	1.391(3)
N(3)-C(31)	1.421(3)	C(12)-C(15)	1.505(3)

C(16)-C(21)	1.402(3)	C(31)-N(3)-Si(1)	119.31(14)
C(16)-C(17)	1.403(3)	C(31)-N(3)-Si(2)	115.76(14)
C(17)-C(18)	1.391(3)	Si(1)-N(3)-Si(2)	124.73(11)
C(17)-C(22)	1.512(3)	C(6)-C(1)-C(2)	117.56(19)
C(18)-C(19)	1.390(3)	C(6)-C(1)-Sn(1)	129.72(15)
C(19)-C(20)	1.392(3)	C(2)-C(1)-Sn(1)	112.13(14)
C(19)-C(23)	1.504(3)	C(3)-C(2)-C(1)	121.1(2)
C(20)-C(21)	1.393(3)	C(3)-C(2)-C(7)	117.73(19)
C(21)-C(24)	1.507(3)	C(1)-C(2)-C(7)	121.17(18)
C(25)-C(27)	1.524(3)	C(4)-C(3)-C(2)	120.5(2)
C(25)-C(26)	1.527(3)	C(3)-C(4)-C(5)	119.4(2)
C(28)-C(29)	1.519(3)	C(4)-C(5)-C(6)	120.8(2)
C(28)-C(30)	1.522(3)	C(5)-C(6)-C(1)	120.6(2)
		C(5)-C(6)-C(16)	116.32(19)
N(1)-Sn(1)-C(1)	104.57(7)	C(1)-C(6)-C(16)	123.08(19)
N(1)-Sn(1)-N(2)	59.19(6)	C(12)-C(7)-C(8)	119.38(19)
C(1)-Sn(1)-N(2)	114.73(7)	C(12)-C(7)-C(2)	119.63(18)
N(1)-Sn(1)-C(31)	30.26(6)	C(8)-C(7)-C(2)	120.96(19)
C(1)-Sn(1)-C(31)	119.14(7)	C(9)-C(8)-C(7)	119.5(2)
N(2)-Sn(1)-C(31)	30.26(6)	C(9)-C(8)-C(13)	119.7(2)
N(3)-Si(1)-C(32)	109.02(10)	C(7)-C(8)-C(13)	120.8(2)
N(3)-Si(1)-C(33)	108.98(11)	C(8)-C(9)-C(10)	121.7(2)
C(32)-Si(1)-C(33)	110.06(13)	C(11)-C(10)-C(9)	118.1(2)
N(3)-Si(1)-C(34)	112.39(12)	C(11)-C(10)-C(14)	120.5(2)
C(32)-Si(1)-C(34)	109.23(14)	C(9)-C(10)-C(14)	121.4(2)
C(33)-Si(1)-C(34)	107.14(14)	C(10)-C(11)-C(12)	121.8(2)
N(3)-Si(2)-C(36)	109.79(13)	C(11)-C(12)-C(7)	119.34(19)
N(3)-Si(2)-C(37)	108.59(10)	C(11)-C(12)-C(15)	119.71(19)
C(36)-Si(2)-C(37)	110.89(16)	C(7)-C(12)-C(15)	120.92(19)
N(3)-Si(2)-C(35)	113.44(12)	C(21)-C(16)-C(17)	119.97(19)
C(36)-Si(2)-C(35)	106.71(16)	C(21)-C(16)-C(6)	120.62(19)
C(37)-Si(2)-C(35)	107.41(14)	C(17)-C(16)-C(6)	119.26(19)
C(31)-N(1)-C(25)	124.95(18)	C(18)-C(17)-C(16)	119.3(2)
C(31)-N(1)-Sn(1)	93.30(12)	C(18)-C(17)-C(22)	119.9(2)
C(25)-N(1)-Sn(1)	138.14(14)	C(16)-C(17)-C(22)	120.8(2)
C(31)-N(2)-C(28)	122.01(18)	C(19)-C(18)-C(17)	121.7(2)
C(31)-N(2)-Sn(1)	90.45(12)	C(18)-C(19)-C(20)	118.2(2)
C(28)-N(2)-Sn(1)	132.57(13)	C(18)-C(19)-C(23)	121.0(2)

C(20)-C(19)-C(23)	120.7(2)	N(2)-C(28)-C(30)	111.42(18)
C(19)-C(20)-C(21)	121.8(2)	C(29)-C(28)-C(30)	109.85(19)
C(20)-C(21)-C(16)	119.1(2)	N(2)-C(31)-N(1)	112.20(18)
C(20)-C(21)-C(24)	120.3(2)	N(2)-C(31)-N(3)	124.04(18)
C(16)-C(21)-C(24)	120.6(2)	N(1)-C(31)-N(3)	123.54(18)
N(1)-C(25)-C(27)	109.86(18)	N(2)-C(31)-Sn(1)	59.29(11)
N(1)-C(25)-C(26)	110.32(18)	N(1)-C(31)-Sn(1)	56.44(10)
C(27)-C(25)-C(26)	110.97(19)	N(3)-C(31)-Sn(1)	158.08(14)
N(2)-C(28)-C(29)	109.17(18)		

Reaction of ^{Dipp}TerSn{N(SiMe$_3$)$_2$} (1b) and ^{i}PrN=C=N^{i}Pr – Synthesis of ^{Dipp}TerSn{N(^{i}Pr)C(N(SiMe$_3$)$_2$)N(^{i}Pr)} (2b)

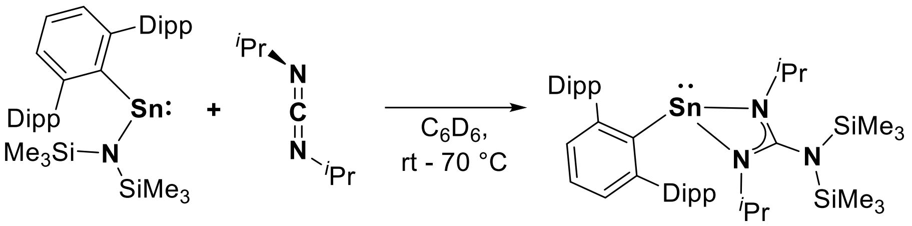

In a typical experiment, ^{Dipp}TerSn{N(SiMe$_3$)$_2$} (**1b**) (0.030 g, 0.044 mmol) was dissolved in 0.3 mL of C_6D_6 followed by addition of ^{i}PrN=C=N^{i}Pr (5.6 mg, 0.044 mmol) in 0.3 mL of C_6D_6. The reaction progress was monitored by ^{1}H NMR spectroscopy and results in clean formation of ^{Dipp}TerSn{N(^{i}Pr)C(N(SiMe$_3$)$_2$)N(^{i}Pr)} (**2b**) (Figure S9). The end of the reaction is clearly visible by a colour change from clear orange/red to clear yellow. All volatile components were removed under vacuum to give **2b** as a colourless solid.

Yield: 0.031 g (0.039 mmol; 89%).
^{1}H NMR (400 MHz, C_6D_6, 298 K): δ = 0.08 (s, 9H, Si(CH$_3$)$_3$), 0.18 (s, 9H, Si(CH$_3$)$_3$), 0.71-0.75 (m, 12H, NCH(C*H*$_3$)$_2$), 1.13 (d, $^{3}J_{H,H}$ = 6.8 Hz, 12H, CH(C*H*$_3$)$_2$), 1.46 (d, $^{3}J_{H,H}$ = 7.0 Hz, 12H, CH(C*H*$_3$)$_2$), 3.25 (hept, $^{3}J_{H,H}$ = 6.6 Hz, 4H, C*H*(CH$_3$)$_2$), 3.76 (hept, $^{3}J_{H,H}$ = 6.4 Hz, 2H, NC*H*(CH$_3$)$_2$), 6.98-7.00 (m, 2H, CH$_{Aryl}$), 7.09-7.13 (m, 1H, CH$_{Aryl}$), 7.23-7.32 (m, 6H, CH$_{Aryl}$) ppm.
^{1}H NMR (400 MHz, C_7D_8, 298 K): δ = 0.06 (s, 9H, Si(CH$_3$)$_3$), 0.16 (s, 9H, Si(CH$_3$)$_3$), 0.65 (d, $^{3}J_{H,H}$ = 6.6 Hz, 6H, NCH(C*H*$_3$)$_2$), 0.69 (d, $^{3}J_{H,H}$ = 6.5 Hz, 6H, NCH(C*H*$_3$)$_2$), 1.10 (d, $^{3}J_{H,H}$ = 6.8 Hz, 12H, CH(C*H*$_3$)$_2$), 1.41 (d, $^{3}J_{H,H}$ = 6.9 Hz, 12H, CH(C*H*$_3$)$_2$), 3.18 (hept, $^{3}J_{H,H}$ = 6.9 Hz, 4H, C*H*(CH$_3$)$_2$), 3.71 (hept, $^{3}J_{H,H}$ = 6.6 Hz, 2H, NC*H*(CH$_3$)$_2$), 6.92-6.94 (m, 2H, CH$_{Aryl}$), 7.08-7.11 (m, 1H, CH$_{Aryl}$)*, 7.19-7.21 (m, 4H, CH$_{Aryl}$), 7.23-7.26 (m, 2H, CH$_{Aryl}$) ppm.
* = overlap with C_7D_7H signal
^{13}C{^{1}H} NMR (101 MHz, C_7D_8, 298 K): δ = 2.4 (Si(CH$_3$)$_3$), 2.5 (Si(CH$_3$)$_3$), 24.5 (CH($\underline{C}$H$_3$)$_2$), 25.27 (CH($\underline{C}$H$_3$)$_2$), 25.33 (NCH($\underline{C}$H$_3$)$_2$), 27.1 (NCH($\underline{C}$H$_3$)$_2$), 31.5 ($\underline{C}$H(CH$_3$)$_2$), 46.2 (N$\underline{C}$H(CH$_3$)$_2$), 124.3 (CH$_{Aryl}$), 125.6 (CH$_{Aryl}$), 128.3 (CH$_{Aryl}$), 130.8 (CH$_{Aryl}$), 143.7 (C$_{q,Aryl}$), 146.2 (C$_{q,Aryl}$), 147.3 (C$_{q,Aryl}$), 161.4 (C$_q$(N^{i}Pr)$_2$N(SiMe$_3$)$_2$), 178.1 (C$_q$Sn) ppm.
^{29}Si{^{1}H} NMR (80 MHz, C_7D_8, 298 K): δ = 4.7, 7.8 ppm. (assigned by ^{1}H/^{29}Si HMBC)
^{119}Sn{^{1}H} NMR (149 MHz, C_7D_8, 298 K): δ = 95.2 ppm.
EA: Anal. calcd. for $C_{43}H_{69}N_3Si_2Sn$: C, 64.32; H, 8.66; N, 5.23; Found: C, 63.71; H, 8.39; N, 5.00.

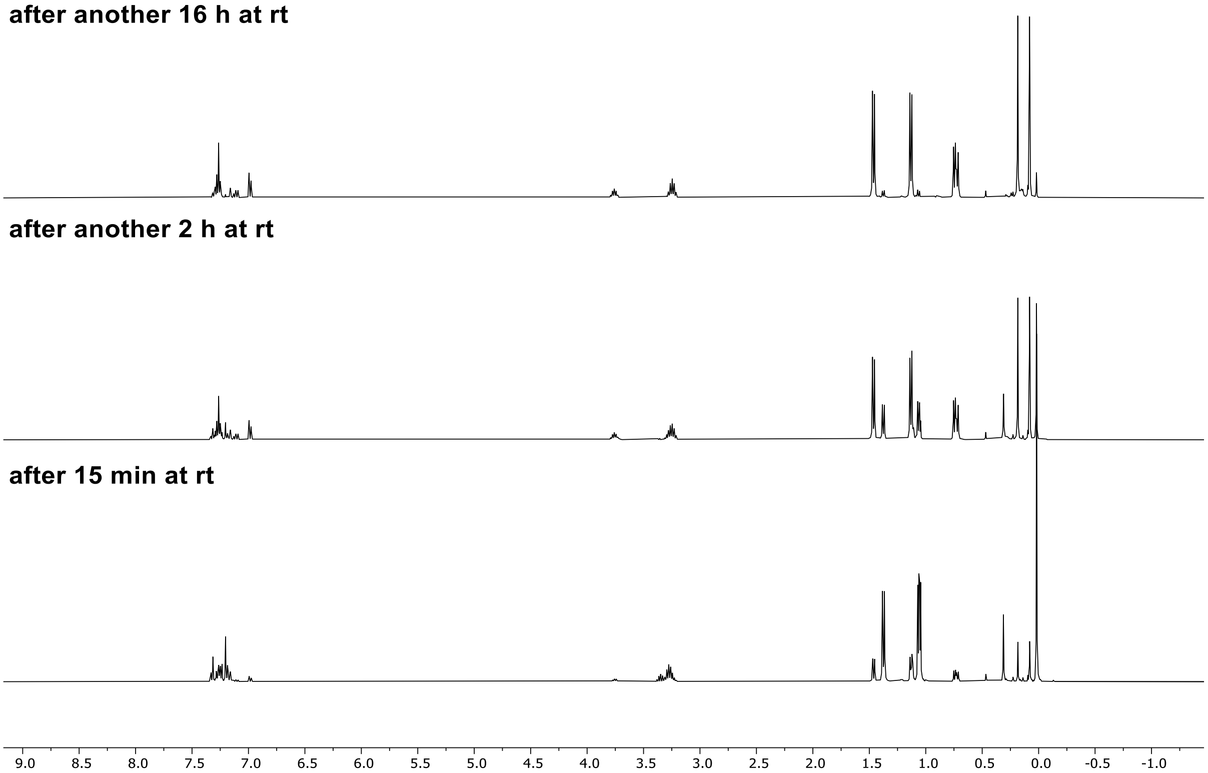

Figure S9. Monitoring of the reaction of ^{Dipp}TerSn{N(SiMe$_3$)$_2$} (**1b**) and ^{i}PrN=C=N^{i}Pr *via* ^{1}H NMR spectroscopy (400 MHz, C_6D_6, 298 K).

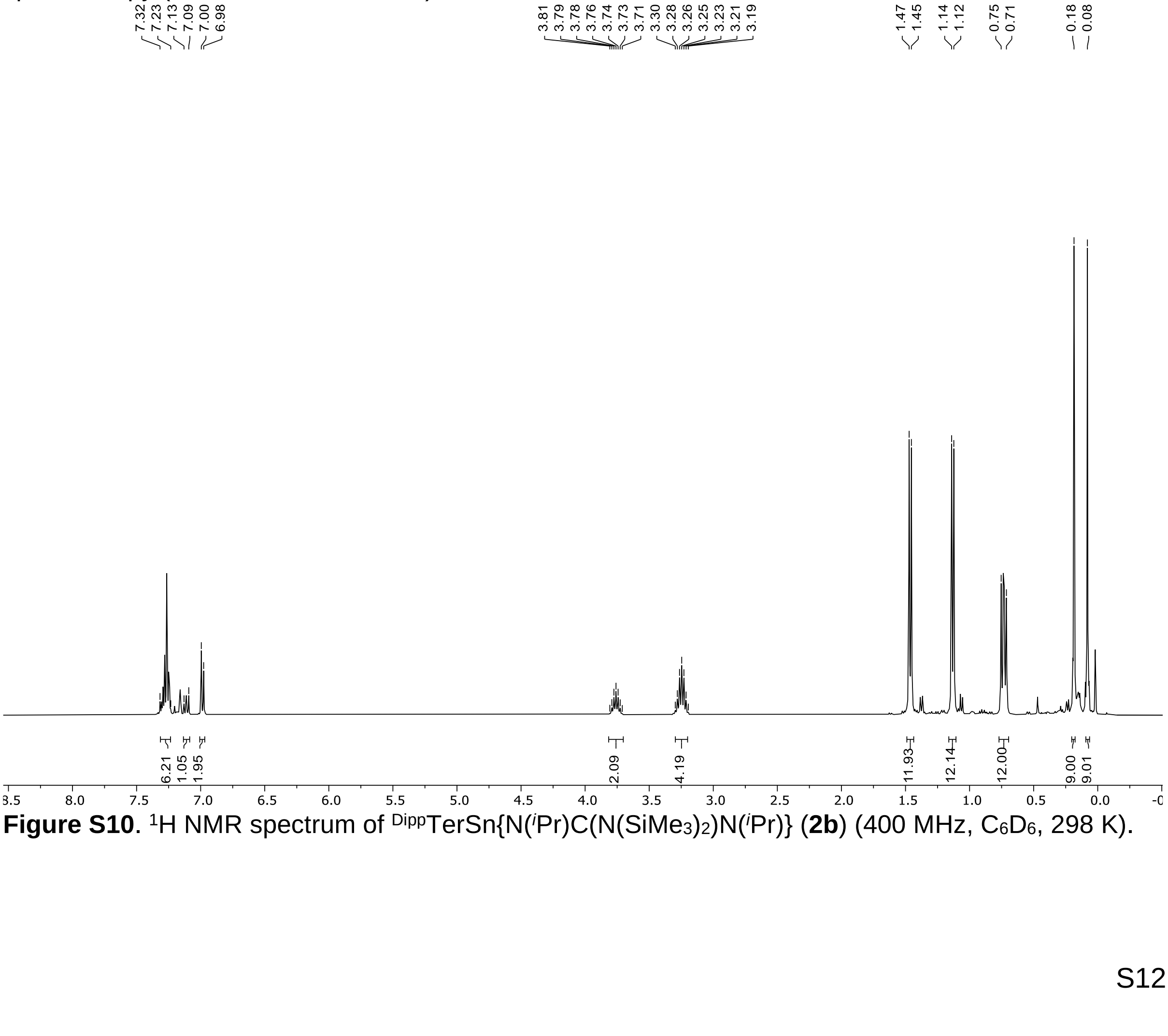

Figure S10. ^{1}H NMR spectrum of ^{Dipp}TerSn{N(^{i}Pr)C(N(SiMe$_3$)$_2$)N(^{i}Pr)} (**2b**) (400 MHz, C_6D_6, 298 K).

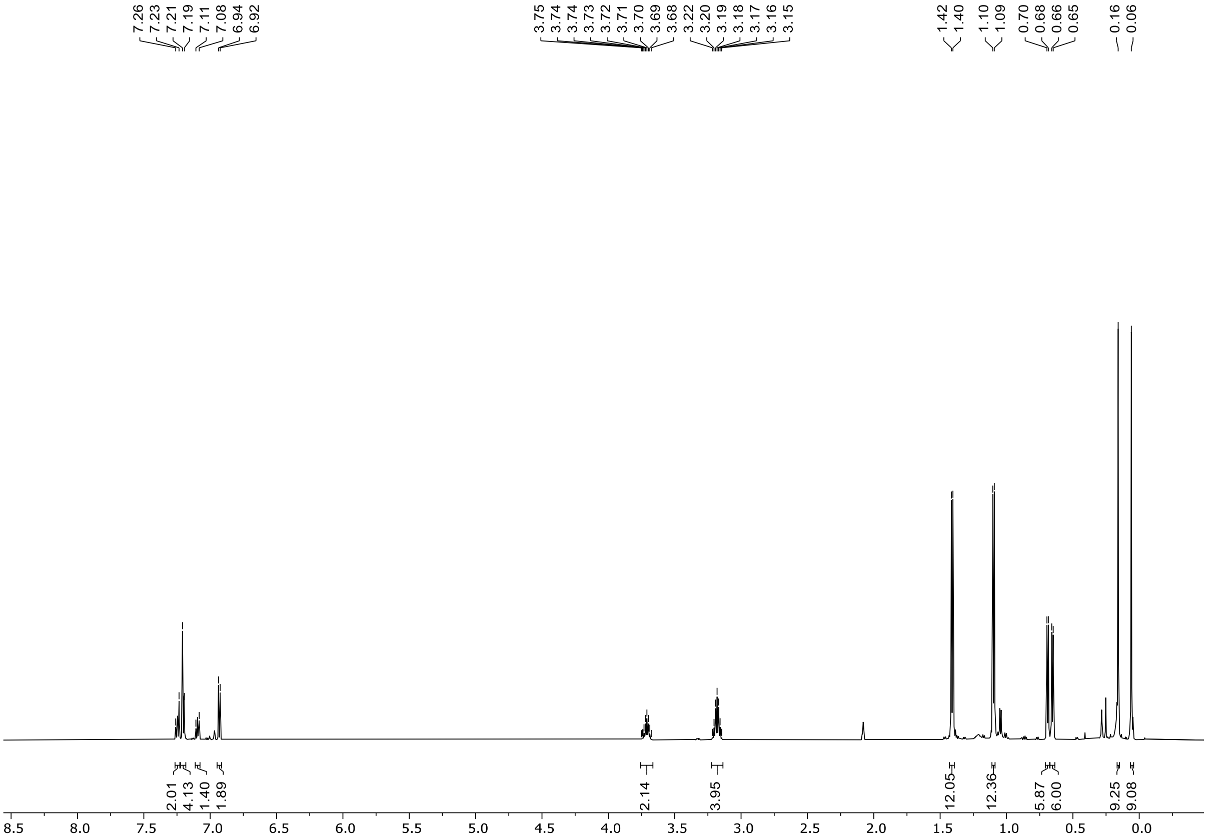

Figure S11. ^{1}H NMR spectrum of ^{Dipp}TerSn{N(^{i}Pr)C(N(SiMe$_3$)$_2$)N(^{i}Pr)} (**2b**) (400 MHz, C_7D_8, 298 K).

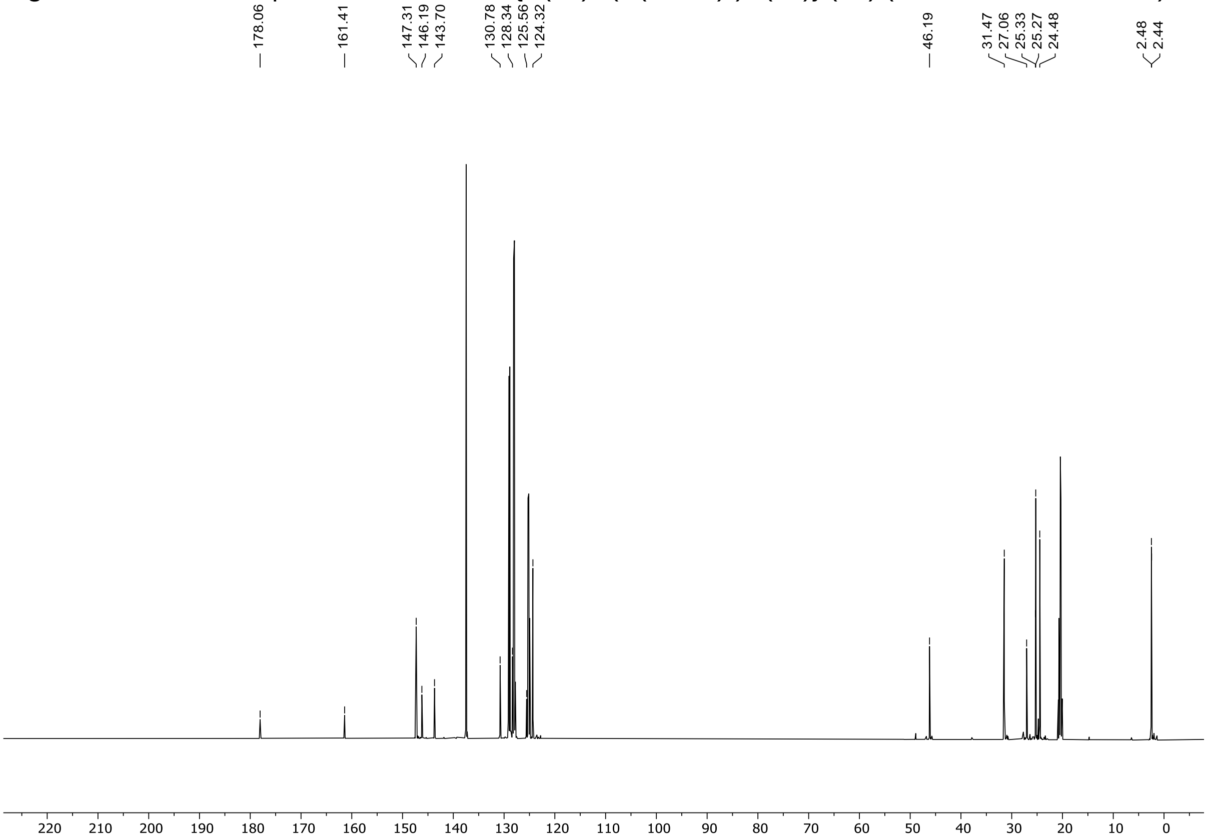

Figure S12. ^{13}C{^{1}H} NMR spectrum of ^{Dipp}TerSn{N(^{i}Pr)C(N(SiMe$_3$)$_2$)N(^{i}Pr)} (**2b**) (126 MHz, C_7D_8, 298 K).

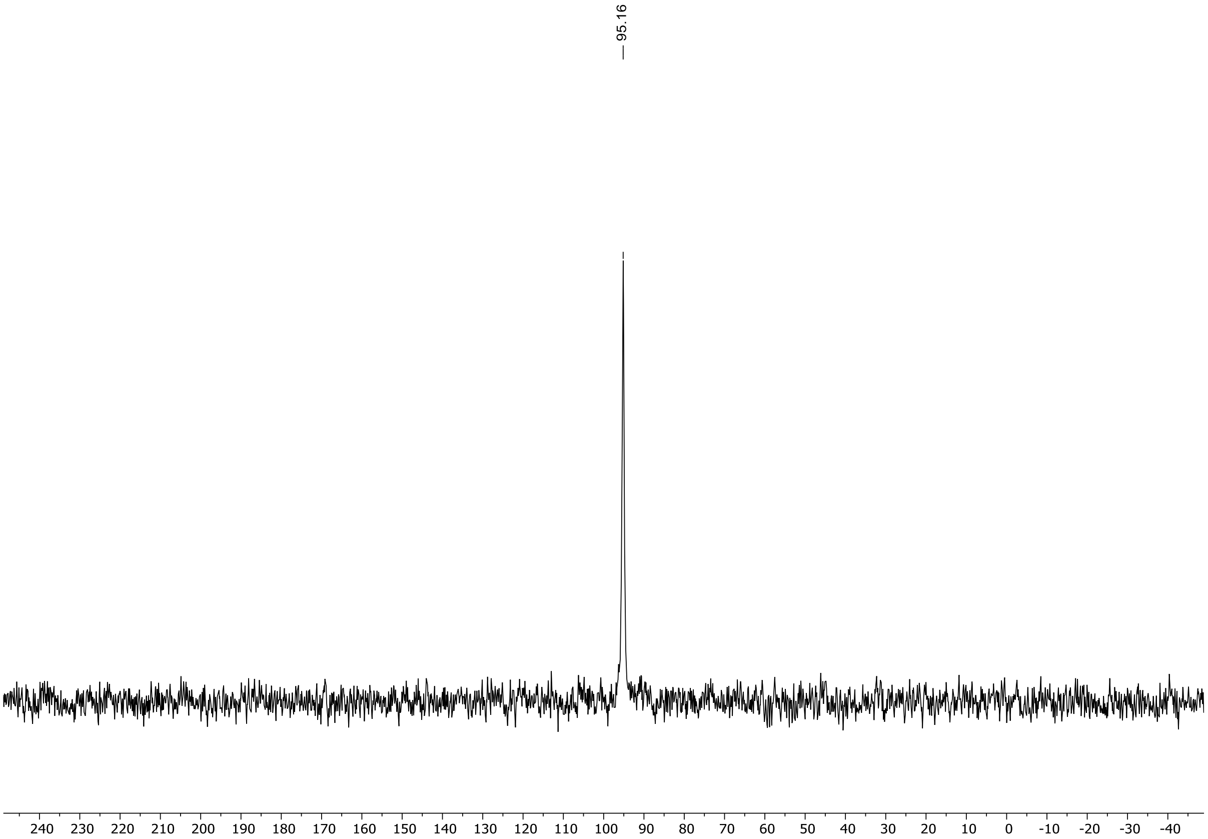

Figure S13. $^{119}Sn\{^1H\}$ NMR spectrum of ^{Dipp}TerSn{N(^{i}Pr)C(N(SiMe$_3$)$_2$)N(^{i}Pr)} (**2b**) (149 MHz, C_7D_8, 298 K).

Reaction of ^{Mes}TerSn{N(^{i}Pr)C(N(SiMe$_3$)$_2$)N(^{i}Pr)} (2a) with Se – Formation of ^{Mes}TerSn(Se){N(^{i}Pr)C(N(SiMe$_3$)$_2$)N(^{i}Pr)} (3a)

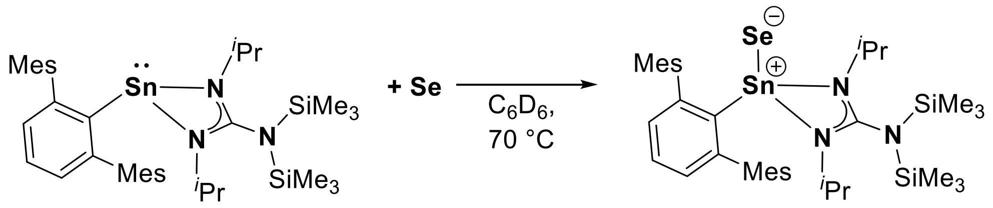

^{Mes}TerSn{N(SiMe$_3$)$_2$} (**1a**) (0.030 g, 0.051 mmol) was dissolved in 0.3 mL of C_6D_6 followed by addition of ^{i}PrN=C=N^{i}Pr (6.4 mg, 0.051 mmol) in 0.3 mL of C_6D_6. The reaction progress was monitored by ^{1}H NMR spectroscopy until both starting materials have been consumed and ^{Mes}TerSn{N(^{i}Pr)C(N(SiMe$_3$)$_2$)N(^{i}Pr)} (**2a**) has formed. Elemental Se (0.010 g, 0.13 mmol) was added and the reaction mixture was heated to 70 °C for one hour leading to a colour change to a more intense yellow and clean formation of ^{Mes}TerSn(Se){N(^{i}Pr)C(N(SiMe$_3$)$_2$)N(^{i}Pr)} (**3a**) was verified by ^{1}H NMR spectroscopy. The supernatant was filtered and all volatile components were removed under vacuum to give **3a** as a yellow solid.

Yield: 0.035 g (0.044 mmol; 86%).
^{1}H NMR (400 MHz, C_6D_6, 298 K): δ = 0.18 (s, 9H, Si(CH$_3$)$_3$), 0.23 (s, 9H, Si(CH$_3$)$_3$), 0.76 (d, $^3J_{H,H}$ = 6.7 Hz, 6H, CH(C*H*$_3$)$_2$), 0.85 (d, $^3J_{H,H}$ = 6.5 Hz, 6H, CH(C*H*$_3$)$_2$), 2.19 (s, 6H, CH$_3$), 2.43 (s, 12H, CH$_3$), 3.72 (hept, $^3J_{H,H}$ = 6.6 Hz, 2H, C*H*(CH$_3$)$_2$), 6,75-6.77 (m, 2H, CH$_{Aryl}$), 6.85 (s, 4H, CH$_{Aryl}$), 7.09-7.11 (m, 1H, CH$_{Aryl}$) ppm.
^{13}C{^{1}H} NMR (101 MHz, C_6D_6, 298 K): δ = 2.4 (Si(CH$_3$)$_3$), 3.0 (Si(CH$_3$)$_3$), 22.1 (CH$_3$), 23.0 (CH$_3$), 23.8 (CH(<u>C</u>H$_3$)$_2$), 24.0 (CH(<u>C</u>H$_3$)$_2$), 47.8 (<u>C</u>H(CH$_3$)$_2$), 129.8 (CH$_{Aryl}$), 130.4 (CH$_{Aryl}$), 130.5 (CH$_{Aryl}$), 137.8 (C$_{q,Aryl}$), 138.0 (C$_{q,Aryl}$), 140.7 (C$_{q,Aryl}$), 147.8 (C$_{q,Aryl}$), 149.5 (C$_{q,Aryl}$), 169.5 (C$_q$(N^{i}Pr)$_2$N(SiMe$_3$)$_2$) ppm.
^{29}Si{^{1}H} NMR (80 MHz, C_6D_6, 298 K): δ = 4.7, 10.1 ppm. (assigned by ^{1}H/^{29}Si HMBC)
^{77}Se{^{1}H} NMR (115 MHz, C_6D_6, 298 K): δ = -134.6 ppm.
^{119}Sn{^{1}H} NMR (149 MHz, C_6D_6, 298 K): δ = -165.3 ppm.
MS (LIFDI): m/z calcd. for $C_{37}H_{57}N_3SeSi_2Sn$: 799.2278; found: 799.1.
EA: Anal. calcd. for $C_{37}H_{57}N_3SeSi_2Sn$: C, 55.71; H, 7.20; N, 5.27; Found: C, 55.69; H, 7.27; N, 5.28.

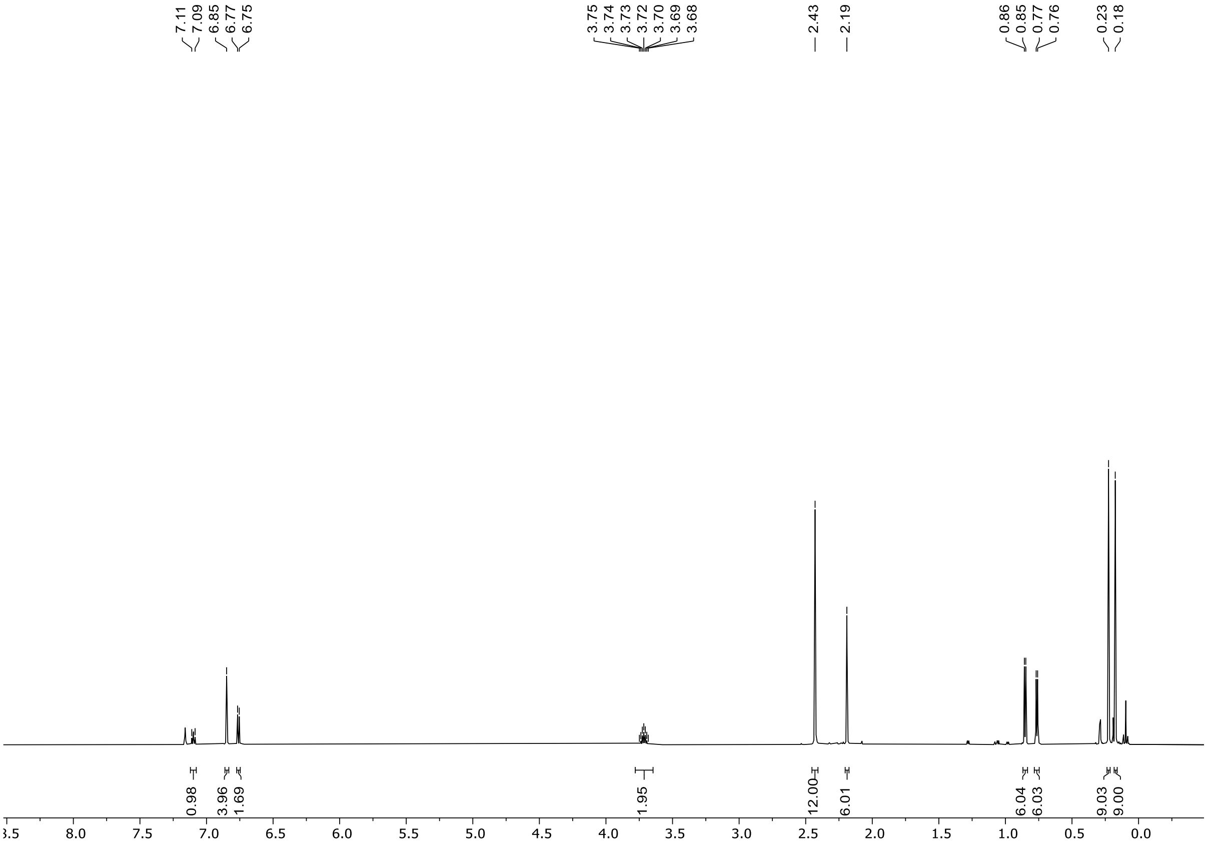

Figure S14. ^{1}H NMR spectrum of ^{Mes}TerSn(Se){N(^{i}Pr)C(N(SiMe$_3$)$_2$)N(^{i}Pr)} (**3a**) (400 MHz, C_6D_6, 298 K).

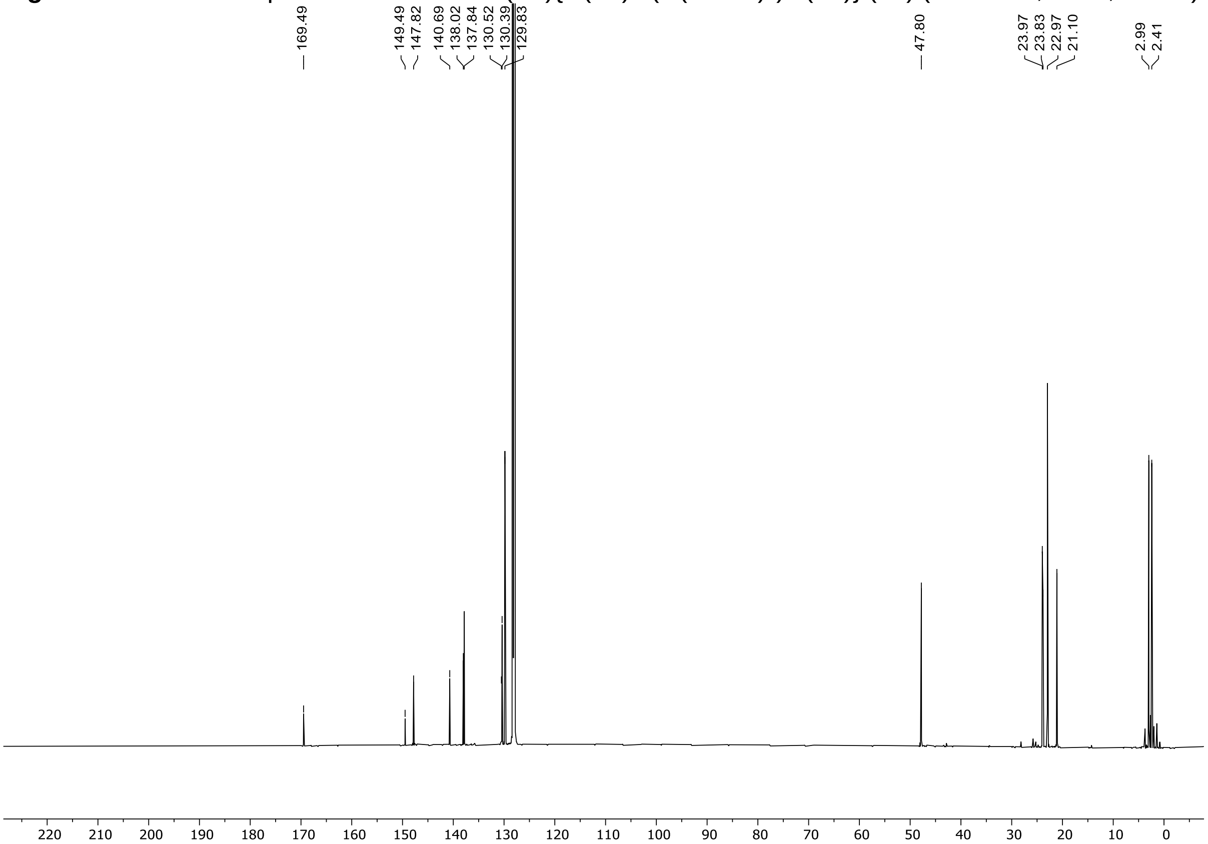

Figure S15. ^{13}C{^{1}H} NMR spectrum of ^{Mes}TerSn(Se){N(^{i}Pr)C(N(SiMe$_3$)$_2$)N(^{i}Pr)} (**3a**) (126 MHz, C_6D_6, 298 K).

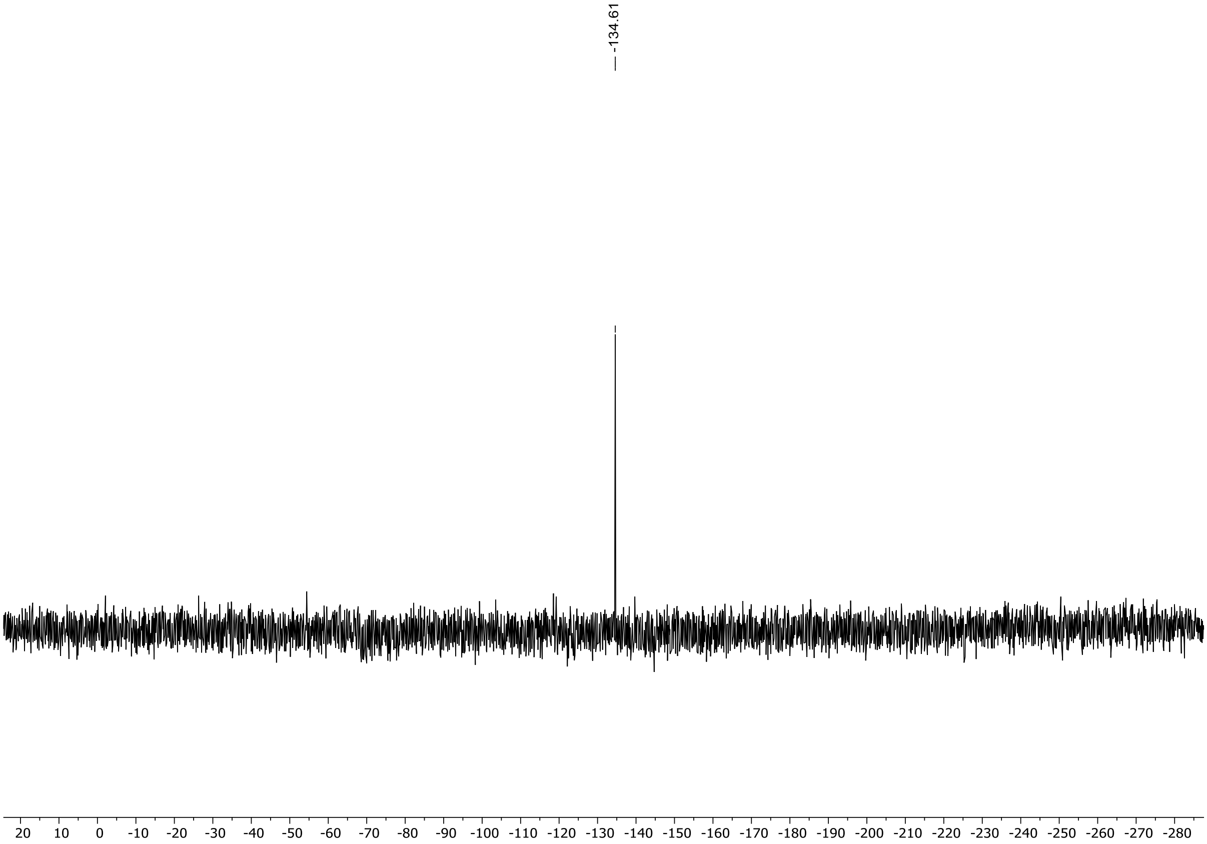

Figure S16. $^{77}Se\{^{1}H\}$ NMR spectrum of ^{Mes}TerSn(Se){N(^{i}Pr)C(N(SiMe$_3$)$_2$)N(^{i}Pr)} (**3a**) (115 MHz, C_6D_6, 298 K).

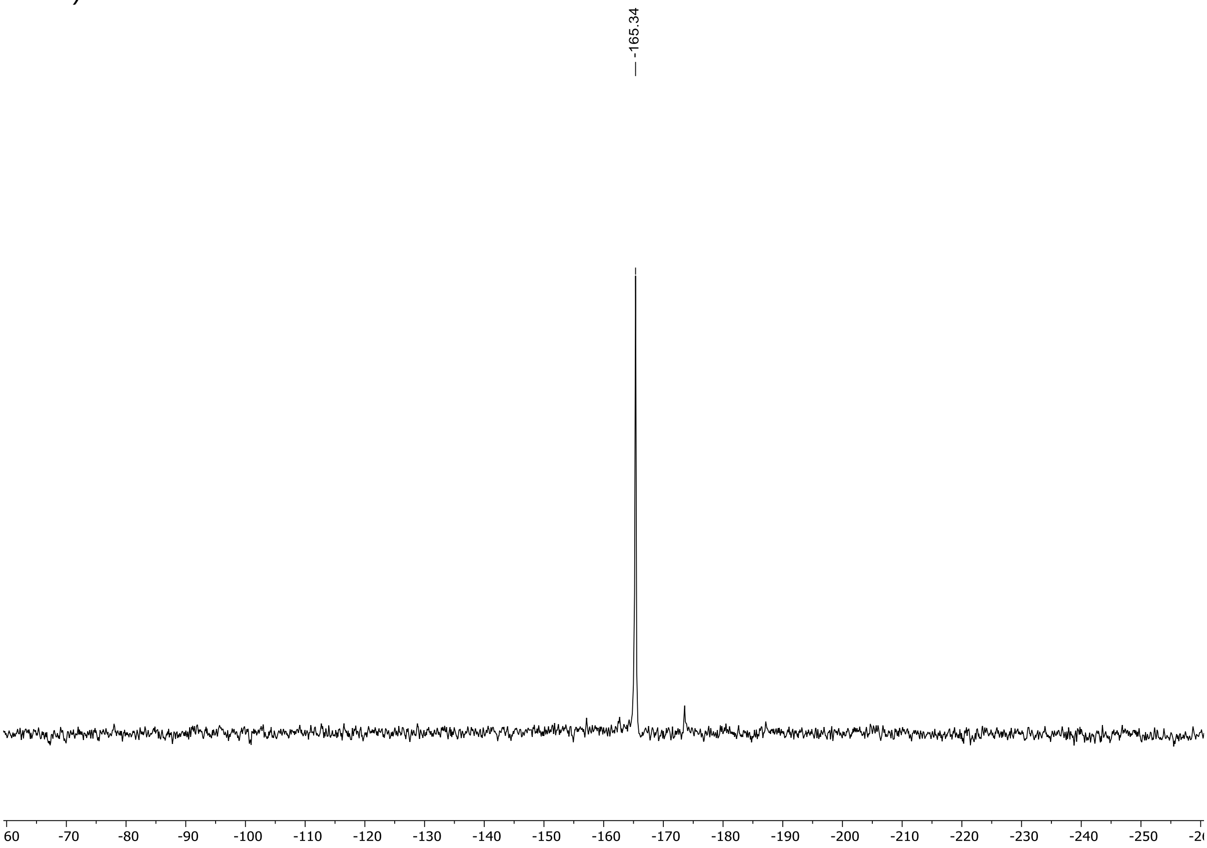

Figure S17. $^{119}Sn\{^{1}H\}$ NMR spectrum of ^{Mes}TerSn(Se){N(^{i}Pr)C(N(SiMe$_3$)$_2$)N(^{i}Pr)} (**3a**) (149 MHz, C_6D_6, 298 K).

Reaction of ^{Dipp}TerSn{N(^{i}Pr)C(N(SiMe$_3$)$_2$)N(^{i}Pr)} (2b) with Se – Formation of ^{Dipp}TerSn(Se){N(^{i}Pr)C(N(SiMe$_3$)$_2$)N(^{i}Pr)} (3b)

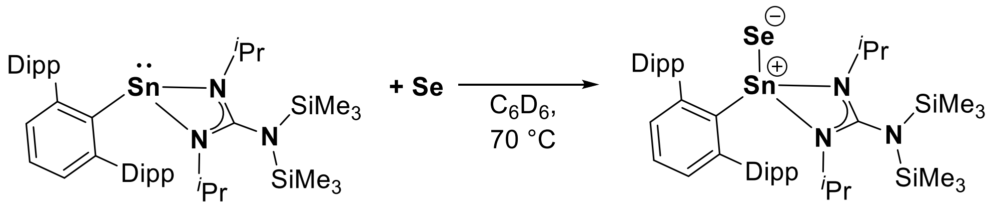

^{Dipp}TerSn{N(SiMe$_3$)$_2$} (**1b**) (0.030 g, 0.037 mmol) was dissolved in 0.3 mL of C_6D_6 followed by addition of ^{i}PrN=C=N^{i}Pr (4.7 mg, 0.037 mmol) in 0.3 mL of C_6D_6. The reaction progress was monitored by ^{1}H NMR spectroscopy until both starting materials have been consumed and ^{Dipp}TerSn{N(^{i}Pr)C(N(SiMe$_3$)$_2$)N(^{i}Pr)} (**2b**) has formed. Elemental Se (0.010 g, 0.13 mmol) was added and the reaction mixture was heated to 70 °C for one hour leading to a colour change to a more intense yellow and clean formation of ^{Dipp}TerSn(Se){N(^{i}Pr)C(N(SiMe$_3$)$_2$)N(^{i}Pr)} (**3b**) was verified by ^{1}H NMR spectroscopy. All volatile components were removed under vacuum, 0.6 mL of *n*-hexane were added, the suspension was filtered and stored at -30 °C to give **3b** a yellow crystalline material. Crystals obtained this way were suitable for single crystal X-ray diffraction.

Yield: 0.018 g (0.020 mmol; 54%).
^{1}H NMR (400 MHz, C_6D_6, 298 K): δ = 0.11 (s, 9H, Si(CH$_3$)$_3$), 0.23 (s, 9H, Si(CH$_3$)$_3$), 0.57 (d, $^{3}J_{H,H}$ = 6.5 Hz, 6H, NCH(C*H*$_3$)$_2$), 1.00 (d, $^{3}J_{H,H}$ = 6.6 Hz, 6H, NCH(C*H*$_3$)$_2$), 1.04 (d, $^{3}J_{H,H}$ = 6.8 Hz, 6H, CH(C*H*$_3$)$_2$), 1.31-1.75 (m(br), 12H, CH(C*H*$_3$)$_2$), 2.64-3.16 (m(br), 2H, C*H*(CH$_3$)$_2$), 3.45-3.83 (m, 2H, CH(C*H*$_3$)$_2$), 3.68 (hept, $^{3}J_{H,H}$ = 6.6 Hz, 2H, NC*H*(CH$_3$)$_2$), 7.02-7.06 (m, 3H, CH$_{Aryl}$), 7.20-7.30 (m, 6H, CH$_{Aryl}$) ppm.
^{1}H NMR (400 MHz, C_7D_8, 243 K): δ = 0.09 (s, 9H, Si(CH$_3$)$_3$), 0.21 (s, 9H, Si(CH$_3$)$_3$), 0.56 (d, $^{3}J_{H,H}$ = 6.5 Hz, 6H, NCH(C*H*$_3$)$_2$), 0.97 (d, $^{3}J_{H,H}$ = 6.6 Hz, 6H, NCH(C*H*$_3$)$_2$), 1.03-1.05 (m, 12H, CH(C*H*$_3$)$_2$), 1.28 (d, $^{3}J_{H,H}$ = 7.0 Hz, 6H, CH(C*H*$_3$)$_2$), 1.77 (d, $^{3}J_{H,H}$ = 6.5 Hz, 6H, CH(C*H*$_3$)$_2$), 2.73 (hept, $^{3}J_{H,H}$ = 6.9 Hz, 2H, C*H*(CH$_3$)$_2$), 3.67 (hept, $^{3}J_{H,H}$ = 6.9 Hz, 2H, NC*H*(CH$_3$)$_2$), 3.74 (hept, $^{3}J_{H,H}$ = 6.9 Hz, 2H, C*H*(CH$_3$)$_2$), 6.98-7.00 (m, 2H, CH$_{Aryl}$), 7.03-7.06 (m, 2H, CH$_{Aryl}$), 7.12-7.13 (m, 1H, CH$_{Aryl}$), 7.23-7.28 (m, 4H, CH$_{Aryl}$) ppm.
^{13}C{^{1}H} NMR (101 MHz, C_6D_6, 298 K): δ = 2.5 (Si(CH$_3$)$_3$), 3.2 (Si(CH$_3$)$_3$), 25.2 (NCH($\underline{C}$H$_3$)$_2$), 25.3 (NCH($\underline{C}$H$_3$)$_2$), 47.8 (N$\underline{C}$H(CH$_3$)$_2$), 129.7 (CH$_{Aryl}$), 132.2 (CH$_{Aryl}$), 141.7 (C$_{q,Aryl}$), 145.1 (C$_{q,Aryl}$), 148.5 (C$_{q,Aryl}$), 153.7 (C$_{q,Aryl}$), 168.9 (C$_q$(N^{i}Pr)$_2$N(SiMe$_3$)$_2$) ppm.
Note: the corresponding signals of the broad signals in the ^{1}H NMR can not be observed at room temperature.
^{13}C{^{1}H} NMR (101 MHz, C_7D_8, 243 K): δ = 2.2 (Si(CH$_3$)$_3$), 2.8 (Si(CH$_3$)$_3$), 23.8 (CH($\underline{C}$H$_3$)$_2$), 24.5 (CH($\underline{C}$H$_3$)$_2$), 25.05 (CH($\underline{C}$H$_3$)$_2$), 25.1 (N$\underline{C}$H(CH$_3$)$_2$), 25.2 (N$\underline{C}$H(CH$_3$)$_2$), 26.8 (CH($\underline{C}$H$_3$)$_2$), 30.6 ($\underline{C}$H(CH$_3$)$_2$), 31.6 ($\underline{C}$H(CH$_3$)$_2$), 47.6 (N$\underline{C}$H(CH$_3$)$_2$), 123.9 (CH$_{Aryl}$), 124.7 (CH$_{Aryl}$), 129.6 (CH$_{Aryl}$), 131.7 (CH$_{Aryl}$), 141.4 (C$_{q,Aryl}$), 145.0 (C$_{q,Aryl}$), 147.9 (C$_{q,Aryl}$), 148.3 (C$_{q,Aryl}$), 153.0 (C$_{q,Aryl}$), 168.7 (C$_q$(N^{i}Pr)$_2$N(SiMe$_3$)$_2$) ppm.
^{29}Si{^{1}H} NMR (80 MHz, C_6D_6, 298 K): δ = 5.5, 10.9 ppm. (assigned by ^{1}H/^{29}Si HMBC)
^{77}Se{^{1}H} NMR (115 MHz, C_6D_6, 298 K): δ = -100.6 ppm.
^{119}Sn{^{1}H} NMR (149 MHz, C_6D_6, 298 K): δ = -173.1 ppm.
EA: Anal. calcd. for $C_{43}H_{69}N_3SeSi_2Sn\bullet C_6H_{14}$: C, 60.79; H, 8.64; N, 4.34; Found: C, 61.21; H, 7.86; N, 4.67.

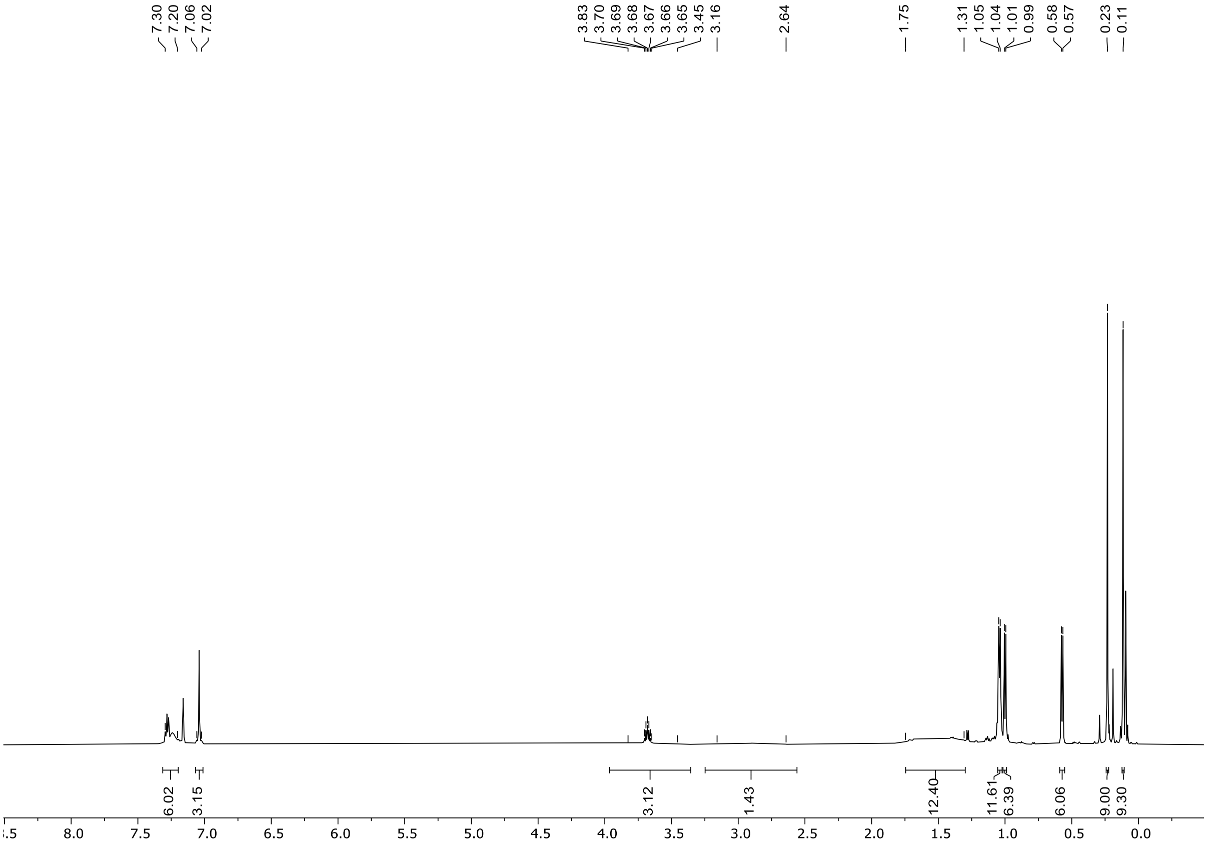

Figure S18. ^{1}H NMR spectrum of ^{Dipp}TerSn(Se){N(^{i}Pr)C(N(SiMe$_3$)$_2$)N(^{i}Pr)} (**3b**) (400 MHz, C_6D_6, 298 K).

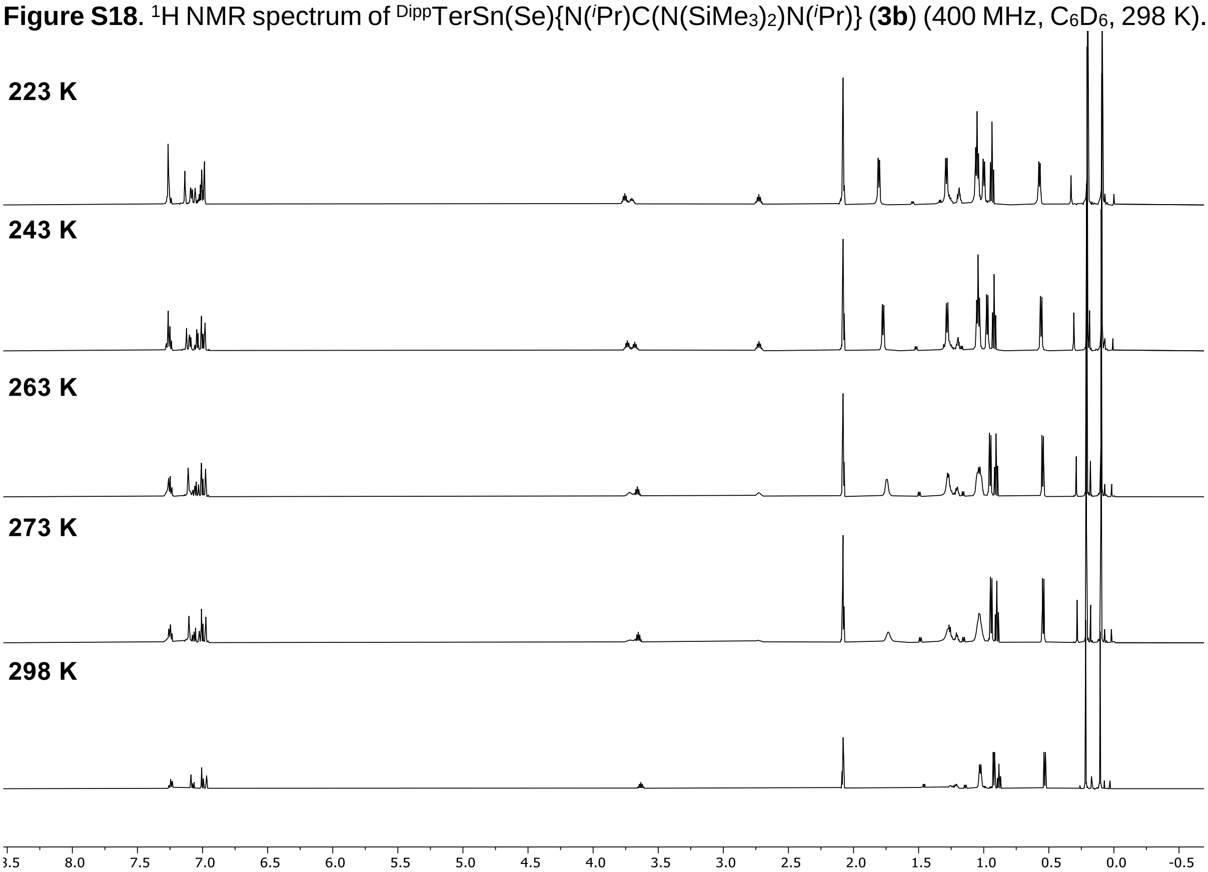

Figure S19. VT ^{1}H NMR experiment of ^{Dipp}TerSn(Se){N(^{i}Pr)C(N(SiMe$_3$)$_2$)N(^{i}Pr)} (**3b**) (400 MHz, C_7D_8, 223 K – 298 K).

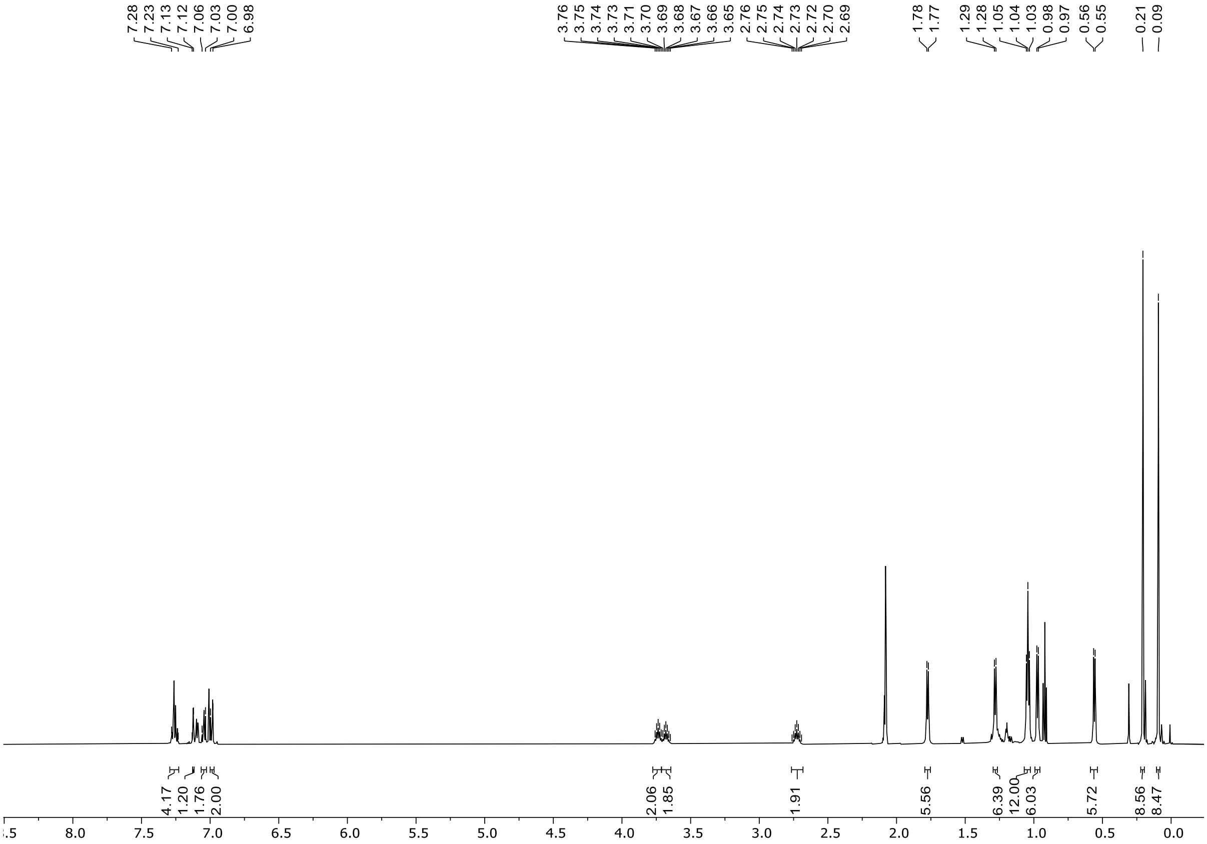

Figure S20. ^{1}H NMR spectrum of ^{Dipp}TerSn(Se){N(^{i}Pr)C(N(SiMe$_3$)$_2$)N(^{i}Pr)} (**3b**) (400 MHz, C_7D_8, 243 K).

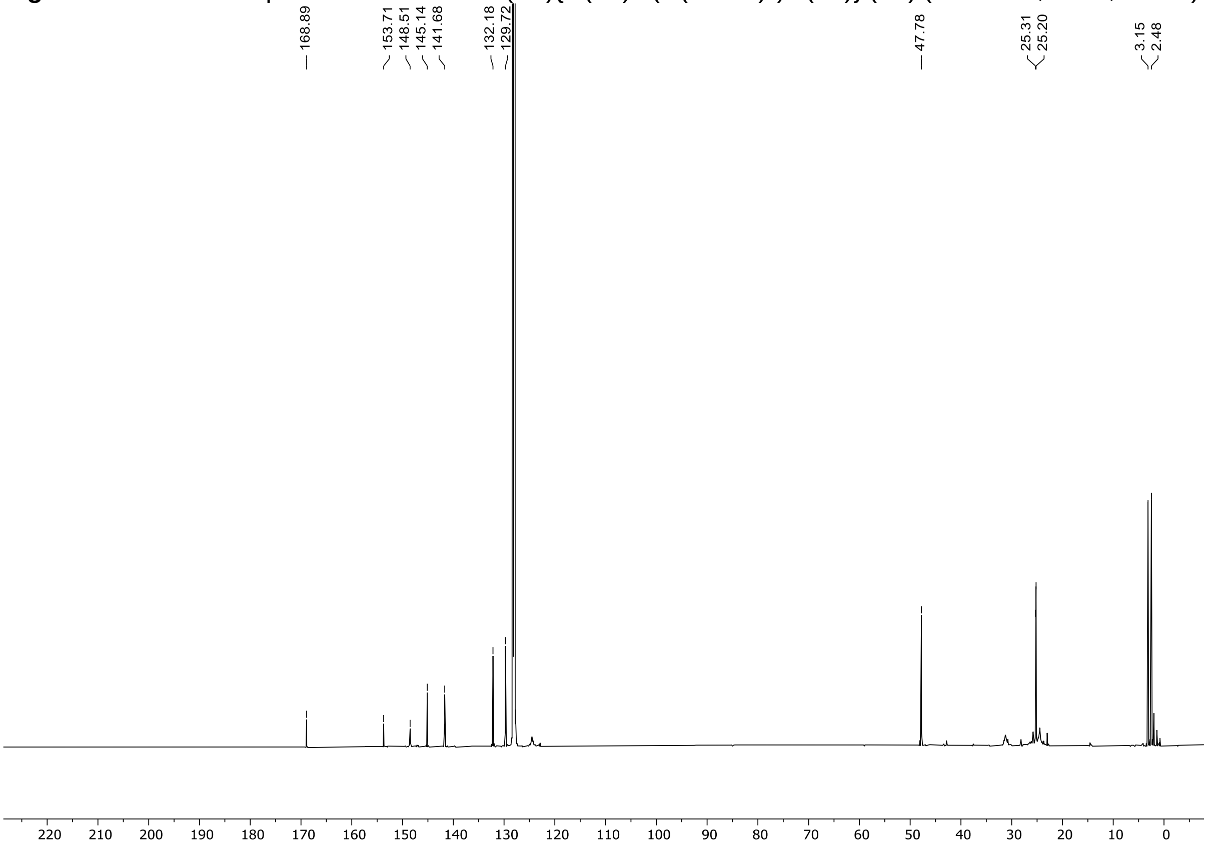

Figure S21. ^{13}C{^{1}H} NMR spectrum of ^{Dipp}TerSn(Se){N(^{i}Pr)C(N(SiMe$_3$)$_2$)N(^{i}Pr)} (**3b**) (126 MHz, C_6D_6, 298 K).

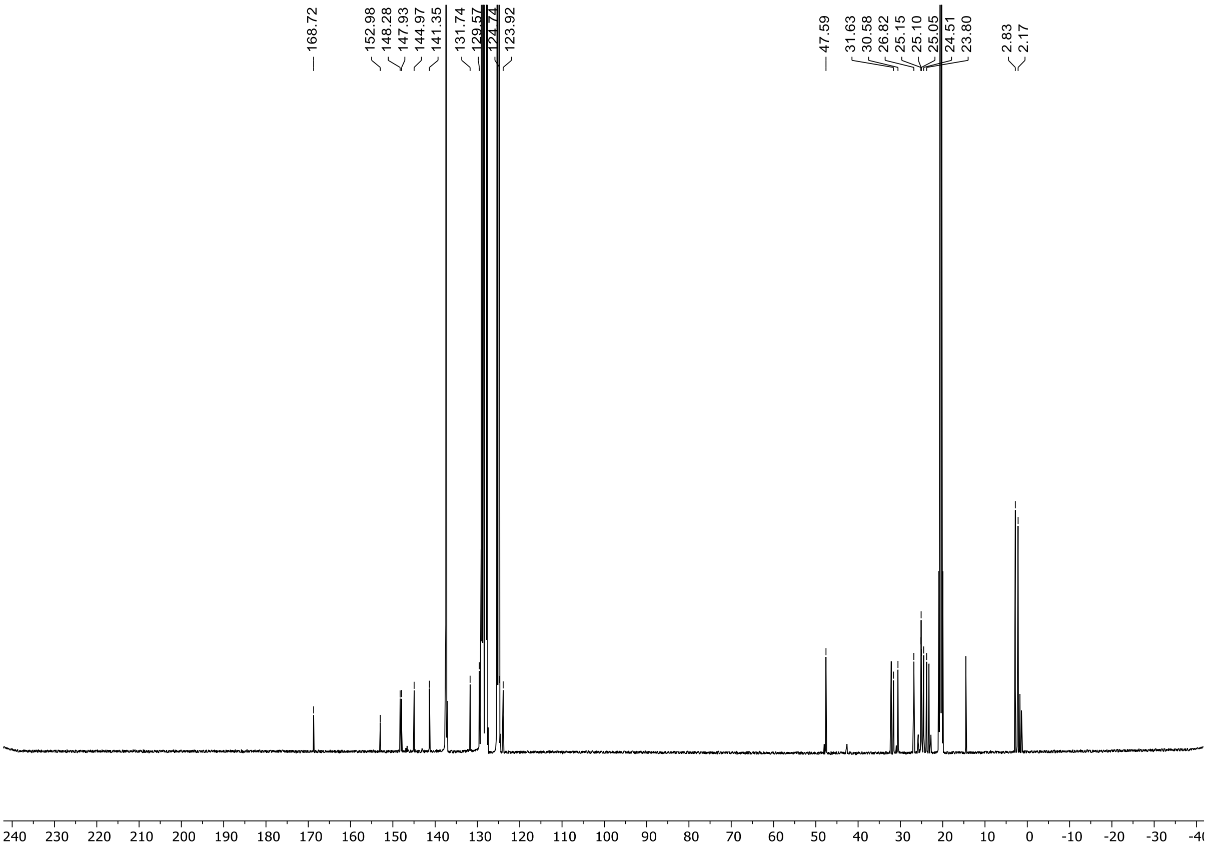

Figure S22. $^{13}C\{^{1}H\}$ NMR spectrum of ^{Dipp}TerSn(Se){N(^{i}Pr)C(N(SiMe$_3$)$_2$)N(^{i}Pr)} (**3b**) (126 MHz, C_7D_8, 243 K).

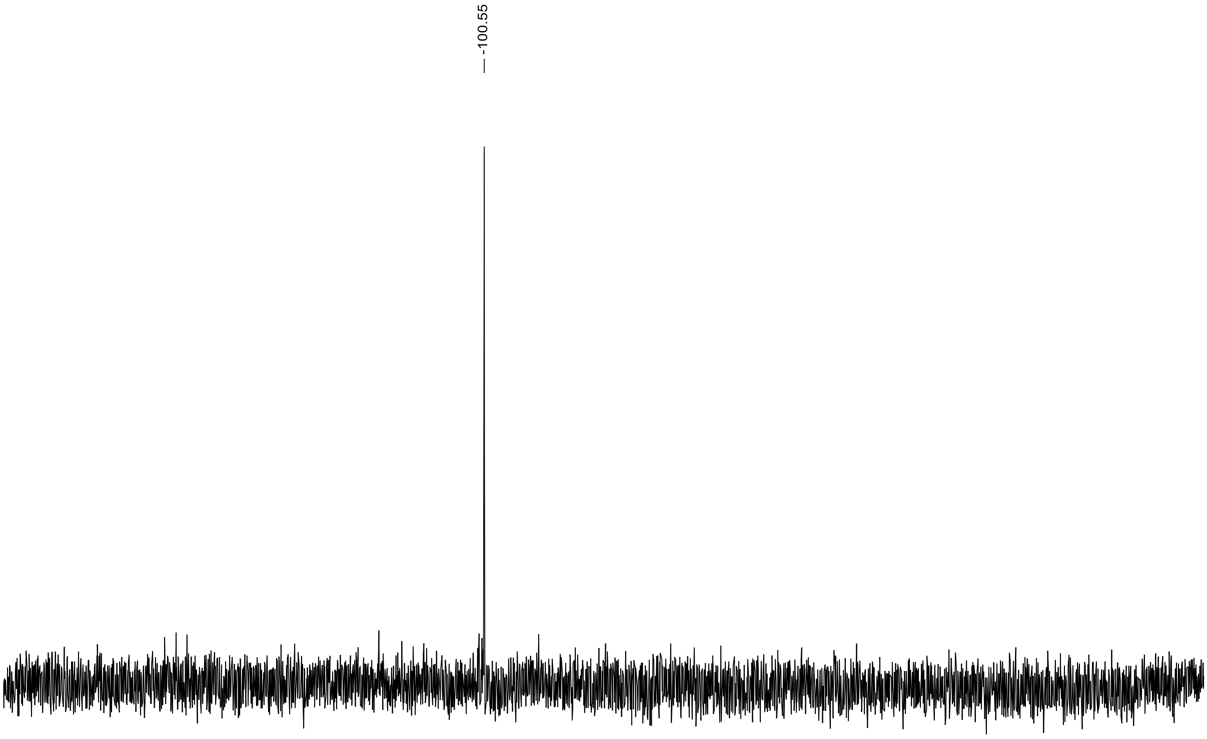

Figure S23. $^{77}Se\{^{1}H\}$ NMR spectrum of ^{Dipp}TerSn(Se){N(^{i}Pr)C(N(SiMe$_3$)$_2$)N(^{i}Pr)} (**3b**) (115 MHz, C_6D_6, 298 K).

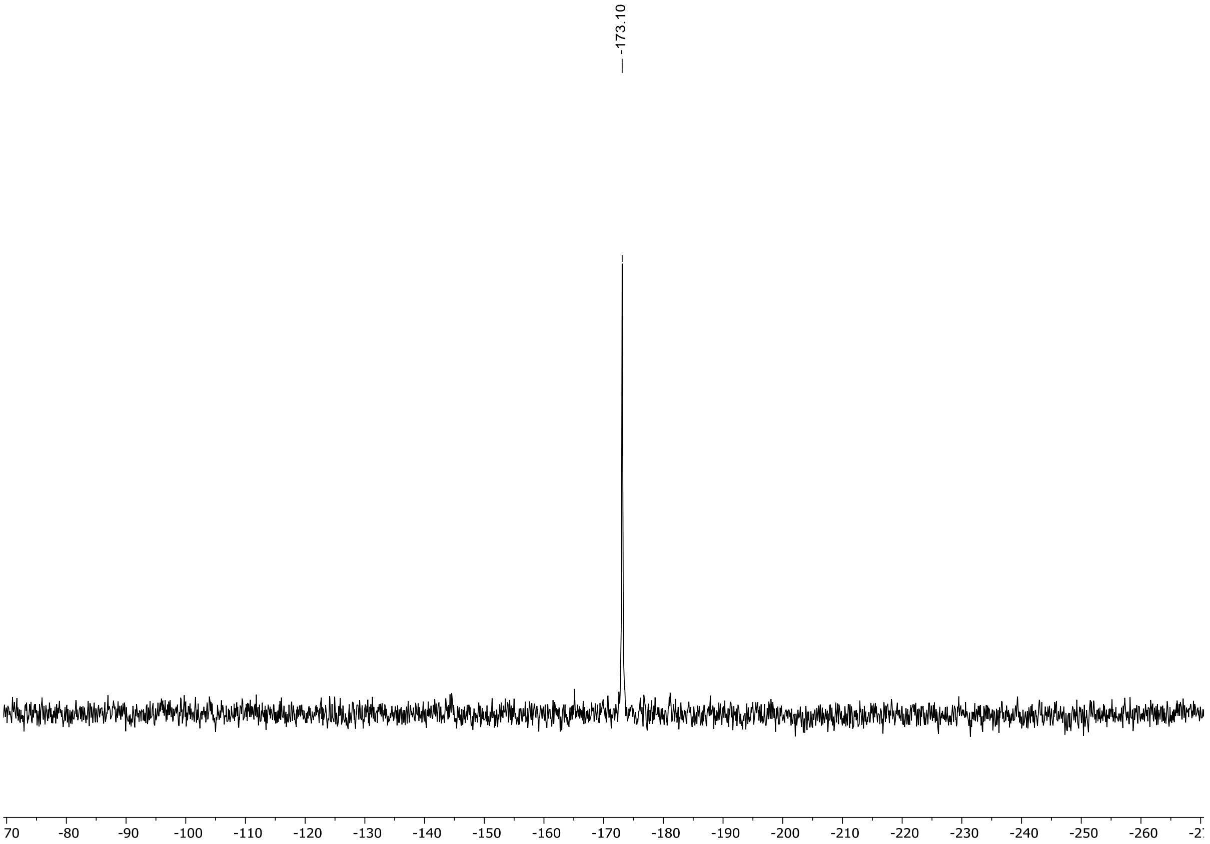

Figure S24. ^{119}Sn{^{1}H} NMR spectrum of ^{Dipp}TerSn(Se){N(^{i}Pr)C(N(SiMe$_3$)$_2$)N(^{i}Pr)} (**3b**) (149 MHz, C_6D_6, 298 K).

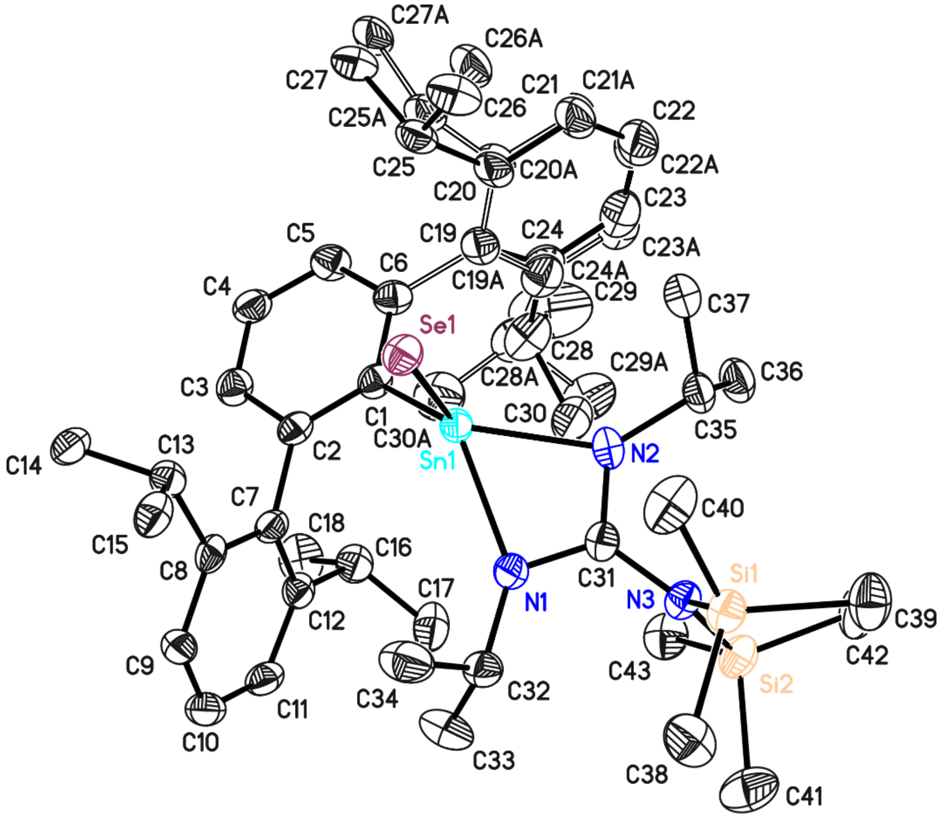

Figure S25. Molecular structure of ^{Dipp}TerSn(Se){N(^{i}Pr)C(N(SiMe$_3$)$_2$)N(^{i}Pr)} (**3b**) in the crystal. Thermal ellipsoids are drawn at the 50% probability level (hydrogen atoms have been omitted for clarity).

The ^{Dipp}Ter moiety was disordered about two positions. The disorder was treated with distance restraints and restraints for the anisotropic displacement parameters. The occupancy of the minor component refined to 0.075(9). The Platon SQUEEZE procedure was used to treat residual electron density associated with highly disordered solvent molecules.[S3] The number of electrons (15857) and the void volume (40738 Å^3) counts for approximately 317 molecules of hexane per unit cell.

Table S2. Bond lengths [Å] and angles [°] for **3b.**

Sn(1)-N(2)	2.151(4)	C(12)-C(16)	1.519(7)
Sn(1)-N(1)	2.170(4)	C(13)-C(15)	1.523(7)
Sn(1)-C(1)	2.184(4)	C(13)-C(14)	1.529(6)
Sn(1)-Se(1)	2.3818(6)	C(16)-C(18)	1.522(7)
Si(1)-N(3)	1.776(4)	C(16)-C(17)	1.550(7)
Si(1)-C(40)	1.845(5)	C(19)-C(24)	1.412(7)
Si(1)-C(38)	1.861(6)	C(19)-C(20)	1.426(7)
Si(1)-C(39)	1.864(5)	C(20)-C(21)	1.402(7)
N(1)-C(31)	1.329(6)	C(20)-C(25)	1.519(7)
N(1)-C(32)	1.479(6)	C(21)-C(22)	1.384(9)
C(1)-C(2)	1.403(7)	C(22)-C(23)	1.397(9)
C(1)-C(6)	1.405(7)	C(23)-C(24)	1.394(8)
Si(2)-N(3)	1.768(4)	C(24)-C(28)	1.529(8)
Si(2)-C(42)	1.855(6)	C(25)-C(26)	1.525(8)
Si(2)-C(43)	1.862(5)	C(25)-C(27)	1.531(8)
Si(2)-C(41)	1.878(6)	C(28)-C(30)	1.476(9)
N(2)-C(31)	1.344(6)	C(28)-C(29)	1.522(10)
N(2)-C(35)	1.483(6)	C(19A)-C(20A)	1.41(2)
C(2)-C(3)	1.410(6)	C(19A)-C(24A)	1.43(2)
C(2)-C(7)	1.504(7)	C(20A)-C(21A)	1.41(2)
N(3)-C(31)	1.399(6)	C(20A)-C(25A)	1.53(2)
C(3)-C(4)	1.370(7)	C(21A)-C(22A)	1.39(2)
C(4)-C(5)	1.392(7)	C(22A)-C(23A)	1.39(2)
C(5)-C(6)	1.400(7)	C(23A)-C(24A)	1.39(2)
C(6)-C(19A)	1.500(7)	C(24A)-C(28A)	1.53(2)
C(6)-C(19)	1.500(7)	C(25A)-C(27A)	1.51(2)
C(7)-C(8)	1.410(7)	C(25A)-C(26A)	1.53(2)
C(7)-C(12)	1.425(6)	C(28A)-C(30A)	1.50(2)
C(8)-C(9)	1.381(7)	C(28A)-C(29A)	1.52(3)
C(8)-C(13)	1.526(6)	C(32)-C(33)	1.504(7)
C(9)-C(10)	1.375(6)	C(32)-C(34)	1.509(7)
C(10)-C(11)	1.388(7)	C(35)-C(36)	1.500(7)
C(11)-C(12)	1.396(7)	C(35)-C(37)	1.507(7)

		C(5)-C(6)-C(19A)	114.7(4)
N(2)-Sn(1)-N(1)	61.49(15)	C(1)-C(6)-C(19A)	125.2(4)
N(2)-Sn(1)-C(1)	122.82(16)	C(5)-C(6)-C(19)	114.7(4)
N(1)-Sn(1)-C(1)	123.30(16)	C(1)-C(6)-C(19)	125.2(4)
N(2)-Sn(1)-Se(1)	113.02(10)	C(8)-C(7)-C(12)	120.0(4)
N(1)-Sn(1)-Se(1)	111.80(10)	C(8)-C(7)-C(2)	122.5(4)
C(1)-Sn(1)-Se(1)	114.27(12)	C(12)-C(7)-C(2)	116.7(4)
N(3)-Si(1)-C(40)	109.8(2)	C(9)-C(8)-C(7)	118.7(4)
N(3)-Si(1)-C(38)	110.7(2)	C(9)-C(8)-C(13)	120.0(4)
C(40)-Si(1)-C(38)	109.4(3)	C(7)-C(8)-C(13)	120.9(4)
N(3)-Si(1)-C(39)	109.2(2)	C(10)-C(9)-C(8)	122.6(4)
C(40)-Si(1)-C(39)	109.3(3)	C(9)-C(10)-C(11)	118.7(4)
C(38)-Si(1)-C(39)	108.4(3)	C(10)-C(11)-C(12)	121.9(5)
C(31)-N(1)-C(32)	121.4(4)	C(11)-C(12)-C(7)	118.0(4)
C(31)-N(1)-Sn(1)	92.5(3)	C(11)-C(12)-C(16)	119.9(4)
C(32)-N(1)-Sn(1)	140.3(3)	C(7)-C(12)-C(16)	121.9(4)
C(2)-C(1)-C(6)	119.0(4)	C(15)-C(13)-C(8)	114.0(4)
C(2)-C(1)-Sn(1)	119.4(3)	C(15)-C(13)-C(14)	109.0(4)
C(6)-C(1)-Sn(1)	120.1(3)	C(8)-C(13)-C(14)	110.0(4)
N(3)-Si(2)-C(42)	111.4(2)	C(12)-C(16)-C(18)	111.7(4)
N(3)-Si(2)-C(43)	108.9(2)	C(12)-C(16)-C(17)	111.2(4)
C(42)-Si(2)-C(43)	110.1(3)	C(18)-C(16)-C(17)	109.8(5)
N(3)-Si(2)-C(41)	110.1(2)	C(24)-C(19)-C(20)	119.8(5)
C(42)-Si(2)-C(41)	107.9(3)	C(24)-C(19)-C(6)	118.5(5)
C(43)-Si(2)-C(41)	108.4(3)	C(20)-C(19)-C(6)	121.1(4)
C(31)-N(2)-C(35)	123.1(4)	C(21)-C(20)-C(19)	118.7(5)
C(31)-N(2)-Sn(1)	93.0(3)	C(21)-C(20)-C(25)	119.8(5)
C(35)-N(2)-Sn(1)	139.3(3)	C(19)-C(20)-C(25)	121.3(5)
C(1)-C(2)-C(3)	119.4(4)	C(22)-C(21)-C(20)	120.8(6)
C(1)-C(2)-C(7)	126.3(4)	C(21)-C(22)-C(23)	120.7(6)
C(3)-C(2)-C(7)	114.0(4)	C(24)-C(23)-C(22)	120.1(6)
C(31)-N(3)-Si(2)	118.0(3)	C(23)-C(24)-C(19)	119.8(5)
C(31)-N(3)-Si(1)	118.4(3)	C(23)-C(24)-C(28)	119.4(5)
Si(2)-N(3)-Si(1)	123.6(2)	C(19)-C(24)-C(28)	120.8(5)
C(4)-C(3)-C(2)	121.4(5)	C(20)-C(25)-C(26)	113.6(5)
C(3)-C(4)-C(5)	119.2(5)	C(20)-C(25)-C(27)	110.9(5)
C(4)-C(5)-C(6)	120.9(5)	C(26)-C(25)-C(27)	109.6(5)
C(5)-C(6)-C(1)	119.9(4)	C(30)-C(28)-C(29)	108.0(6)

C(30)-C(28)-C(24)	114.3(6)	C(27A)-C(25A)-C(26A)	107(3)
C(29)-C(28)-C(24)	113.6(7)	C(20A)-C(25A)-C(26A)	114(3)
C(20A)-C(19A)-C(24A)	121(2)	C(30A)-C(28A)-C(29A)	108(3)
C(20A)-C(19A)-C(6)	125.8(17)	C(30A)-C(28A)-C(24A)	114(3)
C(24A)-C(19A)-C(6)	112.5(16)	C(29A)-C(28A)-C(24A)	112(3)
C(21A)-C(20A)-C(19A)	117(2)	N(1)-C(31)-N(2)	111.5(4)
C(21A)-C(20A)-C(25A)	117(2)	N(1)-C(31)-N(3)	124.6(4)
C(19A)-C(20A)-C(25A)	125(3)	N(2)-C(31)-N(3)	123.9(4)
C(22A)-C(21A)-C(20A)	122(3)	N(1)-C(32)-C(33)	111.6(4)
C(23A)-C(22A)-C(21A)	120(3)	N(1)-C(32)-C(34)	110.5(4)
C(22A)-C(23A)-C(24A)	120(3)	C(33)-C(32)-C(34)	111.6(5)
C(23A)-C(24A)-C(19A)	120(2)	N(2)-C(35)-C(36)	112.1(4)
C(23A)-C(24A)-C(28A)	116(3)	N(2)-C(35)-C(37)	109.3(4)
C(19A)-C(24A)-C(28A)	122(3)	C(36)-C(35)-C(37)	111.8(4)
C(27A)-C(25A)-C(20A)	113(3)		

Reaction of ^{Mes}TerSn{N(^{i}Pr)C(N(SiMe$_3$)$_2$)N(^{i}Pr)} (2a) with N_2O – Formation of ^{Mes}TerSn(OSiMe$_3$){N(^{i}Pr)C(=NSiMe$_3$)N(^{i}Pr)} (4a)

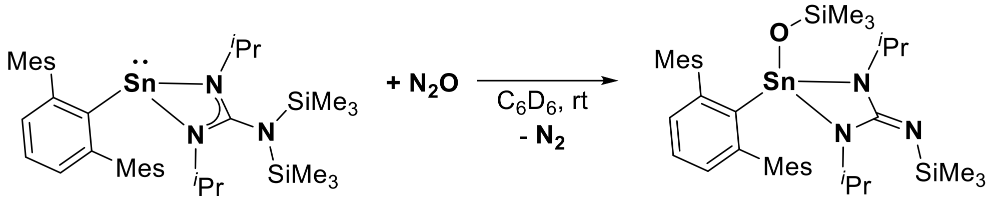

^{Mes}TerSn{N(SiMe$_3$)$_2$} (**1a**) (0.030 g, 0.051 mmol) was dissolved in 0.3 mL of C_6D_6 followed by addition of ^{i}PrN=C=N^{i}Pr (6.4 mg, 0.051 mmol) in 0.3 mL of C_6D_6. The reaction progress was monitored by ^{1}H NMR spectroscopy until both starting materials have been consumed and ^{Mes}TerSn{N(^{i}Pr)C(N(SiMe$_3$)$_2$)N(^{i}Pr)} (**2a**) has formed. The solution was freeze-pump-thaw degassed three times and backfilled with approximately 1 bar N_2O. The reaction progress was monitored by ^{1}H NMR spectroscopy (Figure S26) and shows the clean formation of a single new product. All volatile components were removed under vacuum to give ^{Mes}TerSn(OSiMe$_3$){N(^{i}Pr)C(=NSiMe$_3$)N(^{i}Pr)} (**4a**) as a colourless solid.

Yield: 0.029 g (0.039 mmol; 76%).
^{1}H NMR (400 MHz, C_6D_6, 298 K): δ = 0.17 (s, 9H, Si(CH$_3$)$_3$), 0.45 (s, 9H, Si(CH$_3$)$_3$), 0.82 (d, $^3J_{H,H}$ = 6.7 Hz, 6H, CH(CH_3)$_2$), 1.08 (d, $^3J_{H,H}$ = 6.4 Hz, 6H, CH(CH_3)$_2$), 2.09 (s, 12H, CH$_3$), 2.16 (s, 6H, CH$_3$), 3.17 (hept, $^3J_{H,H}$ = 6.5 Hz, 2H, CH(CH$_3$)$_2$), 6.75-6.76 (m, 2H, CH$_{Aryl}$), 6.80 (s, 4H, CH$_{Aryl}$), 7.08-7.10 (m, 1H, CH$_{Aryl}$) ppm.
^{13}C{^{1}H} NMR (101 MHz, C_6D_6, 298 K): δ = 3.1 (Si(CH$_3$)$_3$), 4.2 (Si(CH$_3$)$_3$), 21.1 (CH$_3$), 21.4 (CH$_3$), 23.8 (CH($\underline{C}$H$_3$)$_2$), 24.4 (CH($\underline{C}$H$_3$)$_2$), 45.2 ($\underline{C}$H(CH$_3$)$_2$), 129.5 (CH$_{Aryl}$), 129.7 (CH$_{Aryl}$), 132.2 (CH$_{Aryl}$), 137.0 (C$_{q,Aryl}$), 138.7 (C$_{q,Aryl}$), 139.5 (C$_{q,Aryl}$), 140.8 (C$_q$Sn), 149.2 (C$_{q,Aryl}$), 155.9 (C$_q$(N^{i}Pr)$_2$N(SiMe$_3$)$_2$) ppm.
^{29}Si{^{1}H} NMR (80 MHz, C_6D_6, 298 K): δ = -23.5, 10.9 ppm. (assigned by ^{1}H/^{29}Si HMBC)
^{119}Sn{^{1}H} NMR (149 MHz, C_6D_6, 298 K): δ = -209.9 ppm.
MS (LIFDI): m/z calcd. for $C_{37}H_{57}N_3OSi_2Sn$: 735.3062; found: 735.2.
EA: Anal. calcd. for $C_{37}H_{57}N_3Si_2Sn$: C, 60.48; H, 7.82; N, 5.72; Found: C, 61.09; H, 8.19; N, 5.35.

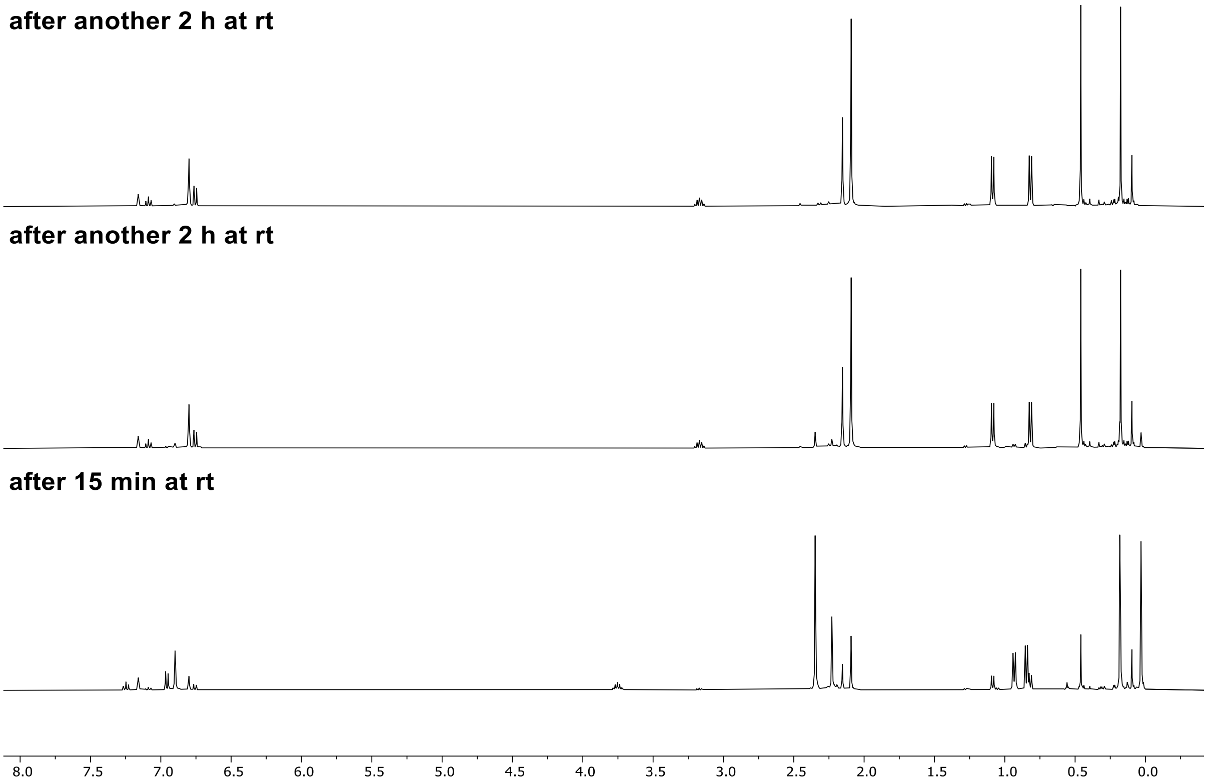

Figure S26. Monitoring of the reaction of ^{Mes}TerSn{N(^{i}Pr)C(N(SiMe$_3$)$_2$)N(^{i}Pr)} (**2a**) and N_2O via ^{1}H NMR spectroscopy (400 MHz, C_6D_6, 298 K).

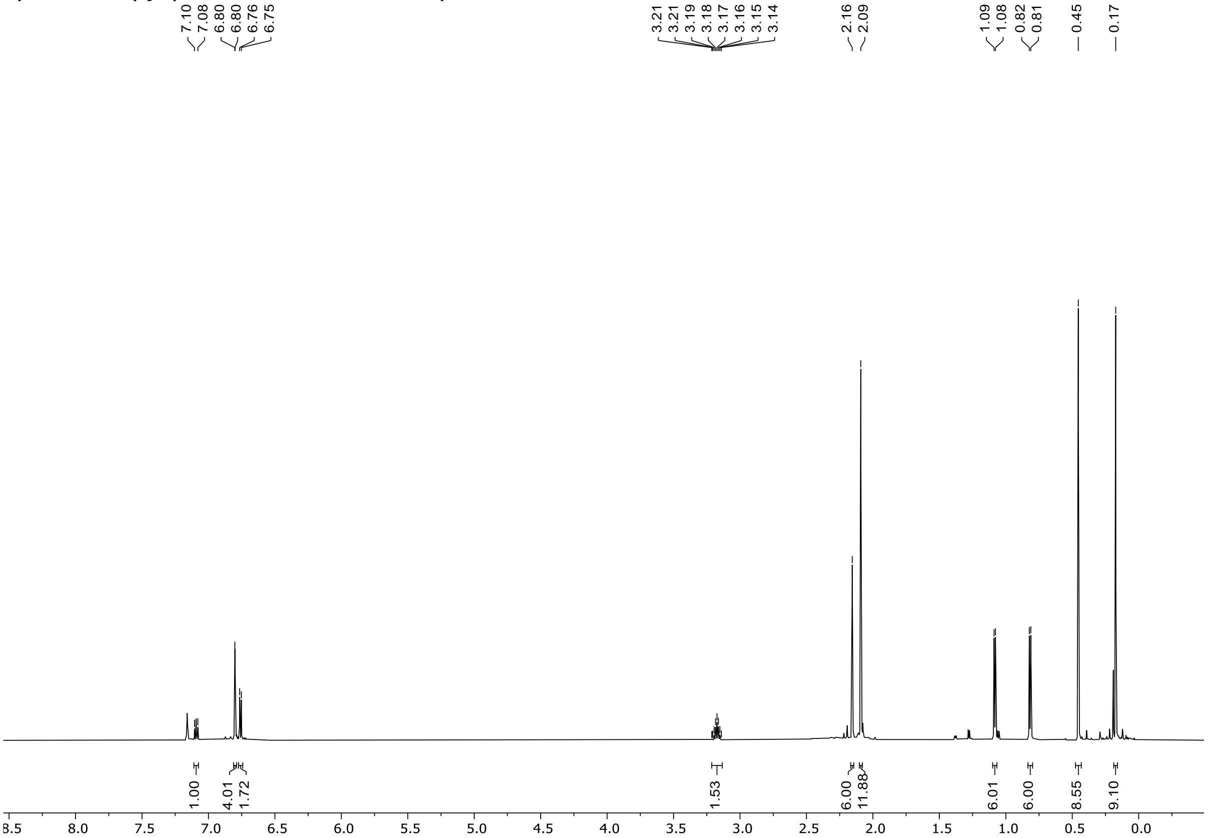

Figure S27. ^{1}H NMR spectrum of ^{Mes}TerSn(OSiMe$_3$){N(^{i}Pr)C(=NSiMe$_3$)N(^{i}Pr)} (**4a**) (400 MHz, C_6D_6, 298 K).

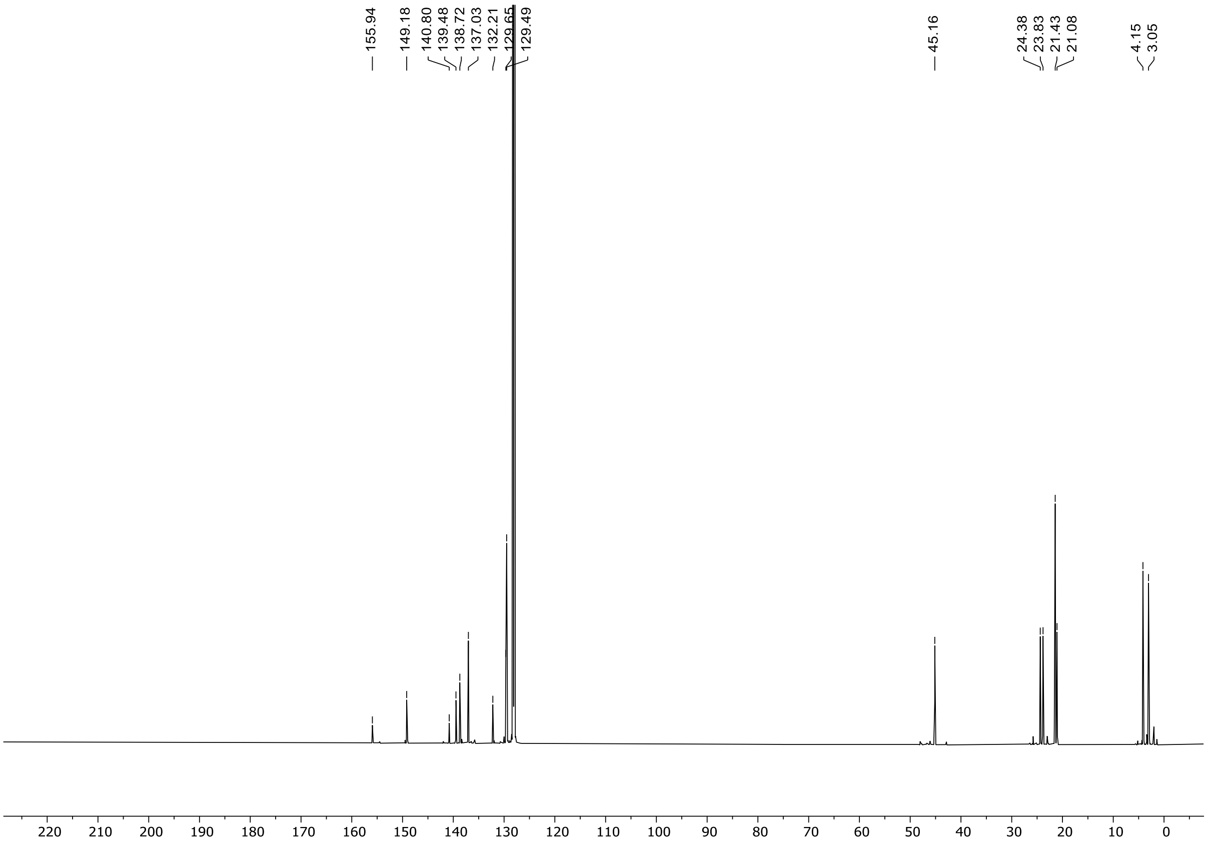

Figure S28. $^{13}C\{^1H\}$ NMR spectrum of ^{Mes}TerSn(OSiMe$_3$){N(^{i}Pr)C(=NSiMe$_3$)N(^{i}Pr)} (**4a**) (126 MHz, C_6D_6, 298 K).

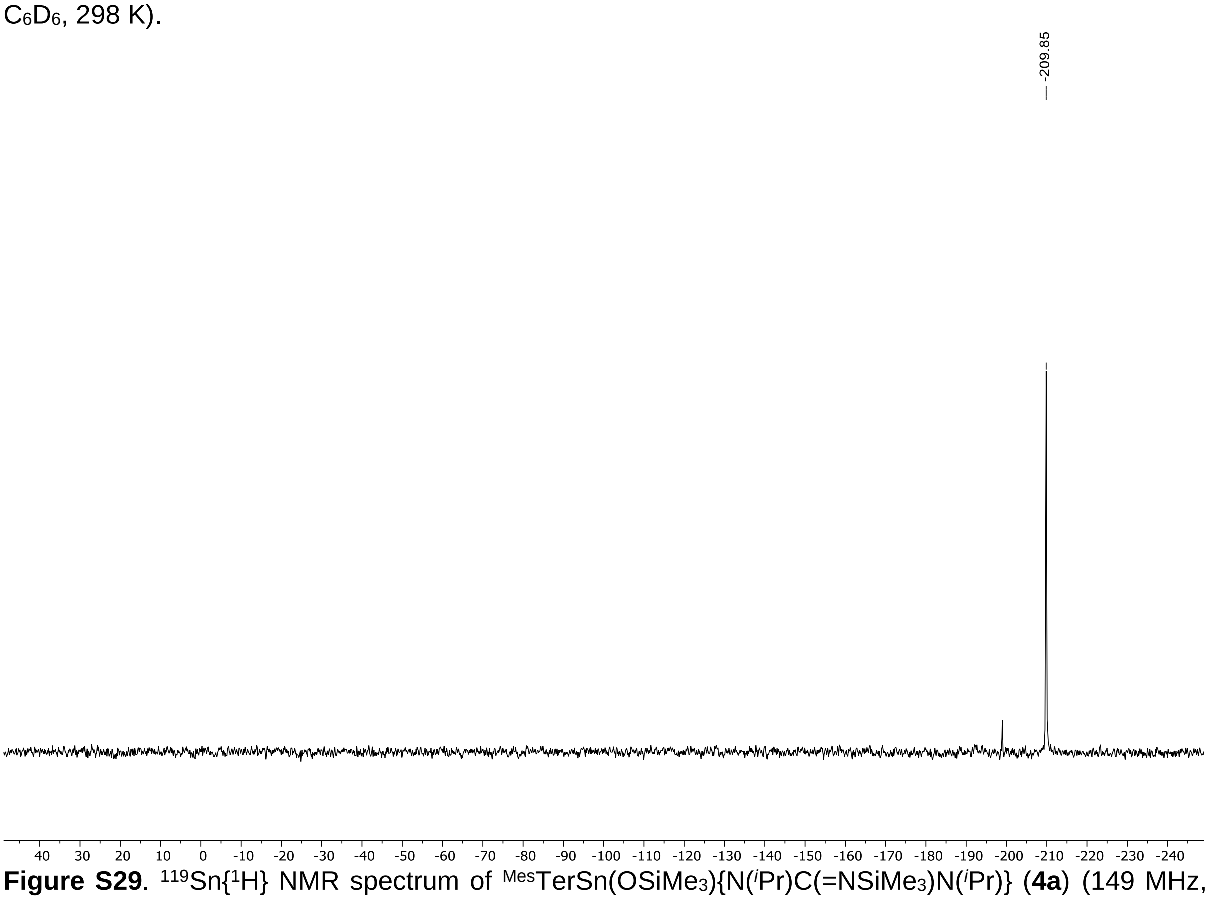

Figure S29. $^{119}Sn\{^1H\}$ NMR spectrum of ^{Mes}TerSn(OSiMe$_3$){N(^{i}Pr)C(=NSiMe$_3$)N(^{i}Pr)} (**4a**) (149 MHz, C_6D_6, 298 K).

Reaction of ^{Dipp}TerSn{N(^{i}Pr)C(N(SiMe$_3$)$_2$)N(^{i}Pr)} (2b) with N_2O – Formation of ^{Dipp}TerSn(OSiMe$_3$){N(^{i}Pr)C(=NSiMe$_3$)N(^{i}Pr)} (4b)

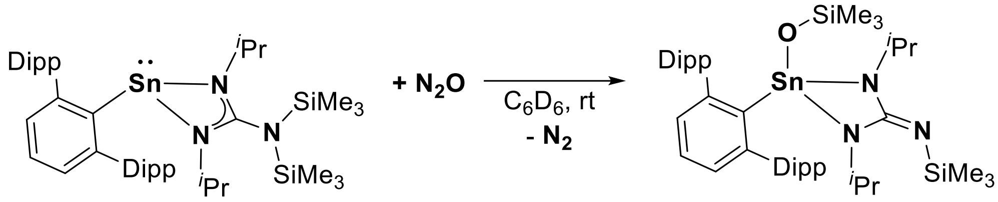

^{Dipp}TerSn{N(SiMe$_3$)$_2$} (**1b**) (0.030 g, 0.039 mmol) was dissolved in 0.3 mL of C_6D_6 followed by addition of ^{i}PrN=C=N^{i}Pr (5.0 mg, 0.039 mmol) in 0.3 mL of C_6D_6. The reaction progress was monitored by ^{1}H NMR spectroscopy until both starting materials have been consumed and ^{Dipp}TerSn{N(^{i}Pr)C(N(SiMe$_3$)$_2$)N(^{i}Pr)} (**2b**) has formed. The solution was freeze-pump-thaw degassed three times and backfilled with approximately 1 bar N_2O. The reaction progress was monitored by ^{1}H NMR spectroscopy and shows the clean formation of a single new product. All volatile components were removed under vacuum, followed by the addition of 0.6 mL of *n*-hexane and filtration. The clear colourless solution was stored at -30 °C to give ^{Dipp}TerSn(OSiMe$_3$){N(^{i}Pr)C(=NSiMe$_3$)N(^{i}Pr)} (**4b**) as a colourless crystalline material. Crystals obtained this way were suitable for single crystal X-ray diffraction.

Yield: 0.012 g (0.033 mmol; 65%).
^{1}H NMR (400 MHz, C_6D_6, 298 K): δ = 0.05 (s, 9H, Si(CH$_3$)$_3$), 0.44 (s, 9H, Si(CH$_3$)$_3$), 0.85 (d, $^3J_{H,H}$ = 6.3 Hz, 6H, NCH(C*H*$_3$)$_2$), 0.98 (d, $^3J_{H,H}$ = 6.7 Hz, 12H, CH(C*H*$_3$)$_2$), 1.01 (d, $^3J_{H,H}$ = 6.4 Hz, 6H, NCH(C*H*$_3$)$_2$), 1.34 (d, $^3J_{H,H}$ = 6.9 Hz, 12H, CH(C*H*$_3$)$_2$), 2.81 (hept, $^3J_{H,H}$ = 6.8 Hz, 4H, C*H*(CH$_3$)$_2$), 3.68 (hept, $^3J_{H,H}$ = 6.4 Hz, 2H, NC*H*(CH$_3$)$_2$), 6.99-7.01 (m, 1H, CH_{Aryl}), 7.06-7.07 (m, 2H, CH_{Aryl}), 7.14-7.15 (m, 4H, CH_{Aryl})*, 7.27-7.29 (m, 2H, CH_{Aryl}) ppm.
* = overlap with C_6D_5H signal
^{13}C{^{1}H} NMR (101 MHz, C_6D_6, 298 K): δ = 3.2 (Si(CH$_3$)$_3$), 4.3 (Si(CH$_3$)$_3$), 23.6 (CH($\underline{C}$H$_3$)$_2$), 24.5 (NCH($\underline{C}$H$_3$)$_2$), 25.6 (NCH($\underline{C}$H$_3$)$_2$), 26.0 (CH($\underline{C}$H$_3$)$_2$), 31.4 ($\underline{C}$H(CH$_3$)$_2$), 44.6 (N$\underline{C}$H(CH$_3$)$_2$), 123.8 (CH_{Aryl}), 129.9 (CH_{Aryl}), 130.0 (CH_{Aryl}), 132.0 (CH_{Aryl}), 139.7 ($C_{q,Aryl}$), 139.9 (C_qSn), 147.4 ($C_{q,Aryl}$), 148.5 ($C_{q,Aryl}$), 155.2 (C_q(N^{i}Pr)$_2$N(SiMe$_3$)$_2$) ppm.
^{29}Si{^{1}H} NMR (80 MHz, C_6D_6, 298 K): δ = -23.5, 10.1 ppm. (assigned by ^{1}H/^{29}Si HMBC)
^{119}Sn{^{1}H} NMR (149 MHz, C_6D_6, 298 K): δ = -203.7 ppm.
EA: Anal. calcd. for $C_{43}H_{69}N_3OSi_2Sn$: C, 63.07; H, 8.49; N, 5.13; Found: C, 63.00; H, 7.97; N, 5.43.

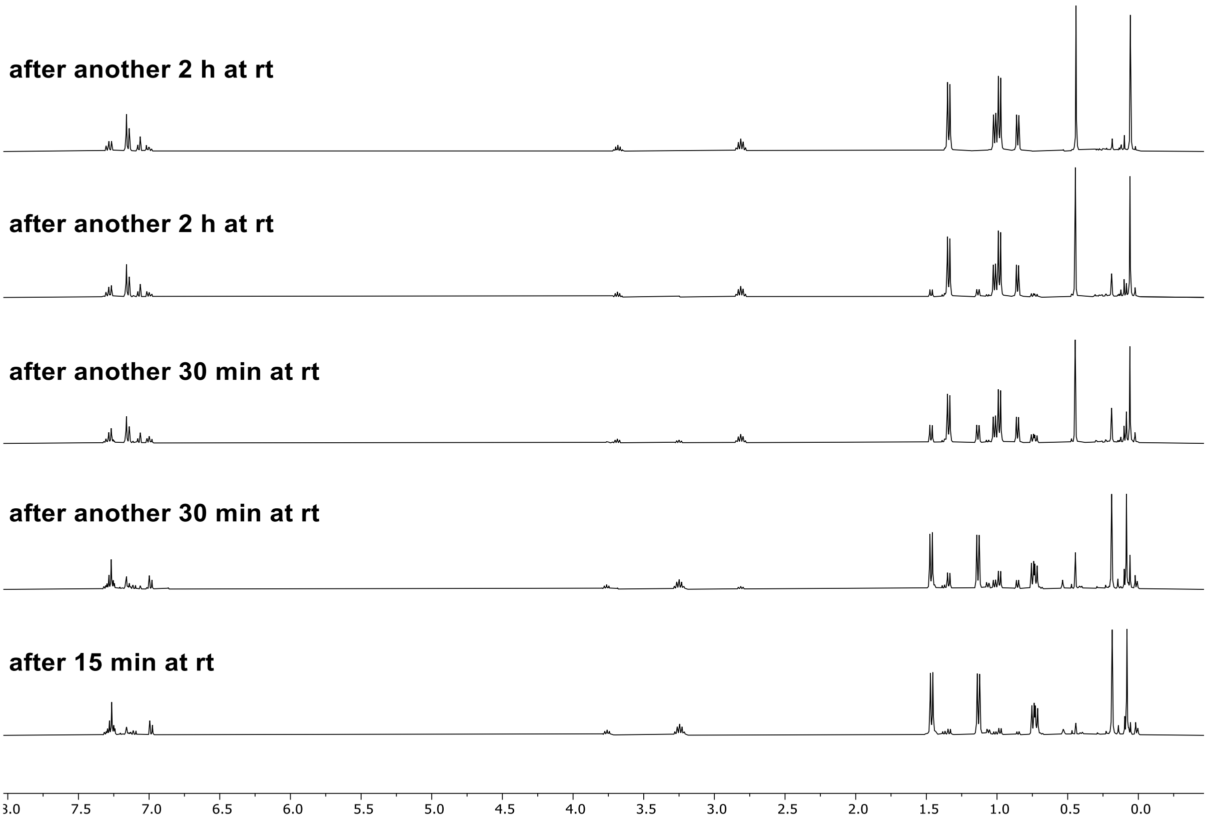

Figure S30. Monitoring of the reaction of ^{Dipp}TerSn{N(^{i}Pr)C(N(SiMe$_3$)$_2$)N(^{i}Pr)} (**2b**) and N_2O via ^{1}H NMR spectroscopy (400 MHz, C_6D_6, 298 K).

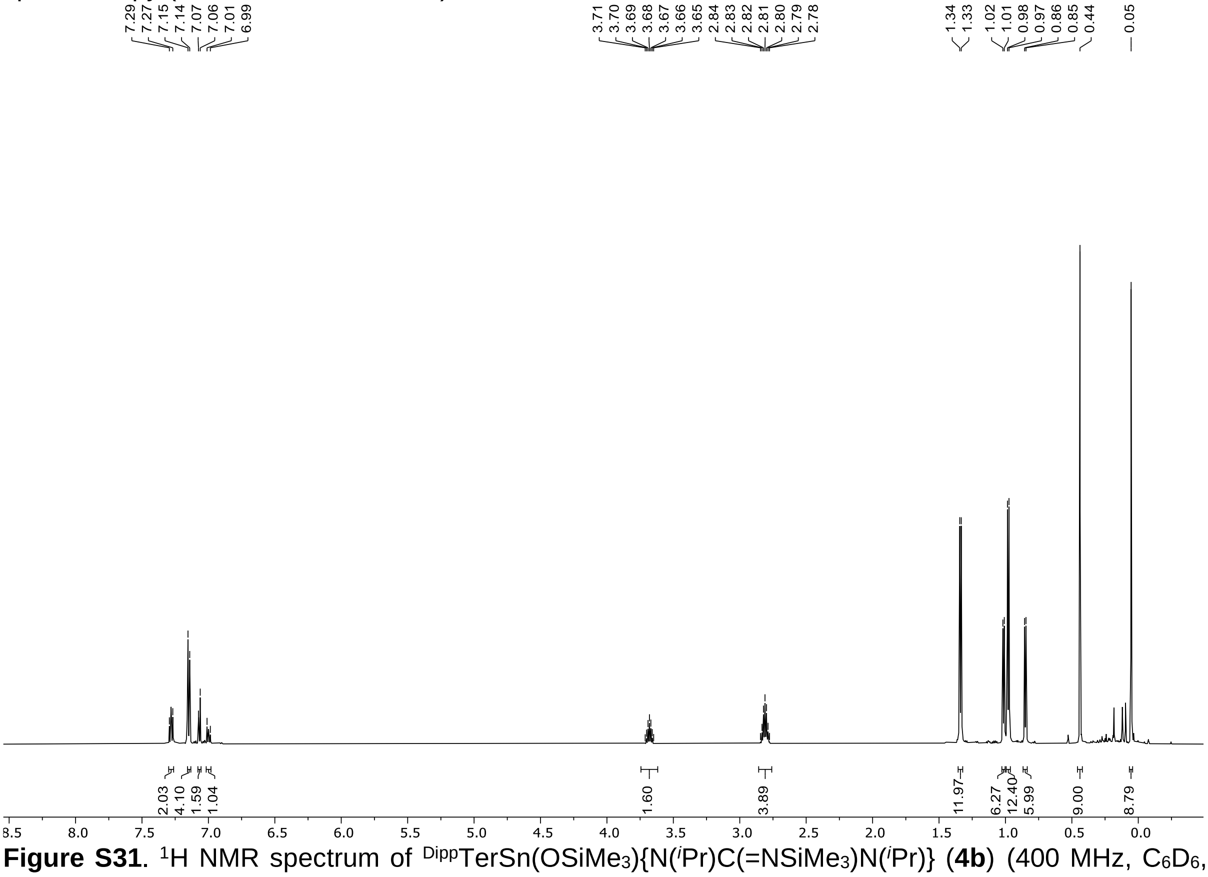

Figure S31. ^{1}H NMR spectrum of ^{Dipp}TerSn(OSiMe$_3$){N(^{i}Pr)C(=NSiMe$_3$)N(^{i}Pr)} (**4b**) (400 MHz, C_6D_6, 298 K).

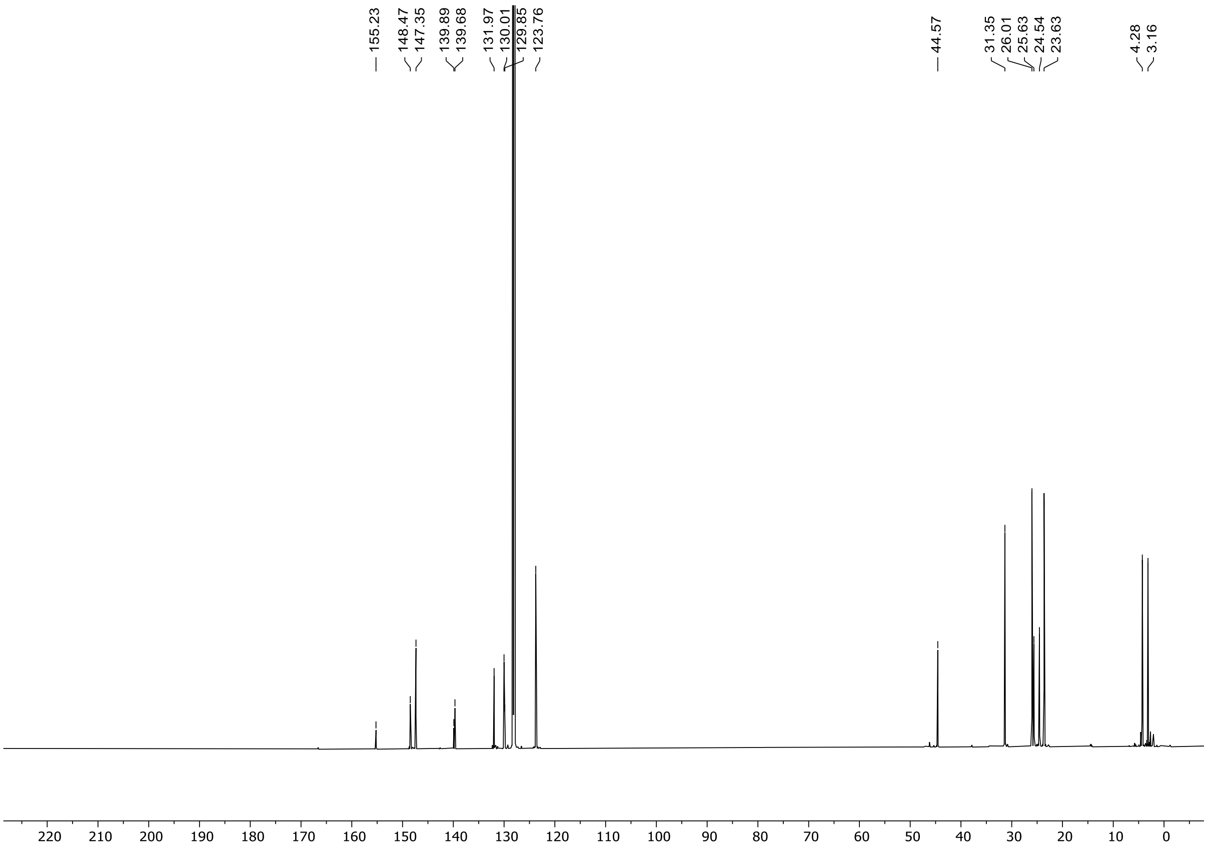

Figure S32. $^{13}C\{^1H\}$ NMR spectrum of $^{Dipp}TerSn(OSiMe_3)\{N(^iPr)C(=NSiMe_3)N(^iPr)\}$ (**4b**) (126 MHz, C_6D_6, 298 K).

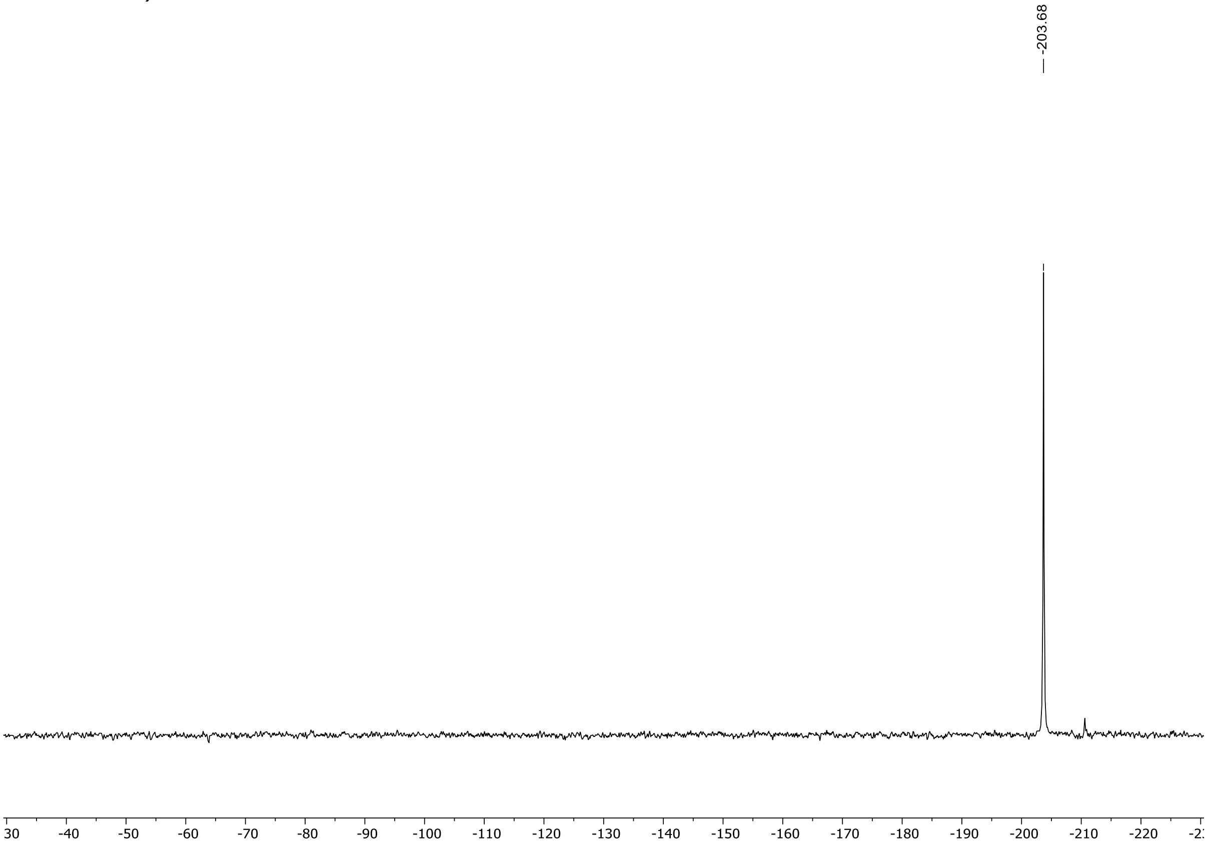

Figure S33. $^{119}Sn\{^1H\}$ NMR spectrum of $^{Dipp}TerSn(OSiMe_3)\{N(^iPr)C(=NSiMe_3)N(^iPr)\}$ (**4b**) (149 MHz, C_6D_6, 298 K).

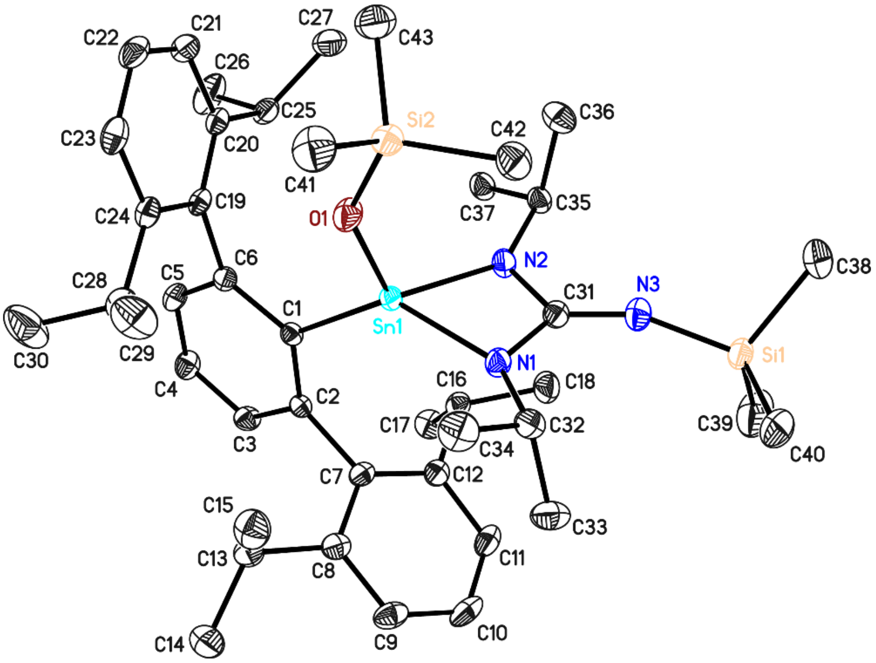

Figure S34. Molecular structure of ^{Dipp}TerSn(OSiMe$_3$){N(^{i}Pr)C(=NSiMe$_3$)N(^{i}Pr)} (**4b**) in the crystal. Thermal ellipsoids are drawn at the 50% probability level (hydrogen atoms have been omitted for clarity).

Table S3. Bond lengths [Å] and angles [°] for **4b.**

Si(2)-O(1)	1.6394(14)	N(3)-C(31)	1.263(2)
Si(2)-C(43)	1.859(2)	C(3)-C(4)	1.381(3)
Si(2)-C(42)	1.860(2)	C(4)-C(5)	1.386(3)
Si(2)-C(41)	1.861(2)	C(5)-C(6)	1.396(2)
N(2)-C(31)	1.400(2)	C(6)-C(19)	1.506(2)
N(2)-C(35)	1.454(2)	C(7)-C(12)	1.406(3)
N(2)-Sn(1)	2.0389(15)	C(7)-C(8)	1.416(2)
C(2)-C(3)	1.399(2)	C(8)-C(9)	1.395(3)
C(2)-C(1)	1.409(2)	C(8)-C(13)	1.523(3)
C(2)-C(7)	1.507(2)	C(9)-C(10)	1.378(3)
C(1)-C(6)	1.416(2)	C(10)-C(11)	1.380(3)
C(1)-Sn(1)	2.1373(17)	C(11)-C(12)	1.395(3)
Sn(1)-O(1)	1.9427(13)	C(12)-C(16)	1.523(3)
Sn(1)-N(1)	2.0466(15)	C(13)-C(15)	1.522(3)
Sn(1)-C(31)	2.5587(18)	C(13)-C(14)	1.528(3)
Si(1)-N(3)	1.6762(16)	C(16)-C(17)	1.530(3)
Si(1)-C(38)	1.861(2)	C(16)-C(18)	1.530(3)
Si(1)-C(39)	1.863(2)	C(19)-C(20)	1.406(2)
Si(1)-C(40)	1.875(2)	C(19)-C(24)	1.411(2)
N(1)-C(31)	1.396(2)	C(20)-C(21)	1.395(2)
N(1)-C(32)	1.458(2)	C(20)-C(25)	1.523(2)

C(21)-C(22)	1.379(3)	N(3)-Si(1)-C(38)	107.74(9)
C(22)-C(23)	1.382(3)	N(3)-Si(1)-C(39)	108.21(10)
C(23)-C(24)	1.394(3)	C(38)-Si(1)-C(39)	109.16(12)
C(24)-C(28)	1.526(3)	N(3)-Si(1)-C(40)	117.69(10)
C(25)-C(26)	1.530(3)	C(38)-Si(1)-C(40)	105.66(10)
C(25)-C(27)	1.535(3)	C(39)-Si(1)-C(40)	108.15(13)
C(28)-C(29)	1.519(3)	Si(2)-O(1)-Sn(1)	139.79(8)
C(28)-C(30)	1.523(3)	C(31)-N(1)-C(32)	121.60(15)
C(32)-C(34)	1.520(3)	C(31)-N(1)-Sn(1)	94.10(10)
C(32)-C(33)	1.521(3)	C(32)-N(1)-Sn(1)	142.93(12)
C(35)-C(37)	1.517(2)	C(31)-N(3)-Si(1)	158.25(15)
C(35)-C(36)	1.527(2)	C(4)-C(3)-C(2)	121.19(16)
		C(3)-C(4)-C(5)	119.69(16)
O(1)-Si(2)-C(43)	110.32(9)	C(4)-C(5)-C(6)	121.41(16)
O(1)-Si(2)-C(42)	112.23(8)	C(5)-C(6)-C(1)	118.54(16)
C(43)-Si(2)-C(42)	108.56(10)	C(5)-C(6)-C(19)	116.22(15)
O(1)-Si(2)-C(41)	107.80(9)	C(1)-C(6)-C(19)	125.06(15)
C(43)-Si(2)-C(41)	108.94(11)	C(12)-C(7)-C(8)	120.67(16)
C(42)-Si(2)-C(41)	108.93(10)	C(12)-C(7)-C(2)	120.78(15)
C(31)-N(2)-C(35)	121.55(14)	C(8)-C(7)-C(2)	118.00(15)
C(31)-N(2)-Sn(1)	94.31(10)	C(9)-C(8)-C(7)	118.22(17)
C(35)-N(2)-Sn(1)	142.00(12)	C(9)-C(8)-C(13)	119.55(17)
C(3)-C(2)-C(1)	118.91(15)	C(7)-C(8)-C(13)	122.23(16)
C(3)-C(2)-C(7)	116.10(15)	C(10)-C(9)-C(8)	121.30(18)
C(1)-C(2)-C(7)	124.83(15)	C(9)-C(10)-C(11)	120.08(17)
C(2)-C(1)-C(6)	120.15(15)	C(10)-C(11)-C(12)	121.23(18)
C(2)-C(1)-Sn(1)	119.55(12)	C(11)-C(12)-C(7)	118.47(17)
C(6)-C(1)-Sn(1)	119.89(12)	C(11)-C(12)-C(16)	119.66(17)
O(1)-Sn(1)-N(2)	113.06(6)	C(7)-C(12)-C(16)	121.67(16)
O(1)-Sn(1)-N(1)	107.02(6)	C(15)-C(13)-C(8)	111.18(16)
N(2)-Sn(1)-N(1)	65.94(6)	C(15)-C(13)-C(14)	109.68(17)
O(1)-Sn(1)-C(1)	110.81(6)	C(8)-C(13)-C(14)	112.91(17)
N(2)-Sn(1)-C(1)	118.46(6)	C(12)-C(16)-C(17)	110.36(15)
N(1)-Sn(1)-C(1)	134.23(6)	C(12)-C(16)-C(18)	112.86(16)
O(1)-Sn(1)-C(31)	112.12(6)	C(17)-C(16)-C(18)	109.91(16)
N(2)-Sn(1)-C(31)	33.07(6)	C(20)-C(19)-C(24)	120.59(16)
N(1)-Sn(1)-C(31)	32.98(6)	C(20)-C(19)-C(6)	120.77(15)
C(1)-Sn(1)-C(31)	136.30(6)	C(24)-C(19)-C(6)	118.30(15)

C(21)-C(20)-C(19)	118.61(16)	C(30)-C(28)-C(24)	111.10(16)
C(21)-C(20)-C(25)	119.04(16)	N(3)-C(31)-N(1)	129.75(17)
C(19)-C(20)-C(25)	122.33(16)	N(3)-C(31)-N(2)	124.90(17)
C(22)-C(21)-C(20)	121.24(17)	N(1)-C(31)-N(2)	105.33(14)
C(21)-C(22)-C(23)	119.84(17)	N(3)-C(31)-Sn(1)	174.26(14)
C(22)-C(23)-C(24)	121.29(17)	N(1)-C(31)-Sn(1)	52.92(8)
C(23)-C(24)-C(19)	118.43(16)	N(2)-C(31)-Sn(1)	52.62(8)
C(23)-C(24)-C(28)	119.32(16)	N(1)-C(32)-C(34)	110.55(15)
C(19)-C(24)-C(28)	122.23(16)	N(1)-C(32)-C(33)	110.92(15)
C(20)-C(25)-C(26)	111.02(15)	C(34)-C(32)-C(33)	111.35(16)
C(20)-C(25)-C(27)	111.62(16)	N(2)-C(35)-C(37)	109.95(14)
C(26)-C(25)-C(27)	109.53(17)	N(2)-C(35)-C(36)	111.08(15)
C(29)-C(28)-C(30)	110.68(18)	C(37)-C(35)-C(36)	111.62(15)
C(29)-C(28)-C(24)	112.63(17)		

Reaction of ^{Mes}TerSnCl (5) with lithium dicyclohexylamide ($LiNCy_2$) – Formation of ^{Mes}TerSn{NCy_2} (6)

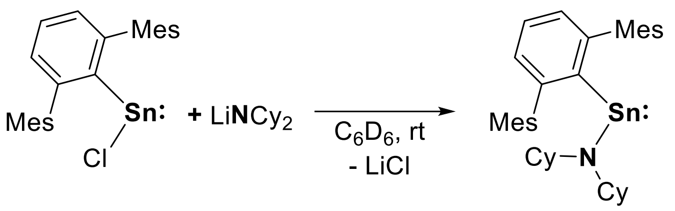

Synthesis of lithium dicyclochexylamide: Dicyclohexylamine (5.000 g, 27.58 mmol) was dissolved in 20 mL of *n*-hexane followed by dropwise addition of *n*-butyllithium (2.5 M in hexanes; 11.6 mL, 28.95 mmol). The suspension was stirred over night at room temperate. The supernatant was removed and the colourless solid dried under vacuum to give lithium dicyclohexylamide as a colourless solid.

Yield: 4.701 g (25.10 mmol; 91%).
^{1}H NMR (400 MHz, C_6D_6, 298 K): δ = 1.04-1.12 (m, 4H, CH_2), 1.16-1.28 (m, 2H, CH_2), 1.36-1.45 (m, 4H, CH_2), 1.68-1.74 (m, 2H, CH_2), 1.83-1.88 (m, 4H, CH_2), 1.93-1.95 (m, 2H, CH_2), 2.11-2.13 (m, 2H, CH_2), 2.59-2.63 (m, 1H, CH), 2.79-2.82 (m, 1H, CH) ppm.
^{13}C{^{1}H} NMR (101 MHz, C_6D_6, 298 K): δ = 27.0 (CH_2), 27.1 (CH_2), 27.2 (CH_2), 27.4 (CH_2), 40.1 (CH_2), 40.9 (CH_2), 58.9 (CH), 60.5 (CH) ppm.
^{7}Li NMR (140 MHz, C_6D_6, 298 K): δ = 2.9 ppm.

Synthesis of 6: ^{Mes}TerSnCl (**5**) (0.030 g, 0.064 mmol) and $LiNCy_2$ (0.012 g, 0.064 mmol) were suspended in 0.6 mL of C_6D_6 and the reaction progress was monitored by ^{1}H NMR spectroscopy until **5** was completely consumed. All volatiles were removed under vacuum, the residue was suspended in 0.6 mL of *n*-hexane, filtered, and stored at -30 °C to give ^{Mes}TerSn{NCy_2} (**6**) as a colorless crystalline material. Crystals obtained this way were suitable for single crystal X-ray diffraction.

Yield: 0.018 g (0.029 mmol; 45%).
^{1}H NMR (400 MHz, C_6D_6, 298 K): δ = 0.88-0.94 (m, 2H, $CH_{2,Cy}$), 1.13-1.15 (m, 4H, $CH_{2,Cy}$), 1.27-1.33 (m, 4H, $CH_{2,Cy}$), 1.43-1.45 (m, 6H, $CH_{2,Cy}$), 1.61-1.62 (m, 4H, $CH_{2,Cy}$), 2.16 (s, 6H, CH_3), 2.25 (s(br), 6H, CH_3), 2.36 (s(br), 6H, CH_3), 3.44 (m(br), 2H, CH_{Cy}), 6.82-6.90 (m(br), 4H, CH_{Aryl}), 7.12-7.13 (m, 2H, CH_{Aryl}), 7.32-7.35 (m, 1H, CH_{Aryl}) ppm.
^{13}C{^{1}H} NMR (126 MHz, C_6D_6, 298 K): δ = 20.7 ($CH_{2,Cy}$), 21.3 ($CH_{2,Cy}$), 22.1 ($CH_{2,Cy}$), 25.7 (CH_3), 27.0 (2xCH_3), 41.4 (br, $CH_{2,Cy}$)*, 62.9 (br, CH_{Cy}), 127.8 (CH_{Aryl})**, 128.1 (CH_{Aryl}), 128.7 (br, CH_{Aryl}), 129.0 (br, CH_{Aryl}), 134.1 (br, $C_{q,Aryl}$), 136.5 ($C_{q,Aryl}$), 139.4 ($C_{q,Aryl}$), 147.6 ($C_{q,Aryl}$), 174.0 (C_qSn) ppm.
* = assigned to four CH_2 groups by ^{1}H/^{13}C HSQC experiment
** = overlap with C_6D_6 signal
^{119}Sn{^{1}H} NMR (149 MHz, C_6D_6, 298 K): δ = 924.3 ppm.
MS (LIFDI): m/z calcd. for $C_{36}H_{47}NSn$: 613.2730; found: 613.4.
EA: Anal. calcd. for $C_{36}H_{47}NSn$: C, 70.60; H, 7.73; N, 2.29; Found: C, 70.34; H, 7.87; N, 2.31.

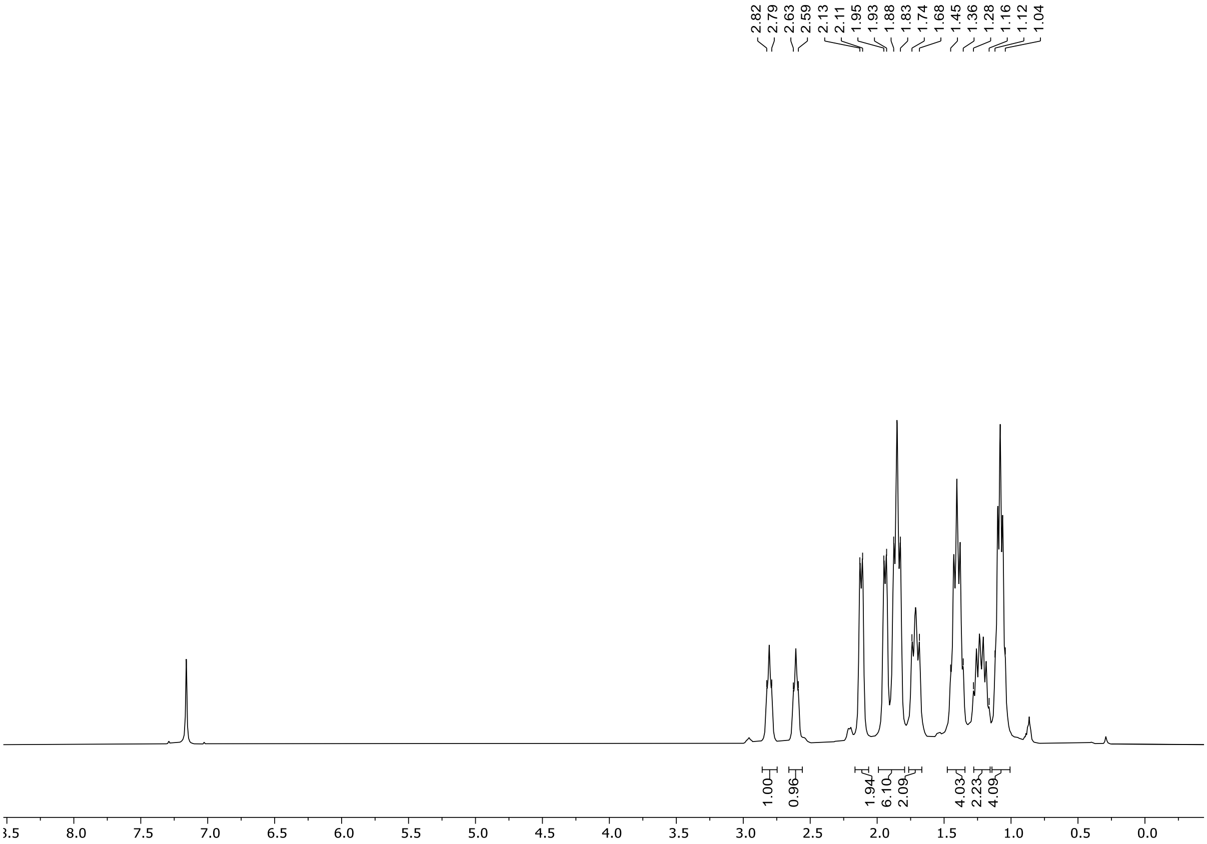

Figure S35. ^{1}H NMR spectrum of $LiNCy_2$ (400 MHz, C_6D_6, 298 K).

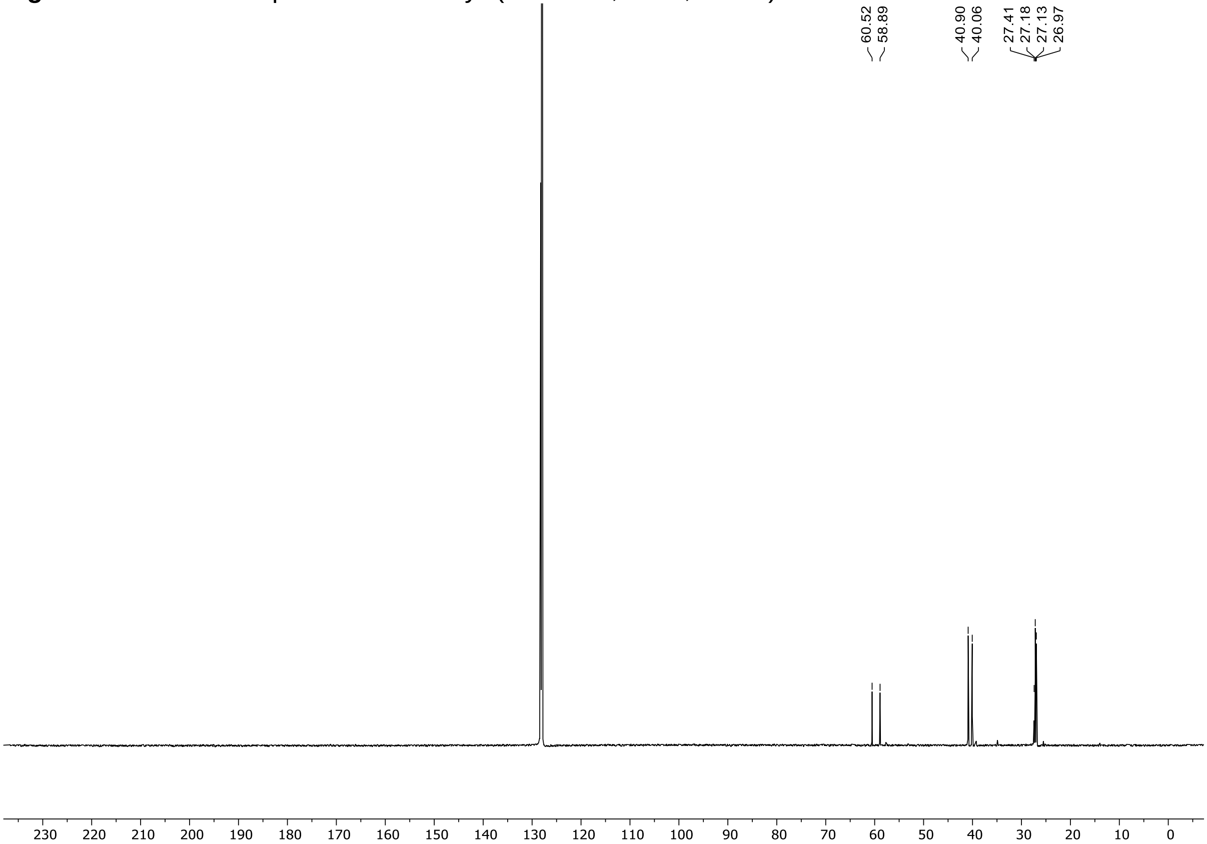

Figure S36. $^{13}C\{^1H\}$ NMR spectrum of $LiNCy_2$ (126 MHz, C_6D_6, 298 K).

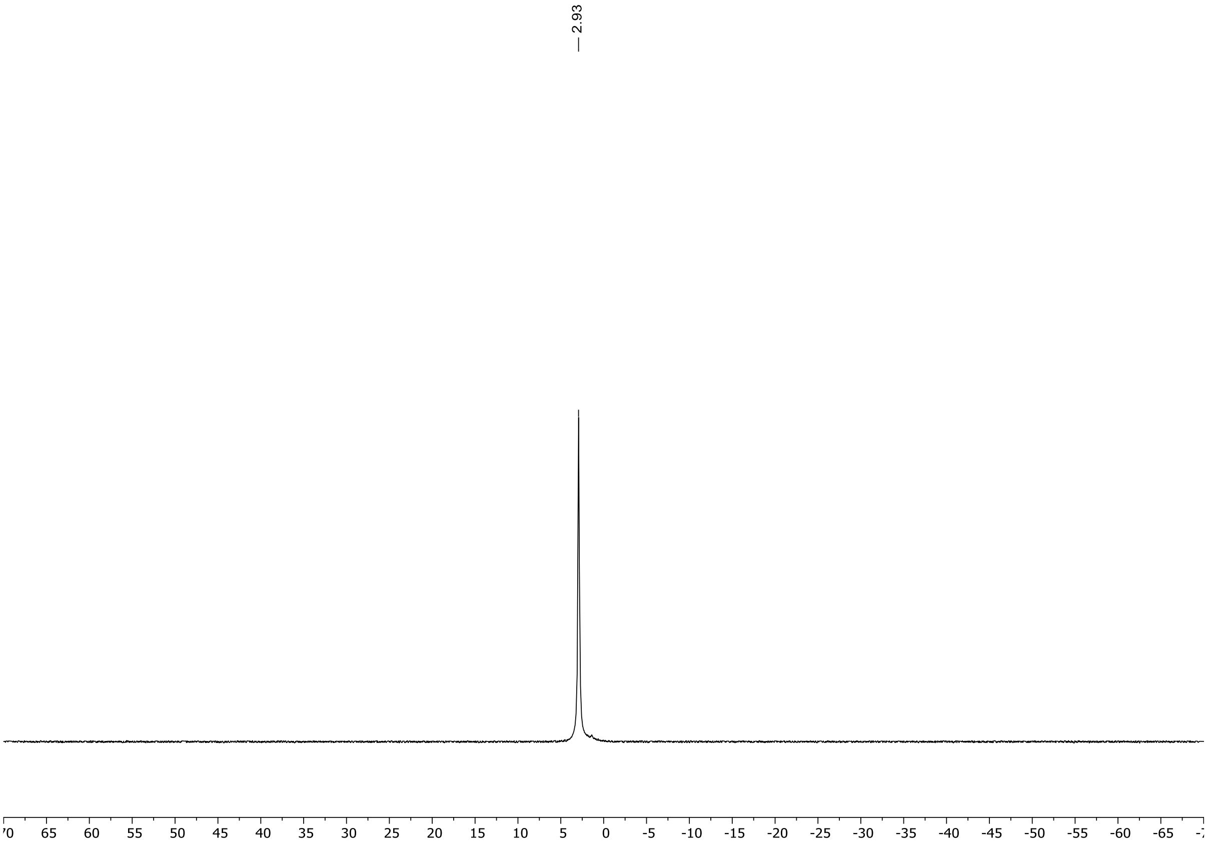

Figure S37. ^{7}Li NMR spectrum of $LiNCy_2$ (140 MHz, C_6D_6, 298 K).

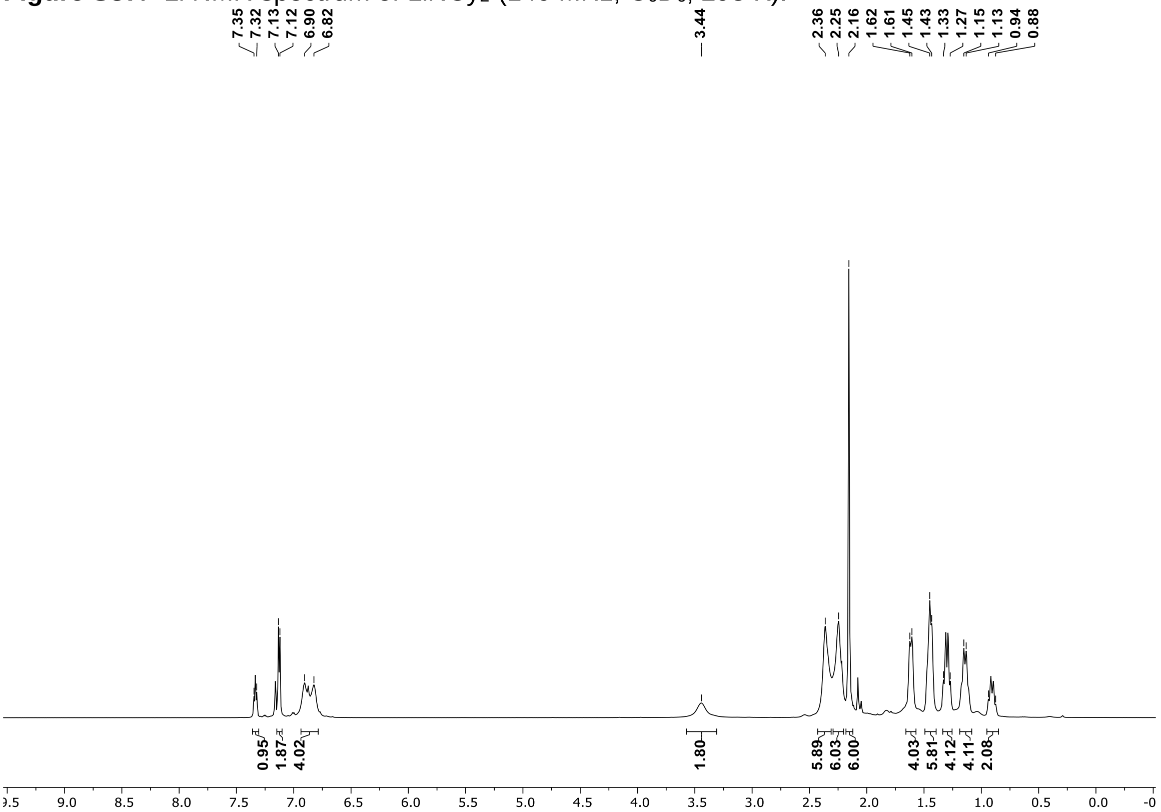

Figure S38. ^{1}H NMR spectrum of $^{Mes}TerSn\{NCy_2\}$ (**6**) (400 MHz, C_6D_6, 298 K).

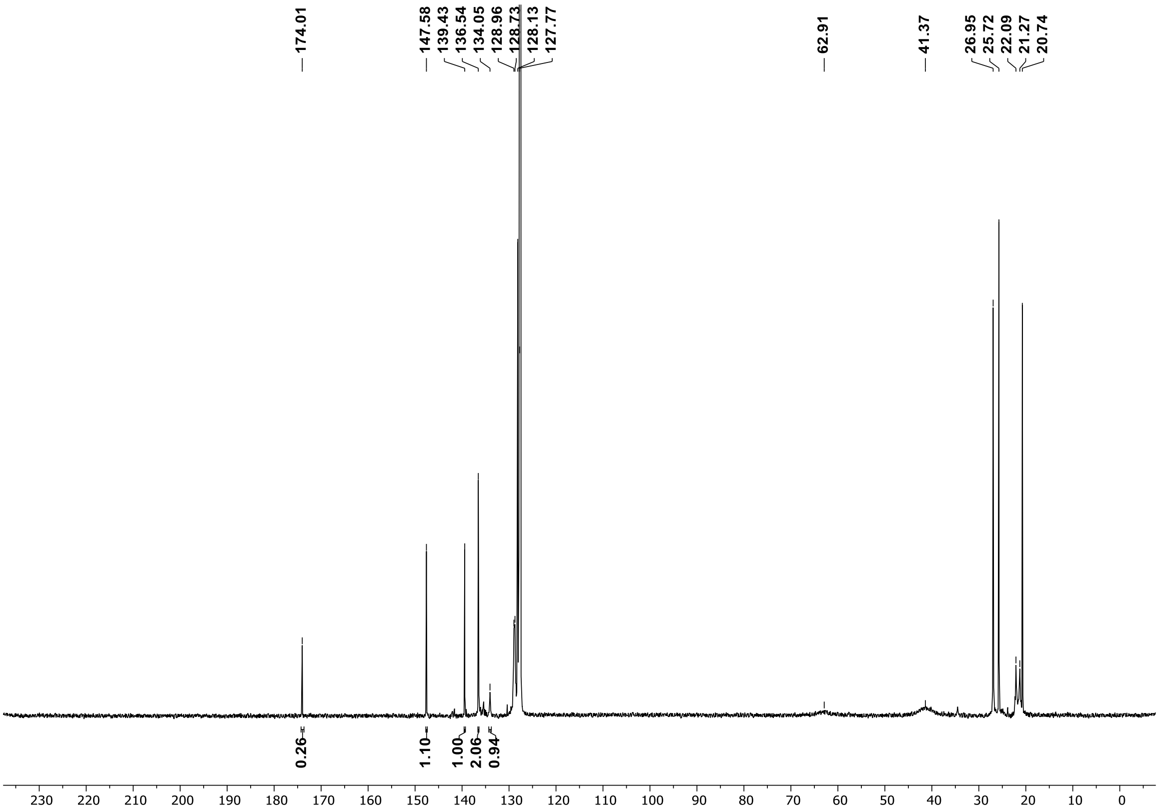

Figure S39. $^{13}C\{^1H\}$ NMR spectrum of $^{Mes}TerSn\{NCy_2\}$ (**6**) (126 MHz, C_6D_6, 298 K).

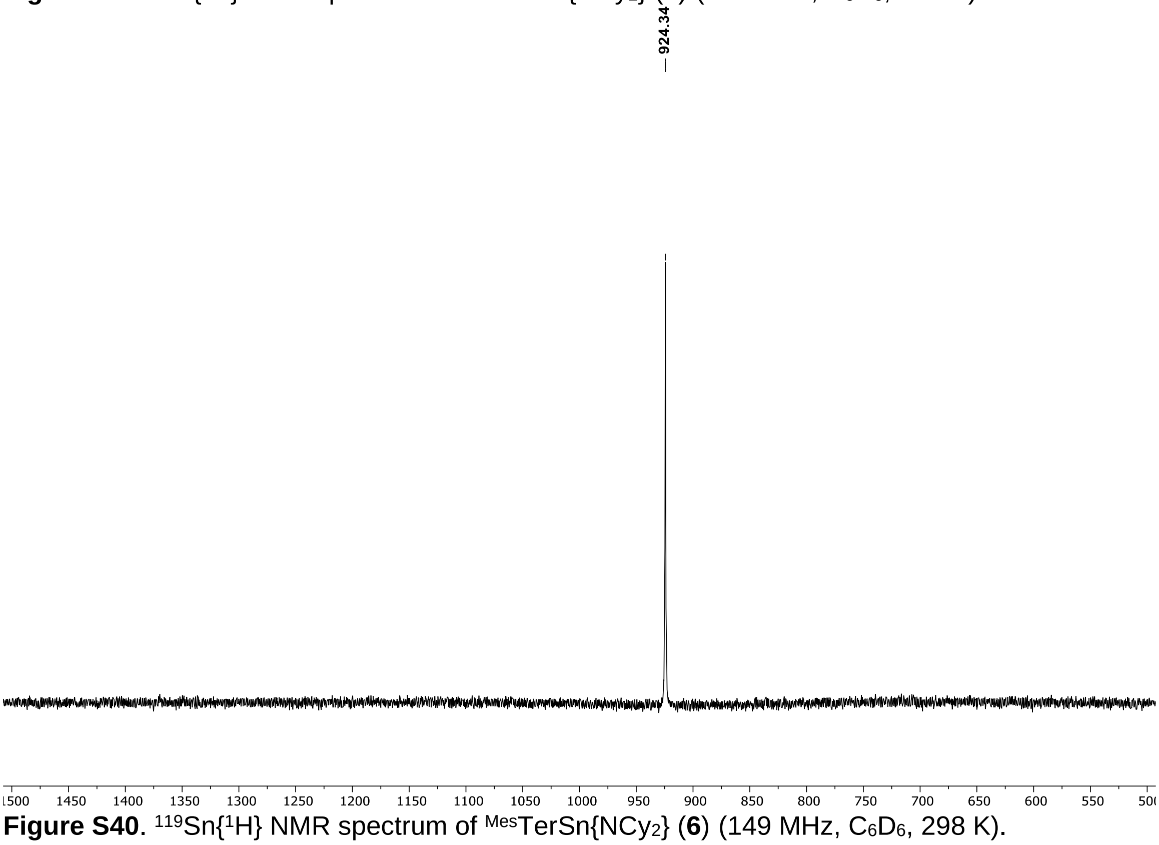

Figure S40. $^{119}Sn\{^1H\}$ NMR spectrum of $^{Mes}TerSn\{NCy_2\}$ (**6**) (149 MHz, C_6D_6, 298 K).

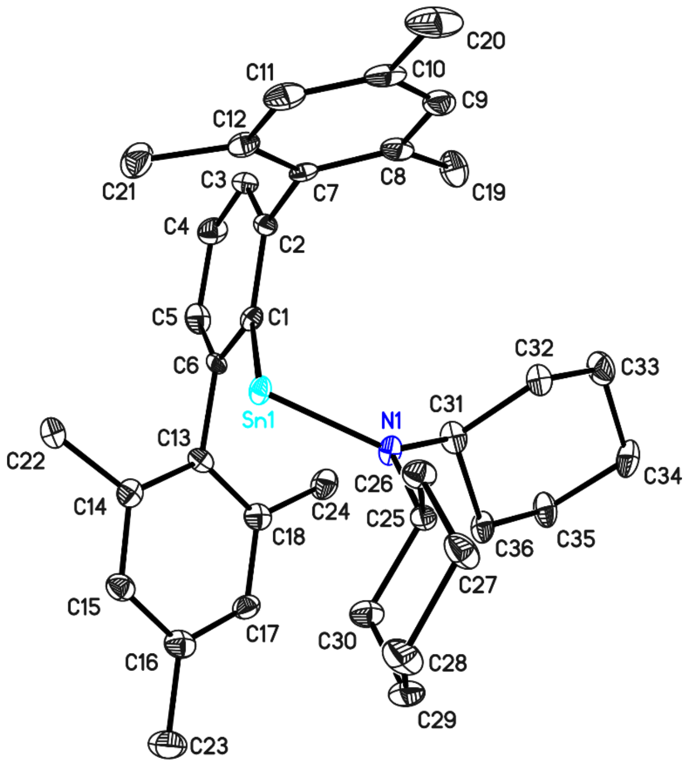

Figure S41. Molecular structure of ^{Mes}TerSnNCy$_2$ (**6**) in the crystal. Thermal ellipsoids are drawn at the 50% probability level (hydrogen atoms have been omitted for clarity).

The data were collected on a non-merohedral twin with the twin law 0 -1 0 -1 0 0 -0.20 0.20 -1. The fractional contribution of the minor component refined to 0.3413(11).

Table S4. Bond lengths [Å] and angles [°] for **6.**

Sn(1)-N(1)	2.026(4)	C(12)-C(14)	1.398(6)
Sn(1)-C(1)	2.221(4)	C(12)-C(18)	1.403(6)
N(1)-C(31)	1.461(6)	C(2)-C(3)	1.385(6)
N(1)-C(25)	1.478(5)	C(3)-C(4)	1.389(6)
C(1)-C(6)	1.397(6)	C(13)-C(21)	1.511(7)
C(1)-C(2)	1.405(6)	C(14)-C(15)	1.403(6)
C(5)-C(4)	1.387(6)	C(14)-C(22)	1.497(7)
C(5)-C(6)	1.401(6)	C(15)-C(16)	1.387(7)
C(6)-C(12)	1.511(6)	C(16)-C(17)	1.380(7)
C(7)-C(8)	1.400(7)	C(16)-C(23)	1.505(6)
C(7)-C(13)	1.405(6)	C(17)-C(18)	1.404(6)
C(7)-C(2)	1.501(6)	C(18)-C(24)	1.507(6)
C(8)-C(9)	1.399(6)	C(25)-C(26)	1.529(6)
C(8)-C(19)	1.513(7)	C(25)-C(30)	1.533(6)
C(9)-C(10)	1.393(8)	C(26)-C(27)	1.534(6)
C(10)-C(11)	1.382(7)	C(27)-C(28)	1.520(7)
C(10)-C(20)	1.507(7)	C(28)-C(29)	1.532(7)
C(11)-C(13)	1.403(6)	C(29)-C(30)	1.526(6)

C(31)-C(36)	1.541(6)
C(31)-C(32)	1.547(6)
C(32)-C(33)	1.523(6)
C(33)-C(34)	1.527(7)
C(34)-C(35)	1.534(7)
C(35)-C(36)	1.524(6)
N(1)-Sn(1)-C(1)	101.77(14)
C(31)-N(1)-C(25)	115.8(3)
C(31)-N(1)-Sn(1)	127.0(3)
C(25)-N(1)-Sn(1)	117.2(3)
C(6)-C(1)-C(2)	118.9(4)
C(6)-C(1)-Sn(1)	120.1(3)
C(2)-C(1)-Sn(1)	120.0(3)
C(4)-C(5)-C(6)	119.5(4)
C(1)-C(6)-C(5)	120.5(4)
C(1)-C(6)-C(12)	117.8(4)
C(5)-C(6)-C(12)	121.7(4)
C(8)-C(7)-C(13)	119.6(4)
C(8)-C(7)-C(2)	119.9(4)
C(13)-C(7)-C(2)	120.5(4)
C(9)-C(8)-C(7)	119.6(5)
C(9)-C(8)-C(19)	119.9(4)
C(7)-C(8)-C(19)	120.4(4)
C(10)-C(9)-C(8)	121.6(5)
C(11)-C(10)-C(9)	117.9(4)
C(11)-C(10)-C(20)	121.2(5)
C(9)-C(10)-C(20)	120.9(5)
C(10)-C(11)-C(13)	122.4(4)
C(14)-C(12)-C(18)	120.8(4)
C(14)-C(12)-C(6)	120.2(4)
C(18)-C(12)-C(6)	118.8(4)
C(3)-C(2)-C(1)	120.6(4)
C(3)-C(2)-C(7)	121.4(4)
C(1)-C(2)-C(7)	118.0(4)
C(2)-C(3)-C(4)	119.9(4)
C(5)-C(4)-C(3)	120.6(4)
C(11)-C(13)-C(7)	118.8(4)
C(11)-C(13)-C(21)	119.2(4)
C(7)-C(13)-C(21)	122.0(4)
C(12)-C(14)-C(15)	118.5(4)
C(12)-C(14)-C(22)	121.5(4)
C(15)-C(14)-C(22)	119.9(4)
C(16)-C(15)-C(14)	121.7(4)
C(17)-C(16)-C(15)	118.5(4)
C(17)-C(16)-C(23)	120.8(5)
C(15)-C(16)-C(23)	120.6(5)
C(16)-C(17)-C(18)	122.1(4)
C(12)-C(18)-C(17)	118.2(4)
C(12)-C(18)-C(24)	122.4(4)
C(17)-C(18)-C(24)	119.1(4)
N(1)-C(25)-C(26)	113.8(4)
N(1)-C(25)-C(30)	112.2(4)
C(26)-C(25)-C(30)	108.7(4)
C(25)-C(26)-C(27)	112.2(4)
C(28)-C(27)-C(26)	111.2(4)
C(27)-C(28)-C(29)	110.9(4)
C(30)-C(29)-C(28)	110.8(4)
C(29)-C(30)-C(25)	112.2(4)
N(1)-C(31)-C(36)	114.0(3)
N(1)-C(31)-C(32)	111.2(4)
C(36)-C(31)-C(32)	109.1(4)
C(33)-C(32)-C(31)	113.2(4)
C(32)-C(33)-C(34)	110.2(4)
C(33)-C(34)-C(35)	109.8(4)
C(36)-C(35)-C(34)	110.9(4)
C(35)-C(36)-C(31)	112.5(4)

Reaction of ^{Mes}TerSn{NCy$_2$} (6) and ^{i}PrN=C=N^{i}Pr – Synthesis of ^{Mes}TerSn{N(^{i}Pr)C(NCy$_2$)N(^{i}Pr)} (7)

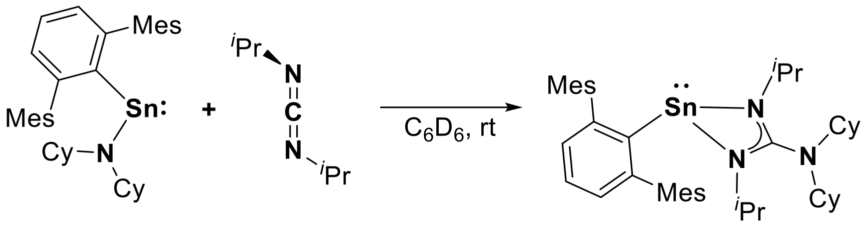

In a typical experiment ^{Mes}TerSnCl (**5**) (0.030 g, 0.064 mmol) and LiNCy$_2$ (0.012 g, 0.064 mmol) were suspended in 0.6 mL of C_6D_6 and the reaction progress was monitored by ^{1}H NMR spectroscopy until **5** was completely consumed and **6** was formed. ^{i}PrN=C=N^{i}Pr (0.008 g, 0.064 mmol) in 0.1 mL of C_6D_6 was added and the reaction was left untouched over night at room temperature leading to the clean formation of ^{Mes}Ter{N(^{i}Pr)C(NCy$_2$)N(^{i}Pr)} (**7**). All volatile components were removed under vacuum, the residue was suspended in 0.6 mL of *n*-hexane, filtered and stored at -30 °C to give **7** as a colourless crystalline material. Crystals obtained this way were suitable for single crystal X-ray diffraction.

Yield: 0.026 g (0.035 mmol; 55%).
^{1}H NMR (400 MHz, C_6D_6, 298 K): δ = 0.83 (d, $^3J_{H,H}$ = 6.4 Hz, 6H, CH(C*H*$_3$)$_2$), 0.98-1.01 (m(br), 2H, $CH_{2,Cy}$), 1.09 (d, $^3J_{H,H}$ = 6.4 Hz, 6H, CH(C*H*$_3$)$_2$), 1.15-1.29 (m(br), 8H, $CH_{2,Cy}$), 1.40-1.50 (m(br), 3H, $CH_{2,Cy}$), 1.63-1.85 (m(br), 7H, $CH_{2,Cy}$), 2.24 (s, 6H, CH_3), 2.38 (s, 12H, CH_3), 2.80 (m(br), 2H, CH_{Cy}), 3.77 (hept, $^3J_{H,H}$ = 6.4 Hz, 2H, C*H*(CH$_3$)$_2$), 6.92 (s, 4H, CH_{Aryl}), 7.00-7.01 (m, 2H, CH_{Aryl}), 7.25-7.27 (m, 1H, CH_{Aryl}) ppm.
^{13}C{^{1}H} NMR (126 MHz, C_6D_6, 298 K): δ = 21.2 (CH_3), 22.4 (CH_3), 24.8 (CH($\underline{C}$H$_3$)$_2$), 26.4 (br, $CH_{2,Cy}$), 26.8 (br, $CH_{2,Cy}$), 28.2 (CH($\underline{C}$H$_3$)$_2$), 33.9 ($CH_{2,Cy}$), 46.6 ($\underline{C}$H(CH$_3$)$_2$), 58.2 (br, CH_{Cy}), 59.1 (br, CH_{Cy}), 127.9 (CH_{Aryl})*, 128.9 (CH_{Aryl}), 129.2 (CH_{Aryl}), 136.1 ($C_{q,Aryl}$), 136.5 ($C_{q,Aryl}$), 142.3 ($C_{q,Aryl}$), 148.8 ($C_{q,Aryl}$), 162.1 (C_q(N^{i}Pr)$_2$NCy$_2$), 172.4 (C_qSn)
* = overlap with C_6D_5H signal
^{119}Sn{^{1}H} NMR (149 MHz, C_6D_6, 298 K): δ = 103.8 ppm.
MS (LIFDI): m/z calcd. for $C_{43}H_{61}N_3Sn$: 739.3887; found: 739.3.
EA: Anal. calcd. for $C_{43}H_{61}N_3Sn$: C, 69.92; H, 8.32; N, 5.69; Found: C, 70.15; H, 8.57; N, 5.50.

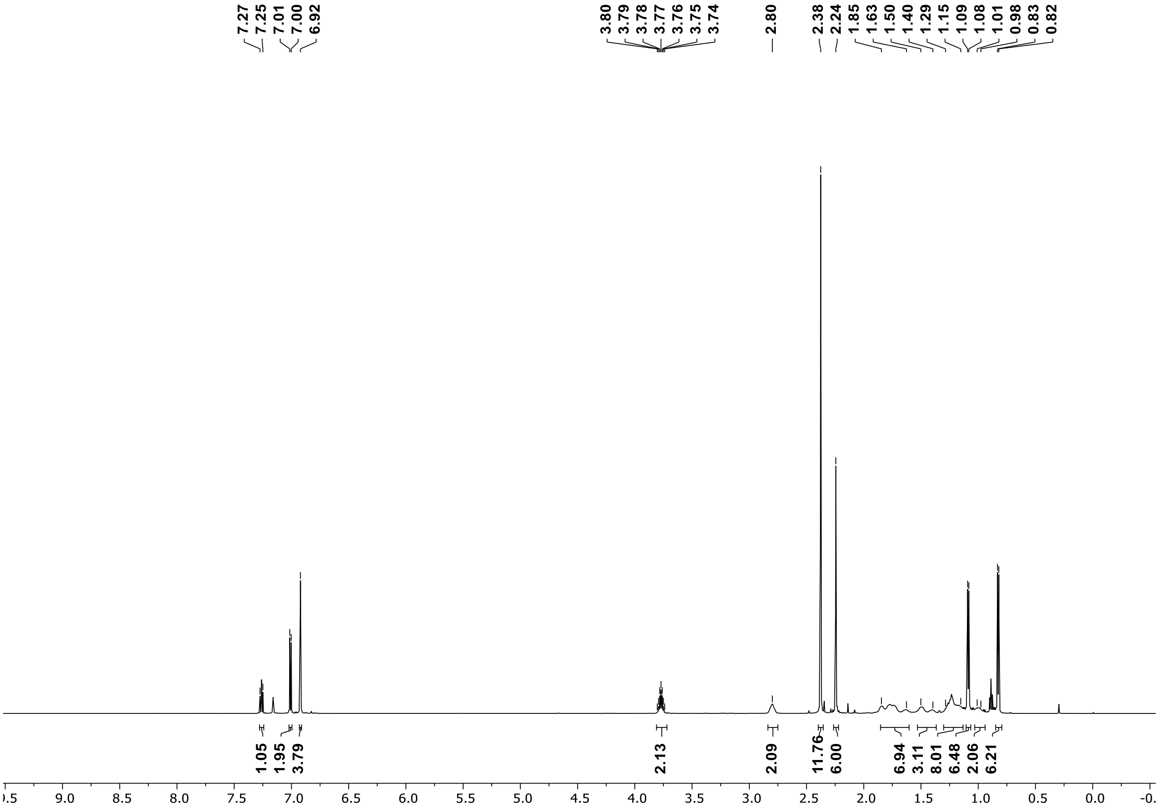

Figure S42. ^{1}H NMR spectrum of ^{Mes}TerSn{N(^{i}Pr)N(Cy$_2$)C(^{i}Pr)} (**7**) (400 MHz, C_6D_6, 298 K).

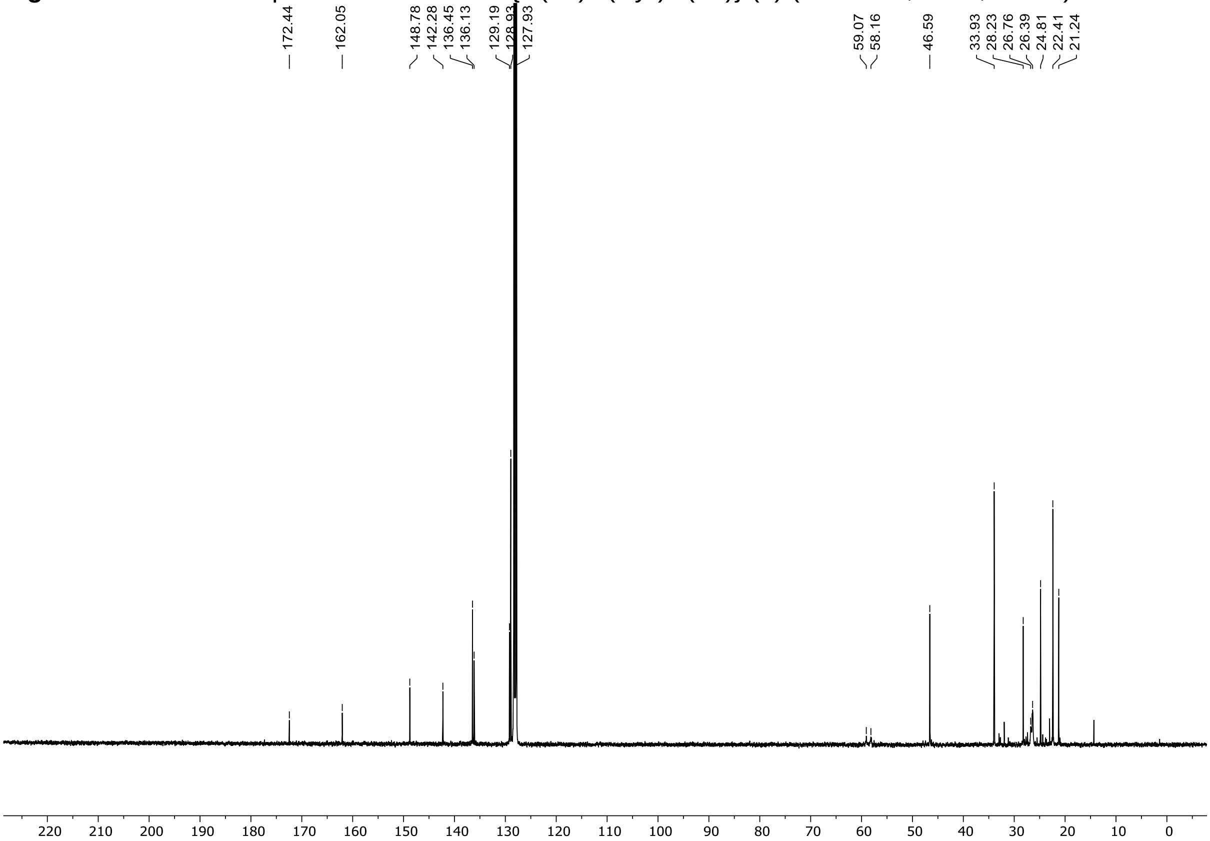

Figure S43. ^{13}C{^{1}H} NMR spectrum of ^{Mes}TerSn{N(^{i}Pr)N(Cy$_2$)C(^{i}Pr)} (**7**) (126 MHz, C_6D_6, 298 K).

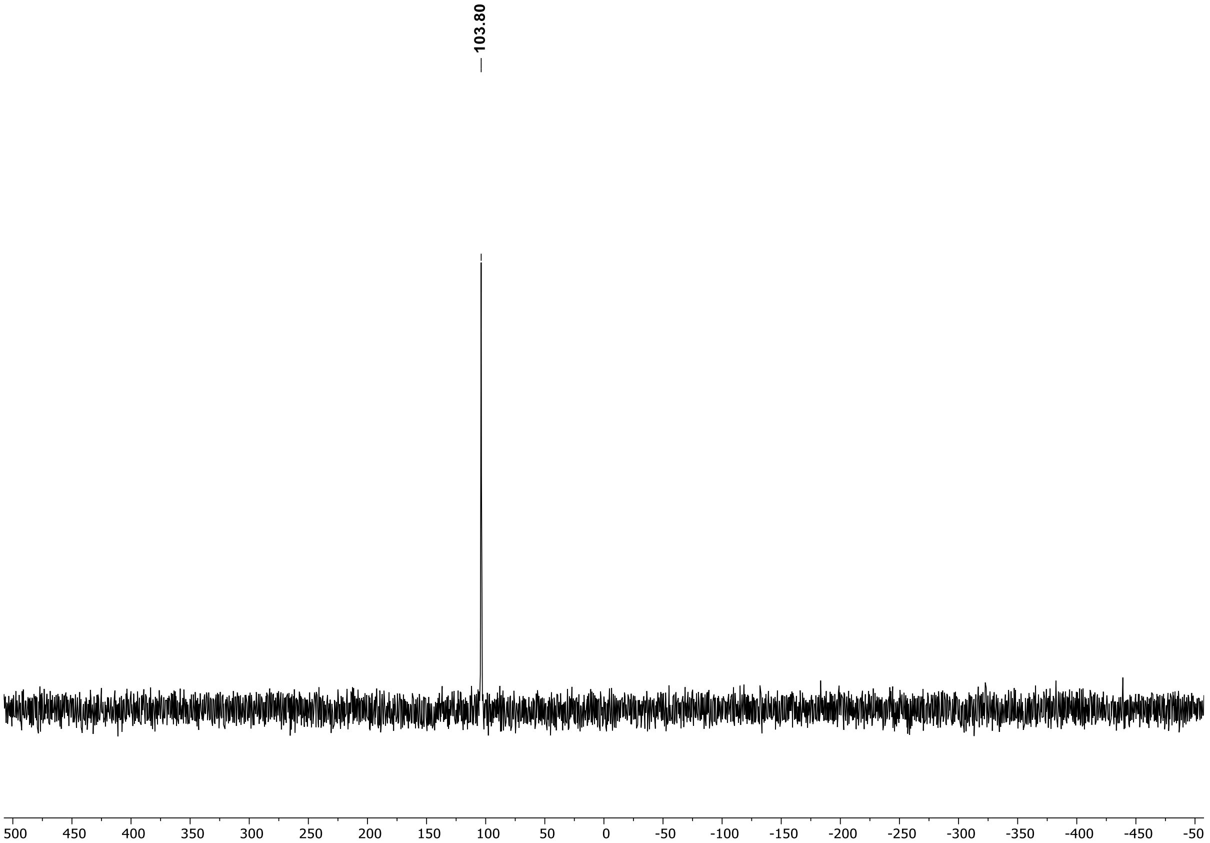

Figure S44. $^{119}Sn\{^{1}H\}$ NMR spectrum of ^{Mes}TerSn{N(^{i}Pr)N(Cy_2)C(^{i}Pr)} (**7**) (149 MHz, C_6D_6, 298 K).

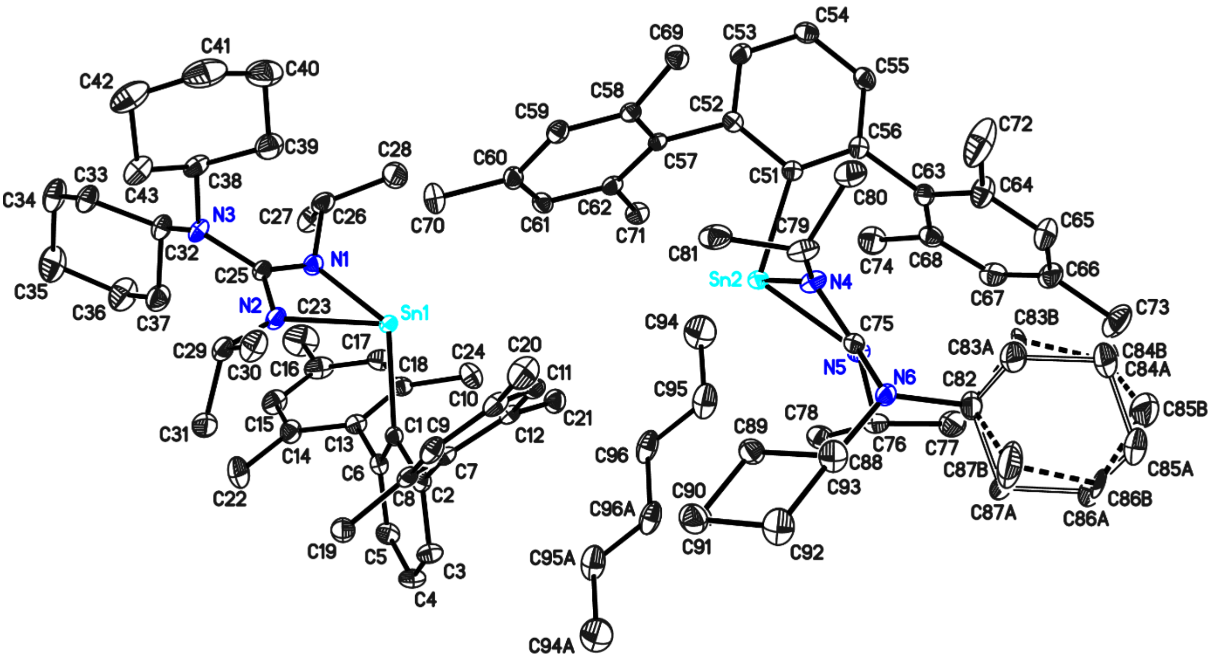

Figure S45. Molecular structure of ^{Mes}TerSn{N(^{i}Pr)N(Cy_2)C(^{i}Pr)} (**7**) in the crystal. Thermal ellipsoids are drawn at the 50% probability level (hydrogen atoms have been omitted for clarity).

Two cyclohexyl groups are disordered about two positions. The disorder was treated with distance restraints and restraints for the anisotropic displacement parameters. The occupancies of the minor component refined to 0.221(3) and 0.063(2), respectively.

Table S5. Bond lengths [Å] and angles [°] for **7.**

Sn(1)-N(2)	2.1871(13)	C(7)-C(12)	1.4135(19)
Sn(1)-C(1)	2.2420(15)	C(8)-C(9)	1.396(2)
Sn(1)-N(1)	2.2654(12)	C(8)-C(19)	1.510(2)
Sn(1)-C(25)	2.6478(15)	C(9)-C(10)	1.389(2)
N(1)-C(25)	1.3377(19)	C(10)-C(11)	1.390(2)
N(1)-C(26)	1.4705(19)	C(10)-C(20)	1.506(2)
N(2)-C(25)	1.3250(18)	C(11)-C(12)	1.390(2)
N(2)-C(29)	1.4577(19)	C(12)-C(21)	1.504(2)
N(3)-C(25)	1.4179(18)	C(13)-C(14)	1.403(2)
N(3)-C(32)	1.4787(18)	C(13)-C(18)	1.405(2)
N(3)-C(38)	1.487(2)	C(14)-C(15)	1.397(2)
C(1)-C(6)	1.4092(19)	C(14)-C(22)	1.507(2)
C(1)-C(2)	1.4149(18)	C(15)-C(16)	1.385(3)
C(2)-C(3)	1.394(2)	C(16)-C(17)	1.391(2)
C(2)-C(7)	1.5008(19)	C(16)-C(23)	1.509(2)
C(3)-C(4)	1.381(2)	C(17)-C(18)	1.396(2)
C(4)-C(5)	1.387(2)	C(18)-C(24)	1.504(2)
C(5)-C(6)	1.400(2)	C(26)-C(28)	1.526(2)
C(6)-C(13)	1.4965(19)	C(26)-C(27)	1.526(2)
C(7)-C(8)	1.4099(19)	C(29)-C(31)	1.524(2)

C(29)-C(30)	1.527(2)
C(32)-C(33)	1.529(2)
C(32)-C(37)	1.531(2)
C(33)-C(34)	1.528(2)
C(34)-C(35)	1.518(3)
C(35)-C(36)	1.524(3)
C(36)-C(37)	1.535(2)
C(38)-C(43)	1.531(2)
C(38)-C(39)	1.531(2)
C(39)-C(40)	1.530(2)
C(40)-C(41)	1.520(3)
C(41)-C(42)	1.524(3)
C(42)-C(43)	1.527(2)
Sn(2)-N(4)	2.2012(13)
Sn(2)-C(51)	2.2369(14)
Sn(2)-N(5)	2.2480(13)
Sn(2)-C(75)	2.6288(14)
N(4)-C(75)	1.3309(18)
N(4)-C(79)	1.4608(19)
N(5)-C(75)	1.3425(18)
N(5)-C(76)	1.4743(18)
C(51)-C(56)	1.4125(19)
C(51)-C(52)	1.4160(19)
C(52)-C(53)	1.3955(19)
C(52)-C(57)	1.5054(19)
C(53)-C(54)	1.384(2)
C(54)-C(55)	1.389(2)
C(55)-C(56)	1.398(2)
C(56)-C(63)	1.4994(19)
C(57)-C(58)	1.4047(19)
C(57)-C(62)	1.4081(19)
C(58)-C(59)	1.3975(19)
C(58)-C(69)	1.505(2)
C(59)-C(60)	1.387(2)
C(60)-C(61)	1.391(2)
C(60)-C(70)	1.510(2)
C(61)-C(62)	1.395(2)
C(62)-C(71)	1.508(2)
C(63)-C(64)	1.398(2)
C(63)-C(68)	1.402(2)
C(64)-C(65)	1.398(2)
C(64)-C(72)	1.503(2)
C(65)-C(66)	1.383(2)
C(66)-C(67)	1.384(2)
C(66)-C(73)	1.513(2)
C(67)-C(68)	1.396(2)
C(68)-C(74)	1.507(2)
C(75)-N(6)	1.4091(17)
C(76)-C(78)	1.523(2)
C(76)-C(77)	1.524(2)
C(79)-C(81)	1.524(2)
C(79)-C(80)	1.526(2)
N(6)-C(82)	1.4681(18)
N(6)-C(88)	1.4812(18)
C(82)-C(87B)	1.445(6)
C(82)-C(83A)	1.496(2)
C(82)-C(83B)	1.509(7)
C(82)-C(87A)	1.534(3)
C(83A)-C(84A)	1.519(3)
C(84A)-C(85A)	1.507(4)
C(85A)-C(86A)	1.528(3)
C(86A)-C(87A)	1.535(3)
C(83B)-C(84B)	1.534(9)
C(84B)-C(85B)	1.504(9)
C(85B)-C(86B)	1.426(10)
C(86B)-C(87B)	1.552(9)
C(88)-C(89B)	1.388(12)
C(88)-C(89A)	1.525(2)
C(88)-C(93A)	1.535(2)
C(88)-C(93B)	1.537(13)
C(89A)-C(90A)	1.528(2)
C(90A)-C(91A)	1.523(2)
C(91A)-C(92A)	1.525(2)
C(92A)-C(93A)	1.535(3)
C(89B)-C(90B)	1.534(13)
C(90B)-C(91B)	1.503(13)

C(91B)-C(92B)	1.511(13)	C(7)-C(8)-C(19)	122.46(13)
C(92B)-C(93B)	1.528(14)	C(10)-C(9)-C(8)	121.96(13)
C(94)-C(95)	1.523(3)	C(9)-C(10)-C(11)	118.27(13)
C(95)-C(96)	1.512(3)	C(9)-C(10)-C(20)	120.90(14)
C(96)-C(96)#1	1.522(3)	C(11)-C(10)-C(20)	120.82(15)
		C(10)-C(11)-C(12)	121.72(14)
N(2)-Sn(1)-C(1)	99.20(5)	C(11)-C(12)-C(7)	119.55(13)
N(2)-Sn(1)-N(1)	59.56(5)	C(11)-C(12)-C(21)	119.11(13)
C(1)-Sn(1)-N(1)	111.83(5)	C(7)-C(12)-C(21)	121.25(13)
N(2)-Sn(1)-C(25)	29.92(4)	C(14)-C(13)-C(18)	119.25(13)
C(1)-Sn(1)-C(25)	112.46(5)	C(14)-C(13)-C(6)	119.98(13)
N(1)-Sn(1)-C(25)	30.34(4)	C(18)-C(13)-C(6)	120.33(13)
C(25)-N(1)-C(26)	122.45(12)	C(15)-C(14)-C(13)	119.47(15)
C(25)-N(1)-Sn(1)	90.85(9)	C(15)-C(14)-C(22)	119.50(14)
C(26)-N(1)-Sn(1)	130.98(10)	C(13)-C(14)-C(22)	121.03(14)
C(25)-N(2)-C(29)	125.34(12)	C(16)-C(15)-C(14)	121.85(15)
C(25)-N(2)-Sn(1)	94.67(9)	C(15)-C(16)-C(17)	118.19(14)
C(29)-N(2)-Sn(1)	139.94(9)	C(15)-C(16)-C(23)	120.90(16)
C(25)-N(3)-C(32)	116.36(12)	C(17)-C(16)-C(23)	120.90(17)
C(25)-N(3)-C(38)	116.78(12)	C(16)-C(17)-C(18)	121.61(15)
C(32)-N(3)-C(38)	117.24(12)	C(17)-C(18)-C(13)	119.57(14)
C(6)-C(1)-C(2)	117.51(12)	C(17)-C(18)-C(24)	119.42(14)
C(6)-C(1)-Sn(1)	129.42(10)	C(13)-C(18)-C(24)	121.01(13)
C(2)-C(1)-Sn(1)	112.95(10)	N(2)-C(25)-N(1)	112.39(12)
C(3)-C(2)-C(1)	121.15(13)	N(2)-C(25)-N(3)	121.13(13)
C(3)-C(2)-C(7)	116.85(12)	N(1)-C(25)-N(3)	126.46(13)
C(1)-C(2)-C(7)	122.00(12)	N(2)-C(25)-Sn(1)	55.41(7)
C(4)-C(3)-C(2)	120.43(13)	N(1)-C(25)-Sn(1)	58.81(7)
C(3)-C(4)-C(5)	119.55(14)	N(3)-C(25)-Sn(1)	165.41(10)
C(4)-C(5)-C(6)	120.96(13)	N(1)-C(26)-C(28)	110.15(13)
C(5)-C(6)-C(1)	120.33(13)	N(1)-C(26)-C(27)	111.22(14)
C(5)-C(6)-C(13)	115.60(12)	C(28)-C(26)-C(27)	109.58(14)
C(1)-C(6)-C(13)	124.05(13)	N(2)-C(29)-C(31)	109.48(13)
C(8)-C(7)-C(12)	119.08(13)	N(2)-C(29)-C(30)	108.96(13)
C(8)-C(7)-C(2)	120.68(12)	C(31)-C(29)-C(30)	112.68(13)
C(12)-C(7)-C(2)	120.03(12)	N(3)-C(32)-C(33)	111.96(12)
C(9)-C(8)-C(7)	119.17(13)	N(3)-C(32)-C(37)	110.04(12)
C(9)-C(8)-C(19)	118.32(13)	C(33)-C(32)-C(37)	108.76(13)

C(34)-C(33)-C(32)	109.99(13)	C(58)-C(57)-C(52)	119.98(12)
C(35)-C(34)-C(33)	111.32(14)	C(62)-C(57)-C(52)	120.58(12)
C(34)-C(35)-C(36)	110.45(16)	C(59)-C(58)-C(57)	119.41(13)
C(35)-C(36)-C(37)	111.55(16)	C(59)-C(58)-C(69)	119.22(13)
C(32)-C(37)-C(36)	111.07(14)	C(57)-C(58)-C(69)	121.35(12)
N(3)-C(38)-C(43)	112.20(13)	C(60)-C(59)-C(58)	121.84(13)
N(3)-C(38)-C(39)	114.05(12)	C(59)-C(60)-C(61)	118.08(13)
C(43)-C(38)-C(39)	109.15(14)	C(59)-C(60)-C(70)	121.15(14)
C(40)-C(39)-C(38)	110.52(14)	C(61)-C(60)-C(70)	120.76(13)
C(41)-C(40)-C(39)	111.92(17)	C(60)-C(61)-C(62)	121.93(13)
C(40)-C(41)-C(42)	111.34(16)	C(61)-C(62)-C(57)	119.30(13)
C(41)-C(42)-C(43)	110.84(15)	C(61)-C(62)-C(71)	119.13(13)
C(42)-C(43)-C(38)	111.35(15)	C(57)-C(62)-C(71)	121.57(13)
N(4)-Sn(2)-C(51)	102.06(5)	C(64)-C(63)-C(68)	119.16(13)
N(4)-Sn(2)-N(5)	59.81(5)	C(64)-C(63)-C(56)	120.94(13)
C(51)-Sn(2)-N(5)	110.48(5)	C(68)-C(63)-C(56)	119.65(13)
N(4)-Sn(2)-C(75)	30.37(4)	C(65)-C(64)-C(63)	119.63(15)
C(51)-Sn(2)-C(75)	115.21(5)	C(65)-C(64)-C(72)	119.52(15)
N(5)-Sn(2)-C(75)	30.71(4)	C(63)-C(64)-C(72)	120.84(14)
C(75)-N(4)-C(79)	124.58(12)	C(66)-C(65)-C(64)	121.74(16)
C(75)-N(4)-Sn(2)	92.88(9)	C(65)-C(66)-C(67)	118.07(14)
C(79)-N(4)-Sn(2)	140.33(10)	C(65)-C(66)-C(73)	120.57(16)
C(75)-N(5)-C(76)	122.10(12)	C(67)-C(66)-C(73)	121.33(16)
C(75)-N(5)-Sn(2)	90.52(9)	C(66)-C(67)-C(68)	121.92(15)
C(76)-N(5)-Sn(2)	129.20(9)	C(67)-C(68)-C(63)	119.43(14)
C(56)-C(51)-C(52)	117.31(12)	C(67)-C(68)-C(74)	119.74(14)
C(56)-C(51)-Sn(2)	131.00(10)	C(63)-C(68)-C(74)	120.82(14)
C(52)-C(51)-Sn(2)	111.49(9)	N(4)-C(75)-N(5)	112.18(12)
C(53)-C(52)-C(51)	121.45(13)	N(4)-C(75)-N(6)	122.54(13)
C(53)-C(52)-C(57)	116.86(12)	N(5)-C(75)-N(6)	125.22(13)
C(51)-C(52)-C(57)	121.69(12)	N(4)-C(75)-Sn(2)	56.75(7)
C(54)-C(53)-C(52)	120.23(13)	N(5)-C(75)-Sn(2)	58.77(7)
C(53)-C(54)-C(55)	119.46(13)	N(6)-C(75)-Sn(2)	160.49(10)
C(54)-C(55)-C(56)	121.18(13)	N(5)-C(76)-C(78)	109.24(12)
C(55)-C(56)-C(51)	120.33(13)	N(5)-C(76)-C(77)	112.43(12)
C(55)-C(56)-C(63)	116.07(12)	C(78)-C(76)-C(77)	108.20(13)
C(51)-C(56)-C(63)	123.54(12)	N(4)-C(79)-C(81)	109.77(13)
C(58)-C(57)-C(62)	119.35(12)	N(4)-C(79)-C(80)	110.91(13)

C(81)-C(79)-C(80)	110.85(13)	C(82)-C(87B)-C(86B)	114.3(5)
C(75)-N(6)-C(82)	116.12(12)	C(89B)-C(88)-N(6)	132.5(6)
C(75)-N(6)-C(88)	118.94(11)	N(6)-C(88)-C(89A)	112.38(11)
C(82)-N(6)-C(88)	120.89(12)	N(6)-C(88)-C(93A)	113.20(14)
C(87B)-C(82)-N(6)	118.6(3)	C(89A)-C(88)-C(93A)	110.70(14)
N(6)-C(82)-C(83A)	116.78(13)	C(89B)-C(88)-C(93B)	120.9(12)
C(87B)-C(82)-C(83B)	113.9(4)	N(6)-C(88)-C(93B)	104.7(8)
N(6)-C(82)-C(83B)	117.0(3)	C(88)-C(89A)-C(90A)	111.75(12)
N(6)-C(82)-C(87A)	109.61(14)	C(91A)-C(90A)-C(89A)	111.69(13)
C(83A)-C(82)-C(87A)	111.20(15)	C(90A)-C(91A)-C(92A)	110.31(13)
C(82)-C(83A)-C(84A)	112.01(16)	C(91A)-C(92A)-C(93A)	110.56(16)
C(85A)-C(84A)-C(83A)	111.8(2)	C(88)-C(93A)-C(92A)	110.92(17)
C(84A)-C(85A)-C(86A)	111.0(2)	C(88)-C(89B)-C(90B)	111.7(12)
C(85A)-C(86A)-C(87A)	110.38(19)	C(91B)-C(90B)-C(89B)	113.8(13)
C(82)-C(87A)-C(86A)	110.77(18)	C(90B)-C(91B)-C(92B)	114.3(14)
C(82)-C(83B)-C(84B)	114.2(6)	C(91B)-C(92B)-C(93B)	107.3(14)
C(85B)-C(84B)-C(83B)	112.7(6)	C(92B)-C(93B)-C(88)	115.9(13)
C(86B)-C(85B)-C(84B)	116.0(8)	C(96)-C(95)-C(94)	112.72(16)
C(85B)-C(86B)-C(87B)	113.8(8)	C(95)-C(96)-C(96)#1	113.84(17)

Symmetry transformations used to generate equivalent atoms:

#1 -x,-y+2,-z+1

Reaction of ^{Mes}TerSn{N(^{i}Pr)C(NCy$_2$)N(^{i}Pr)} (7) with Se – Formation of ^{Mes}TerSn(Se$_4$){N(^{i}Pr)C(NCy$_2$)N(^{i}Pr)} (8) and ^{Mes}TerSn(NCy$_2$)(μ-Se$_2$)Sn{N(^{i}Pr)C(NCy$_2$)N(^{i}Pr)}^{Mes}Ter (9)

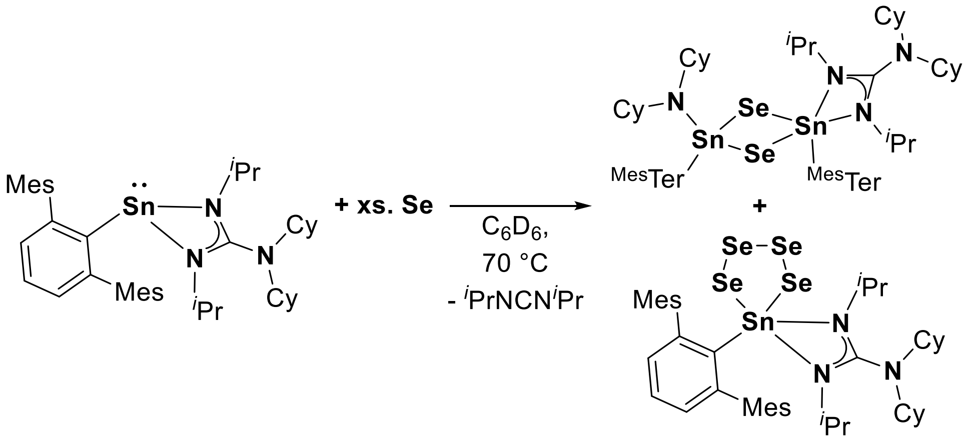

^{Mes}TerSn{N(^{i}Pr)C(NCy$_2$)N(^{i}Pr)} (**7**) (0.030 g, 0.041 mmol) and elemental Se (0.003 g, 0.041 mmol) were suspended in 0.5 mL of C_6D_6. Storage of the suspension for 16 h at room temperature did not lead to any consumption of the starting materials as verified by ^{1}H NMR spectroscopy. Heating of the reaction mixture to 70 °C lead to full consumption of the elemental Se but significant amounts of **7** remained unreacted. By adding an excess of elemental Se (0.015 g, 0.190 mmol) either to the above-mentioned reaction mixture or starting the reaction from **7** and an excess of elemental Se, **7** is consumed completely over the course of 4 h at 70 °C. It was found that the outcome of the reaction is independent of the amount of elemental Se used. The ^{1}H NMR spectrum shown in Figure S47 is therefore representative of this reaction. It was evident that ^{i}PrN=C=N^{i}Pr is formed during the reaction by its characteristic ^{1}H NMR chemical shifts. Removal of all volatile components from the reaction mixture, suspending the remaining yellowish solid in 0.6 mL of *n*-hexane and subsequent storage at -30 °C leads to co-crystallisation of an orange crystalline material and small amounts of a clear yellow crystalline material which were both suitable for single crystal X-ray diffraction (representative picture of the crystalline material obtained is shown in Figure S46, left). The orange crystalline material was found to be the tetraselenastannolane ^{Mes}TerSn(Se$_4$){N(^{i}Pr)C(NCy$_2$)N(^{i}Pr)} (**8**). Small amounts of **8** could be seperated (Figure S46, right) and were further analyzed by elemental micronanalysis, ^{1}H and ^{119}Sn NMR spectroscopy. The yellow crystalline material was found to be the selenium bridged compound ^{Mes}TerSn(NCy$_2$)(μ-Se$_2$)Sn{N(^{i}Pr)C(NCy$_2$)N(^{i}Pr)}^{Mes}Ter (**9**).

Selected analytical data of **8**:
^{1}H NMR (400 MHz, C_6D_6, 298 K): δ = 0.94-1.04 (m, 6H, $CH_{2,Cy}$), 1.20-1.37 (m, 16H, CH(C*H*$_3$)$_2$, $CH_{2,Cy}$)*, 1.54-1.62 (m, 4H, $CH_{2,Cy}$), 1.73-1.77 (m, 6H, $CH_{2,Cy}$), 2.24 (s, 12H, CH_3), 2.37-2.46 (m, 6H, CH_3), 2.71-2.89 (m, 2H, CH_{Cy}), 3.69 (m(br), 2H, C*H*(CH$_3$)$_2$, 6.72-6.89 (m, 4H, CH_{Aryl}), 6.96 (m, 2H, CH_{Aryl}), 7.09-7.14 (m, 1H, CH_{Aryl}) ppm.
* = overlap with *n*-hexane signals
^{119}Sn{^{1}H} NMR (149 MHz, C_6D_6, 298 K): δ = -252.9 ppm.
EA: Anal. calcd. for $C_{43}H_{61}N_3Se_4Sn$: C, 48.97; H, 5.83; N, 3.98; Found: C, 48.57; H, 5.76; N, 3.70.

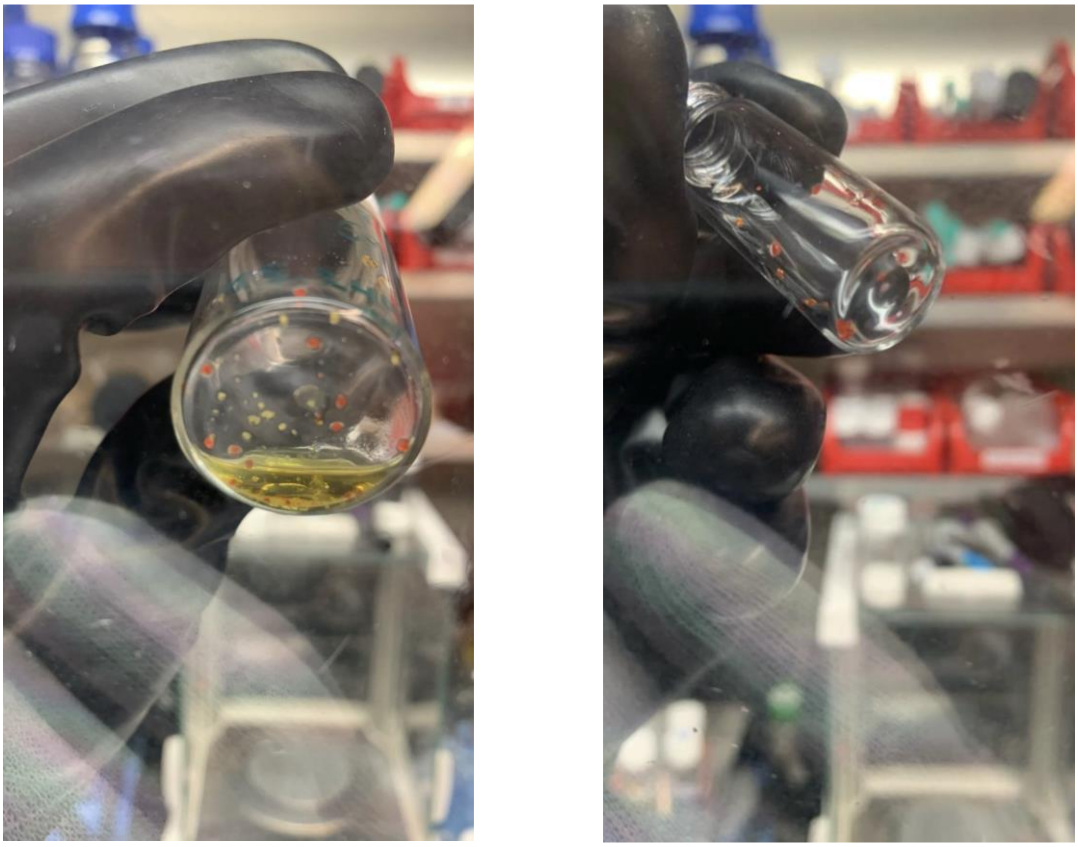

Figure S46. Photo of the obtained crystalline material containing **8** and **9** (left) and separated crystalline material of **8** (right).

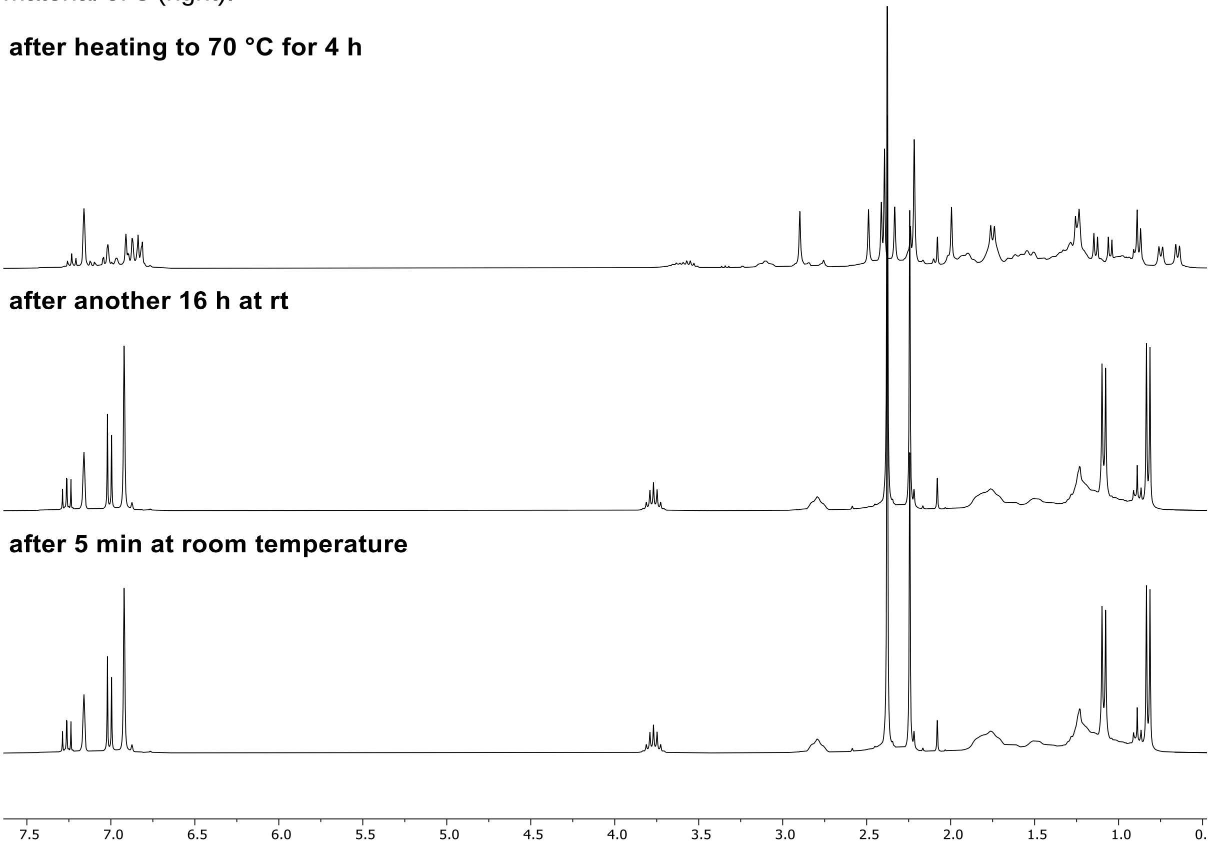

Figure S47. Monitoring of the reaction of ^{Mes}TerSn{N(^{i}Pr)C(NCy$_2$)N(^{i}Pr)} (**7**) with elemental selenium via 1H NMR spectroscopy (400 MHz, C_6D_6, 298 K).

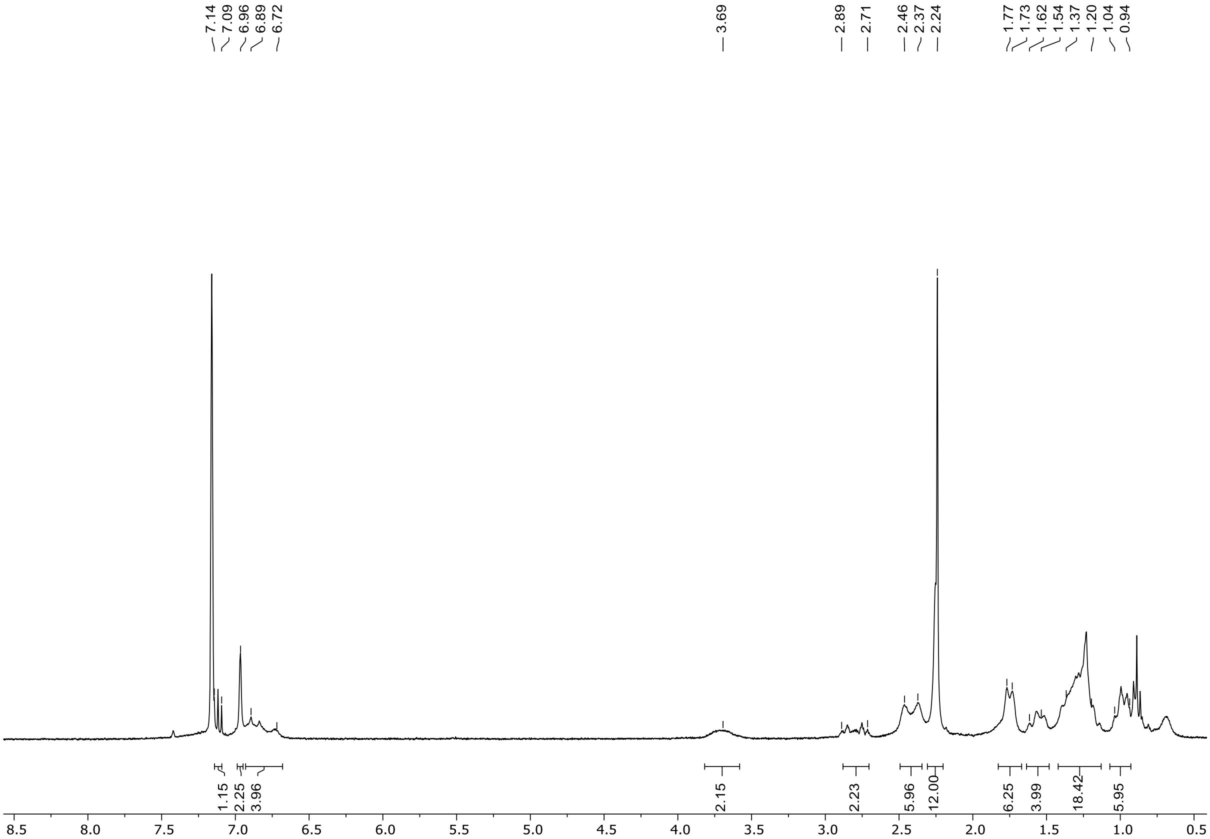

Figure S48. ^{1}H NMR spectrum of ^{Mes}TerSn(Se$_4$){N(^{i}Pr)C(NCy$_2$)N(^{i}Pr)} (**8**) (400 MHz, C_6D_6, 298 K).

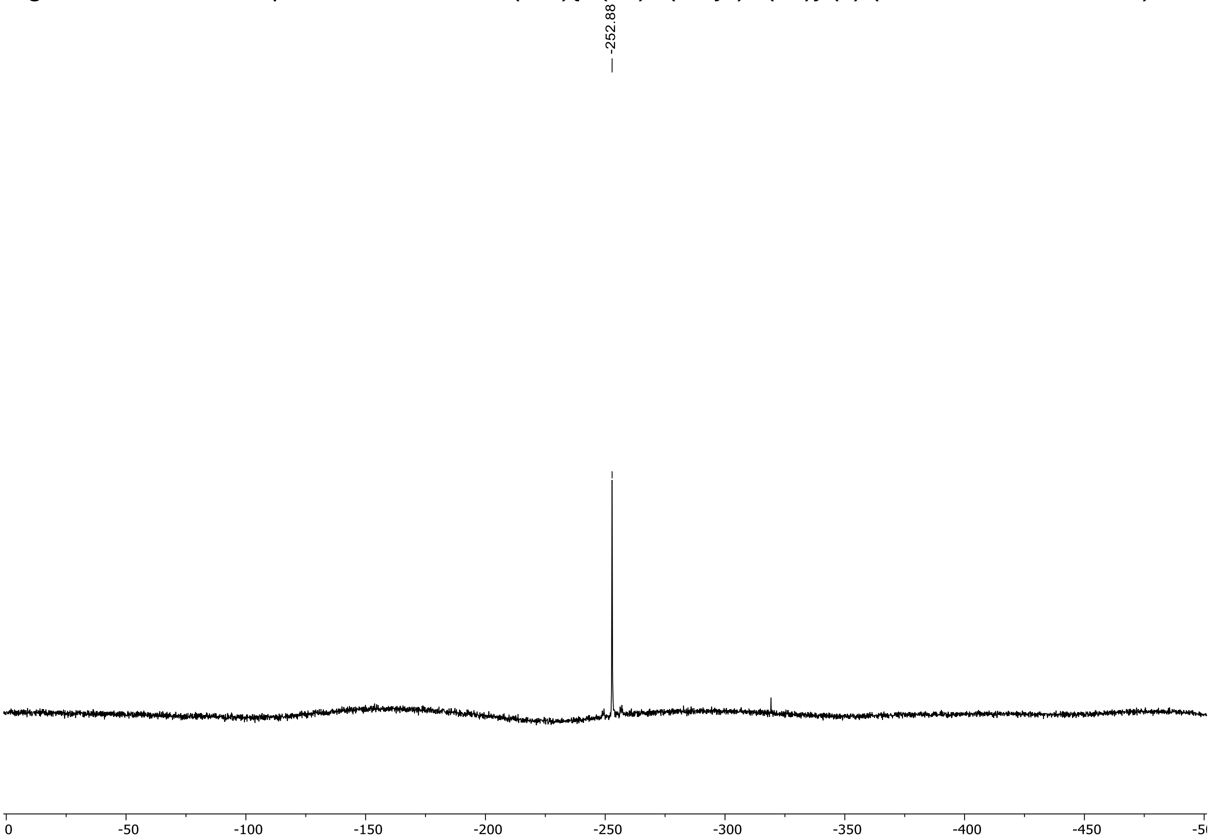

Figure S49. ^{119}Sn{^{1}H} NMR spectrum of ^{Mes}TerSn(Se$_4$){N(^{i}Pr)C(NCy$_2$)N(^{i}Pr)} (**8**) (149 MHz, C_6D_6, 298 K).

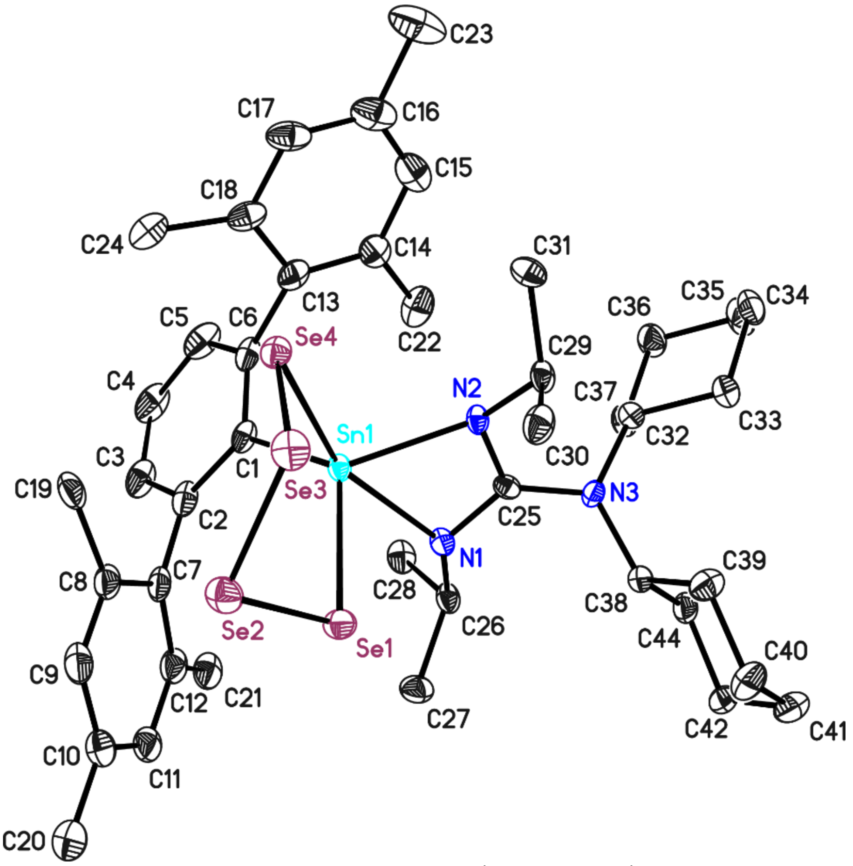

Figure S50. Molecular structure of ^{Mes}TerSn(Se$_4$){N(^{i}Pr)N(Cy$_2$)C(^{i}Pr)} (**8**) in the crystal. Thermal ellipsoids are drawn at the 50% probability level (hydrogen atoms have been omitted for clarity).

The data were collected on an inversion twin. The fractional contribution of the minor compoenent refined to 0.489(8).

Table S6. Bond lengths [Å] and angles [°] for **8.**

C(1)-C(2)	1.416(6)	C(10)-C(11)	1.382(8)
C(1)-C(6)	1.421(7)	C(10)-C(20)	1.503(9)
C(1)-Sn(1)	2.183(4)	C(11)-C(12)	1.386(8)
C(2)-C(3)	1.405(7)	C(12)-C(21)	1.513(7)
C(2)-C(7)	1.500(8)	C(13)-C(14)	1.395(7)
C(3)-C(4)	1.378(9)	C(13)-C(18)	1.413(6)
C(4)-C(5)	1.381(8)	C(14)-C(15)	1.392(8)
C(5)-C(6)	1.401(6)	C(14)-C(22)	1.505(7)
C(6)-C(13)	1.502(6)	C(15)-C(16)	1.380(9)
C(7)-C(8)	1.403(7)	C(16)-C(17)	1.381(9)
C(7)-C(12)	1.413(7)	C(16)-C(23)	1.504(8)
C(8)-C(9)	1.377(8)	C(17)-C(18)	1.393(7)
C(8)-C(19)	1.514(7)	C(18)-C(24)	1.503(7)
C(9)-C(10)	1.394(8)	C(25)-N(1)	1.324(5)

C(25)-N(2)	1.354(5)	C(4)-C(5)-C(6)	120.7(5)
C(25)-N(3)	1.391(5)	C(5)-C(6)-C(1)	119.8(4)
C(26)-N(1)	1.469(6)	C(5)-C(6)-C(13)	116.0(4)
C(26)-C(28)	1.521(7)	C(1)-C(6)-C(13)	124.0(4)
C(26)-C(27)	1.522(7)	C(8)-C(7)-C(12)	118.4(5)
C(29)-N(2)	1.486(5)	C(8)-C(7)-C(2)	118.0(4)
C(29)-C(31)	1.513(7)	C(12)-C(7)-C(2)	123.1(4)
C(29)-C(30)	1.521(7)	C(9)-C(8)-C(7)	119.8(5)
C(32)-N(3)	1.483(5)	C(9)-C(8)-C(19)	118.9(5)
C(32)-C(37)	1.534(6)	C(7)-C(8)-C(19)	121.0(5)
C(32)-C(33)	1.536(6)	C(8)-C(9)-C(10)	122.3(5)
C(33)-C(34)	1.527(7)	C(11)-C(10)-C(9)	117.3(5)
C(34)-C(35)	1.508(9)	C(11)-C(10)-C(20)	122.2(5)
C(35)-C(36)	1.527(7)	C(9)-C(10)-C(20)	120.5(6)
C(36)-C(37)	1.524(7)	C(10)-C(11)-C(12)	122.4(5)
C(38)-N(3)	1.475(6)	C(11)-C(12)-C(7)	119.4(5)
C(38)-C(44)	1.530(6)	C(11)-C(12)-C(21)	118.1(5)
C(38)-C(39)	1.541(7)	C(7)-C(12)-C(21)	122.4(5)
C(39)-C(40)	1.523(7)	C(14)-C(13)-C(18)	119.3(5)
C(40)-C(41)	1.527(8)	C(14)-C(13)-C(6)	121.5(4)
C(41)-C(42)	1.515(8)	C(18)-C(13)-C(6)	118.7(4)
C(42)-C(44)	1.527(7)	C(15)-C(14)-C(13)	119.5(5)
N(1)-Sn(1)	2.315(3)	C(15)-C(14)-C(22)	120.8(5)
N(2)-Sn(1)	2.152(4)	C(13)-C(14)-C(22)	119.7(5)
Se(1)-Se(2)	2.3276(7)	C(16)-C(15)-C(14)	122.0(5)
Se(1)-Sn(1)	2.5654(7)	C(15)-C(16)-C(17)	118.1(5)
Se(2)-Se(3)	2.3244(7)	C(15)-C(16)-C(23)	121.4(6)
Se(3)-Se(4)	2.3400(8)	C(17)-C(16)-C(23)	120.5(6)
Se(4)-Sn(1)	2.6520(6)	C(16)-C(17)-C(18)	122.1(5)
		C(17)-C(18)-C(13)	118.8(5)
C(2)-C(1)-C(6)	118.5(4)	C(17)-C(18)-C(24)	119.1(5)
C(2)-C(1)-Sn(1)	122.6(3)	C(13)-C(18)-C(24)	121.8(4)
C(6)-C(1)-Sn(1)	118.9(3)	N(1)-C(25)-N(2)	112.4(4)
C(3)-C(2)-C(1)	119.2(5)	N(1)-C(25)-N(3)	124.8(4)
C(3)-C(2)-C(7)	116.7(4)	N(2)-C(25)-N(3)	122.8(4)
C(1)-C(2)-C(7)	123.7(4)	N(1)-C(26)-C(28)	109.8(4)
C(4)-C(3)-C(2)	121.4(5)	N(1)-C(26)-C(27)	109.0(4)
C(3)-C(4)-C(5)	119.8(5)	C(28)-C(26)-C(27)	114.3(4)

N(2)-C(29)-C(31)	111.3(4)
N(2)-C(29)-C(30)	111.0(4)
C(31)-C(29)-C(30)	112.8(4)
N(3)-C(32)-C(37)	112.3(4)
N(3)-C(32)-C(33)	115.2(4)
C(37)-C(32)-C(33)	110.3(4)
C(34)-C(33)-C(32)	109.7(4)
C(35)-C(34)-C(33)	111.3(5)
C(34)-C(35)-C(36)	111.2(4)
C(37)-C(36)-C(35)	110.4(4)
C(36)-C(37)-C(32)	111.6(4)
N(3)-C(38)-C(44)	115.1(4)
N(3)-C(38)-C(39)	112.9(4)
C(44)-C(38)-C(39)	110.4(4)
C(40)-C(39)-C(38)	111.4(4)
C(39)-C(40)-C(41)	111.2(4)
C(42)-C(41)-C(40)	110.4(4)
C(41)-C(42)-C(44)	111.2(4)
C(42)-C(44)-C(38)	110.4(4)
C(25)-N(1)-C(26)	123.5(3)
C(25)-N(1)-Sn(1)	90.7(2)
C(26)-N(1)-Sn(1)	142.8(3)
C(25)-N(2)-C(29)	119.1(4)
C(25)-N(2)-Sn(1)	97.1(3)
C(29)-N(2)-Sn(1)	131.2(3)
C(25)-N(3)-C(38)	118.5(3)
C(25)-N(3)-C(32)	116.9(3)
C(38)-N(3)-C(32)	124.6(3)
Se(2)-Se(1)-Sn(1)	99.89(2)
Se(3)-Se(2)-Se(1)	100.18(2)
Se(2)-Se(3)-Se(4)	99.22(3)
Se(3)-Se(4)-Sn(1)	100.95(2)
N(2)-Sn(1)-C(1)	120.96(16)
N(2)-Sn(1)-N(1)	59.64(13)
C(1)-Sn(1)-N(1)	98.69(14)
N(2)-Sn(1)-Se(1)	108.04(10)
C(1)-Sn(1)-Se(1)	123.15(12)
N(1)-Sn(1)-Se(1)	81.70(9)
N(2)-Sn(1)-Se(4)	98.98(10)
C(1)-Sn(1)-Se(4)	99.61(11)
N(1)-Sn(1)-Se(4)	157.20(9)
Se(1)-Sn(1)-Se(4)	99.389(17)

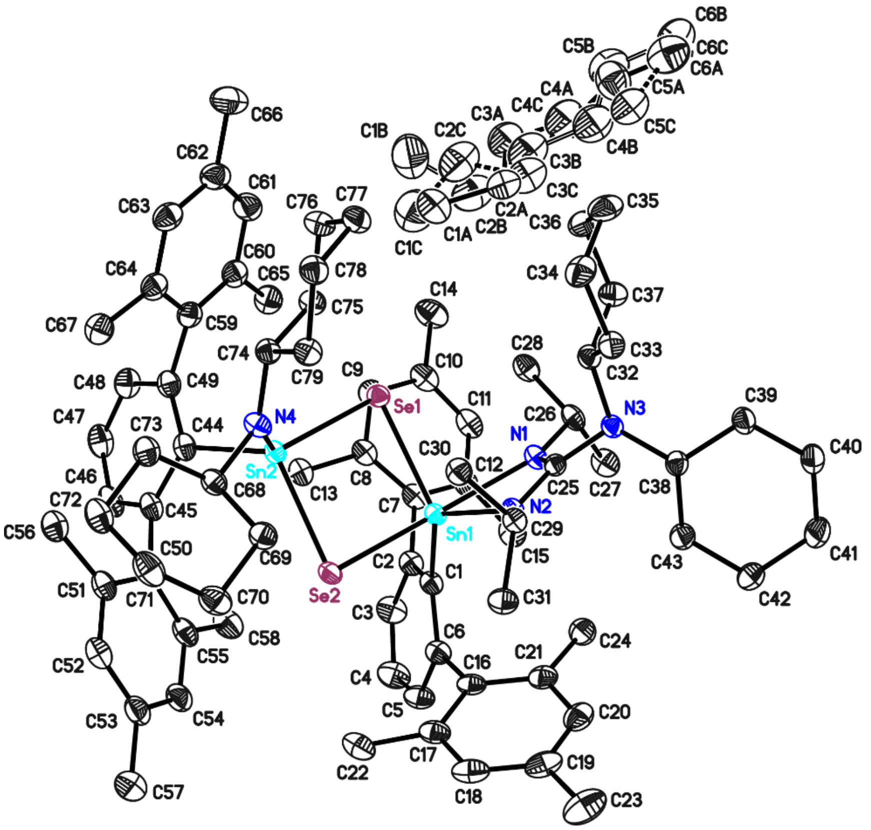

Figure S51. Molecular structure of ^{Mes}TerSn(NCy$_2$)(μ-Se$_2$)Sn{N(^{i}Pr)C(NCy$_2$)N(*i*Pr)}MesTer (**9**) in the crystal. Thermal ellipsoids are drawn at the 50% probability level (hydrogen atoms have been omitted for clarity).

One molecule of hexane was disordered about three positions. The disorder was treated with distance restraints and restraints for the anisotropic displacement parameters. Occupancies refined to 0.637(3), 0.193(3) and 0.170(2) with their sum being fixed to 1.

Table S7. Bond lengths [Å] and angles [°] for **9.**

Sn(1)-N(2)	2.1543(16)	N(3)-C(38)	1.472(2)
Sn(1)-C(1)	2.2132(19)	N(3)-C(32)	1.493(2)
Sn(1)-N(1)	2.2877(16)	N(4)-C(74)	1.474(2)
Sn(1)-Se(1)	2.5582(5)	N(4)-C(68)	1.483(2)
Sn(1)-Se(2)	2.6315(4)	C(1)-C(6)	1.412(3)
Sn(1)-C(25)	2.6604(19)	C(1)-C(2)	1.416(3)
Sn(2)-N(4)	2.0452(17)	C(2)-C(3)	1.400(3)
Sn(2)-C(44)	2.1975(19)	C(2)-C(7)	1.499(3)
Sn(2)-Se(2)	2.5224(5)	C(3)-C(4)	1.377(3)
Sn(2)-Se(1)	2.5542(4)	C(4)-C(5)	1.379(3)
N(1)-C(25)	1.314(2)	C(5)-C(6)	1.400(3)
N(1)-C(26)	1.460(2)	C(6)-C(16)	1.497(3)
N(2)-C(25)	1.347(2)	C(7)-C(8)	1.396(3)
N(2)-C(29)	1.471(2)	C(7)-C(12)	1.413(3)
N(3)-C(25)	1.418(2)	C(8)-C(9)	1.394(3)

C(8)-C(13)	1.507(3)	C(49)-C(59)	1.507(3)
C(9)-C(10)	1.392(3)	C(50)-C(55)	1.401(3)
C(10)-C(11)	1.386(3)	C(50)-C(51)	1.412(3)
C(10)-C(14)	1.513(3)	C(51)-C(52)	1.390(3)
C(11)-C(12)	1.390(3)	C(51)-C(56)	1.512(3)
C(12)-C(15)	1.507(3)	C(52)-C(53)	1.389(3)
C(16)-C(17)	1.406(3)	C(53)-C(54)	1.387(3)
C(16)-C(21)	1.408(3)	C(53)-C(57)	1.506(3)
C(17)-C(18)	1.396(3)	C(54)-C(55)	1.392(3)
C(17)-C(22)	1.507(3)	C(55)-C(58)	1.508(3)
C(18)-C(19)	1.382(4)	C(59)-C(60)	1.402(3)
C(19)-C(20)	1.391(3)	C(59)-C(64)	1.410(3)
C(19)-C(23)	1.506(3)	C(60)-C(61)	1.396(3)
C(20)-C(21)	1.392(3)	C(60)-C(65)	1.505(3)
C(21)-C(24)	1.507(3)	C(61)-C(62)	1.386(3)
C(26)-C(28)	1.521(3)	C(62)-C(63)	1.388(3)
C(26)-C(27)	1.528(3)	C(62)-C(66)	1.501(3)
C(29)-C(31)	1.524(3)	C(63)-C(64)	1.393(3)
C(29)-C(30)	1.527(3)	C(64)-C(67)	1.506(3)
C(32)-C(33)	1.530(3)	C(68)-C(69)	1.538(3)
C(32)-C(37)	1.536(3)	C(68)-C(73)	1.539(3)
C(33)-C(34)	1.529(3)	C(69)-C(70)	1.529(3)
C(34)-C(35)	1.522(3)	C(70)-C(71)	1.521(3)
C(35)-C(36)	1.521(3)	C(71)-C(72)	1.521(3)
C(36)-C(37)	1.534(3)	C(72)-C(73)	1.528(3)
C(38)-C(43)	1.529(3)	C(74)-C(75)	1.528(3)
C(38)-C(39)	1.530(3)	C(74)-C(79)	1.539(3)
C(39)-C(40)	1.529(3)	C(75)-C(76)	1.531(3)
C(40)-C(41)	1.522(3)	C(76)-C(77)	1.528(3)
C(41)-C(42)	1.524(3)	C(77)-C(78)	1.516(3)
C(42)-C(43)	1.530(3)	C(78)-C(79)	1.531(3)
C(44)-C(49)	1.409(3)	C(1A)-C(2A)	1.496(6)
C(44)-C(45)	1.419(3)	C(2A)-C(3A)	1.513(6)
C(45)-C(46)	1.402(3)	C(3A)-C(4A)	1.500(7)
C(45)-C(50)	1.500(3)	C(4A)-C(5A)	1.475(7)
C(46)-C(47)	1.379(3)	C(5A)-C(6A)	1.508(8)
C(47)-C(48)	1.384(3)	C(1B)-C(2B)	1.511(14)
C(48)-C(49)	1.399(3)	C(2B)-C(3B)	1.481(13)

C(3B)-C(4B)	1.429(12)	C(25)-N(3)-C(38)	120.31(15)
C(4B)-C(5B)	1.490(13)	C(25)-N(3)-C(32)	112.58(14)
C(5B)-C(6B)	1.488(14)	C(38)-N(3)-C(32)	120.79(15)
C(1C)-C(2C)	1.445(13)	C(74)-N(4)-C(68)	118.78(15)
C(2C)-C(3C)	1.528(13)	C(74)-N(4)-Sn(2)	127.30(12)
C(3C)-C(4C)	1.478(13)	C(68)-N(4)-Sn(2)	112.43(12)
C(4C)-C(5C)	1.480(13)	C(6)-C(1)-C(2)	118.09(17)
C(5C)-C(6C)	1.488(14)	C(6)-C(1)-Sn(1)	116.61(13)
		C(2)-C(1)-Sn(1)	124.23(14)
N(2)-Sn(1)-C(1)	128.07(7)	C(3)-C(2)-C(1)	119.30(19)
N(2)-Sn(1)-N(1)	59.45(6)	C(3)-C(2)-C(7)	114.60(18)
C(1)-Sn(1)-N(1)	103.46(6)	C(1)-C(2)-C(7)	125.85(18)
N(2)-Sn(1)-Se(1)	102.43(5)	C(4)-C(3)-C(2)	121.9(2)
C(1)-Sn(1)-Se(1)	126.67(5)	C(3)-C(4)-C(5)	118.9(2)
N(1)-Sn(1)-Se(1)	86.69(4)	C(4)-C(5)-C(6)	121.2(2)
N(2)-Sn(1)-Se(2)	99.79(5)	C(5)-C(6)-C(1)	120.12(19)
C(1)-Sn(1)-Se(2)	95.49(5)	C(5)-C(6)-C(16)	114.84(18)
N(1)-Sn(1)-Se(2)	157.90(4)	C(1)-C(6)-C(16)	124.85(17)
Se(1)-Sn(1)-Se(2)	91.086(19)	C(8)-C(7)-C(12)	119.38(19)
N(2)-Sn(1)-C(25)	30.23(6)	C(8)-C(7)-C(2)	121.95(18)
C(1)-Sn(1)-C(25)	122.22(6)	C(12)-C(7)-C(2)	118.01(19)
N(1)-Sn(1)-C(25)	29.59(6)	C(9)-C(8)-C(7)	119.08(19)
Se(1)-Sn(1)-C(25)	91.79(4)	C(9)-C(8)-C(13)	120.52(19)
Se(2)-Sn(1)-C(25)	128.76(4)	C(7)-C(8)-C(13)	120.20(19)
N(4)-Sn(2)-C(44)	116.17(7)	C(10)-C(9)-C(8)	121.9(2)
N(4)-Sn(2)-Se(2)	106.31(5)	C(11)-C(10)-C(9)	117.9(2)
C(44)-Sn(2)-Se(2)	112.04(6)	C(11)-C(10)-C(14)	121.3(2)
N(4)-Sn(2)-Se(1)	116.64(5)	C(9)-C(10)-C(14)	120.8(2)
C(44)-Sn(2)-Se(1)	109.65(5)	C(10)-C(11)-C(12)	121.8(2)
Se(2)-Sn(2)-Se(1)	93.729(19)	C(11)-C(12)-C(7)	119.1(2)
Sn(2)-Se(1)-Sn(1)	88.05(2)	C(11)-C(12)-C(15)	118.88(19)
Sn(2)-Se(2)-Sn(1)	87.131(19)	C(7)-C(12)-C(15)	121.9(2)
C(25)-N(1)-C(26)	123.51(16)	C(17)-C(16)-C(21)	119.47(19)
C(25)-N(1)-Sn(1)	91.12(11)	C(17)-C(16)-C(6)	120.44(19)
C(26)-N(1)-Sn(1)	144.92(12)	C(21)-C(16)-C(6)	119.19(18)
C(25)-N(2)-C(29)	122.77(15)	C(18)-C(17)-C(16)	119.2(2)
C(25)-N(2)-Sn(1)	96.15(11)	C(18)-C(17)-C(22)	119.2(2)
C(29)-N(2)-Sn(1)	135.60(12)	C(16)-C(17)-C(22)	121.5(2)

C(19)-C(18)-C(17)	121.9(2)	C(45)-C(44)-Sn(2)	121.92(15)
C(18)-C(19)-C(20)	118.3(2)	C(46)-C(45)-C(44)	119.34(19)
C(18)-C(19)-C(23)	121.4(2)	C(46)-C(45)-C(50)	116.02(18)
C(20)-C(19)-C(23)	120.3(2)	C(44)-C(45)-C(50)	124.26(18)
C(19)-C(20)-C(21)	121.8(2)	C(47)-C(46)-C(45)	121.3(2)
C(20)-C(21)-C(16)	119.3(2)	C(46)-C(47)-C(48)	119.48(19)
C(20)-C(21)-C(24)	119.5(2)	C(47)-C(48)-C(49)	121.0(2)
C(16)-C(21)-C(24)	121.21(19)	C(48)-C(49)-C(44)	119.95(19)
N(1)-C(25)-N(2)	111.96(16)	C(48)-C(49)-C(59)	115.76(19)
N(1)-C(25)-N(3)	122.28(17)	C(44)-C(49)-C(59)	124.03(18)
N(2)-C(25)-N(3)	125.66(17)	C(55)-C(50)-C(51)	119.16(19)
N(1)-C(25)-Sn(1)	59.29(10)	C(55)-C(50)-C(45)	122.56(18)
N(2)-C(25)-Sn(1)	53.62(9)	C(51)-C(50)-C(45)	117.84(19)
N(3)-C(25)-Sn(1)	167.97(13)	C(52)-C(51)-C(50)	119.2(2)
N(1)-C(26)-C(28)	109.02(16)	C(52)-C(51)-C(56)	119.56(19)
N(1)-C(26)-C(27)	110.65(16)	C(50)-C(51)-C(56)	121.06(19)
C(28)-C(26)-C(27)	112.36(17)	C(53)-C(52)-C(51)	122.1(2)
N(2)-C(29)-C(31)	110.41(15)	C(54)-C(53)-C(52)	117.8(2)
N(2)-C(29)-C(30)	112.56(15)	C(54)-C(53)-C(57)	120.7(2)
C(31)-C(29)-C(30)	110.88(16)	C(52)-C(53)-C(57)	121.4(2)
N(3)-C(32)-C(33)	114.12(16)	C(53)-C(54)-C(55)	122.1(2)
N(3)-C(32)-C(37)	112.85(16)	C(54)-C(55)-C(50)	119.4(2)
C(33)-C(32)-C(37)	110.24(16)	C(54)-C(55)-C(58)	119.5(2)
C(34)-C(33)-C(32)	111.01(17)	C(50)-C(55)-C(58)	121.0(2)
C(35)-C(34)-C(33)	111.58(18)	C(60)-C(59)-C(64)	119.45(19)
C(36)-C(35)-C(34)	110.47(19)	C(60)-C(59)-C(49)	121.61(19)
C(35)-C(36)-C(37)	111.04(18)	C(64)-C(59)-C(49)	118.38(18)
C(36)-C(37)-C(32)	111.14(17)	C(61)-C(60)-C(59)	119.2(2)
N(3)-C(38)-C(43)	111.18(16)	C(61)-C(60)-C(65)	119.2(2)
N(3)-C(38)-C(39)	111.05(16)	C(59)-C(60)-C(65)	121.6(2)
C(43)-C(38)-C(39)	108.90(16)	C(62)-C(61)-C(60)	122.0(2)
C(40)-C(39)-C(38)	110.73(17)	C(61)-C(62)-C(63)	118.0(2)
C(41)-C(40)-C(39)	110.92(18)	C(61)-C(62)-C(66)	120.7(2)
C(40)-C(41)-C(42)	110.26(18)	C(63)-C(62)-C(66)	121.2(2)
C(41)-C(42)-C(43)	111.76(18)	C(62)-C(63)-C(64)	122.0(2)
C(38)-C(43)-C(42)	111.77(17)	C(63)-C(64)-C(59)	119.2(2)
C(49)-C(44)-C(45)	118.59(18)	C(63)-C(64)-C(67)	119.1(2)
C(49)-C(44)-Sn(2)	118.59(14)	C(59)-C(64)-C(67)	121.67(19)

N(4)-C(68)-C(69)	113.12(16)	C(77)-C(78)-C(79)	111.40(17)
N(4)-C(68)-C(73)	114.52(16)	C(78)-C(79)-C(74)	111.02(16)
C(69)-C(68)-C(73)	110.70(16)	C(1A)-C(2A)-C(3A)	112.4(5)
C(70)-C(69)-C(68)	111.56(17)	C(4A)-C(3A)-C(2A)	114.7(5)
C(71)-C(70)-C(69)	111.09(18)	C(5A)-C(4A)-C(3A)	116.5(5)
C(70)-C(71)-C(72)	109.95(18)	C(4A)-C(5A)-C(6A)	114.8(7)
C(71)-C(72)-C(73)	111.70(18)	C(3B)-C(2B)-C(1B)	106.6(15)
C(72)-C(73)-C(68)	111.91(17)	C(4B)-C(3B)-C(2B)	159(2)
N(4)-C(74)-C(75)	111.48(15)	C(3B)-C(4B)-C(5B)	120.4(17)
N(4)-C(74)-C(79)	113.84(16)	C(6B)-C(5B)-C(4B)	116.3(18)
C(75)-C(74)-C(79)	107.69(16)	C(1C)-C(2C)-C(3C)	106.1(14)
C(74)-C(75)-C(76)	111.75(16)	C(4C)-C(3C)-C(2C)	100.7(13)
C(77)-C(76)-C(75)	111.61(17)	C(3C)-C(4C)-C(5C)	115.1(14)
C(78)-C(77)-C(76)	110.42(17)	C(4C)-C(5C)-C(6C)	113.3(19)

Reaction of ^{Mes}TerSn{N(^{i}Pr)C(NCy$_2$)N(^{i}Pr)} (7) with N_2O – Formation of ^{Mes}TerSn{N(^{i}Pr)C(NCy$_2$)N(^{i}Pr)}(μ-O_2)Sn{N(^{i}Pr)C(NCy$_2$)N(^{i}Pr)}^{Mes}Ter (10)

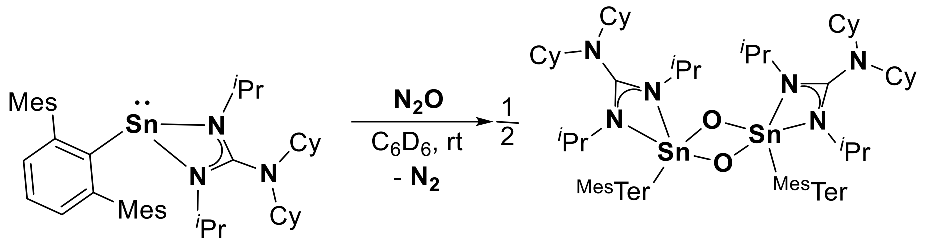

^{Mes}TerSn{N(^{i}Pr)C(NCy$_2$)N(^{i}Pr)} (**7**) (0.030 g, 0.041 mmol) was dissolved in 0.5 mL of C_6D_6. The solution was freeze-pump-thaw degassed three times and backfilled with approximately 1 bar N_2O. The reaction progress was monitored by ^{1}H NMR spectroscopy and shows consumption of **7** within 4 hours at room temperature leading to the formation of ^{Mes}TerSn{N(^{i}Pr)C(NCy$_2$)N(^{i}Pr)}(μ-O_2)Sn{N(^{i}Pr)C(NCy$_2$)N(^{i}Pr)}^{Mes}Ter (**10**) (Figure S52). All volatile components were removed under vacuum, the slightly yellow solid was dissolved in 0.6 mL of *n*-hexane, filtered and stored at -30 °C for several days which leads to precipitation of **10** as a white microcrystalline solid which was analyzed by multinuclear NMR spectroscopy (Figure S53-S55). Crystals of **10** suitable for single crystal X-ray diffraction were obtained from a saturated *n*-pentane solution at -30 °C.

^{1}H NMR (400 MHz, C_6D_6, 298 K): δ = 0.67-0.69 (m, 6H, CH(C*H*$_3$)$_2$), 1.12-1.27 (m, 6H, $CH_{2,Cy}$), 1.18 (d, $^3J_{H,H}$ = 6.3 Hz, 3H, CH(C*H*$_3$)$_2$), 1.23 (d, $^3J_{H,H}$ = 6.8 Hz, 3H, CH(C*H*$_3$)$_2$), 1.39-1.62 (m, 8H, $CH_{2,Cy}$), 1.68-1.74 (m, 4H, $CH_{2,Cy}$), 1.99 (s, 3H, CH_3), 2.17 (CH_3), 2.20 (CH_3), 2.24 (CH_3), 2.36 (CH_3), 2.97 (CH_3), 2.98-3.07 (m, 2H, CH_{Cy}, C*H*(CH$_3$)$_2$), 3.23-3.28 (m, 1H, CH_{Cy}), 3.51 (hept, $^3J_{H,H}$ = 6.5 Hz, 1H, C*H*(CH$_3$)$_2$), 6.83-6.85 (m, 3H, CH_{Aryl}), 6.96-6.97 (m, 2H, CH_{Aryl}), 7.01-7.03 (m, 1H, CH_{Aryl}), 7.19-7.22 (m, 1H, CH_{Aryl}) ppm.
^{13}C{^{1}H} NMR (101 MHz, C_6D_6, 298 K): δ = 21.2 (2xCH_3), 22.66 (CH_3), 22.74 (CH_3), 23.5 (CH_3), 24.0 (CH($\underline{C}$H$_3$)$_2$), 24.4 (CH($\underline{C}$H$_3$)$_2$), 24.71 (CH($\underline{C}$H$_3$)$_2$ / CH_3), 24.73 (CH($\underline{C}$H$_3$)$_2$ / CH_3), 25.8 (CH($\underline{C}$H$_3$)$_2$), 26.2 (2x$CH_{2,Cy}$), 27.4 ($CH_{2,Cy}$), 27.6 ($CH_{2,Cy}$), 27.8 ($CH_{2,Cy}$), 27.9 ($CH_{2,Cy}$), 34.7 ($CH_{2,Cy}$), 35.1 ($CH_{2,Cy}$), 36.1 ($CH_{2,Cy}$), 36.6 ($CH_{2,Cy}$), 47.3 ($\underline{C}$H(CH$_3$)$_2$), 48.0 ($\underline{C}$H(CH$_3$)$_2$), 60.0 (CH_{Cy}), 61.4 (CH_{Cy}), 128.2 (CH_{Aryl})*, 128.8 (CH_{Aryl}), 129.2 (CH_{Aryl}), 129.5 (CH_{Aryl}), 129.8 (CH_{Aryl}), 129.9 (CH_{Aryl}), 130.7 (CH_{Aryl}), 136.0 ($C_{q,Aryl}$), 136.9 ($C_{q,Aryl}$), 138.3 ($C_{q,Aryl}$), 138.4 ($C_{q,Aryl}$), 138.7 ($C_{q,Aryl}$), 139.4 ($C_{q,Aryl}$), 142.6 ($C_{q,Aryl}$), 143.7 ($C_{q,Aryl}$), 149.7 ($C_{q,Aryl}$), 150.0 ($C_{q,Aryl}$), 150.5 ($C_{q,Aryl}$), 169.7 (C_q(N^{i}Pr)$_2$NCy$_2$) ppm.
* = overlap with C_6D_6 signal
^{119}Sn{^{1}H} NMR (149 MHz, C_6D_6, 298 K): δ = -317.4 ppm.
MS (LIFDI): m/z calcd. for $C_{86}H_{122}N_6O_2Sn$: 1508.7667; found: 1508.5.

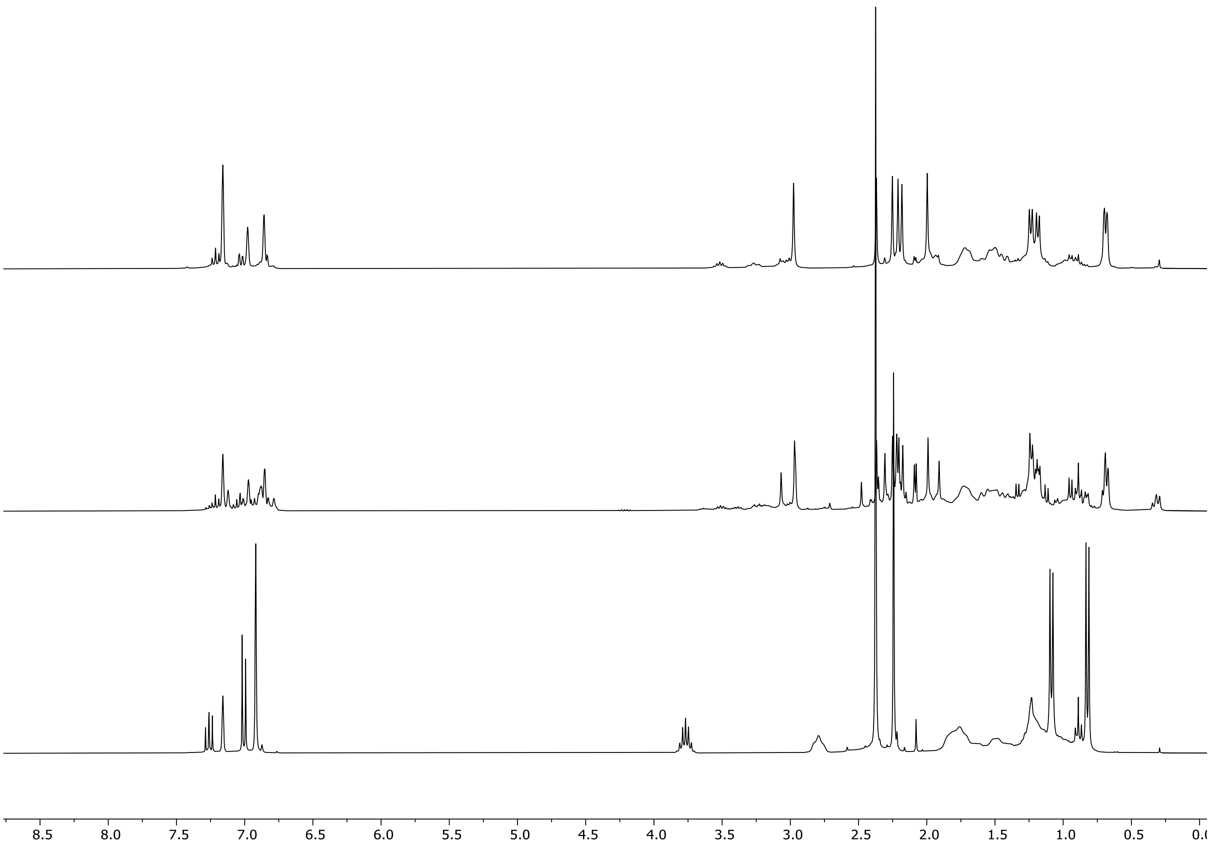

Figure S52. Monitoring of the reaction of ^{Mes}TerSn{N(^{i}Pr)C(NCy$_2$)N(^{i}Pr)} (**7**) with N_2O via ^{1}H NMR spectroscopy. Bottom: **7** before backfilling with N_2O; Middle: After backfilling with N_2O and complete consumption of **7**; Top: 1H NMR of isolated ^{Mes}TerSn{N(^{i}Pr)C(NCy$_2$)N(^{i}Pr)}(μ-O$_2$)Sn{N(^{i}Pr)C(NCy$_2$)N(^{i}Pr)}^{Mes}Ter (**10**).

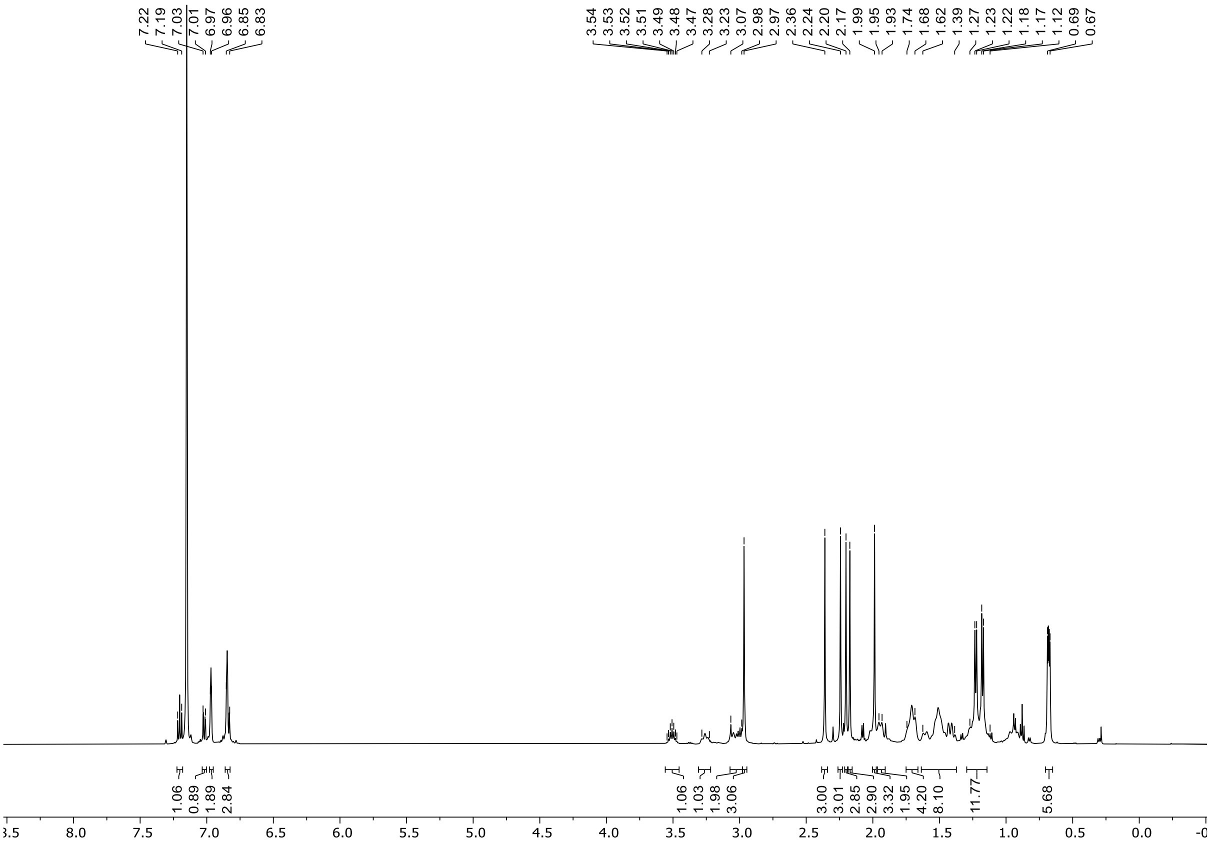

Figure S53. ^{1}H NMR spectrum of ^{Mes}TerSn{N(^{i}Pr)C(NCy$_2$)N(^{i}Pr)}(μ-O_2)Sn{N(^{i}Pr)C(NCy$_2$)N(^{i}Pr)}^{Mes}Ter (**10**) (400 MHz, C_6D_6, 298 K).

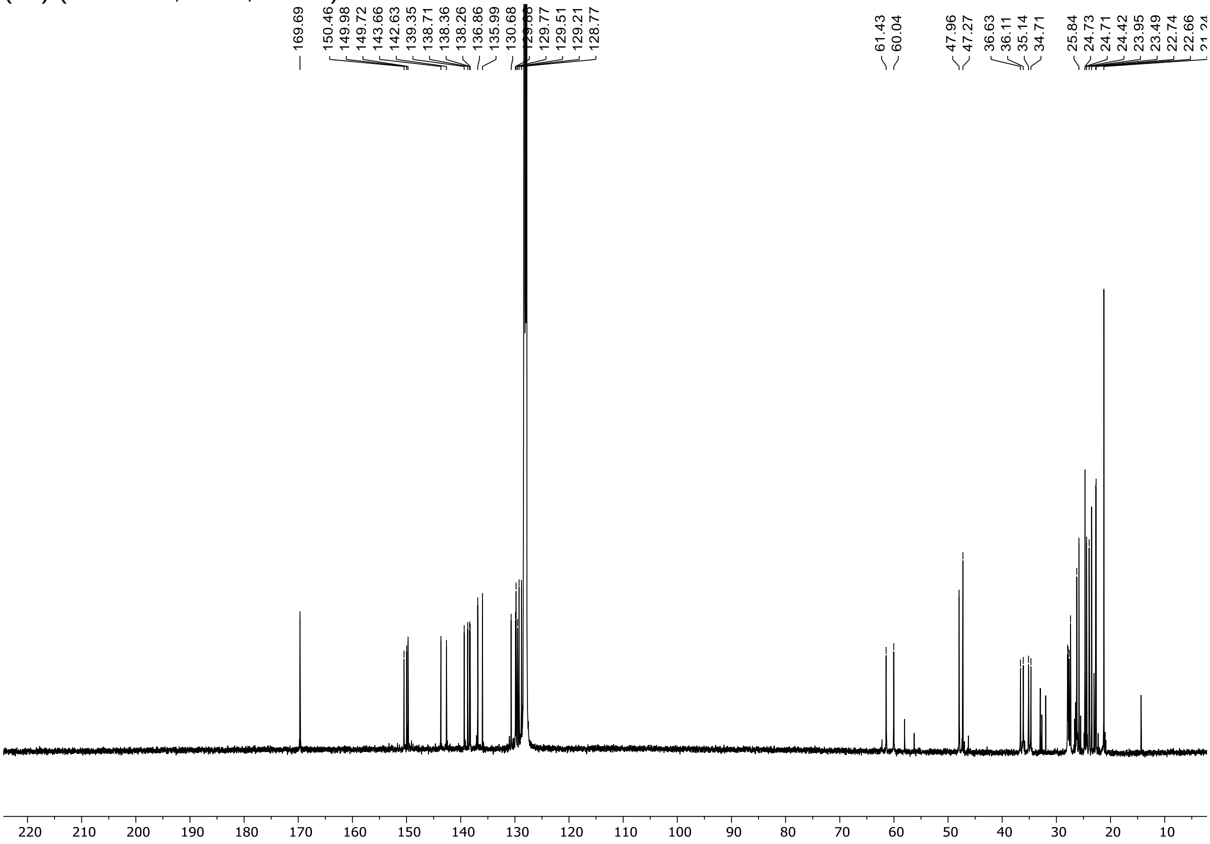

Figure S54. ^{13}C{^{1}H} NMR spectrum of ^{Mes}TerSn{N(^{i}Pr)C(NCy$_2$)N(^{i}Pr)}(μ-O_2)Sn{N(^{i}Pr)C(NCy$_2$)N(^{i}Pr)}^{Mes}Ter (**10**) (126 MHz, C_6D_6, 298 K).

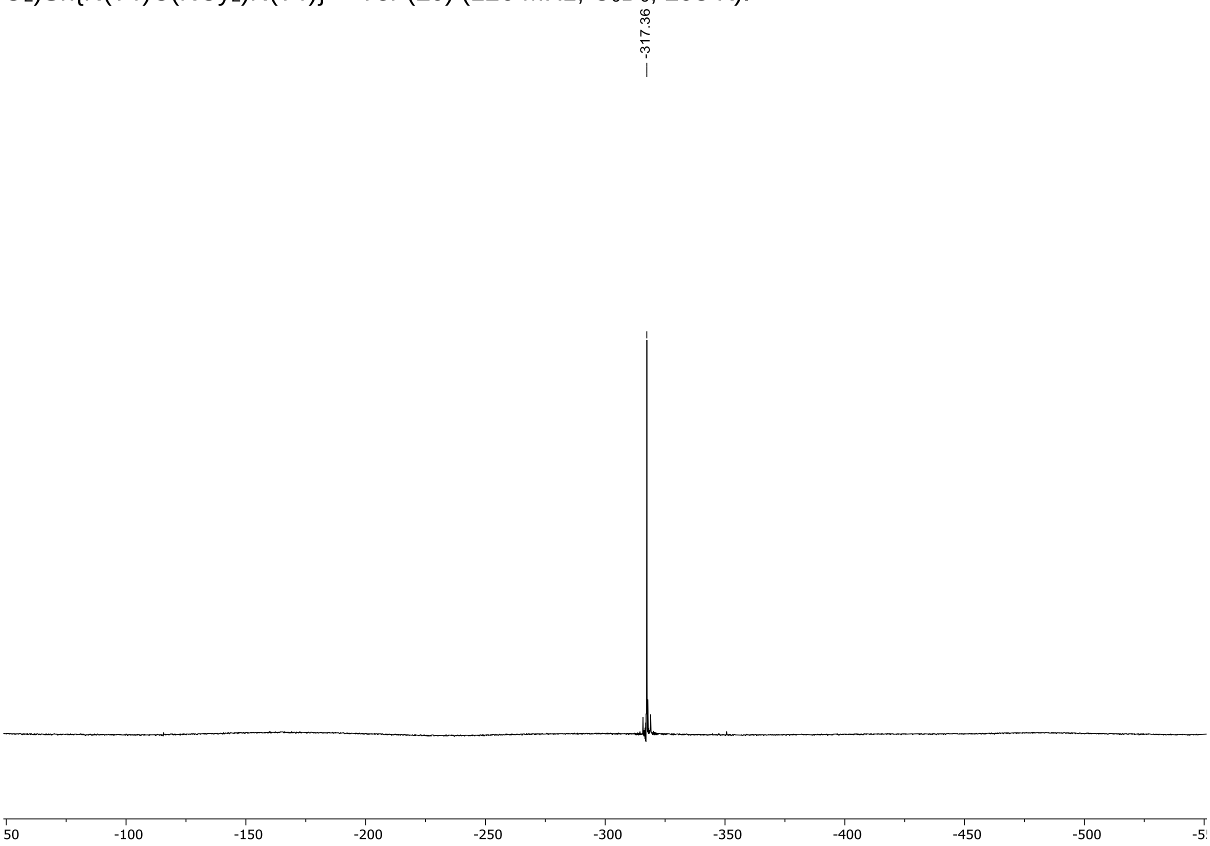

Figure S55. $^{119}Sn\{^{1}H\}$ NMR spectrum of $^{Mes}TerSn\{N(^{i}Pr)C(NCy_2)N(^{i}Pr)\}(\mu\text{-}O_2)Sn\{N(^{i}Pr)C(NCy_2)N(^{i}Pr)\}^{Mes}Ter$ (**10**) (149 MHz, C_6D_6, 298 K).

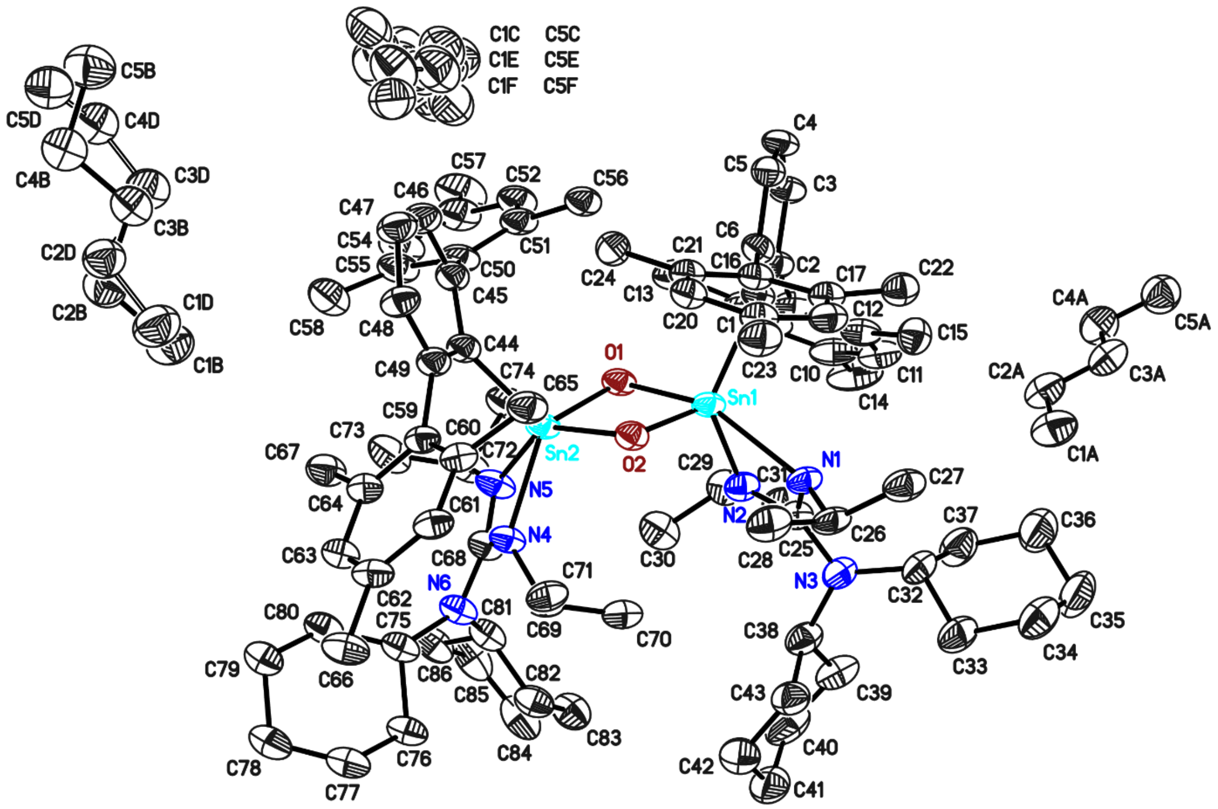

Figure S56. Molecular structure of ^{Mes}TerSn{N(^{i}Pr)C(NCy$_2$)N^{i}Pr}(μ-O$_2$)Sn{N(^{i}Pr)C(NCy$_2$)N^{i}Pr}^{Mes}Ter (**10**) in the crystal. Thermal ellipsoids are drawn at the 50% probability level (hydrogen atoms, mesityl groups and lattice solvent have been omitted for clarity).

The asymmetric unit contains three molecules of hexane, two of which were disordered. The disorder was treated with distance restraints and restraints for the anisotropic displacement parameters. One molecule was disordered about two positions. The occupancy of the minor component refined to 0.390(12). One molecule was disordered about three positions. The occupancies refined to 0.445(3), 0.384(3) and 0.170(3) with their sum being fixed to 1.

Table S8. Bond lengths [Å] and angles [°] for **10.**

C(1)-C(2)	1.400(6)	Sn(2)-C(44)	2.193(5)
C(1)-C(6)	1.406(6)	Sn(2)-N(5)	2.236(4)
C(1)-Sn(1)	2.185(4)	Sn(2)-C(68)	2.660(4)
N(1)-C(25)	1.338(5)	N(2)-C(25)	1.350(5)
N(1)-C(26)	1.472(5)	N(2)-C(29)	1.474(5)
N(1)-Sn(1)	2.269(3)	C(2)-C(3)	1.407(6)
O(1)-Sn(2)	1.988(3)	C(2)-C(7)	1.506(6)
O(1)-Sn(1)	2.039(3)	N(3)-C(25)	1.393(5)
Sn(1)-O(2)	1.984(3)	N(3)-C(38)	1.474(6)
Sn(1)-N(2)	2.140(4)	N(3)-C(32)	1.477(6)
Sn(1)-C(25)	2.663(4)	C(3)-C(4)	1.373(6)
Sn(1)-Sn(2)	3.0327(8)	N(4)-C(68)	1.358(5)
O(2)-Sn(2)	2.052(3)	N(4)-C(69)	1.484(5)
Sn(2)-N(4)	2.160(3)	C(4)-C(5)	1.377(6)

N(5)-C(68)	1.329(5)	C(40)-C(41)	1.518(9)
N(5)-C(72)	1.475(5)	C(41)-C(42)	1.519(8)
C(5)-C(6)	1.393(6)	C(42)-C(43)	1.539(7)
N(6)-C(68)	1.395(5)	C(44)-C(49)	1.403(6)
N(6)-C(81)	1.469(6)	C(44)-C(45)	1.427(6)
N(6)-C(75)	1.477(6)	C(45)-C(46)	1.379(7)
C(6)-C(16)	1.502(6)	C(45)-C(50)	1.502(6)
C(7)-C(12)	1.406(7)	C(46)-C(47)	1.378(7)
C(7)-C(8)	1.409(6)	C(47)-C(48)	1.388(6)
C(8)-C(9)	1.384(7)	C(48)-C(49)	1.394(6)
C(8)-C(13)	1.497(8)	C(49)-C(59)	1.517(5)
C(9)-C(10)	1.373(8)	C(50)-C(51)	1.397(7)
C(10)-C(11)	1.407(8)	C(50)-C(55)	1.416(6)
C(10)-C(14)	1.513(7)	C(51)-C(52)	1.393(6)
C(11)-C(12)	1.383(7)	C(51)-C(56)	1.499(6)
C(12)-C(15)	1.510(7)	C(52)-C(53)	1.401(7)
C(16)-C(21)	1.397(6)	C(53)-C(54)	1.369(8)
C(16)-C(17)	1.411(6)	C(53)-C(57)	1.511(7)
C(17)-C(18)	1.397(6)	C(54)-C(55)	1.396(7)
C(17)-C(22)	1.503(6)	C(55)-C(58)	1.494(8)
C(18)-C(19)	1.383(6)	C(59)-C(64)	1.397(6)
C(19)-C(20)	1.386(6)	C(59)-C(60)	1.404(6)
C(19)-C(23)	1.504(6)	C(60)-C(61)	1.392(6)
C(20)-C(21)	1.394(6)	C(60)-C(65)	1.507(6)
C(21)-C(24)	1.504(6)	C(61)-C(62)	1.385(6)
C(26)-C(28)	1.509(7)	C(62)-C(63)	1.387(6)
C(26)-C(27)	1.527(6)	C(62)-C(66)	1.511(6)
C(29)-C(31)	1.486(7)	C(63)-C(64)	1.400(6)
C(29)-C(30)	1.525(7)	C(64)-C(67)	1.507(6)
C(32)-C(33)	1.531(6)	C(69)-C(71)	1.512(6)
C(32)-C(37)	1.532(7)	C(69)-C(70)	1.516(7)
C(33)-C(34)	1.518(8)	C(72)-C(73)	1.523(8)
C(34)-C(35)	1.498(9)	C(72)-C(74)	1.531(6)
C(35)-C(36)	1.528(8)	C(75)-C(76)	1.532(6)
C(36)-C(37)	1.539(8)	C(75)-C(80)	1.536(7)
C(38)-C(39)	1.517(7)	C(76)-C(77)	1.528(6)
C(38)-C(43)	1.530(7)	C(77)-C(78)	1.520(8)
C(39)-C(40)	1.515(8)	C(78)-C(79)	1.525(7)

C(79)-C(80)	1.524(7)	Sn(2)-O(1)-Sn(1)	97.73(11)
C(81)-C(86)	1.534(6)	O(2)-Sn(1)-O(1)	82.10(11)
C(81)-C(82)	1.541(7)	O(2)-Sn(1)-N(2)	115.43(13)
C(82)-C(83)	1.528(7)	O(1)-Sn(1)-N(2)	92.03(13)
C(83)-C(84)	1.521(7)	O(2)-Sn(1)-C(1)	121.89(14)
C(84)-C(85)	1.509(9)	O(1)-Sn(1)-C(1)	98.96(13)
C(85)-C(86)	1.534(7)	N(2)-Sn(1)-C(1)	122.54(15)
C(1A)-C(2A)	1.518(7)	O(2)-Sn(1)-N(1)	101.05(12)
C(2A)-C(3A)	1.503(7)	O(1)-Sn(1)-N(1)	151.04(12)
C(3A)-C(4A)	1.509(7)	N(2)-Sn(1)-N(1)	60.39(13)
C(4A)-C(5A)	1.499(8)	C(1)-Sn(1)-N(1)	103.38(14)
C(1B)-C(2B)	1.520(11)	O(2)-Sn(1)-C(25)	110.83(12)
C(2B)-C(3B)	1.508(10)	O(1)-Sn(1)-C(25)	121.76(12)
C(3B)-C(4B)	1.519(10)	N(2)-Sn(1)-C(25)	30.23(13)
C(4B)-C(5B)	1.510(11)	C(1)-Sn(1)-C(25)	116.63(15)
C(1D)-C(2D)	1.522(13)	N(1)-Sn(1)-C(25)	30.16(12)
C(2D)-C(3D)	1.505(13)	O(2)-Sn(1)-Sn(2)	42.15(8)
C(3D)-C(4D)	1.520(13)	O(1)-Sn(1)-Sn(2)	40.50(8)
C(4D)-C(5D)	1.496(13)	N(2)-Sn(1)-Sn(2)	102.68(10)
C(1C)-C(2C)	1.499(14)	C(1)-Sn(1)-Sn(2)	121.68(11)
C(2C)-C(3C)	1.502(14)	N(1)-Sn(1)-Sn(2)	131.88(9)
C(3C)-C(4C)	1.492(14)	C(25)-Sn(1)-Sn(2)	120.69(9)
C(4C)-C(5C)	1.504(14)	Sn(1)-O(2)-Sn(2)	97.39(11)
C(1E)-C(2E)	1.491(14)	O(1)-Sn(2)-O(2)	81.67(11)
C(2E)-C(3E)	1.505(13)	O(1)-Sn(2)-N(4)	131.52(13)
C(3E)-C(4E)	1.516(14)	O(2)-Sn(2)-N(4)	94.56(12)
C(4E)-C(5E)	1.488(14)	O(1)-Sn(2)-C(44)	116.10(13)
C(1F)-C(2F)	1.495(16)	O(2)-Sn(2)-C(44)	106.32(13)
C(2F)-C(3F)	1.503(16)	N(4)-Sn(2)-C(44)	111.33(15)
C(3F)-C(4F)	1.501(16)	O(1)-Sn(2)-N(5)	91.98(13)
C(4F)-C(5F)	1.482(16)	O(2)-Sn(2)-N(5)	139.47(13)
		N(4)-Sn(2)-N(5)	60.44(13)
C(2)-C(1)-C(6)	118.6(4)	C(44)-Sn(2)-N(5)	112.28(15)
C(2)-C(1)-Sn(1)	118.9(3)	O(1)-Sn(2)-C(68)	113.32(13)
C(6)-C(1)-Sn(1)	121.1(3)	O(2)-Sn(2)-C(68)	119.06(12)
C(25)-N(1)-C(26)	121.0(3)	N(4)-Sn(2)-C(68)	30.53(13)
C(25)-N(1)-Sn(1)	91.4(2)	C(44)-Sn(2)-C(68)	115.85(15)
C(26)-N(1)-Sn(1)	135.2(3)	N(5)-Sn(2)-C(68)	29.92(13)

O(1)-Sn(2)-Sn(1)	41.77(8)	C(9)-C(10)-C(14)	121.1(5)
O(2)-Sn(2)-Sn(1)	40.46(8)	C(11)-C(10)-C(14)	120.5(6)
N(4)-Sn(2)-Sn(1)	114.53(10)	C(12)-C(11)-C(10)	121.5(5)
C(44)-Sn(2)-Sn(1)	123.54(11)	C(11)-C(12)-C(7)	118.9(5)
N(5)-Sn(2)-Sn(1)	118.06(11)	C(11)-C(12)-C(15)	120.2(5)
C(68)-Sn(2)-Sn(1)	120.46(10)	C(7)-C(12)-C(15)	120.9(4)
C(25)-N(2)-C(29)	133.9(4)	C(21)-C(16)-C(17)	119.8(4)
C(25)-N(2)-Sn(1)	96.8(3)	C(21)-C(16)-C(6)	121.6(4)
C(29)-N(2)-Sn(1)	126.0(3)	C(17)-C(16)-C(6)	117.9(4)
C(1)-C(2)-C(3)	119.4(4)	C(18)-C(17)-C(16)	118.7(4)
C(1)-C(2)-C(7)	126.8(4)	C(18)-C(17)-C(22)	120.8(4)
C(3)-C(2)-C(7)	113.5(4)	C(16)-C(17)-C(22)	120.4(4)
C(25)-N(3)-C(38)	118.6(4)	C(19)-C(18)-C(17)	122.2(4)
C(25)-N(3)-C(32)	117.5(4)	C(18)-C(19)-C(20)	118.0(4)
C(38)-N(3)-C(32)	123.5(4)	C(18)-C(19)-C(23)	121.9(4)
C(4)-C(3)-C(2)	121.4(4)	C(20)-C(19)-C(23)	120.0(4)
C(68)-N(4)-C(69)	118.9(3)	C(19)-C(20)-C(21)	122.1(4)
C(68)-N(4)-Sn(2)	95.6(2)	C(20)-C(21)-C(16)	119.2(4)
C(69)-N(4)-Sn(2)	129.6(3)	C(20)-C(21)-C(24)	119.9(4)
C(3)-C(4)-C(5)	119.0(4)	C(16)-C(21)-C(24)	120.9(4)
C(68)-N(5)-C(72)	127.1(4)	N(1)-C(25)-N(2)	111.3(4)
C(68)-N(5)-Sn(2)	93.0(3)	N(1)-C(25)-N(3)	124.0(4)
C(72)-N(5)-Sn(2)	138.7(3)	N(2)-C(25)-N(3)	124.7(4)
C(4)-C(5)-C(6)	121.3(4)	N(1)-C(25)-Sn(1)	58.4(2)
C(68)-N(6)-C(81)	118.3(4)	N(2)-C(25)-Sn(1)	52.9(2)
C(68)-N(6)-C(75)	116.6(4)	N(3)-C(25)-Sn(1)	177.5(3)
C(81)-N(6)-C(75)	125.0(3)	N(1)-C(26)-C(28)	110.2(4)
C(5)-C(6)-C(1)	119.9(4)	N(1)-C(26)-C(27)	112.7(4)
C(5)-C(6)-C(16)	115.5(4)	C(28)-C(26)-C(27)	109.6(4)
C(1)-C(6)-C(16)	124.3(4)	N(2)-C(29)-C(31)	117.9(4)
C(12)-C(7)-C(8)	120.2(4)	N(2)-C(29)-C(30)	110.4(4)
C(12)-C(7)-C(2)	118.3(4)	C(31)-C(29)-C(30)	112.2(4)
C(8)-C(7)-C(2)	120.7(4)	N(3)-C(32)-C(33)	116.0(4)
C(9)-C(8)-C(7)	118.7(5)	N(3)-C(32)-C(37)	113.3(4)
C(9)-C(8)-C(13)	119.4(4)	C(33)-C(32)-C(37)	109.1(4)
C(7)-C(8)-C(13)	121.8(4)	C(34)-C(33)-C(32)	111.0(4)
C(10)-C(9)-C(8)	122.4(5)	C(35)-C(34)-C(33)	111.0(5)
C(9)-C(10)-C(11)	118.3(5)	C(34)-C(35)-C(36)	111.5(5)

C(35)-C(36)-C(37)	111.3(5)	C(60)-C(59)-C(49)	120.3(4)
C(32)-C(37)-C(36)	109.8(5)	C(61)-C(60)-C(59)	119.3(4)
N(3)-C(38)-C(39)	116.5(4)	C(61)-C(60)-C(65)	119.6(4)
N(3)-C(38)-C(43)	114.1(4)	C(59)-C(60)-C(65)	121.0(4)
C(39)-C(38)-C(43)	108.8(4)	C(62)-C(61)-C(60)	122.2(4)
C(40)-C(39)-C(38)	110.3(5)	C(61)-C(62)-C(63)	117.7(4)
C(39)-C(40)-C(41)	110.7(5)	C(61)-C(62)-C(66)	121.2(4)
C(40)-C(41)-C(42)	111.6(5)	C(63)-C(62)-C(66)	121.1(4)
C(41)-C(42)-C(43)	111.9(5)	C(62)-C(63)-C(64)	121.9(4)
C(38)-C(43)-C(42)	109.6(4)	C(59)-C(64)-C(63)	119.5(4)
C(49)-C(44)-C(45)	117.2(4)	C(59)-C(64)-C(67)	121.1(4)
C(49)-C(44)-Sn(2)	119.4(3)	C(63)-C(64)-C(67)	119.3(4)
C(45)-C(44)-Sn(2)	121.9(3)	N(5)-C(68)-N(4)	110.9(4)
C(46)-C(45)-C(44)	120.1(4)	N(5)-C(68)-N(6)	127.5(4)
C(46)-C(45)-C(50)	113.6(4)	N(4)-C(68)-N(6)	121.6(4)
C(44)-C(45)-C(50)	125.7(4)	N(5)-C(68)-Sn(2)	57.0(2)
C(47)-C(46)-C(45)	122.1(4)	N(4)-C(68)-Sn(2)	53.9(2)
C(46)-C(47)-C(48)	118.3(4)	N(6)-C(68)-Sn(2)	175.4(3)
C(47)-C(48)-C(49)	121.2(4)	N(4)-C(69)-C(71)	110.6(3)
C(48)-C(49)-C(44)	120.6(4)	N(4)-C(69)-C(70)	111.3(3)
C(48)-C(49)-C(59)	111.7(4)	C(71)-C(69)-C(70)	111.1(4)
C(44)-C(49)-C(59)	127.3(4)	N(5)-C(72)-C(73)	112.6(4)
C(51)-C(50)-C(55)	119.8(4)	N(5)-C(72)-C(74)	107.4(4)
C(51)-C(50)-C(45)	122.4(4)	C(73)-C(72)-C(74)	111.2(4)
C(55)-C(50)-C(45)	116.7(4)	N(6)-C(75)-C(76)	116.3(4)
C(52)-C(51)-C(50)	119.2(4)	N(6)-C(75)-C(80)	113.3(4)
C(52)-C(51)-C(56)	119.9(4)	C(76)-C(75)-C(80)	109.5(4)
C(50)-C(51)-C(56)	120.9(4)	C(77)-C(76)-C(75)	110.0(4)
C(51)-C(52)-C(53)	121.5(5)	C(78)-C(77)-C(76)	111.6(4)
C(54)-C(53)-C(52)	118.5(4)	C(77)-C(78)-C(79)	110.6(4)
C(54)-C(53)-C(57)	121.8(5)	C(80)-C(79)-C(78)	111.7(4)
C(52)-C(53)-C(57)	119.7(5)	C(79)-C(80)-C(75)	110.8(4)
C(53)-C(54)-C(55)	122.2(5)	N(6)-C(81)-C(86)	115.9(4)
C(54)-C(55)-C(50)	118.6(5)	N(6)-C(81)-C(82)	114.7(4)
C(54)-C(55)-C(58)	120.1(5)	C(86)-C(81)-C(82)	109.0(4)
C(50)-C(55)-C(58)	121.2(4)	C(83)-C(82)-C(81)	110.2(4)
C(64)-C(59)-C(60)	119.3(4)	C(84)-C(83)-C(82)	110.8(4)
C(64)-C(59)-C(49)	119.1(4)	C(85)-C(84)-C(83)	111.4(5)

C(84)-C(85)-C(86)	112.8(5)
C(85)-C(86)-C(81)	109.9(4)
C(3A)-C(2A)-C(1A)	113.4(5)
C(2A)-C(3A)-C(4A)	113.6(5)
C(5A)-C(4A)-C(3A)	112.6(5)
C(3B)-C(2B)-C(1B)	113.1(10)
C(2B)-C(3B)-C(4B)	112.2(9)
C(5B)-C(4B)-C(3B)	113.2(9)
C(3D)-C(2D)-C(1D)	113.1(14)
C(2D)-C(3D)-C(4D)	113.6(13)
C(5D)-C(4D)-C(3D)	114.2(12)
C(1C)-C(2C)-C(3C)	113.7(16)
C(4C)-C(3C)-C(2C)	118.6(17)
C(3C)-C(4C)-C(5C)	110.2(16)
C(1E)-C(2E)-C(3E)	114.5(15)
C(2E)-C(3E)-C(4E)	114.4(14)
C(5E)-C(4E)-C(3E)	111.5(15)
C(1F)-C(2F)-C(3F)	116(2)
C(4F)-C(3F)-C(2F)	113(2)
C(5F)-C(4F)-C(3F)	120(2)

Crystallographic Details

Single crystal X-ray data were collected at 100 K or 110 K using an open flow nitrogen stream on a Bruker Venture D8 diffractometer with a Photon 100 detector in shutterless mode using an Incoatec microfocus source (compounds **6**, **7** and **8**), on a Bruker Smart Apex diffractometer equipped with a Bruker rotating anode (Mo) Incoatec , Incoatec mirror optics and a Photon III detector (compounds **2a**, **3b**, **4b** and **10**) or a Bruker D8 venture diffractometer with an Excilum Metaljet X-ray source (In), Incoatec mirror optics and a Dectris Eiger 2 CdTe 1M detector (compound **9**). The data were integrated with SAINT[S4]. A multi-scan absorption correction was applied using SADABS[S5]. All structures were solved using the dual-space algorithm in ShelXT[S6] and refined against F^2 with the use of SHELXL[S7] within the OLEX2[S8] program package (compounds **6**, **7** and **8**) or in the graphical user interface SelXle[S9] (compounds **2a**, **3b**, **4b**, **9** and **10**). All non-hydrogen atoms were refined using anisotropic displacement parameters. Unless noted otherwise, hydrogen atoms were refined using a riding model with their U_{iso} values constrained to 1.5 U_{eq} of their pivot atoms for terminal sp^3 carbon atoms and 1.2 times for all other carbon atoms. Crystallographic data for the structural analyses have been deposited with the Cambridge Crystallographic Data Centre under reference numbers 2330043–2330050. Copies of this information may be obtained free of charge from The Director, CCDC, 12 Union Road, Cambridge CB2 1EZ, UK (Fax: +44-1223-336033; email: deposit@ccdc.cam.ac.uk or http://www.ccdc.cam.ac.uk).

Table S9. Crystal structure data for compounds **2a**, **3b** and **4b**.

	**2a**	**3b**	**4b**
CCDC	2330043	2330044	2330045
empirical formula	$C_{37}H_{57}N_3Si_2Sn$	$C_{43}H_{69}N_3SeSi_2Sn$	$C_{43}H_{69}N_3OSi_2Sn$
formula weight	718.72	881.84	818.88
colour	yellow	yellow	colourless
habit	block	block	block
cryst. dimens, mm	0.37 x 0.18 x 0.15	0.31 x 0.27 x 0.21	0.38 x 0.16 x 0.10
T, K	100(2)	100(2)	100(2)
crystal system	monoclinic	cubic	monoclinic
space group	$P2_1/c$	$F\bar{4}3c$	$P2_1/c$
a, Å	16.497(2)	51.964(5)	10.904(2)
b, Å	12.691(2)	51.964(5)	13.130(2)
c, Å	18.626(3)	51.964(5)	31.450(3)
β, deg	93.86(2)	90	96.84(2)
V, Å^3	3890.8(10)	140316(41)	4470.6(12)
Z	4	96	4
D_{calc}, g•cm^{-3}	1.227	1.002	1.217
μ, mm^{-1}	0.745	1.125	0.658
radiation	Mo $K\alpha$ (λ = 0.71073)	Mo $K\alpha$ (λ = 0.71073)	Mo $K\alpha$ (λ = 0.71073)
θ range, deg	1.943 – 26.411	1.753 – 25.056	1.881 – 26.383
no. of rflns collected	295157	454650	199591
no. of indep. reflns.	7953	10386	9160
R(int)	0.0452	0.1407	0.0614
[I>2σ(I)]	$R1$ = 0.0264<br>$wR2$ = 0.0708	$R1$ = 0.0281<br>$wR2$ = 0.0686	$R1$ = 0.0240<br>$wR2$ = 0.0529
R indices (all data)	$R1$ = 0.0289<br>$wR2$ = 0.0728	$R1$ = 0.0338<br>$wR2$ = 0.0716	$R1$ = 0.0305<br>$wR2$ = 0.0561
GOF on F^2	1.139	1.044	1.077
Flack x param.[S10]	-	0.008(4)	-
$\Delta\rho_{max}/\Delta\rho_{min,}$ e Å^{-3}	1.072 / -0.548	0.379 / -0.234	0.426 / -0.492

Table S10. Crystal structure data for compounds **6**, **7** and **8**.

	**6**	**7**	**8**
CCDC	2330046	2330047	2330048
empirical formula	$C_{36}H_{47}NSn$	$C_{43}H_{61}N_3Sn{\bullet}0.25C_6H_{14}$	$C_{43}H_{61}N_3Se_4Sn$
formula weight	612.43	760.18	1054.47
colour	clear brown	clear colourless	clear orange
habit	prism	prism	block
cryst. dimens, mm	0.17 x 0.16 x 0.12	0.18 x 0.17 x 0.13	0.17 x 0.14 x 0.13
T, K	100(2)	100(2)	100(2)
crystal system	triclinic	triclinic	orthorhombic
space group	$P\bar{1}$	$P\bar{1}$	$P2_12_12_1$
a, Å	9.372(2)	10.222(2)	10.311(2)
b, Å	9.414(2)	20.121(3)	17.058(2)
c, Å	19.478(3)	21.673(3)	24.712(3)
α, deg	82.16(3)	70.79(2)	90
β, deg	85.49(2)	79.37(2)	90
γ, deg	65.67(2)	79.18(2)	90
V, Å^3	1550.6(6)	4098.3(13)	4346.5(11)
Z	2	4	4
D_{calc}, g•cm^{-3}	1.312	1.232	1.611
μ, mm^{-1}	0.848	0.656	3.970
radiation	Mo $K\alpha$ (λ = 0.71073)	Mo $K\alpha$ (λ = 0.71073)	Mo $K\alpha$ (λ = 0.71073)
θ range, deg	2.386 – 29.620	2.396 – 30.523	2.308 – 30.530
no. of rflns collected	40269	144864	257124
no. of indep. reflns.	8659	25017	13255
R(int)	0.0890	0.0501	0.1133
[I>2σ(I)]	R1 = 0.0445<br>wR2 = 0.1065	R1 = 0.0275<br>wR2 = 0.0643	R1 = 0.0326<br>wR2 = 0.0771
R indices (all data)	R1 = 0.0509<br>wR2 = 0.1238	R1 = 0.0359<br>wR2 = 0.0688	R1 = 0.0383<br>wR2 = 0.0810
Flack x parameter	-	-	0.489(8)
GOF on F^2	1.107	1.033	1.092
$\Delta\rho_{max}/\Delta\rho_{min,}$ e Å^{-3}	1.797 / -0.962	0.948 / -0.492	1.608 / -0.823

Table S11. Crystal structure data for compounds **9** and **10**.

	**9**	**10**
CCDC	2330049	2330050
empirical formula	$C_{79}H_{108}N_4Se_2Sn_2$, C_6H_{14}	$C_{86}H_{122}N_6O_2Sn_2$, $3(C_5H_{12})$
formula weight	1595.16	1725.70
colour	yellow	colourless
habit	block	block
cryst. dimens, mm	0.47 x 0.25 x 0.15	0.38 x 0.36 x 0.18
T, K	110(2)	100(2)
crystal system	triclinic	triclinic
space group	$P\bar{1}$	$P\bar{1}$
a, Å	13.873(2)	14.363(2)
b, Å	14.609(2)	15.397(2)
c, Å	20.054(3)	21.564(3)
α, deg	102.95(2)	97.15(2)
β, deg	94.97(2)	91.81(2)
γ, deg	101.92(2)	97.79(2)
V, $Å^3$	3837.7(10)	4682.4(11)
Z	2	2
D_{calc}, $g•cm^{-3}$	1.380	1.224
μ, mm^{-1}	0.692	0.584
radiation	In Kα (λ = 0.51340)	Mo Kα (λ = 0.71073)
θ range, deg	1.417 – 19.638	2.008 – 25.146
no. of rflns collected	509189	447810
no. of indep. reflns.	17863	16649
R(int)	0.0508	0.0647
[I>2σ(I)]	R1 = 0.0246<br>wR2 = 0.0616	R1 = 0.0519<br>wR2 = 0.1271
R indices (all data)	R1 = 0.0293<br>wR2 = 0.0642	R1 = 0.0554<br>wR2 = 0.1291
GOF on F^2	1.051	1.201
$\Delta\rho_{max}/\Delta\rho_{min,}$ e $Å^{-3}$	1.955 / -0.810	1.193 / -1.130

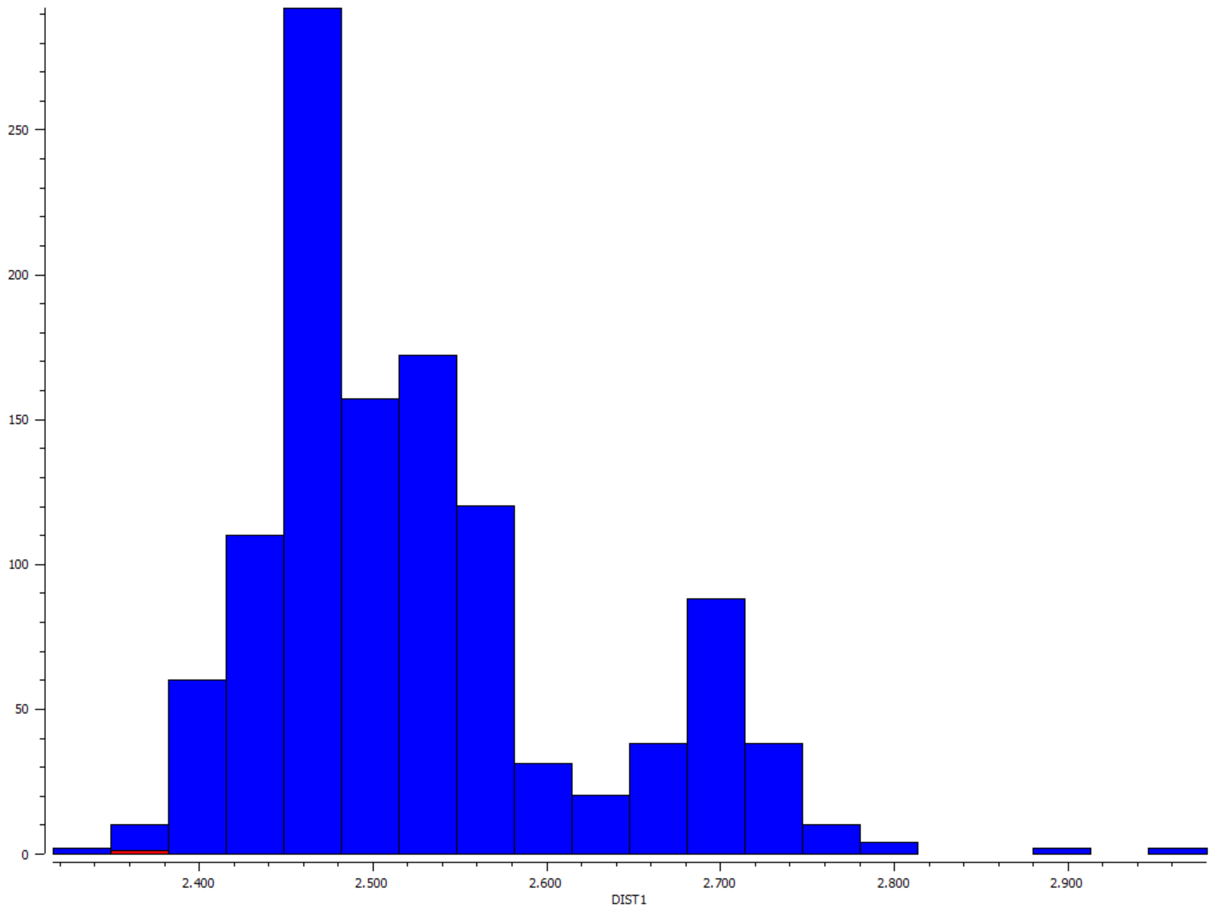

Figure S57. Histogram of all Sn–Se bond length with terminal Se reported in CSD (v5.44).[S11]

Computational Details

Geometry optimizations, frequency calculations and PCM solvent corrections were run with Gaussian 16 Revision A.03[S12] using the BP86[S13,S14] functional. For geometry optimisations, all atoms were described with def2-SVP basis sets of Ahlrichs and Weigand.[S15] Single point energy calculations were performed on the optimised geometries, at the BP86/def2-TZVP level of theory. Stationary points were fully characterized using analytical frequency calculations as either minima (all positive eigenvalues) or transition states (one negative eigenvalue). IRC calculations and subsequent geometry optimizations were used to confirm the minima linked by the transition states. Energies reported in the text are based on the gas-phase free energies and incorporate a correction for dispersion effects using Grimme's D3 parameter set with Becke-Johnson dampening[S16,S17] (i.e. BP86-D3BJ) as well as solvation (PCM approach) in benzene. Energies are given in atomic units (a.u.) unless otherwise stated. Natural Bond Orbital (NBO) and Natural Localised Molecular Orbital (NLMO) analysis was performed using NBO-7 using the single point calculations performed at the BP86/def2-TZVP level of theory.[S18]

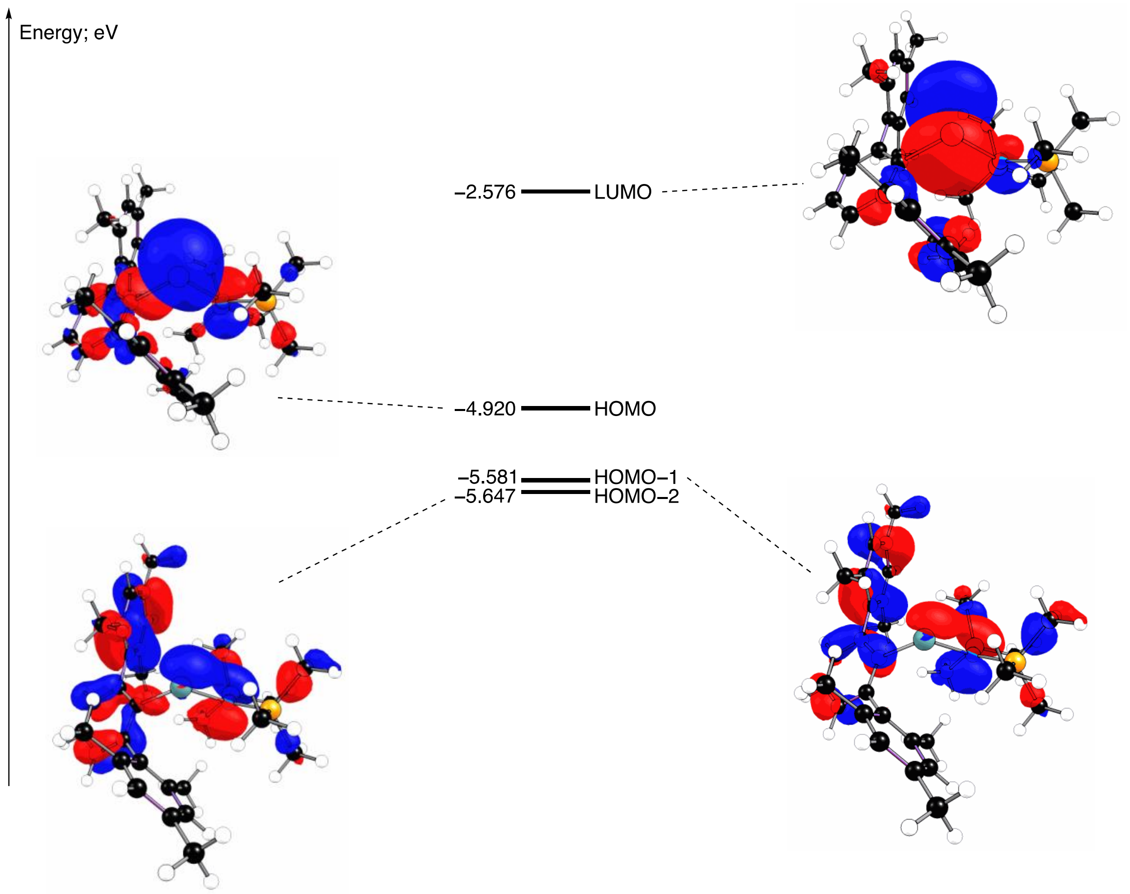

Figure S58. Molecular Orbital (BP86-D3BJ/def2-TZVP) diagram of **1a**.

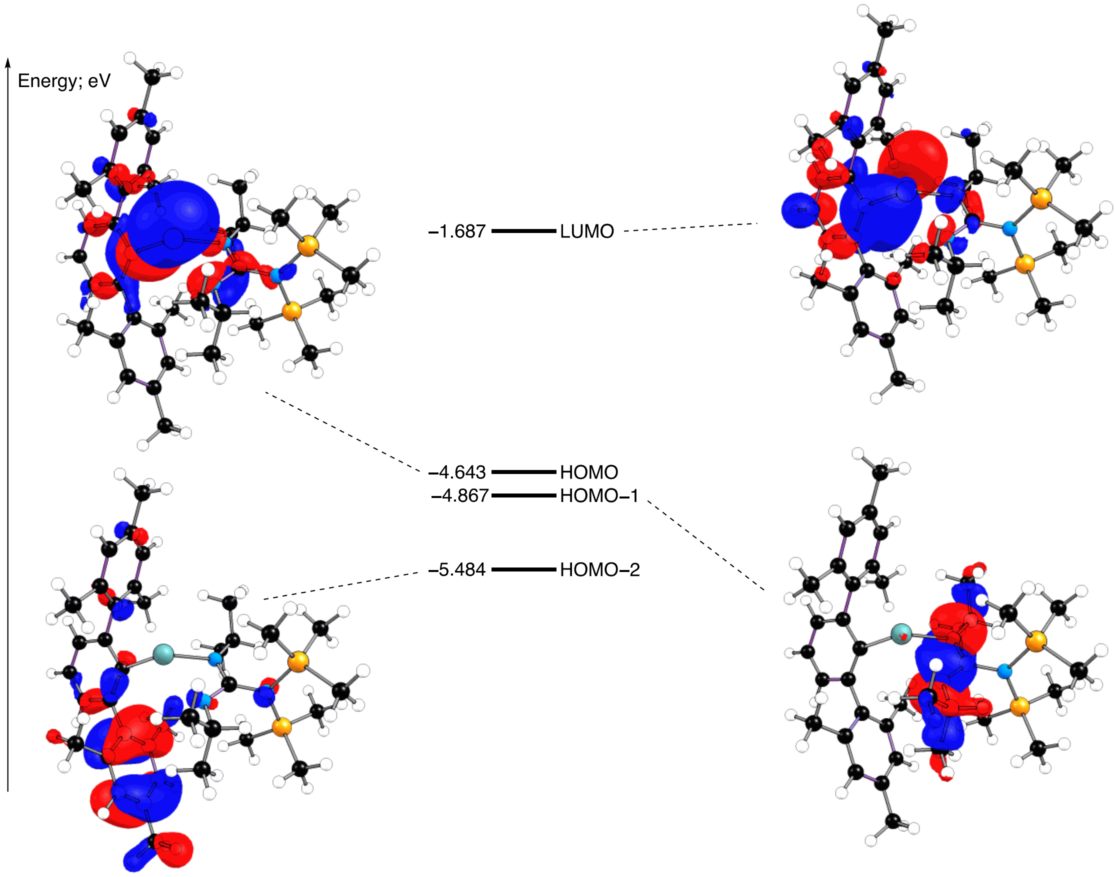

Figure S59. Molecular Orbital (BP86-D3BJ/def2-TZVP) diagram of **2a**.

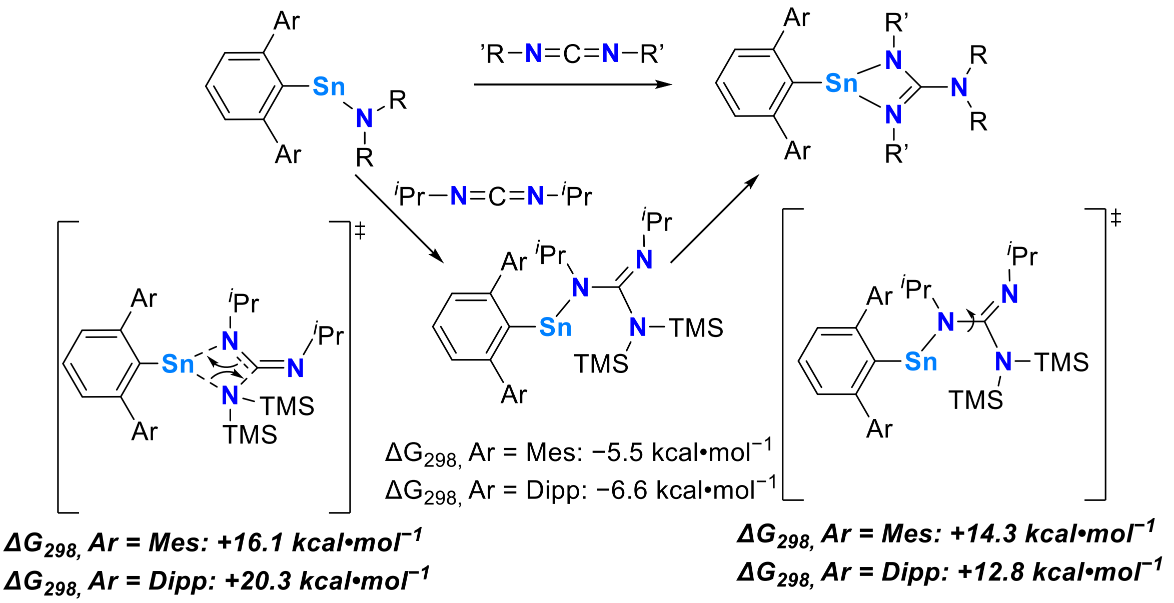

Figure S60. Calculated mechanism (BP86-D3BJ/def2-TZVP) for the formation of **2a**,**b**.

Table S12. Relative calculated Gibbs free energies (BP86-D3BJ/def2-TZVP; 298 K, kcal•mol^{-1}) for the formation of **2a**,**b** and **7**, varying the R substituent on the carbodiimide.

R	2a	2b	7
**^{i}Pr**	-14.5	-14.2	-6.7
**^{t}Bu**	+3.2	+6.3	+14.5
**TMS**	+5.1	+14.7	+10.1

DFT Optimised Geometries

iPrNCNiPr

SCF = -384.376673968
H(0 K) = -384.180072
H(298 K) = -384.167396
G(298 K) = -384.219070
SCF+D3BJ = -384.404560029
PCM SCF (Benzene) = -384.378212024
BS2 (def2-tzvp) = -384.799259737
Low Freq. = 21.5269 cm^{-1}, 26.6126 cm^{-1}

23
iPrNCNiPr

C	-0.000009011	-0.391312521	-0.000135881
N	-1.187978672	-0.486634656	0.316590349
C	-2.358490145	0.233402113	-0.197924227
N	1.187941783	-0.486364478	-0.316991575
C	2.358459219	0.233425943	0.197864260
C	-2.993333512	1.047253481	0.940472926
H	-2.045174255	0.938797911	-1.004318050
C	-3.345892636	-0.780196621	-0.797063542
C	2.993845375	1.047047689	-0.940379071
H	2.045045130	0.938934613	1.004120767
C	3.345439386	-0.780368455	0.797388975
H	-3.888991004	1.593675056	0.578745838
H	-3.301743775	0.376706814	1.769503574
H	-2.275768929	1.787848826	1.348579606
H	-4.246102450	-0.263320456	-1.189739689
H	-2.878977557	-1.346117916	-1.628383056
H	-3.667686353	-1.509193622	-0.024421216
H	4.245653321	-0.263675353	1.190301442
H	2.878136954	-1.346111407	1.628612041
H	3.667302425	-1.509499587	0.024901568
H	3.889490278	1.593315887	-0.578392532
H	3.302394050	0.376369609	-1.769251294
H	2.276568336	1.787753791	-1.348791066

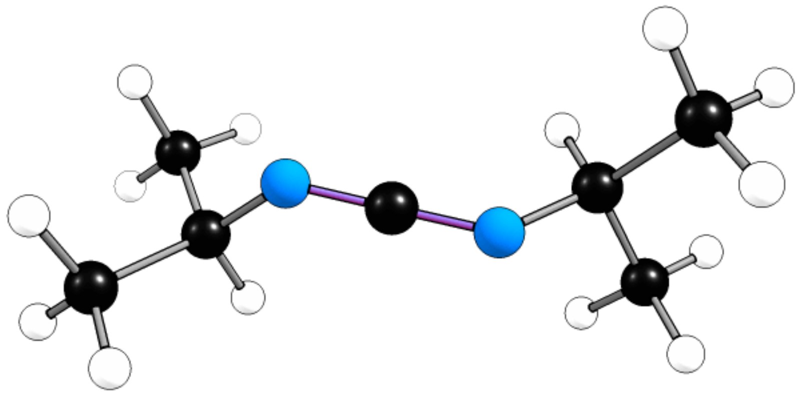

tBuNCNtBu
SCF = -462.945143232
H(0 K) = -462.695346
H(298 K) = -462.679966
G(298 K) = -462.737380
SCF+D3BJ = -462.987070923
PCM SCF (Benzene) = -462.946720734
BS2 (def2-tzvp) = -463.451461549
Low Freq. = 14.7925 cm^{-1}, 19.1238 cm^{-1}

29
tBuNCNtBu

C	-2.994725000	-1.260433000	0.662763000
C	-2.394453000	0.002126000	0.007749000
C	-3.359498000	0.545267000	-1.069274000
N	-1.159443000	-0.409489000	-0.685198000
C	0.000000000	-0.000208000	-0.583825000
N	1.159458000	0.408951000	-0.685545000
C	2.394451000	-0.002115000	0.007750000
C	3.359182000	-0.546971000	-1.068689000
C	2.995183000	1.261137000	0.661002000
C	-2.120429000	1.082187000	1.073616000
C	2.120276000	-1.080680000	1.075095000
H	-3.964810000	-1.027523000	1.149069000
H	-3.162153000	-2.049272000	-0.098244000
H	-2.307442000	-1.667844000	1.432603000
H	-4.338028000	0.813606000	-0.619295000
H	-2.937832000	1.449738000	-1.553846000
H	-3.530270000	-0.216923000	-1.856155000
H	4.337705000	-0.814975000	-0.618493000
H	2.937199000	-1.451981000	-1.551979000
H	3.530050000	0.214109000	-1.856623000
H	3.965250000	1.028588000	1.147516000
H	3.162764000	2.048903000	-0.101082000
H	2.308103000	1.669790000	1.430364000
H	3.060814000	-1.383970000	1.580288000
H	1.423209000	-0.701469000	1.851538000
H	1.669079000	-1.985904000	0.618082000
H	-3.060979000	1.385887000	1.578540000
H	-1.423141000	0.704213000	1.850464000
H	-1.669551000	1.986926000	0.615330000

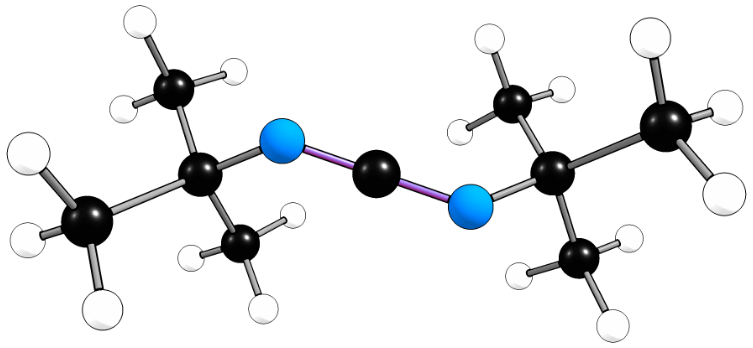

TMSNCNTMS
SCF = -965.781946949
H(0 K) = -965.551943
H(298 K) = -965.531679
G(298 K) = -965.604057
SCF+D3BJ = -965.826154742
PCM SCF (Benzene) = -965.782901283
BS2 (def2-tzvp) = -966.426339349
Low Freq. = 3.6898 cm^{-1}, 15.4470 cm^{-1}

29
TMSNCNTMS

C	3.554770000	0.911201000	-1.546410000
Si	2.956344000	0.000010000	0.000001000
C	3.553688000	-1.795221000	-0.016315000
N	1.218783000	0.000896000	0.000317000
C	-0.000009000	0.000997000	0.000755000
N	-1.218801000	0.001366000	0.001190000
Si	-2.956362000	-0.000022000	0.000060000
C	-3.553396000	-1.790432000	0.133555000
C	3.555344000	0.883093000	1.562451000
C	-3.553906000	0.778495000	-1.617610000
C	-3.556466000	1.010615000	1.482703000
H	4.664728000	0.935644000	-1.589414000
H	3.188426000	0.414312000	-2.469069000
H	3.189706000	1.959535000	-1.558111000
H	4.663618000	-1.845397000	-0.016874000
H	3.187721000	-2.345582000	0.875638000
H	3.187569000	-2.329331000	-0.918037000
H	-4.663315000	-1.840708000	0.136459000
H	-3.186568000	-2.397983000	-0.720099000
H	-3.187961000	-2.264194000	1.068660000
H	-4.663838000	0.799123000	-1.663308000
H	-3.188897000	1.822212000	-1.716780000
H	-3.186899000	0.206188000	-2.495210000
H	-4.666471000	1.037690000	1.522811000
H	-3.191091000	0.575451000	2.436405000
H	-3.191468000	2.057494000	1.425900000
H	4.665321000	0.907057000	1.605304000
H	3.189994000	1.930934000	1.593431000
H	3.189648000	0.369389000	2.476116000

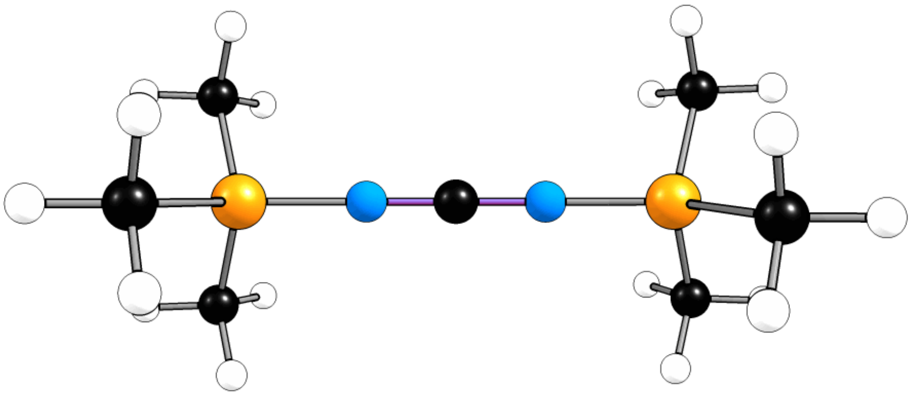

1a
SCF = -2016.34775970
H(0 K) = -2015.724718
H(298 K) = -2015.678988
G(298 K) = -2015.805284
SCF+D3BJ = -2016.55620561
PCM SCF (Benzene) = -2016.34984705
BS2 (def2-tzvp) = -2017.87641272
Low Freq. = 11.5799 cm^{-1}, 19.7113 cm^{-1}

77
1a

C	-2.500557181	-2.632297190	-0.657664046
C	-1.984735143	-1.981148141	0.509842037
C	-2.852872206	-1.167791084	1.296108094
C	-4.192239302	-0.996887073	0.886834065
C	-4.708808337	-1.607913115	-0.271094020
C	-3.842689276	-2.424184177	-1.026761076
C	-0.559304040	-2.264627161	0.929172065
C	0.503721036	-1.468485107	0.420539030
C	1.847513132	-1.873316132	0.668076048
C	2.089358152	-3.007476218	1.481432105
C	1.031247076	-3.743988267	2.037364149
C	-0.294503021	-3.383662244	1.751083125
C	3.027955219	-1.171701084	0.052317004
C	3.229754233	-1.222053088	-1.359983096
C	4.334377312	-0.551930040	-1.925650138
C	5.266590379	0.153368011	-1.142366080
C	5.083715368	0.141310010	0.252986018
C	3.996924287	-0.514218037	0.866613064
C	2.333992170	-2.037916144	-2.272095164
H	1.410796103	-1.490434109	-2.571932185
C	3.918678282	-0.519416038	2.379656171
H	4.430201321	0.367620027	2.805326202
Sn	-0.368846026	0.069705005	-1.029439073
N	-0.690035052	1.879362136	0.101738007
Si	-1.990573143	2.888088206	-0.590816043
C	-3.070196222	1.884023134	-1.798258129
C	-2.359420168	-0.472676034	2.540996181
H	-3.202276233	-0.172551013	3.194980228
C	-1.631974116	-3.559565255	-1.478993106

H	-2.196851160	-3.986281289	-2.331029169
Si	0.536836037	2.531365181	1.222625086
C	0.107442008	4.256246307	1.911248140
H	-1.662594122	-1.105848082	3.125006226
H	-1.239358089	-4.396905319	-0.864955062
H	4.421325319	-1.416264102	2.804158202
H	2.864477206	-2.296273167	-3.210623231
C	0.763331054	1.425465102	2.747532199
C	2.184052156	2.703595194	0.292071021
C	-3.173382229	3.514455254	0.760226053
C	-1.316191096	4.366602312	-1.583839116
H	-1.133126079	-3.980326288	2.148051154
H	1.242779088	-4.619263334	2.672620195
H	3.128758225	-3.326467238	1.663175121
H	-4.857297352	-0.371973027	1.506860109
C	-6.141223421	-1.387706099	-0.702577050
H	-4.228009303	-2.929787209	-1.928452136
H	-1.801799129	0.447116032	2.260541161
H	-0.741267056	-3.035910219	-1.887189138
H	5.815261401	0.662179050	0.894571065
C	6.417182477	0.903649064	-1.775275130
H	4.480846324	-0.603969045	-3.018336216
H	2.877080208	-0.532583039	2.750553197
H	2.001878142	-2.977751216	-1.787397131
H	1.675228123	1.727729126	3.305209235
H	-0.099412007	1.524438108	3.439001246
H	0.861494063	0.355807025	2.473679178
H	0.970770070	4.598078331	2.523162183
H	-0.064779005	5.024104361	1.129906083
H	-0.778960055	4.235308303	2.578069185
H	2.981741212	3.120304227	0.943931068
H	2.549089185	1.725037122	-0.086955006
H	2.066552148	3.383592245	-0.578496039
H	-4.030781291	4.045796293	0.292930021
H	-3.581058260	2.663957193	1.346262096
H	-2.690055194	4.216683301	1.468077106
H	-2.149488156	4.953407355	-2.027512144
H	-0.711139053	5.061486362	-0.966019069
H	-0.672715048	4.012020291	-2.417169176
H	-3.934629282	2.519386180	-2.092759148
H	-2.540939183	1.597406115	-2.730685195
H	-3.481261252	0.959886069	-1.337345094
H	-6.583245494	-2.307841167	-1.137099084
H	-6.779257510	-1.063401076	0.143953010
H	-6.204719452	-0.598430041	-1.483882106
H	6.698481461	0.470939034	-2.756896196
H	6.150163447	1.969201139	-1.952201140
H	7.316571547	0.899123063	-1.125858084

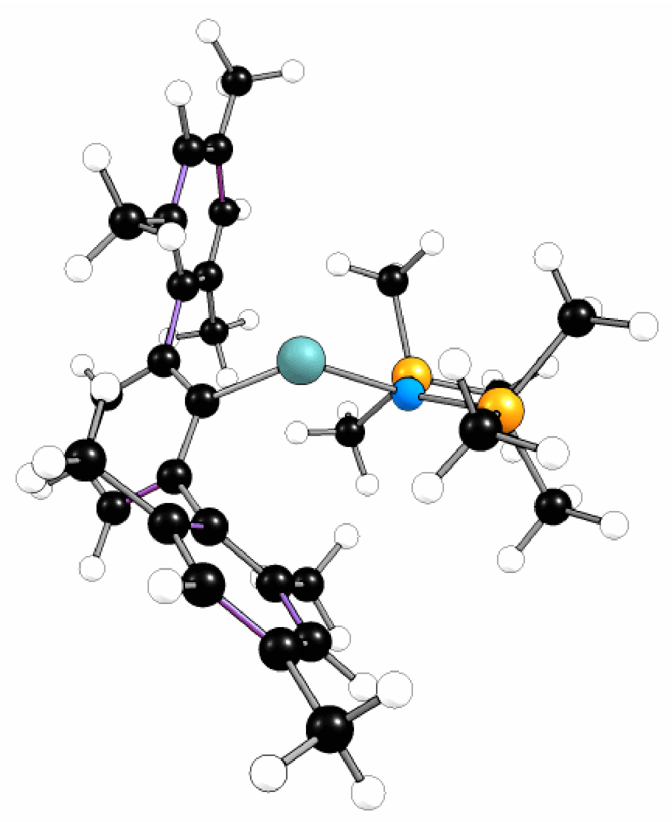

TS 1a–INTa
SCF = -2400.70197438
H(0 K) = -2399.881182
H(298 K) = -2399.823397
G(298 K) = -2399.975315
SCF+D3BJ = -2400.97165412
PCM SCF (Benzene) = -2400.70455406
BS2 (def2-tzvp) = -2402.64435312
Low Freq. = -181.1610 cm^{-1}, 10.1350 cm^{-1}

100
TS 1a–INTa

C	2.425882000	2.949132000	-1.465412000
C	1.519602000	3.007907000	-0.371624000
C	2.023141000	3.228649000	0.942232000
C	3.414202000	3.330167000	1.140399000
C	4.332059000	3.226990000	0.075365000
C	3.812257000	3.041251000	-1.218576000
C	0.032525000	3.044971000	-0.606764000
C	-0.874508000	1.987926000	-0.288013000
C	-2.274946000	2.266968000	-0.415669000
C	-2.727826000	3.509953000	-0.919781000
C	-1.820193000	4.512961000	-1.280400000
C	-0.450338000	4.281968000	-1.103734000
C	-3.369166000	1.323374000	0.035227000
C	-4.166234000	0.622923000	-0.915278000
C	-5.232663000	-0.183046000	-0.463536000
C	-5.562397000	-0.297636000	0.899384000

C	-4.804329000	0.452802000	1.819578000
C	-3.729466000	1.268855000	1.417114000
C	-3.941375000	0.777068000	-2.403956000
C	-2.990832000	2.083714000	2.453922000
C	-6.686795000	-1.196557000	1.363556000
Sn	-0.607854000	-0.106427000	0.773563000
N	1.592145000	-0.567699000	1.284556000
C	1.965592000	-1.656832000	0.688956000
N	2.862435000	-2.474630000	0.482807000
C	3.049179000	-3.853834000	0.098953000
C	2.909170000	-4.791646000	1.312422000
C	1.082948000	3.447917000	2.109394000
C	5.822876000	3.305536000	0.321270000
C	1.928599000	2.847923000	-2.891955000
N	0.176911000	-1.834599000	-0.634660000
Si	0.764717000	-1.458143000	-2.288935000
C	1.044324000	-2.995309000	-3.384299000
Si	-0.917917000	-3.223176000	-0.306663000
C	-0.315798000	-4.936362000	-0.892623000
C	-1.198054000	-3.441556000	1.565925000
C	-2.604920000	-2.952400000	-1.142212000
C	2.282670000	-0.164304000	2.547907000
C	1.900213000	-1.094056000	3.711198000
C	3.801513000	-0.040878000	2.371887000
C	4.410395000	-4.023926000	-0.595323000
C	2.426232000	-0.552522000	-2.219652000
C	-0.476648000	-0.380150000	-3.237467000
H	0.274054000	5.081263000	-1.333695000
H	-2.178660000	5.479773000	-1.669297000
H	-3.812780000	3.688977000	-1.005269000
H	3.791987000	3.514232000	2.161050000
H	4.507140000	2.979870000	-2.073681000
H	-5.067178000	0.415641000	2.890755000
H	-5.833494000	-0.730536000	-1.209942000
H	2.245518000	-4.102602000	-0.632552000
H	3.685145000	-4.564939000	2.073313000
H	3.030551000	-5.850033000	1.001471000
H	1.914333000	-4.684061000	1.789734000
H	4.490781000	-3.363860000	-1.482027000
H	4.546344000	-5.072788000	-0.929597000
H	5.238845000	-3.769168000	0.098188000
H	1.880750000	0.847861000	2.765521000
H	0.799167000	-1.144281000	3.837880000
H	2.341640000	-0.732099000	4.663381000
H	2.276699000	-2.123660000	3.533010000
H	4.263118000	-1.026153000	2.158325000
H	4.257646000	0.361540000	3.300548000
H	4.043145000	0.648997000	1.539531000
H	-0.081017000	-0.131354000	-4.245852000
H	-0.673008000	0.571410000	-2.700777000
H	-1.445697000	-0.902140000	-3.375137000
H	1.745223000	-3.731586000	-2.939374000

H	1.494746000	-2.648171000	-4.339951000
H	0.102363000	-3.523428000	-3.639749000
H	2.405650000	0.316493000	-1.531917000
H	2.688484000	-0.181166000	-3.232888000
H	3.238699000	-1.225272000	-1.879223000
H	-3.306351000	-3.768410000	-0.863392000
H	-2.514712000	-2.954607000	-2.249318000
H	-3.073162000	-1.991992000	-0.840602000
H	-0.058984000	-4.988090000	-1.967660000
H	-1.153796000	-5.646405000	-0.717024000
H	0.549238000	-5.312546000	-0.309482000
H	-1.669281000	-4.432801000	1.744039000
H	-1.863629000	-2.667855000	1.999046000
H	-0.244160000	-3.418405000	2.134301000
H	-4.342636000	-0.092504000	-2.962803000
H	-4.460228000	1.682251000	-2.789935000
H	-2.872084000	0.896165000	-2.658329000
H	-3.545953000	2.108301000	3.412688000
H	-1.985767000	1.649394000	2.666758000
H	-2.818825000	3.125505000	2.114956000
H	-7.431016000	-1.370950000	0.560173000
H	-6.301016000	-2.192818000	1.674431000
H	-7.217967000	-0.768970000	2.238853000
H	2.743254000	2.542514000	-3.578544000
H	1.095435000	2.125472000	-2.992651000
H	1.539733000	3.825359000	-3.251622000
H	1.641558000	3.575441000	3.058234000
H	0.460149000	4.355185000	1.953885000
H	0.363294000	2.612956000	2.239315000
H	6.224810000	2.332012000	0.680463000
H	6.377024000	3.567040000	-0.602769000
H	6.070612000	4.060699000	1.096005000

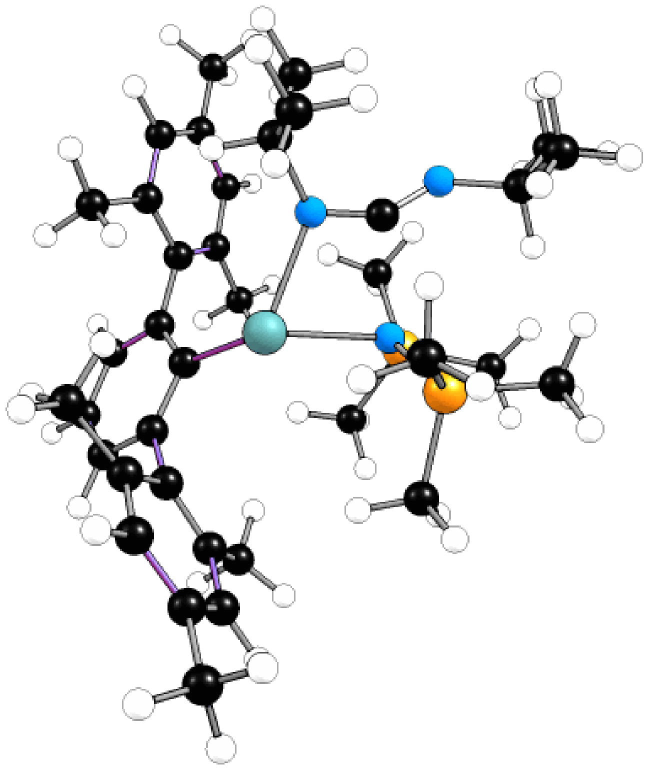

INTa
SCF = -2400.73791662
H(0 K) = -2399.915285
H(298 K) = -2399.857386
G(298 K) = -2400.009990
SCF+D3BJ = -2401.00951221
PCM SCF (Benzene) = -2400.74055992
BS2 (def2-tzvp) = -2402.67803209
Low Freq. = 11.8369 cm^{-1}, 16.1321 cm^{-1}

100
INTa

Sn	0.363105000	-0.161946000	-0.564718000
Si	-2.704599000	2.265256000	-0.758985000
Si	-3.301546000	-0.770531000	-1.481864000
N	-3.090168000	-0.076987000	1.951836000
N	-0.884280000	0.012195000	1.203260000
N	-2.638753000	0.498944000	-0.382889000
C	-0.507875000	-0.216691000	2.620666000
H	-1.281364000	0.310791000	3.219909000
C	-2.268145000	0.128347000	0.970788000
C	2.600484000	-0.325307000	0.011231000
C	2.285469000	-2.842877000	-0.156328000
C	2.540483000	2.783565000	-1.232018000
C	1.767923000	-3.237714000	-1.426100000
C	3.480094000	0.795717000	0.034514000
C	2.988824000	3.017826000	1.176271000
C	3.170520000	-1.629337000	-0.046632000
C	2.014947000	-3.642543000	0.991503000

C	-4.526694000	0.103013000	1.880223000
H	-4.852053000	0.463439000	0.872761000
C	2.967271000	2.211125000	0.002474000
C	2.500401000	4.338871000	1.112069000
H	2.507844000	4.950469000	2.030874000
C	0.600157000	-5.136703000	-0.369189000
C	0.924285000	-4.366946000	-1.502448000
H	0.525905000	-4.664225000	-2.487546000
C	4.881430000	0.597642000	0.056619000
H	5.548528000	1.475740000	0.077937000
C	2.023882000	4.906148000	-0.085460000
C	1.172762000	-4.764670000	0.863760000
H	0.960557000	-5.371933000	1.760287000
C	2.072560000	4.114408000	-1.248811000
H	1.753106000	4.551038000	-2.210441000
C	2.647508000	2.016634000	-2.535117000
H	3.646815000	1.547976000	-2.648359000
H	2.471982000	2.682837000	-3.402964000
H	1.905787000	1.189133000	-2.604005000
C	3.591490000	2.516420000	2.472078000
H	3.453117000	1.427659000	2.608812000
H	3.157879000	3.041061000	3.347356000
H	4.688359000	2.702145000	2.491304000
C	4.572968000	-1.806009000	-0.026387000
H	4.992280000	-2.825451000	-0.069399000
C	0.855625000	0.390127000	2.950371000
H	1.677928000	-0.106383000	2.394235000
H	1.064999000	0.289840000	4.035846000
H	0.878849000	1.468900000	2.694194000
C	5.427626000	-0.694793000	0.033351000
H	6.520598000	-0.834808000	0.046011000
C	2.661640000	-3.330870000	2.324282000
H	3.746683000	-3.571296000	2.306171000
H	2.200618000	-3.918677000	3.142600000
H	2.587040000	-2.255394000	2.581165000
C	-0.605473000	-1.705145000	2.996424000
H	-1.646687000	-2.056792000	2.859027000
H	-0.326456000	-1.862188000	4.060265000
H	0.066124000	-2.322804000	2.365393000
C	-4.965565000	1.148334000	2.925839000
H	-4.646754000	0.833103000	3.941520000
H	-6.069347000	1.271387000	2.924755000
H	-4.511578000	2.139558000	2.722840000
C	2.189833000	-2.533113000	-2.699531000
H	2.053605000	-1.432976000	-2.649566000
H	1.620810000	-2.909037000	-3.572762000
H	3.271048000	-2.697261000	-2.898140000
C	-2.259433000	3.238772000	0.799268000
H	-1.236280000	2.989724000	1.146912000
H	-2.959164000	3.043530000	1.636034000
H	-2.289161000	4.325316000	0.570638000
C	-0.339700000	-6.317403000	-0.466916000

H	-1.385286000	-6.012862000	-0.239586000
H	-0.070708000	-7.116081000	0.254312000
H	-0.341557000	-6.757315000	-1.484854000
C	1.477625000	6.316394000	-0.117843000
H	0.407627000	6.338475000	0.186142000
H	1.538170000	6.756253000	-1.133969000
H	2.024485000	6.983770000	0.579649000
C	-5.223307000	-1.243148000	2.164250000
H	-4.926888000	-2.017441000	1.427542000
H	-6.328288000	-1.136823000	2.128637000
H	-4.939269000	-1.614576000	3.171297000
C	-2.665228000	-2.460480000	-0.910988000
H	-1.580193000	-2.594920000	-1.103680000
H	-3.205273000	-3.258997000	-1.463397000
H	-2.839845000	-2.620081000	0.172852000
C	-1.491404000	2.778258000	-2.125472000
H	-0.436100000	2.693466000	-1.790951000
H	-1.669044000	3.846209000	-2.379638000
H	-1.606242000	2.186329000	-3.054983000
C	-4.445744000	2.755297000	-1.340867000
H	-4.719147000	2.279313000	-2.305866000
H	-4.486949000	3.855125000	-1.495731000
H	-5.222420000	2.488526000	-0.594560000
C	-2.722933000	-0.432964000	-3.257686000
H	-3.165030000	0.493901000	-3.679170000
H	-3.047892000	-1.274054000	-3.907373000
H	-1.618555000	-0.354668000	-3.323929000
C	-5.200673000	-0.848785000	-1.553555000
H	-5.653686000	-1.203511000	-0.606716000
H	-5.494962000	-1.563351000	-2.353440000
H	-5.653141000	0.132587000	-1.801690000

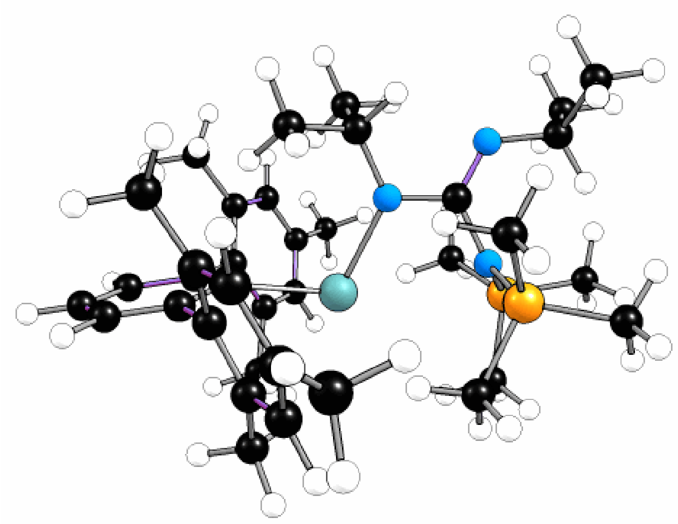

TS INTa–2a

SCF = -2400.71293146

H(0 K) = -2399.890411

H(298 K) = -2399.833510

G(298 K) = -2399.982466

SCF+D3BJ = -2400.97848954
PCM SCF (Benzene) = -2400.71590719
BS2 (def2-tzvp) = -2402.65466549
Low Freq. = -34.9877 cm^{-1}, 11.1746 cm^{-1}

100
TS INTa–2a

C	-3.685627000	-1.706366000	-1.355706000
C	-3.749941000	-1.218070000	-0.018047000
C	-3.938458000	-2.136979000	1.054435000
C	-3.983452000	-3.517430000	0.777676000
C	-3.865041000	-4.024581000	-0.531292000
C	-3.732274000	-3.098370000	-1.582056000
C	-3.711433000	0.262035000	0.251339000
C	-2.505280000	1.017552000	0.171264000
C	-2.577880000	2.430962000	0.342694000
C	-3.810140000	3.050754000	0.655815000
C	-4.984024000	2.291650000	0.772361000
C	-4.933888000	0.906663000	0.558418000
Sn	-0.585043000	0.145400000	-0.750571000
N	0.709919000	-0.698077000	0.724449000
C	0.364758000	-1.072795000	2.122915000
C	-0.489635000	-2.349643000	2.196851000
C	-1.377301000	3.320369000	0.144407000
C	-0.677497000	3.863874000	1.258062000
C	0.417989000	4.723135000	1.030049000
C	0.825210000	5.091782000	-0.265165000
C	0.086175000	4.586792000	-1.353538000
C	-1.008877000	3.718312000	-1.177382000
C	-1.132704000	3.607412000	2.678847000
C	-1.819001000	3.283888000	-2.381071000
C	-4.117460000	-1.654384000	2.479112000
C	-3.639319000	-0.765177000	-2.543654000
C	1.839468000	-1.428967000	0.192332000
N	1.663603000	-2.670408000	-0.105701000
C	2.721275000	-3.533614000	-0.615180000
C	3.116443000	-4.550984000	0.472632000
C	2.196891000	-4.265112000	-1.864540000
N	3.090214000	-0.707515000	0.039471000
Si	3.386075000	0.217733000	-1.483753000
C	2.514496000	-0.608155000	-2.947646000
C	2.837896000	2.025576000	-1.327272000
C	5.232522000	0.230873000	-1.939394000
Si	4.347194000	-0.683317000	1.332556000
C	3.740229000	-1.375277000	2.991337000
C	4.896570000	1.103881000	1.672000000
C	5.869670000	-1.727033000	0.873555000
C	-0.277042000	0.103149000	2.862413000
H	-3.843732000	4.144848000	0.791221000
H	-5.941754000	2.782534000	1.009401000
H	-5.858291000	0.307634000	0.611772000
H	0.960831000	5.130725000	1.900312000

C	2.020349000	5.990939000	-0.488623000
H	0.360501000	4.890482000	-2.378268000
H	-4.115360000	-4.221257000	1.617368000
C	-3.868806000	-5.514059000	-0.793736000
H	-3.682319000	-3.467091000	-2.620953000
H	3.629929000	-2.944283000	-0.892493000
H	1.303822000	-4.872461000	-1.608165000
H	2.975049000	-4.938555000	-2.281581000
H	1.898950000	-3.547751000	-2.654963000
H	3.521953000	-4.047606000	1.373585000
H	3.891745000	-5.248491000	0.091907000
H	2.233839000	-5.147452000	0.785494000
H	1.322772000	-1.294100000	2.636767000
H	0.391904000	0.987133000	2.854475000
H	-0.492027000	-0.167573000	3.917355000
H	-1.235095000	0.393906000	2.381744000
H	-1.473228000	-2.186529000	1.711203000
H	-0.662497000	-2.652377000	3.252431000
H	0.014231000	-3.173985000	1.656680000
H	5.634424000	1.111887000	2.503120000
H	4.029884000	1.725294000	1.980295000
H	5.375983000	1.596585000	0.802133000
H	5.603618000	-2.799323000	0.767606000
H	6.624942000	-1.649308000	1.685818000
H	6.355946000	-1.404569000	-0.067223000
H	3.055907000	-0.680866000	3.519009000
H	4.637080000	-1.509233000	3.634428000
H	3.243262000	-2.363016000	2.908523000
H	1.754499000	2.125791000	-1.109768000
H	3.039577000	2.572378000	-2.273690000
H	3.386451000	2.539190000	-0.511336000
H	5.892178000	0.707850000	-1.187359000
H	5.331730000	0.817126000	-2.878971000
H	5.615785000	-0.789989000	-2.143284000
H	1.426693000	-0.750318000	-2.787858000
H	2.967173000	-1.595295000	-3.173882000
H	2.638842000	0.036677000	-3.844336000
H	-3.887710000	-1.301385000	-3.481173000
H	-4.348725000	0.078754000	-2.422959000
H	-2.632603000	-0.311946000	-2.690669000
H	-4.537445000	-6.052671000	-0.091066000
H	-4.194664000	-5.747716000	-1.827639000
H	-2.850440000	-5.943557000	-0.666122000
H	-3.994043000	-2.486743000	3.200404000
H	-3.395195000	-0.855422000	2.741088000
H	-5.129849000	-1.223586000	2.636950000
H	-1.432234000	3.747978000	-3.309879000
H	-1.803865000	2.182066000	-2.525708000
H	-2.887189000	3.565321000	-2.268909000
H	1.845839000	6.704212000	-1.320695000
H	2.270323000	6.575565000	0.419631000
H	2.920797000	5.395517000	-0.757347000

H	-0.292294000	3.703658000	3.395807000
H	-1.905711000	4.348318000	2.981595000
H	-1.589126000	2.607619000	2.800666000

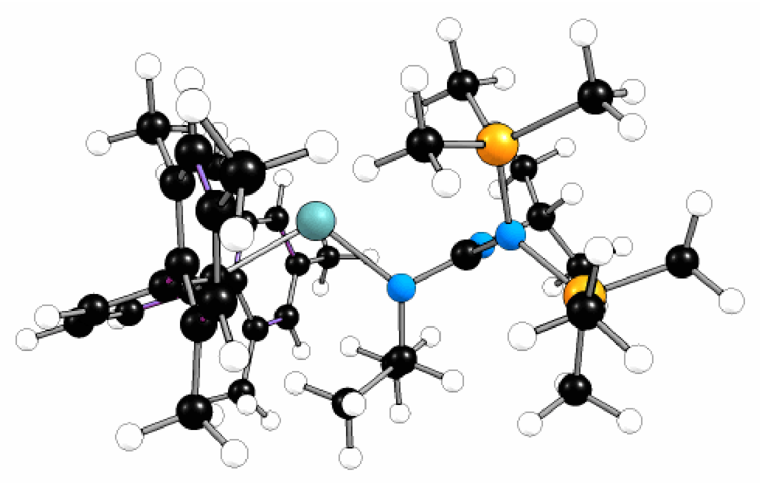

2a
SCF = -2400.76416401
H(0 K) = -2399.940719
H(298 K) = -2399.883129
G(298 K) = -2400.033665
SCF+D3BJ = -2401.02768116
PCM SCF (Benzene) = -2400.76697199
BS2 (def2-tzvp) = -2402.70281565
Low Freq. = 12.5900 cm^{-1}, 19.5643 cm^{-1}

100
2a

Sn	-0.931698067	-0.284007020	-0.852115063
Si	4.169621298	-0.822024060	1.056054075
Si	3.033610219	-3.186150228	-0.727287050
N	1.365156100	0.069277005	-0.947800068
N	0.498532036	-1.228821090	0.662923047
N	2.850390206	-1.582470114	0.086308006
C	0.446270032	-2.189470159	1.757374127
H	1.451488103	-2.662376191	1.840280131
C	1.599065118	-0.905242067	-0.047831003
C	-2.100354149	1.386560102	0.251672018
C	-0.410601029	3.365435242	0.313692022
C	-4.387937315	-0.761335055	-1.133794081
C	-0.166411012	4.109153294	-0.873820062
C	-3.454510246	0.981822069	0.475335034
C	-4.073122292	-1.363559096	1.231432088
C	-1.762634128	2.740863195	0.537431038
C	0.550595042	3.374568244	1.359410097
C	2.276857161	0.419134030	-2.036049149
H	3.104960225	-0.328865024	-2.052781146
C	-3.942269284	-0.415977030	0.176871013
C	-4.568710327	-2.652508189	0.943651069
H	-4.658360335	-3.382378244	1.766687128

C	1.994917143	4.872131349	0.035729003
C	1.028958072	4.847696351	-0.989771073
H	1.204954085	5.430716391	-1.910404139
C	-4.411731320	1.884667138	0.993000071
H	-5.446368395	1.537854111	1.154806085
C	-4.965995359	-3.028185216	-0.353517025
C	1.735999124	4.121896295	1.198899086
H	2.470957180	4.133117297	2.022115144
C	-4.880072352	-2.059118149	-1.372850099
H	-5.215650374	-2.318001169	-2.391760174
C	-4.345922313	0.246267018	-2.260002162
H	-4.793308345	1.216030088	-1.959762141
H	-4.880983352	-0.129031010	-3.154830227
H	-3.297501240	0.465373034	-2.565954186
C	-3.750848267	-0.986437071	2.661567190
H	-2.856183205	-0.336388024	2.728635194
H	-3.588175260	-1.884177135	3.291175238
H	-4.585212328	-0.408502029	3.116387225
C	-2.739414196	3.626480263	1.060423078
H	-2.448024178	4.667288333	1.281155091
C	-0.585434040	-3.294903239	1.468144105
H	-1.605290115	-2.867426205	1.362026096
H	-0.613008043	-4.039869291	2.291105163
H	-0.350527025	-3.829279273	0.525046038
C	-4.055699290	3.206520229	1.292239094
H	-4.802191347	3.909181281	1.696762123
C	0.285853021	2.629384191	2.648532189
H	-0.620974044	3.017583218	3.159532226
H	1.140036081	2.717065194	3.348962240
H	0.098545007	1.552741110	2.457733178
C	0.147815011	-1.497014105	3.101194224
H	0.917533066	-0.735770053	3.338252238
H	0.126115009	-2.234285159	3.931561282
H	-0.835843058	-0.985800069	3.071584222
C	2.894833210	1.811459133	-1.820775129
H	2.106107152	2.590140184	-1.792162129
H	3.605422258	2.059723147	-2.637975188
H	3.441225246	1.866896133	-0.858742061
C	-1.187099084	4.143049298	-1.991967144
H	-1.460070104	3.121460225	-2.330682166
H	-0.807328057	4.707230341	-2.867162204
H	-2.136314156	4.617352332	-1.663320121
C	3.441880246	0.612000045	2.044437148
H	2.907904211	1.338351097	1.398112100
H	4.259863308	1.152149080	2.566789185
H	2.726692198	0.254616018	2.812530204
C	3.278911238	5.656650416	-0.122974009
H	4.039419289	5.074706364	-0.689859047
H	3.727565267	5.911277438	0.858762064
H	3.115047227	6.601656471	-0.681105048
C	-5.460189394	-4.427002317	-0.649361049
H	-4.644608336	-5.061234363	-1.062392078

H	-6.276161450	-4.422175316	-1.401338102
H	-5.836536443	-4.931170353	0.263739019
C	1.555054112	0.364574026	-3.395744245
H	1.112455082	-0.634170048	-3.586565259
H	2.257106160	0.599244042	-4.223239305
H	0.733513053	1.112316078	-3.432507247
C	1.730440122	-3.331959241	-2.089555149
H	0.705802052	-3.115875225	-1.721957125
H	1.733806123	-4.366914314	-2.493018177
H	1.942109138	-2.640780193	-2.930511214
C	4.885961354	-2.105445151	2.258676160
H	4.105877295	-2.513378182	2.934565209
H	5.662050406	-1.622517115	2.890926210
H	5.372379362	-2.958187215	1.740895124
C	5.601541421	-0.178180013	-0.011715001
H	6.116290441	-0.990700070	-0.562343041
H	6.354793432	0.308441022	0.645536045
H	5.265358380	0.576014039	-0.751462052
C	2.854401206	-4.658638333	0.459670033
H	3.542568256	-4.583795329	1.326070094
H	3.101594223	-5.596305424	-0.084261006
H	1.822510132	-4.763414344	0.851005061
C	4.754677342	-3.313316236	-1.517559110
H	4.944468355	-2.488495178	-2.234761159
H	4.816192345	-4.270458309	-2.079242151
H	5.576576426	-3.320020240	-0.772235054

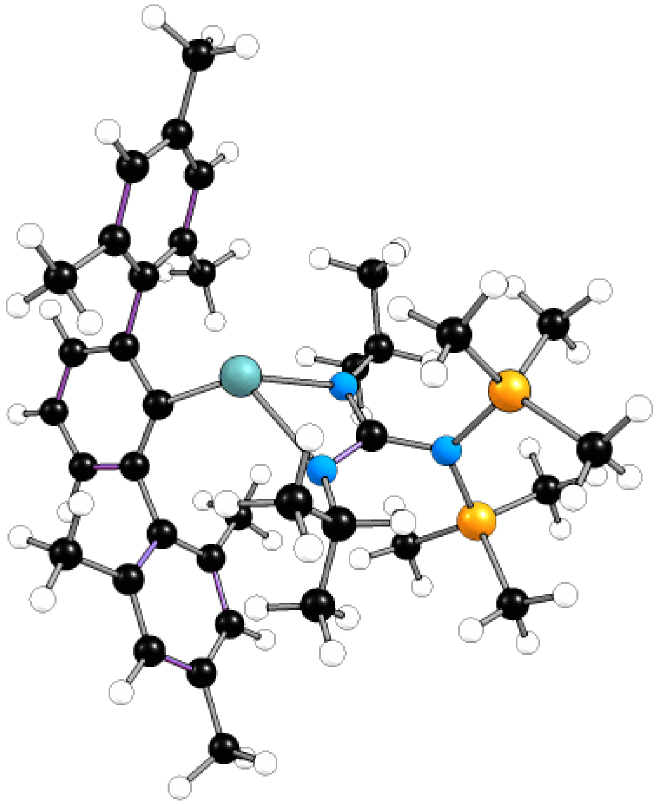

2a_tBu
SCF = -2479.29836599
H(0 K) = -2478.421083
H(298 K) = -2478.361108
G(298 K) = -2478.514741
SCF+D3BJ = -2479.58563125
PCM SCF (Benzene) = -2479.30107605
BS2 (def2-tzvp) = -2481.32029759
Low Freq. = 9.4848 cm^{-1}, 20.0863 cm^{-1}

106
2a_tBu

C	-0.493742000	4.217126000	-0.732637000
C	-0.686487000	3.350858000	0.380243000
C	0.272724000	3.335863000	1.429346000
C	1.397826000	4.181997000	1.347521000
C	1.605231000	5.050240000	0.257459000
C	0.647843000	5.044750000	-0.774830000
C	-2.010759000	2.647498000	0.539698000
C	-2.284822000	1.279708000	0.238891000
C	-3.641240000	0.837929000	0.387267000
C	-4.653077000	1.713589000	0.845858000
C	-4.355800000	3.042134000	1.172378000
C	-3.042234000	3.499374000	1.014054000
C	-4.093900000	-0.568149000	0.067498000
C	-4.372884000	-0.946733000	-1.280257000
C	-4.830384000	-2.250352000	-1.549319000
C	-5.040221000	-3.197464000	-0.526726000
C	-4.818794000	-2.786494000	0.800250000
C	-4.373096000	-1.486059000	1.119942000
C	-4.193518000	0.032474000	-2.418098000
H	-4.657363000	-0.347197000	-3.350179000
C	-4.280762000	-1.077171000	2.576158000
H	-4.096801000	-1.953543000	3.230080000
Sn	-1.000434000	-0.451040000	-0.683345000
N	0.618665000	-1.113505000	0.800512000
C	0.457604000	-2.072045000	1.911313000
C	1.696753000	-2.209208000	2.820441000
C	0.072903000	2.453508000	2.640180000
H	0.860895000	2.626703000	3.399881000
C	-1.517400000	4.298151000	-1.846372000
H	-1.109991000	4.833829000	-2.727238000
N	1.214030000	0.045350000	-1.002057000
C	1.870390000	0.724938000	-2.135820000
C	2.897212000	-0.129059000	-2.910643000
C	1.639700000	-0.802727000	-0.039351000
N	2.967711000	-1.354925000	0.044835000
Si	3.300311000	-2.979425000	-0.695485000
C	4.925277000	-2.967460000	-1.683552000
Si	4.282233000	-0.432432000	0.885526000
C	5.602129000	0.280134000	-0.288015000
C	3.519749000	0.978210000	1.884590000

C	5.268962000	-1.559927000	2.056412000
H	-0.914851000	2.632620000	3.114540000
H	-2.433075000	4.835865000	-1.518050000
H	-5.234665000	-0.613590000	2.912655000
H	-4.631661000	1.023507000	-2.180802000
C	0.021018000	-3.462552000	1.382884000
C	-0.696756000	-1.522833000	2.778792000
C	1.877839000	-3.464451000	-1.852639000
C	3.563469000	-4.382005000	0.568465000
C	0.743172000	1.109634000	-3.123445000
C	2.537010000	2.030698000	-1.646748000
H	-2.793983000	4.546923000	1.254522000
H	-5.143631000	3.720281000	1.538469000
H	-5.684929000	1.336150000	0.942508000
H	2.125957000	4.175037000	2.176660000
C	2.830946000	5.934135000	0.184063000
H	0.785286000	5.714695000	-1.641156000
H	0.096613000	1.381187000	2.353535000
H	-1.856197000	3.293457000	-2.172145000
H	-5.015458000	-3.496058000	1.622568000
C	-5.488257000	-4.605403000	-0.851586000
H	-5.036489000	-2.533068000	-2.596014000
H	-3.488870000	-0.325569000	2.757885000
H	-3.114375000	0.208236000	-2.633851000
H	1.799899000	2.672388000	-1.128672000
H	2.957434000	2.601578000	-2.501841000
H	3.362843000	1.821183000	-0.942239000
H	0.241589000	0.206151000	-3.529417000
H	1.151531000	1.688995000	-3.976951000
H	-0.016588000	1.743572000	-2.619611000
H	-0.433302000	-0.539577000	3.217257000
H	-0.938792000	-2.222469000	3.605521000
H	-1.611634000	-1.391015000	2.166758000
H	-0.916677000	-3.375850000	0.795417000
H	-0.165727000	-4.163387000	2.224278000
H	0.788063000	-3.914869000	0.727503000
H	4.374937000	-4.153230000	1.288050000
H	3.873647000	-5.285981000	-0.000561000
H	2.660695000	-4.651905000	1.149515000
H	4.954596000	-2.198270000	-2.480184000
H	5.028410000	-3.961018000	-2.171866000
H	5.816603000	-2.833893000	-1.035949000
H	0.885151000	-3.124003000	-1.494066000
H	1.842992000	-4.571118000	-1.940191000
H	2.024209000	-3.053276000	-2.871429000
H	4.641994000	-2.049099000	2.827885000
H	6.019801000	-0.930689000	2.581921000
H	5.832761000	-2.347621000	1.514910000
H	6.102990000	-0.510495000	-0.881876000
H	6.383137000	0.768985000	0.334759000
H	5.217330000	1.041360000	-0.994069000
H	2.840696000	1.611012000	1.278802000

H	4.335509000	1.626510000	2.269721000
H	2.944324000	0.604032000	2.754367000
H	3.279407000	0.445988000	-3.780027000
H	3.759205000	-0.406025000	-2.280222000
H	2.430540000	-1.055913000	-3.297815000
H	1.455336000	-2.872781000	3.676844000
H	2.561901000	-2.636251000	2.287716000
H	1.991249000	-1.223200000	3.231229000
H	-6.258315000	-4.615104000	-1.650976000
H	-4.638217000	-5.223231000	-1.216864000
H	-5.909443000	-5.117471000	0.037058000
H	2.638626000	6.852950000	-0.406842000
H	3.175818000	6.240267000	1.193074000
H	3.680539000	5.405738000	-0.303100000

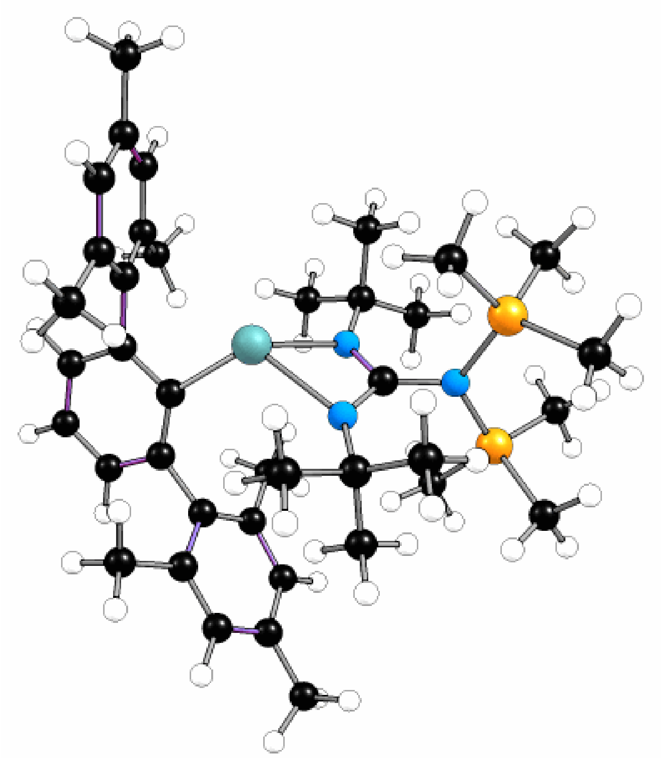

2a_TMS
SCF = -2982.12639638
H(0 K) = -2981.269360
H(298 K) = -2981.204877
G(298 K) = -2981.368424
SCF+D3BJ = -2982.42316979
PCM SCF (Benzene) = -2982.12909153
BS2 (def2-tzvp) = -2984.28841437
Low Freq. = 13.3956 cm^{-1}, 18.3434 cm^{-1}

106
2a_TMS

C	-0.643057000	4.201152000	-0.463093000
C	-0.676354000	3.328014000	0.660900000
C	0.387148000	3.360857000	1.602038000
C	1.457407000	4.257832000	1.406126000

C	1.507094000	5.134263000	0.304893000
C	0.447382000	5.081267000	-0.621734000
C	-1.942081000	2.554676000	0.935953000
C	-2.247133000	1.256597000	0.432004000
C	-3.584029000	0.770998000	0.612420000
C	-4.533888000	1.516452000	1.349290000
C	-4.192080000	2.755058000	1.907428000
C	-2.910425000	3.274609000	1.681707000
C	-4.081658000	-0.505840000	-0.028179000
C	-4.353759000	-0.528751000	-1.430474000
C	-4.853391000	-1.706098000	-2.019238000
C	-5.109411000	-2.870233000	-1.268844000
C	-4.887836000	-2.812214000	0.119133000
C	-4.401146000	-1.651447000	0.756642000
C	-4.124857000	0.688933000	-2.298518000
H	-4.642864000	0.585229000	-3.272754000
C	-4.285992000	-1.642339000	2.266043000
H	-4.167996000	-2.669846000	2.665755000
Sn	-0.996353000	-0.348893000	-0.697682000
N	0.584900000	-1.149553000	0.805463000
Si	0.295655000	-2.082120000	2.286327000
C	1.827758000	-2.558447000	3.296080000
C	0.362283000	2.462438000	2.815841000
H	1.223465000	2.660458000	3.484479000
C	-1.790180000	4.244302000	-1.450639000
H	-1.538311000	4.865069000	-2.333569000
N	1.267727000	0.116980000	-0.948560000
Si	1.988940000	0.908629000	-2.365590000
C	3.155903000	-0.153577000	-3.421527000
C	1.596534000	-0.795767000	-0.014684000
N	2.883202000	-1.436010000	0.033443000
Si	3.006218000	-3.078796000	-0.718890000
C	4.625702000	-3.233042000	-1.698547000
Si	4.289976000	-0.600347000	0.791891000
C	5.567562000	0.057941000	-0.451901000
C	3.629376000	0.836399000	1.824520000
C	5.247279000	-1.808938000	1.900657000
H	-0.571771000	2.591232000	3.401683000
H	-2.705514000	4.669407000	-0.984753000
H	-5.201203000	-1.214685000	2.731657000
H	-4.474306000	1.619196000	-1.806788000
C	-0.708925000	-3.624236000	1.822013000
C	-0.749452000	-1.006102000	3.448509000
C	1.529456000	-3.334579000	-1.871488000
C	3.031284000	-4.524164000	0.514798000
C	0.522944000	1.396613000	-3.466404000
C	2.880133000	2.497869000	-1.857410000
H	-2.646323000	4.272382000	2.070984000
H	-4.931290000	3.329068000	2.489388000
H	-5.556354000	1.120046000	1.465525000
H	2.270393000	4.282501000	2.151898000
C	2.673637000	6.075990000	0.104282000

H	0.457462000	5.760511000	-1.491618000
H	0.396164000	1.396343000	2.512426000
H	-2.073955000	3.231519000	-1.802627000
H	-5.113421000	-3.698651000	0.736782000
C	-5.598116000	-4.136649000	-1.935912000
H	-5.055796000	-1.709558000	-3.104086000
H	-3.438807000	-1.027441000	2.622150000
H	-3.042393000	0.835965000	-2.519237000
H	2.179618000	3.172250000	-1.324427000
H	3.253498000	3.029006000	-2.759540000
H	3.746118000	2.315685000	-1.190119000
H	-0.027494000	0.510803000	-3.847691000
H	0.882740000	1.971856000	-4.346433000
H	-0.194196000	2.045993000	-2.920739000
H	-0.108120000	-0.305900000	4.022545000
H	-1.289438000	-1.641601000	4.182531000
H	-1.496597000	-0.397472000	2.899938000
H	-1.640545000	-3.340750000	1.287331000
H	-1.000392000	-4.193214000	2.730857000
H	-0.143664000	-4.310955000	1.158351000
H	3.854649000	-4.440821000	1.253166000
H	3.192913000	-5.463895000	-0.057337000
H	2.078753000	-4.634459000	1.069535000
H	4.712456000	-2.478702000	-2.505311000
H	4.650993000	-4.238293000	-2.172141000
H	5.525086000	-3.157507000	-1.052840000
H	0.567093000	-3.275272000	-1.322517000
H	1.595025000	-4.342958000	-2.332681000
H	1.496134000	-2.585582000	-2.686993000
H	4.622293000	-2.225992000	2.714746000
H	6.097686000	-1.266386000	2.367537000
H	5.680341000	-2.656280000	1.329200000
H	6.016665000	-0.755916000	-1.056453000
H	6.392926000	0.545931000	0.111273000
H	5.151090000	0.810104000	-1.149924000
H	3.033114000	1.545797000	1.214690000
H	4.477960000	1.397830000	2.269864000
H	2.983947000	0.480971000	2.653053000
H	3.487645000	0.446481000	-4.296594000
H	4.060836000	-0.467815000	-2.866955000
H	2.654918000	-1.063039000	-3.812024000
H	1.482363000	-3.071703000	4.220065000
H	2.525870000	-3.234807000	2.771015000
H	2.387379000	-1.654358000	3.612859000
H	-6.300671000	-3.916915000	-2.766267000
H	-4.751435000	-4.711146000	-2.372988000
H	-6.112496000	-4.806890000	-1.217776000
H	2.361492000	7.013678000	-0.399763000
H	3.152764000	6.345777000	1.067481000
H	3.458588000	5.610323000	-0.532575000

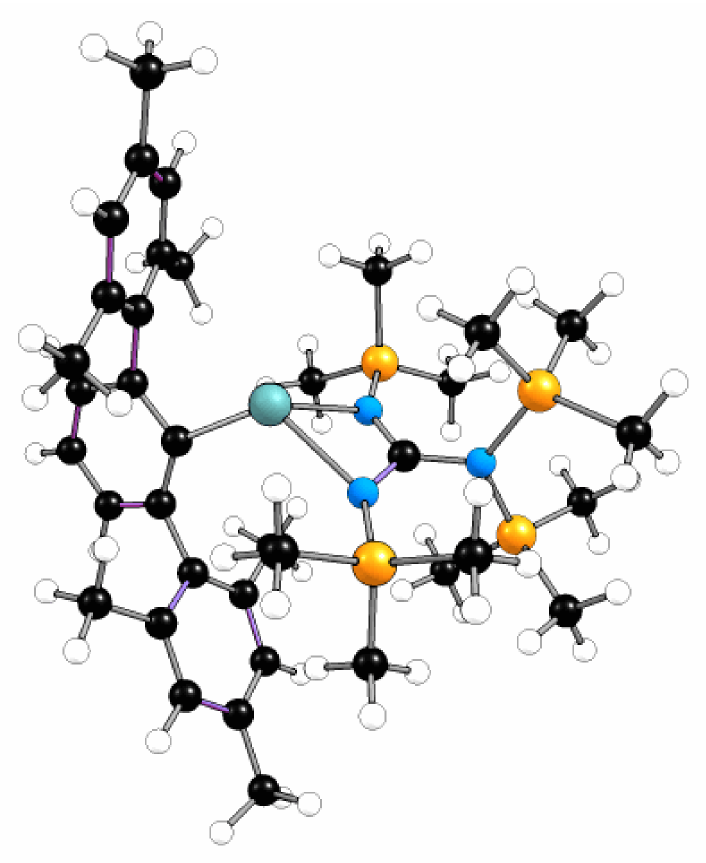

1b

SCF = -2252.02008847

H(0 K) = -2251.229347

H(298 K) = -2251.177013

G(298 K) = -2251.314643

SCF+D3BJ = -2252.27714034

PCM SCF (Benzene) = -2252.02216159

BS2 (def2-tzvp) = -2253.79958272

Low Freq. = 13.5333 cm^{-1}, 18.8090 cm^{-1}

95

1b

C	3.050504000	-0.767137000	-1.423005000
C	2.919689000	-0.528015000	-0.013320000
C	3.797838000	0.402509000	0.627584000
C	4.703581000	1.142854000	-0.163455000
C	4.770776000	0.973613000	-1.551328000
C	3.962097000	0.010965000	-2.168597000
C	1.878257000	-1.293384000	0.771273000
C	0.485057000	-1.053227000	0.578987000
C	-0.458144000	-1.896843000	1.234413000
C	-0.018759000	-2.889193000	2.140468000
C	1.352281000	-3.085350000	2.364883000
C	2.290604000	-2.309901000	1.665888000
Sn	-0.439864000	0.136131000	-1.151752000
N	-0.932462000	2.127229000	-0.519983000
C	-1.929621000	-1.813369000	0.880719000

C	-2.849772000	-1.075613000	1.688279000
C	-4.206359000	-1.022709000	1.304219000
C	-4.665343000	-1.683820000	0.157062000
C	-3.771217000	-2.434320000	-0.616268000
C	-2.405211000	-2.526461000	-0.270857000
C	-2.412696000	-0.404266000	2.991462000
C	-2.687852000	-1.327846000	4.200462000
C	-1.499684000	-3.476839000	-1.063488000
C	-1.633453000	-4.915820000	-0.513483000
C	3.850842000	0.579482000	2.150152000
C	5.022277000	-0.233857000	2.750477000
C	2.366173000	-1.961253000	-2.104412000
C	3.235259000	-3.227524000	-1.917305000
C	-3.062159000	0.975803000	3.211459000
C	-1.745657000	-3.450910000	-2.583997000
C	3.966456000	2.050668000	2.598019000
C	2.040360000	-1.746442000	-3.593486000
H	3.844749000	2.126900000	3.698867000
H	3.196964000	2.687973000	2.122102000
H	4.962121000	-1.310501000	2.494547000
H	5.034596000	-0.146741000	3.857858000
H	5.996360000	0.141744000	2.370219000
H	-0.757964000	-3.529756000	2.649398000
H	1.693020000	-3.867127000	3.062507000
H	3.365846000	-2.507245000	1.801748000
H	-4.921191000	-0.454581000	1.919394000
H	-5.728081000	-1.623796000	-0.128016000
H	-4.143496000	-2.973112000	-1.501168000
H	-1.313909000	-0.256623000	2.929035000
H	-2.923933000	1.635953000	2.331153000
H	-2.613755000	1.477325000	4.094589000
H	-4.152286000	0.896845000	3.409189000
H	-3.775036000	-1.533089000	4.303704000
H	-2.339639000	-0.854659000	5.143173000
H	-2.170259000	-2.302731000	4.099614000
H	-0.449513000	-3.159968000	-0.889076000
H	-0.985866000	-4.069915000	-3.105279000
H	-1.684243000	-2.420026000	-2.990532000
H	-2.738882000	-3.867378000	-2.855507000
H	-2.671333000	-5.292120000	-0.638614000
H	-1.384317000	-4.964075000	0.565629000
H	-0.951873000	-5.607740000	-1.052413000
H	5.371238000	1.871830000	0.321623000
H	5.472805000	1.575360000	-2.150601000
H	4.059254000	-0.157700000	-3.251712000
H	2.907104000	0.171858000	2.569490000
H	1.407522000	-2.152481000	-1.577233000
H	2.956331000	-1.691873000	-4.219285000
H	1.456392000	-0.817133000	-3.759575000
H	1.438632000	-2.595439000	-3.978767000
H	2.743470000	-4.114197000	-2.371264000
H	3.409337000	-3.445713000	-0.844294000

H	4.226153000	-3.099245000	-2.403100000
H	4.961006000	2.480886000	2.354018000
Si	-0.028350000	3.100944000	0.674555000
Si	-2.129077000	2.911232000	-1.596880000
C	0.366702000	2.109298000	2.236371000
C	1.583412000	3.717856000	-0.123253000
C	-0.991780000	4.629006000	1.285927000
C	-3.705574000	3.375162000	-0.640555000
C	-1.416922000	4.456872000	-2.451620000
C	-2.705418000	1.762050000	-3.002534000
H	0.994995000	2.718941000	2.919129000
H	-0.566827000	1.858879000	2.776847000
H	0.899686000	1.162627000	2.020534000
H	-0.338410000	5.188599000	1.990480000
H	-1.284809000	5.333507000	0.481368000
H	-1.908004000	4.342855000	1.843252000
H	2.156073000	4.371394000	0.569907000
H	2.240495000	2.869506000	-0.410216000
H	1.372256000	4.305127000	-1.041259000
H	-4.461560000	3.801914000	-1.334918000
H	-4.148224000	2.470916000	-0.171628000
H	-3.524381000	4.119862000	0.159655000
H	-2.183086000	4.912372000	-3.115609000
H	-1.088125000	5.240108000	-1.738531000
H	-0.543030000	4.186671000	-3.081761000
H	-3.468592000	2.319330000	-3.589994000
H	-1.892436000	1.475846000	-3.701586000
H	-3.188179000	0.829954000	-2.639918000

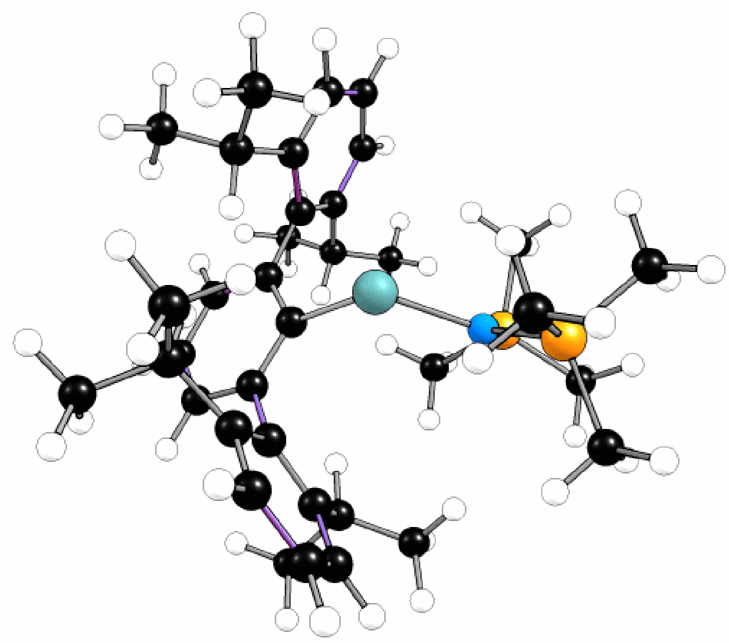

TS 1b–INTb

SCF = -2636.36445351

H(0 K) = -2635.376618

H(298 K) = -2635.312016

G(298 K) = -2635.475079
SCF+D3BJ = -2636.68477519
PCM SCF (Benzene) = -2636.36712397
BS2 (def2-tzvp) = -2638.55831111
Low Freq. = -173.9784 cm^{-1}, 15.7506 cm^{-1}

118
TS 1b–INTb

Sn	-0.107535000	-1.131573000	0.131072000
Si	2.098653000	0.118439000	-2.381510000
Si	2.199183000	-2.851636000	-1.491253000
N	1.650371000	-0.724067000	1.602457000
N	1.910734000	-1.122816000	-1.110680000
N	3.905903000	-0.130782000	0.909626000
C	-1.535549000	0.770067000	0.428974000
C	-2.305161000	-3.435815000	2.955076000
H	-2.025916000	-3.299900000	4.021156000
H	-1.372957000	-3.533997000	2.360508000
C	-1.396436000	2.146254000	0.817484000
C	-4.479545000	-2.179121000	3.276425000
H	-5.091055000	-1.299444000	2.992528000
H	-4.266345000	-2.107585000	4.364396000
H	-5.101151000	-3.085420000	3.112871000
C	-2.440624000	2.787841000	1.533529000
H	-2.305160000	3.839740000	1.833809000
C	-3.649524000	2.141693000	1.820013000
H	-4.445006000	2.664032000	2.375383000
C	-3.851637000	0.846629000	1.332371000
H	-4.828197000	0.352734000	1.464712000
C	-2.820932000	0.157713000	0.647054000
C	-0.297973000	3.107955000	0.395587000
C	0.763410000	3.483842000	1.274650000
C	1.658629000	4.501642000	0.879301000
H	2.481851000	4.788822000	1.551081000
C	1.517519000	5.161353000	-0.346476000
H	2.228125000	5.951887000	-0.637170000
C	0.458649000	4.816142000	-1.194284000
H	0.336394000	5.352072000	-2.148302000
C	-0.464115000	3.808230000	-0.844040000
C	0.921451000	2.869416000	2.668247000
H	0.425561000	1.876386000	2.638462000
C	2.392377000	2.655861000	3.077019000
H	2.961042000	2.079218000	2.319615000
H	2.446982000	2.111848000	4.043789000
H	2.919363000	3.620819000	3.235091000
C	0.204478000	3.722013000	3.740737000
H	0.638990000	4.743826000	3.783721000
H	0.315953000	3.263829000	4.746966000
H	-0.878729000	3.823771000	3.534869000
C	-1.671255000	3.575603000	-1.761287000
H	-2.065919000	2.562161000	-1.539710000
C	-1.328791000	3.625769000	-3.263179000

H	-2.204877000	3.307233000	-3.866365000
H	-0.480288000	2.961089000	-3.517049000
H	-1.068921000	4.652891000	-3.596977000
C	-2.795522000	4.590518000	-1.448484000
H	-2.447568000	5.629756000	-1.632779000
H	-3.127073000	4.525905000	-0.393631000
H	-3.680702000	4.408313000	-2.094640000
C	-3.230173000	-1.197687000	0.091295000
C	-3.440688000	-2.316720000	0.957574000
C	-3.919079000	-3.528022000	0.416722000
H	-4.072912000	-4.393747000	1.080373000
C	-4.195244000	-3.656138000	-0.949480000
H	-4.559741000	-4.613356000	-1.355883000
C	-4.017634000	-2.553053000	-1.793977000
H	-4.261527000	-2.651166000	-2.862551000
C	-3.555599000	-1.314438000	-1.300028000
C	-3.167360000	-2.253693000	2.464109000
H	-2.601864000	-1.317501000	2.659312000
C	-3.519882000	-0.107306000	-2.244300000
H	-2.723975000	0.574539000	-1.877406000
C	-3.191465000	-0.462489000	-3.706112000
H	-4.023359000	-1.009019000	-4.199647000
H	-2.278731000	-1.086231000	-3.788237000
H	-3.021912000	0.462791000	-4.294738000
C	-4.856769000	0.666907000	-2.171721000
H	-4.831284000	1.559367000	-2.832855000
H	-5.073768000	1.012851000	-1.141451000
H	-5.700754000	0.023060000	-2.500479000
C	5.140905000	-0.471049000	0.230089000
H	4.868058000	-0.745298000	-0.816606000
C	5.816432000	-1.684800000	0.894140000
H	6.083794000	-1.455615000	1.946956000
H	6.747607000	-1.952349000	0.353326000
H	5.149562000	-2.570388000	0.888886000
C	6.074543000	0.747985000	0.200132000
H	5.586588000	1.614072000	-0.289424000
H	7.002574000	0.510109000	-0.358540000
H	6.355497000	1.050623000	1.230419000
C	2.748607000	-0.543030000	0.951406000
C	1.738238000	-0.890436000	3.086715000
H	2.333520000	-0.033107000	3.472197000
C	0.336765000	-0.827218000	3.696328000
H	-0.194959000	0.101684000	3.408828000
H	0.400598000	-0.858125000	4.803167000
H	-0.274464000	-1.692878000	3.370703000
C	2.462291000	-2.188215000	3.476252000
H	1.899880000	-3.078677000	3.127420000
H	2.562965000	-2.253529000	4.579820000
H	3.482631000	-2.226493000	3.043933000
C	0.476885000	0.273531000	-3.361331000
H	0.570931000	1.025750000	-4.173524000
H	-0.364556000	0.587417000	-2.708095000

H	0.201045000	-0.692784000	-3.832127000
C	3.446883000	-0.257360000	-3.679656000
H	4.451056000	-0.421282000	-3.236536000
H	3.523061000	0.629370000	-4.346502000
H	3.206431000	-1.129827000	-4.321634000
C	2.547130000	1.789971000	-1.628539000
H	1.845465000	2.096666000	-0.827993000
H	2.521267000	2.576706000	-2.411645000
H	3.567401000	1.776309000	-1.194971000
C	0.945554000	-3.504369000	-2.770743000
H	1.096472000	-4.592941000	-2.937629000
H	1.059672000	-2.998713000	-3.752806000
H	-0.102961000	-3.359988000	-2.434225000
C	3.932070000	-3.248656000	-2.180811000
H	4.106033000	-2.849343000	-3.198299000
H	4.024743000	-4.355423000	-2.237006000
H	4.750898000	-2.886192000	-1.526202000
C	2.051469000	-3.944188000	0.062358000
H	2.253992000	-4.997249000	-0.230089000
H	1.044232000	-3.915436000	0.526450000
H	2.792749000	-3.664537000	0.838884000
H	-2.848303000	-4.401806000	2.884507000

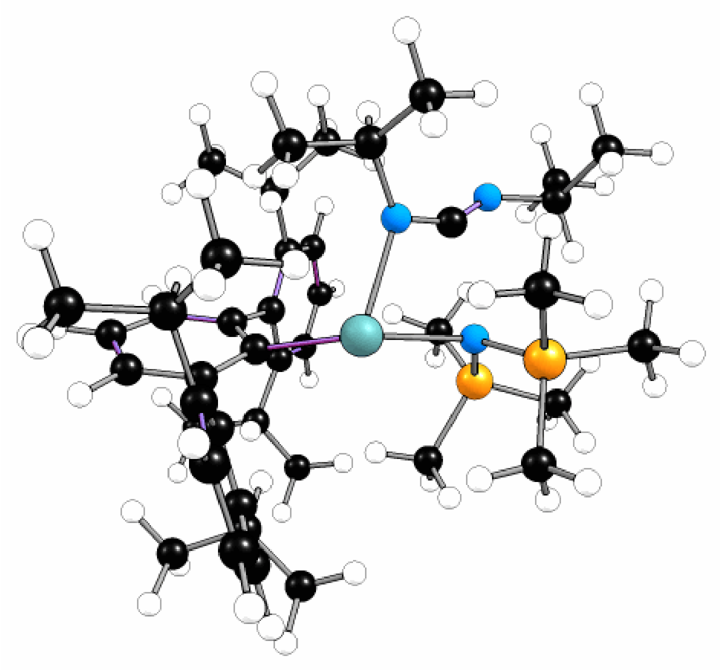

INTb
SCF = -2636.40862002
H(0 K) = -2635.418211
H(298 K) = -2635.353774
G(298 K) = -2635.516357
SCF+D3BJ = -2636.73405495
PCM SCF (Benzene) = -2636.41117141
BS2 (def2-tzvp) = -2638.59918804
Low Freq. = 14.5873 cm^{-1}, 23.0209 cm^{-1}

118
INTb

Sn	-0.055275000	0.069230000	0.527941000
Si	-3.713542000	1.465364000	0.135620000
Si	-3.428008000	-1.260790000	1.780125000
N	-1.226702000	-0.782275000	-1.086747000
N	-3.100522000	-0.224859000	0.336044000
N	-3.340920000	-1.532761000	-1.719332000
C	2.147817000	0.378850000	-0.129671000
C	3.103205000	-3.496307000	-3.178106000
H	3.602654000	-3.172691000	-4.115380000
H	2.028019000	-3.248814000	-3.259575000
C	2.543982000	1.737625000	-0.304866000
C	5.243613000	-3.242060000	-1.869168000
H	5.775316000	-2.740158000	-1.036260000
H	5.782951000	-3.004115000	-2.810699000
H	5.324149000	-4.337311000	-1.700432000
C	3.914561000	2.079021000	-0.392972000
H	4.201511000	3.134200000	-0.534224000
C	4.905899000	1.095765000	-0.266198000
H	5.971523000	1.369988000	-0.322176000
C	4.530367000	-0.235183000	-0.032875000
H	5.307408000	-1.002364000	0.113904000
C	3.166888000	-0.608007000	0.035788000
C	1.538483000	2.869487000	-0.302433000
C	1.045728000	3.423911000	-1.524987000
C	0.147182000	4.510748000	-1.467715000
H	-0.239309000	4.943860000	-2.403252000
C	-0.254095000	5.059539000	-0.242413000
H	-0.955988000	5.908714000	-0.220443000
C	0.251769000	4.534460000	0.953635000
H	-0.050264000	4.986042000	1.911431000
C	1.153755000	3.448905000	0.950987000
C	1.536016000	2.924828000	-2.887302000
H	1.912507000	1.890596000	-2.738368000
C	0.430329000	2.877997000	-3.960442000
H	-0.471414000	2.337184000	-3.607438000
H	0.801009000	2.365121000	-4.872251000
H	0.110286000	3.895164000	-4.271364000
C	2.722734000	3.780220000	-3.388703000
H	2.415356000	4.838764000	-3.528977000
H	3.094141000	3.401306000	-4.364614000
H	3.570681000	3.766785000	-2.675804000
C	1.778833000	2.995071000	2.276089000
H	2.200590000	1.980351000	2.114180000
C	0.773007000	2.897967000	3.439132000
H	1.266268000	2.472980000	4.338275000
H	-0.087780000	2.245948000	3.183603000
H	0.371787000	3.891438000	3.731572000
C	2.958369000	3.920414000	2.654186000
H	2.608341000	4.962763000	2.813908000
H	3.730546000	3.941661000	1.859152000

H	3.443883000	3.576233000	3.592095000
C	2.823877000	-2.047379000	0.337967000
C	3.042376000	-3.074410000	-0.633582000
C	2.648999000	-4.395481000	-0.327588000
H	2.799462000	-5.190113000	-1.075121000
C	2.066941000	-4.717655000	0.904478000
H	1.749864000	-5.751806000	1.114212000
C	1.919960000	-3.724219000	1.881742000
H	1.516318000	-3.996968000	2.868631000
C	2.313174000	-2.391125000	1.633906000
C	3.760591000	-2.810653000	-1.963687000
H	3.739244000	-1.715281000	-2.144075000
C	2.362683000	-1.395998000	2.803324000
H	2.234972000	-0.373231000	2.387848000
C	1.270871000	-1.604185000	3.867676000
H	1.411720000	-2.549982000	4.432677000
H	0.254777000	-1.622142000	3.421867000
H	1.298347000	-0.779676000	4.609872000
C	3.765071000	-1.437858000	3.455543000
H	3.837009000	-0.700803000	4.283579000
H	4.561832000	-1.204693000	2.720973000
H	3.972688000	-2.445623000	3.874575000
C	-4.782407000	-1.667379000	-1.647378000
H	-5.203852000	-1.174046000	-0.737845000
C	-5.151047000	-3.163376000	-1.596309000
H	-4.762341000	-3.685929000	-2.495482000
H	-6.252333000	-3.301993000	-1.557519000
H	-4.710290000	-3.657407000	-0.706085000
C	-5.419601000	-1.005186000	-2.885769000
H	-5.201997000	0.082028000	-2.919503000
H	-6.522792000	-1.134016000	-2.881846000
H	-5.015708000	-1.458983000	-3.815085000
C	-2.612666000	-0.886222000	-0.863562000
C	-0.754220000	-1.437971000	-2.332278000
H	-1.584117000	-1.316377000	-3.061411000
C	0.493795000	-0.771050000	-2.908892000
H	0.322312000	0.310538000	-3.070308000
H	0.739278000	-1.222896000	-3.892322000
H	1.378976000	-0.881111000	-2.250789000
C	-0.574075000	-2.952319000	-2.125125000
H	0.226818000	-3.169020000	-1.387711000
H	-0.306168000	-3.447884000	-3.082717000
H	-1.523651000	-3.400318000	-1.773111000
C	-3.064862000	2.612725000	1.497170000
H	-3.392551000	3.650006000	1.269005000
H	-1.956111000	2.616811000	1.537940000
H	-3.448703000	2.355721000	2.504459000
C	-5.610946000	1.560566000	0.200590000
H	-6.096653000	0.955468000	-0.591672000
H	-5.927932000	2.617147000	0.062768000
H	-6.010510000	1.223896000	1.179874000
C	-3.114690000	2.124019000	-1.533649000

H	-2.018509000	2.292241000	-1.535107000
H	-3.604399000	3.100571000	-1.734259000
H	-3.358518000	1.438670000	-2.370353000
C	-2.956228000	-0.343700000	3.373517000
H	-1.947395000	0.112472000	3.312138000
H	-2.954676000	-1.070200000	4.214667000
H	-3.683083000	0.453417000	3.632898000
C	-5.251315000	-1.761596000	1.984136000
H	-5.928481000	-0.883462000	2.014841000
H	-5.366073000	-2.302403000	2.949110000
H	-5.604994000	-2.435508000	1.178363000
C	-2.406173000	-2.847655000	1.630894000
H	-2.622260000	-3.386619000	0.686028000
H	-2.664164000	-3.523615000	2.473935000
H	-1.312463000	-2.665319000	1.667982000
H	3.194101000	-4.602352000	-3.130580000

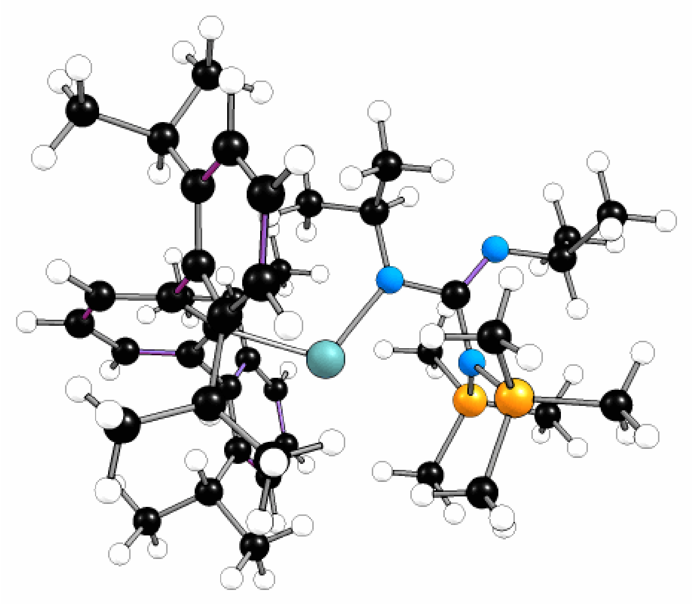

TS INTb–2b
SCF = -2636.38466824
H(0 K) = -2635.394809
H(298 K) = -2635.331208
G(298 K) = -2635.491685
SCF+D3BJ = -2636.70156670
PCM SCF (Benzene) = -2636.38752797
BS2 (def2-tzvp) = -2638.57722807
Low Freq. = -31.5339 cm^{-1}, 13.2521 cm^{-1}

118
TS INTb–2b

Sn	-0.119206000	0.149269000	-0.752102000
Si	4.767644000	-0.413552000	1.493283000
Si	3.803066000	1.060257000	-1.069326000
N	1.234889000	-0.859276000	0.553760000
N	3.604507000	-0.284792000	0.121312000

N	2.566417000	-2.323697000	-0.699946000
C	-2.220006000	0.465486000	0.147978000
C	-3.072236000	-3.092106000	3.462830000
H	-3.376969000	-2.658560000	4.438743000
H	-1.982353000	-2.938786000	3.344756000
C	-2.526684000	1.848076000	0.332816000
C	-5.377423000	-2.710899000	2.501703000
H	-5.992240000	-2.209232000	1.727760000
H	-5.723044000	-2.353268000	3.495057000
H	-5.592140000	-3.799452000	2.441850000
C	-3.863448000	2.270050000	0.528114000
H	-4.072905000	3.341554000	0.681709000
C	-4.917200000	1.347339000	0.489141000
H	-5.958461000	1.683997000	0.617124000
C	-4.634363000	-0.005793000	0.260518000
H	-5.460387000	-0.731858000	0.193743000
C	-3.303747000	-0.466063000	0.103221000
C	-1.482829000	2.942885000	0.254482000
C	-0.838619000	3.425537000	1.436482000
C	0.031419000	4.531633000	1.335394000
H	0.528411000	4.914733000	2.239956000
C	0.268154000	5.161165000	0.106274000
H	0.949222000	6.025547000	0.050314000
C	-0.363401000	4.687349000	-1.050563000
H	-0.175348000	5.189381000	-2.012690000
C	-1.245532000	3.587557000	-1.002873000
C	-1.130804000	2.820362000	2.812758000
H	-1.485703000	1.781775000	2.643612000
C	0.111326000	2.750340000	3.722648000
H	0.972081000	2.274876000	3.209096000
H	-0.112816000	2.160718000	4.635846000
H	0.435789000	3.757146000	4.061928000
C	-2.269221000	3.587743000	3.523717000
H	-1.985323000	4.648696000	3.692651000
H	-2.491377000	3.132879000	4.512567000
H	-3.204144000	3.577536000	2.929176000
C	-1.976683000	3.165004000	-2.282709000
H	-2.402553000	2.154720000	-2.103622000
C	-1.047705000	3.063962000	-3.509073000
H	-1.602336000	2.653996000	-4.379164000
H	-0.182762000	2.397725000	-3.311009000
H	-0.652023000	4.055381000	-3.816235000
C	-3.162007000	4.113251000	-2.572995000
H	-2.806771000	5.151683000	-2.746365000
H	-3.879911000	4.138898000	-1.728717000
H	-3.714126000	3.787031000	-3.479986000
C	-3.124790000	-1.946756000	-0.132504000
C	-3.394665000	-2.875128000	0.923124000
C	-3.252944000	-4.256165000	0.670474000
H	-3.451280000	-4.976326000	1.480157000
C	-2.854975000	-4.731253000	-0.584935000
H	-2.735902000	-5.813065000	-0.756889000

C	-2.624735000	-3.823189000	-1.625980000
H	-2.344178000	-4.204735000	-2.619676000
C	-2.771385000	-2.433186000	-1.431554000
C	-3.867522000	-2.435359000	2.315479000
H	-3.713933000	-1.338709000	2.390544000
C	-2.691867000	-1.505316000	-2.651256000
H	-2.459717000	-0.481320000	-2.285881000
C	-1.599220000	-1.893937000	-3.665306000
H	-1.834053000	-2.845705000	-4.187284000
H	-0.607167000	-2.012777000	-3.180437000
H	-1.504268000	-1.112140000	-4.447343000
C	-4.074681000	-1.420843000	-3.338076000
H	-4.046039000	-0.723347000	-4.202298000
H	-4.854179000	-1.063202000	-2.634982000
H	-4.388724000	-2.418368000	-3.713299000
C	3.790140000	-2.826364000	-1.311334000
H	4.587565000	-2.043038000	-1.328421000
C	3.477959000	-3.258554000	-2.755623000
H	2.703143000	-4.053476000	-2.760571000
H	4.389187000	-3.650913000	-3.254254000
H	3.093228000	-2.409987000	-3.355708000
C	4.308228000	-4.026952000	-0.495618000
H	4.563548000	-3.734024000	0.542897000
H	5.219154000	-4.454056000	-0.965047000
H	3.536440000	-4.823308000	-0.443511000
C	2.500907000	-1.211785000	-0.050701000
C	0.895162000	-1.678342000	1.749617000
H	1.851717000	-1.880511000	2.273874000
C	0.013467000	-0.889142000	2.720725000
H	0.505810000	0.056816000	3.019992000
H	-0.184255000	-1.482246000	3.637568000
H	-0.964730000	-0.638021000	2.258267000
C	0.290635000	-3.043161000	1.377479000
H	-0.708302000	-2.915125000	0.912510000
H	0.175068000	-3.684311000	2.278162000
H	0.944072000	-3.558346000	0.647716000
C	4.964087000	1.275394000	2.341708000
H	5.654274000	1.175990000	3.207141000
H	3.986192000	1.632815000	2.726971000
H	5.377020000	2.063583000	1.680356000
C	6.475675000	-1.029832000	0.927018000
H	6.412911000	-2.055417000	0.507345000
H	7.159684000	-1.068740000	1.802690000
H	6.946206000	-0.380452000	0.163859000
C	4.215958000	-1.623536000	2.846045000
H	3.381492000	-1.228338000	3.459512000
H	5.085339000	-1.768678000	3.523549000
H	3.927262000	-2.622977000	2.462184000
C	2.917861000	2.637448000	-0.505014000
H	1.819687000	2.501578000	-0.419168000
H	3.090773000	3.458020000	-1.234071000
H	3.286640000	2.978527000	0.483716000

C	5.635971000	1.499639000	-1.321377000
H	6.157464000	1.854042000	-0.409996000
H	5.673185000	2.328566000	-2.061500000
H	6.210550000	0.650479000	-1.744990000
C	3.170251000	0.538237000	-2.776009000
H	2.123160000	0.174066000	-2.760828000
H	3.809952000	-0.254736000	-3.214548000
H	3.208212000	1.419024000	-3.452761000
H	-3.255372000	-4.185989000	3.522719000

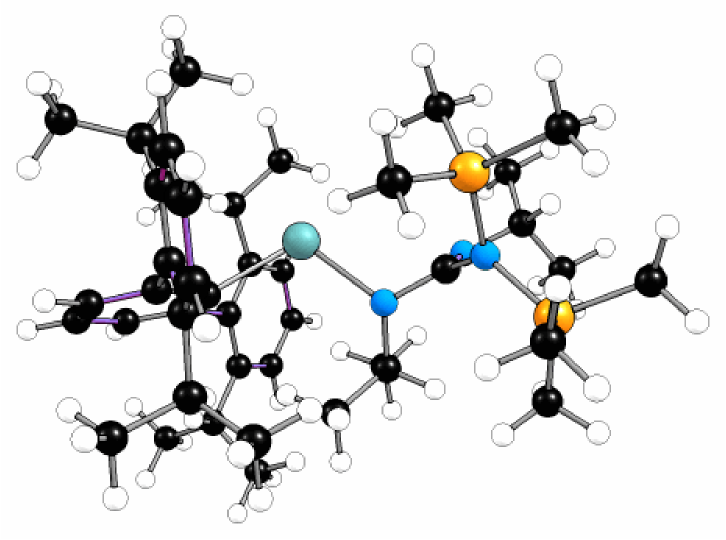

2b

SCF = -2636.43261760

H(0 K) = -2635.441749

H(298 K) = -2635.377177

G(298 K) = -2635.540733

SCF+D3BJ = -2636.74682763

PCM SCF (Benzene) = -2636.43501280

BS2 (def2-tzvp) = -2638.62230589

Low Freq. = 15.2912 cm^{-1}, 20.1724 cm^{-1}

118

2b

C	2.298853000	3.234795000	-1.022969000
C	2.364135000	2.573403000	0.241669000
C	2.048178000	3.282389000	1.440668000
C	1.646512000	4.632179000	1.348217000
C	1.553304000	5.278158000	0.107883000
C	1.877668000	4.581965000	-1.064940000
C	2.938004000	1.181230000	0.348664000
C	2.183173000	-0.022264000	0.184802000
C	2.889946000	-1.254770000	0.344277000
C	4.270977000	-1.268841000	0.662045000
C	4.991789000	-0.078863000	0.815796000
C	4.320177000	1.138981000	0.659260000
Sn	0.216882000	0.017779000	-1.089995000
N	-1.593458000	-1.122099000	-0.192424000

C	-2.317725000	0.009799000	-0.074484000
N	-3.742277000	0.019472000	0.049473000
Si	-4.443044000	-0.128927000	1.708805000
C	-3.043826000	0.019322000	2.970171000
C	2.267734000	-2.624091000	0.214525000
C	1.838914000	-3.305210000	1.393842000
C	1.409152000	-4.645152000	1.287868000
C	1.398602000	-5.308155000	0.052455000
C	1.827998000	-4.637291000	-1.100924000
C	2.275912000	-3.299255000	-1.043895000
C	1.913912000	-2.645118000	2.775757000
C	3.050173000	-3.264690000	3.619177000
C	2.857345000	-2.644910000	-2.303483000
C	4.294665000	-3.151592000	-2.560670000
C	2.196307000	2.638850000	2.825083000
C	3.331751000	3.308554000	3.629714000
C	2.793770000	2.558753000	-2.308029000
C	4.257247000	2.965082000	-2.596326000
N	-1.569159000	1.129492000	-0.125617000
C	-2.139168000	2.477148000	-0.160980000
C	-1.758283000	3.208042000	-1.460822000
C	-5.702995000	1.261019000	1.996546000
C	-5.336007000	-1.781685000	1.982264000
Si	-4.753806000	0.221041000	-1.434352000
C	-3.710709000	-0.201634000	-2.953964000
C	-5.439532000	1.980724000	-1.651583000
C	-6.245776000	-0.949007000	-1.360328000
C	-2.198813000	-2.441590000	-0.390877000
C	-1.632070000	-3.128689000	-1.644788000
C	0.576546000	-2.682864000	3.540339000
C	1.980898000	-2.831316000	-3.557206000
C	0.880790000	2.627943000	3.629024000
C	1.903336000	2.826012000	-3.536182000
C	-2.023299000	-3.346712000	0.840235000
C	-1.744358000	3.309009000	1.068547000
H	1.035712000	2.140879000	4.615189000
H	0.087945000	2.068827000	3.094074000
H	4.295582000	3.271661000	3.081697000
H	3.475297000	2.797505000	4.605524000
H	3.107641000	4.376254000	3.840860000
H	4.777969000	-2.239834000	0.788296000
H	6.066847000	-0.100476000	1.056095000
H	4.865861000	2.088859000	0.785175000
H	1.085577000	-5.184507000	2.193091000
H	1.062352000	-6.355749000	-0.010367000
H	1.833432000	-5.170854000	-2.064886000
H	2.166246000	-1.576926000	2.614468000
H	-0.228460000	-2.183198000	2.965826000
H	0.675015000	-2.160887000	4.515602000
H	0.245955000	-3.721603000	3.755855000
H	2.864376000	-4.341883000	3.819375000
H	3.137762000	-2.750085000	4.599815000

H	4.029211000	-3.183581000	3.104274000
H	2.927464000	-1.553617000	-2.106206000
H	2.415220000	-2.277492000	-4.415911000
H	0.951325000	-2.450345000	-3.396970000
H	1.907250000	-3.897148000	-3.861603000
H	4.303012000	-4.246382000	-2.750799000
H	4.955625000	-2.954954000	-1.692474000
H	4.737415000	-2.649095000	-3.447015000
H	1.405203000	5.190232000	2.267750000
H	1.236918000	6.332456000	0.055069000
H	1.825586000	5.104292000	-2.033367000
H	2.486001000	1.580322000	2.665526000
H	2.791061000	1.461939000	-2.127863000
H	1.915869000	3.895001000	-3.838063000
H	0.849017000	2.535526000	-3.348672000
H	2.264038000	2.239406000	-4.406902000
H	4.638959000	2.448139000	-3.502544000
H	4.924651000	2.705769000	-1.749902000
H	4.337872000	4.059864000	-2.768553000
H	-3.292418000	-2.288558000	-0.543223000
H	-0.951649000	-3.564778000	1.016605000
H	-2.549571000	-4.313968000	0.693438000
H	-2.431241000	-2.870663000	1.753885000
H	-1.762826000	-2.499315000	-2.548355000
H	-2.140441000	-4.099737000	-1.821991000
H	-0.549226000	-3.337910000	-1.521506000
H	-3.245333000	2.365384000	-0.141672000
H	-2.075215000	2.819797000	2.006445000
H	-2.214595000	4.314223000	1.025579000
H	-0.647161000	3.448011000	1.120066000
H	-0.661417000	3.371659000	-1.512992000
H	-2.245477000	4.204827000	-1.508935000
H	-2.065234000	2.630278000	-2.356936000
H	-5.960737000	2.347825000	-0.744343000
H	-6.177086000	1.978611000	-2.483670000
H	-4.648490000	2.713060000	-1.911468000
H	-5.941867000	-2.005907000	-1.215155000
H	-6.796081000	-0.883826000	-2.323780000
H	-6.963363000	-0.681337000	-0.557219000
H	-2.734494000	0.326461000	-2.958010000
H	-4.261095000	0.102816000	-3.869563000
H	-3.509236000	-1.289184000	-3.026551000
H	-5.244698000	2.263302000	1.866466000
H	-6.092295000	1.198130000	3.035582000
H	-6.577265000	1.191262000	1.316219000
H	-6.226326000	-1.896707000	1.332530000
H	-5.683874000	-1.835142000	3.036875000
H	-4.669678000	-2.649937000	1.804830000
H	-2.245637000	-0.729347000	2.791636000
H	-3.449115000	-0.147724000	3.990601000
H	-2.570724000	1.021214000	2.948860000
H	0.501876000	3.654349000	3.823361000

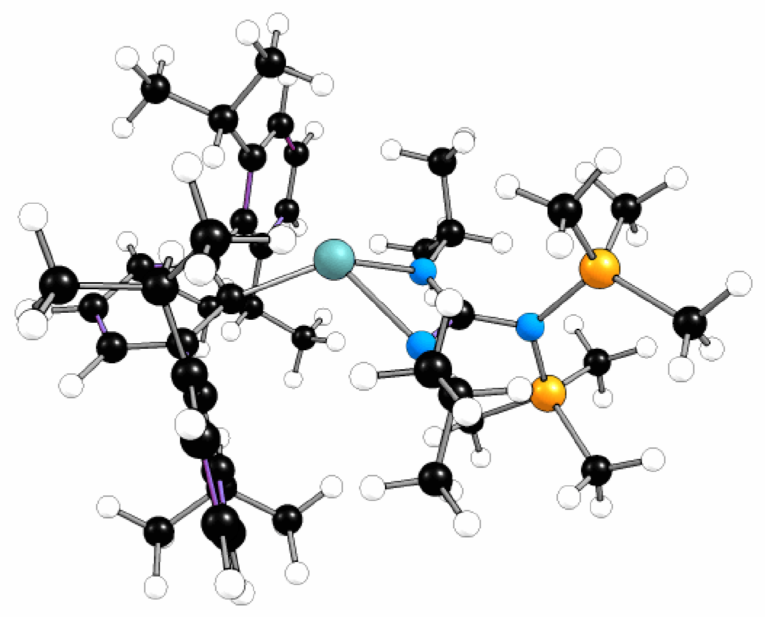

2b_tBu
SCF = -2714.96529786
H(0 K) = -2713.920510
H(298 K) = -2713.853892
G(298 K) = -2714.019073
SCF+D3BJ = -2715.30113122
PCM SCF (Benzene) = -2714.96799675
BS2 (def2-tzvp) = -2717.23805195
Low Freq. = 12.5389 cm^{-1}, 18.4439 cm^{-1}

124
2b_tBu

C	-3.688198000	-1.920402000	-1.558420000
C	-3.479287000	-1.702308000	-0.159736000
C	-3.511585000	-2.810355000	0.742741000
C	-3.698802000	-4.109841000	0.223436000
C	-3.871290000	-4.327870000	-1.148773000
C	-3.873632000	-3.236443000	-2.027515000
C	-3.368833000	-0.280095000	0.362058000
C	-2.167891000	0.509228000	0.323348000
C	-2.272184000	1.872245000	0.749504000
C	-3.500121000	2.371909000	1.255297000
C	-4.650841000	1.577762000	1.314027000
C	-4.584662000	0.262005000	0.844230000
Sn	-0.445438000	-0.862521000	-0.555748000
N	1.539098000	0.140039000	-1.143177000
C	2.240599000	-0.462722000	-0.157631000
N	3.667873000	-0.658851000	-0.166549000
Si	4.773312000	0.649839000	0.425497000
C	3.782893000	1.926203000	1.404127000
C	-1.184702000	2.921681000	0.658439000
C	-0.366371000	3.214957000	1.791077000
C	0.559491000	4.277734000	1.710797000

C	0.662133000	5.066050000	0.557586000
C	-0.177830000	4.808472000	-0.533651000
C	-1.111087000	3.749767000	-0.506417000
C	-0.547520000	2.478471000	3.123398000
C	-1.511775000	3.252995000	4.052114000
C	-2.082798000	3.582778000	-1.683665000
C	-3.333884000	4.473060000	-1.495048000
C	-3.396449000	-2.630356000	2.261321000
C	-4.790289000	-2.621487000	2.931011000
C	-3.776893000	-0.755460000	-2.550495000
C	-5.252333000	-0.368509000	-2.799166000
N	1.402034000	-0.916624000	0.807088000
C	1.572469000	-1.755692000	2.007776000
C	1.470065000	-3.260621000	1.651288000
C	6.127188000	-0.072425000	1.547501000
C	5.731718000	1.559009000	-0.946984000
Si	4.340577000	-2.219587000	-0.805974000
C	2.995459000	-3.156891000	-1.757558000
C	5.057573000	-3.372096000	0.532002000
C	5.817569000	-1.912099000	-1.963490000
C	1.907594000	0.834946000	-2.389816000
C	0.685805000	0.711315000	-3.330120000
C	0.774735000	2.206530000	3.863394000
C	-1.456293000	3.876723000	-3.061375000
C	-2.506597000	-3.694674000	2.935407000
C	-3.049370000	-1.026845000	-3.881637000
C	2.148920000	2.332052000	-2.082095000
C	0.386140000	-1.405411000	2.935106000
H	-2.330150000	-3.431758000	3.999455000
H	-1.520286000	-3.788917000	2.438999000
H	-5.434514000	-1.812124000	2.535630000
H	-4.697985000	-2.475905000	4.028552000
H	-5.315077000	-3.585713000	2.759082000
H	-3.547193000	3.421588000	1.587970000
H	-5.597008000	1.990283000	1.699659000
H	-5.490564000	-0.365855000	0.833573000
H	1.200506000	4.505681000	2.576581000
H	1.387678000	5.894497000	0.514917000
H	-0.111271000	5.449811000	-1.426074000
H	-1.017248000	1.498149000	2.894089000
H	1.503274000	1.684937000	3.212834000
H	0.593693000	1.576201000	4.759136000
H	1.251457000	3.143038000	4.223246000
H	-1.103397000	4.258118000	4.292605000
H	-1.657861000	2.707190000	5.008789000
H	-2.507777000	3.394792000	3.589445000
H	-2.423329000	2.524608000	-1.681614000
H	-2.159782000	3.588313000	-3.870339000
H	-0.509626000	3.324918000	-3.219462000
H	-1.242310000	4.958460000	-3.195621000
H	-3.046439000	5.545772000	-1.456771000
H	-3.876414000	4.232657000	-0.560680000

H	-4.040813000	4.340187000	-2.341765000
H	-3.715071000	-4.970299000	0.910591000
H	-4.013836000	-5.350435000	-1.534075000
H	-4.027206000	-3.411791000	-3.103816000
H	-2.936464000	-1.634572000	2.441438000
H	-3.286381000	0.120728000	-2.075919000
H	-3.555721000	-1.811578000	-4.483092000
H	-2.002317000	-1.354145000	-3.713740000
H	-3.027174000	-0.108053000	-4.504532000
H	-5.323689000	0.497882000	-3.491027000
H	-5.763454000	-0.093227000	-1.854452000
H	-5.812114000	-1.214416000	-3.253033000
C	3.120727000	0.256593000	-3.150941000
H	1.279405000	2.768355000	-1.553025000
H	2.321487000	2.908777000	-3.015735000
H	3.035012000	2.467920000	-1.432905000
H	0.509004000	-0.347930000	-3.610284000
H	0.844135000	1.291762000	-4.262313000
H	-0.231471000	1.100189000	-2.840180000
C	2.872998000	-1.484594000	2.793937000
H	0.442107000	-0.349255000	3.257179000
H	0.382452000	-2.051133000	3.837312000
H	-0.580862000	-1.548732000	2.409888000
H	0.514450000	-3.471398000	1.126991000
H	1.505718000	-3.887393000	2.567559000
H	2.292495000	-3.583474000	0.986541000
H	5.845962000	-2.876640000	1.133380000
H	5.533119000	-4.231735000	0.010566000
H	4.307072000	-3.786067000	1.232666000
H	5.575506000	-1.262995000	-2.827651000
H	6.142499000	-2.897245000	-2.363442000
H	6.690307000	-1.477023000	-1.433745000
H	2.002606000	-3.112710000	-1.265886000
H	3.286619000	-4.226195000	-1.832669000
H	2.874298000	-2.769873000	-2.788838000
H	5.720847000	-0.628473000	2.415732000
H	6.730428000	0.773692000	1.942478000
H	6.826929000	-0.739721000	1.002932000
H	6.317955000	0.871989000	-1.589414000
H	6.454421000	2.243267000	-0.450534000
H	5.091254000	2.178373000	-1.605114000
H	2.818540000	2.191952000	0.926265000
H	4.382475000	2.856678000	1.495887000
H	3.565139000	1.567660000	2.429642000
H	-2.984021000	-4.697457000	2.926602000
H	3.266456000	0.821753000	-4.095360000
H	4.051212000	0.327538000	-2.564885000
H	2.952414000	-0.804961000	-3.418036000
H	2.871341000	-2.081457000	3.729784000
H	3.773159000	-1.752554000	2.216395000
H	2.944434000	-0.415680000	3.075851000

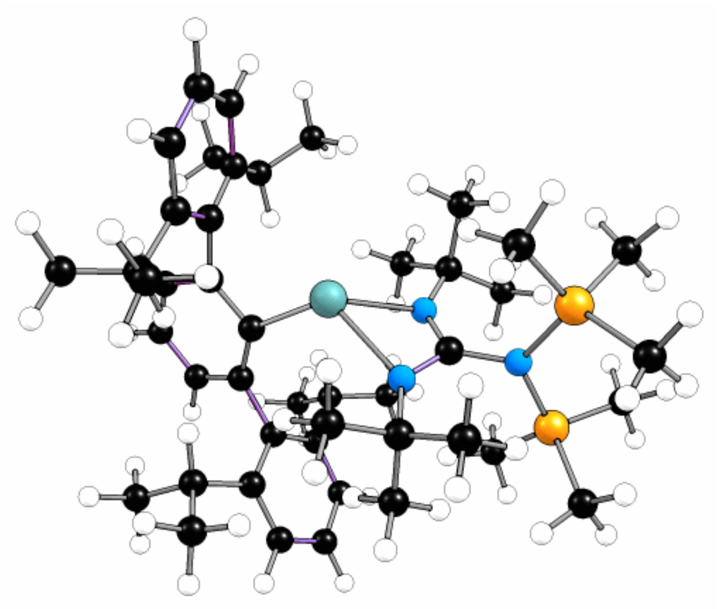

2b_TMS
SCF = -3217.77865979
H(0 K) = -3216.753995
H(298 K) = -3216.682697
G(298 K) = -3216.859676
SCF+D3BJ = -3218.12793897
PCM SCF (Benzene) = -3217.78088790
BS2 (def2-tzvp) = -3220.19089783
Low Freq. = 6.1506 cm^{-1}, 15.0052 cm^{-1}

124
SnSi4C43N3H73

C	-2.502353000	-2.901754000	-1.510771000
C	-2.885157000	-2.441541000	-0.212141000
C	-3.007095000	-3.375104000	0.873336000
C	-2.612134000	-4.712125000	0.655959000
C	-2.142814000	-5.143653000	-0.592567000
C	-2.112137000	-4.250311000	-1.670019000
C	-3.335213000	-1.014853000	0.001087000
C	-2.455771000	0.110371000	0.149687000
C	-3.076870000	1.370662000	0.426738000
C	-4.480207000	1.464594000	0.617344000
C	-5.320761000	0.364150000	0.428604000
C	-4.741816000	-0.864713000	0.097396000
Sn	-0.272592000	-0.161406000	-0.881266000
N	1.666770000	1.059612000	-0.284596000
C	2.322548000	-0.073294000	0.036109000
N	3.763495000	-0.164124000	0.032661000
Si	4.718873000	0.396455000	1.461715000
C	3.552408000	0.540310000	2.940795000
C	-2.379115000	2.703647000	0.543283000
C	-1.680706000	3.043532000	1.740204000
C	-1.199747000	4.360485000	1.901257000
C	-1.393093000	5.331271000	0.908402000

C	-2.087555000	4.996988000	-0.261505000
C	-2.602600000	3.698228000	-0.463405000
C	-1.544953000	2.051205000	2.898022000
C	-2.491449000	2.432558000	4.057285000
C	-3.445592000	3.430989000	-1.720873000
C	-4.782827000	4.204355000	-1.662828000
C	-3.641269000	-2.974189000	2.219860000
C	-4.263850000	-4.157734000	2.983781000
C	-2.675190000	-2.021241000	-2.756217000
C	-4.105151000	-2.190539000	-3.321855000
N	1.536594000	-1.141950000	0.270579000
Si	1.779248000	-2.823607000	0.809098000
C	1.575845000	-3.984492000	-0.676387000
C	6.126788000	-0.818540000	1.850065000
C	5.596714000	2.066367000	1.225734000
Si	4.558743000	-0.844045000	-1.448848000
C	3.312522000	-0.803475000	-2.871730000
C	5.189648000	-2.630923000	-1.286891000
C	6.113682000	0.150314000	-1.897855000
Si	2.130363000	2.698375000	-0.813725000
C	0.737401000	3.255510000	-1.956703000
C	-0.092145000	1.895347000	3.382473000
C	-2.711168000	3.750317000	-3.039679000
C	-2.710856000	-2.172892000	3.155188000
C	-1.630074000	-2.269766000	-3.858683000
C	2.209729000	3.854505000	0.683818000
C	0.414185000	-3.173781000	2.056016000
H	-3.289574000	-1.777549000	4.016647000
H	-2.246976000	-1.311951000	2.633696000
H	-4.955379000	-4.748867000	2.348185000
H	-4.840997000	-3.781147000	3.853961000
H	-3.494189000	-4.850832000	3.386187000
H	-4.910118000	2.440833000	0.893639000
H	-6.412565000	0.463098000	0.537877000
H	-5.381905000	-1.747995000	-0.066123000
H	-0.681540000	4.636880000	2.833928000
H	-1.011321000	6.354884000	1.052737000
H	-2.248315000	5.768451000	-1.032108000
H	-1.870498000	1.060952000	2.519767000
H	0.572050000	1.595434000	2.546736000
H	-0.025104000	1.116080000	4.170674000
H	0.308719000	2.836216000	3.817231000
H	-2.228711000	3.423646000	4.485989000
H	-2.432469000	1.684142000	4.876100000
H	-3.546293000	2.482890000	3.716956000
H	-3.688972000	2.348851000	-1.735375000
H	-3.368397000	3.525253000	-3.906230000
H	-1.784622000	3.154749000	-3.153116000
H	-2.430962000	4.823041000	-3.111252000
H	-4.612790000	5.302326000	-1.668450000
H	-5.361738000	3.961981000	-0.749065000
H	-5.416164000	3.958500000	-2.541673000

H	-2.683279000	-5.439045000	1.478408000
H	-1.832808000	-6.191597000	-0.733578000
H	-1.806608000	-4.613657000	-2.663213000
H	-4.476987000	-2.289933000	1.965476000
H	-2.579594000	-0.960919000	-2.435809000
H	-1.745278000	-3.271042000	-4.325488000
H	-0.594139000	-2.187629000	-3.469130000
H	-1.744878000	-1.521119000	-4.670035000
H	-4.257985000	-1.534103000	-4.204920000
H	-4.875151000	-1.931270000	-2.567949000
H	-4.280969000	-3.240012000	-3.641276000
C	3.727212000	2.894848000	-1.821406000
H	1.214919000	3.915816000	1.168304000
H	2.496234000	4.879352000	0.363820000
H	2.941266000	3.520695000	1.447074000
H	0.681321000	2.616534000	-2.863415000
H	0.900754000	4.301969000	-2.291005000
H	-0.241825000	3.226411000	-1.438744000
C	3.390600000	-3.206828000	1.731184000
H	0.496164000	-2.491251000	2.927134000
H	0.480814000	-4.218404000	2.427821000
H	-0.586807000	-3.041926000	1.605171000
H	0.562673000	-3.872685000	-1.115894000
H	1.683430000	-5.043578000	-0.357531000
H	2.317661000	-3.794003000	-1.478557000
H	5.938920000	-2.743499000	-0.476891000
H	5.695974000	-2.894895000	-2.241277000
H	4.387195000	-3.375198000	-1.121251000
H	5.909932000	1.217891000	-2.105691000
H	6.551544000	-0.296559000	-2.816648000
H	6.893080000	0.091748000	-1.110006000
H	2.409763000	-1.403832000	-2.635507000
H	3.779107000	-1.238073000	-3.781126000
H	2.977174000	0.223733000	-3.115737000
H	5.770813000	-1.833748000	2.110213000
H	6.690565000	-0.422356000	2.722271000
H	6.851438000	-0.908650000	1.014335000
H	6.328000000	2.039210000	0.392177000
H	6.165153000	2.291255000	2.154702000
H	4.904358000	2.911597000	1.047823000
H	2.779884000	1.317371000	2.772943000
H	4.131780000	0.829164000	3.843220000
H	3.027770000	-0.411249000	3.159163000
H	-1.897891000	-2.810306000	3.557351000
H	3.787969000	3.960625000	-2.133182000
H	4.644440000	2.659933000	-1.252182000
H	3.718529000	2.282254000	-2.745576000
H	3.342969000	-4.273702000	2.041253000
H	4.308275000	-3.067912000	1.134589000
H	3.475902000	-2.601093000	2.656222000

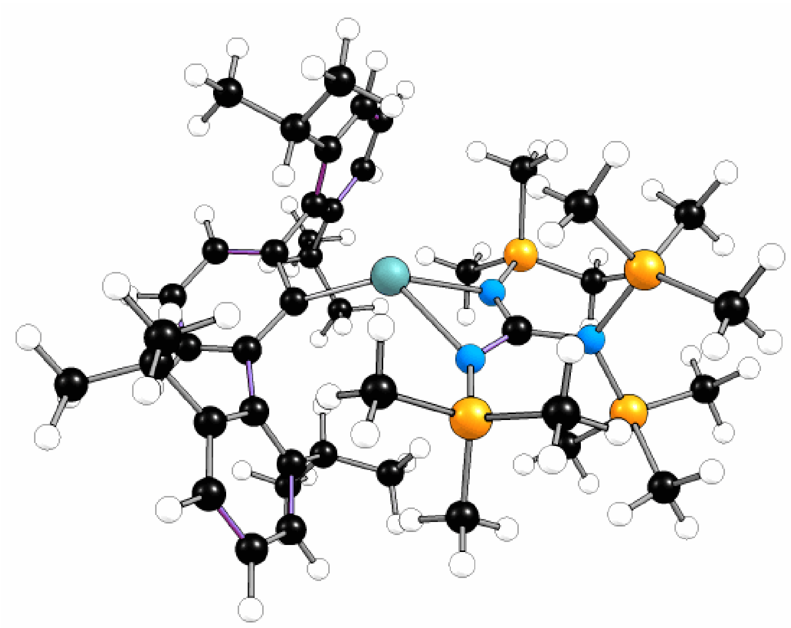

1c
SCF = -1668.27756964
H(0 K) = -1667.559759
H(298 K) = -1667.517348
G(298 K) = -1667.638803
SCF+D3BJ = -1668.48969791
PCM SCF (Benzene) = -1668.27965370
BS2 (def2-tzvp) = -1669.82167446
Low Freq. = 12.9316 cm^{-1}, 16.0387 cm^{-1}

85
1c

C	1.268767000	3.255999000	-1.249685000
C	-0.000107000	2.948523000	-0.416168000
C	-1.267995000	3.255675000	-1.251273000
C	-1.269086000	4.694604000	-1.797084000
C	0.001013000	4.993754000	-2.612212000
C	1.270212000	4.694892000	-1.795580000
N	-0.000263000	1.586259000	0.156328000
C	-0.000390000	1.505127000	1.625810000
C	-1.273944000	2.115883000	2.268073000
C	-1.265690000	2.016320000	3.804554000
C	-0.000953000	2.647260000	4.409095000
C	1.264101000	2.016601000	3.804926000
C	1.272823000	2.116218000	2.268447000
Sn	-0.000058000	-0.016523000	-1.207220000
C	0.000060000	-1.874999000	0.104059000
C	1.225213000	-2.545652000	0.376470000
C	1.214809000	-3.835123000	0.957021000
C	0.000018000	-4.474487000	1.252070000
C	-1.214753000	-3.835154000	0.956885000
C	-1.225113000	-2.545676000	0.376332000
C	2.541320000	-1.903301000	0.026131000
C	3.037322000	-1.969852000	-1.308160000

C	4.259785000	-1.337004000	-1.618128000
C	5.017578000	-0.655222000	-0.647599000
C	4.532214000	-0.643712000	0.675473000
C	3.317514000	-1.261632000	1.032274000
C	2.305360000	-2.751557000	-2.379716000
H	2.901411000	-2.802578000	-3.312378000
C	2.871568000	-1.274626000	2.478075000
H	1.804891000	-0.990850000	2.582618000
C	-2.541189000	-1.903330000	0.025873000
C	-3.317509000	-1.261709000	1.031927000
C	-4.532127000	-0.643675000	0.674956000
C	-5.017271000	-0.655054000	-0.648173000
C	-4.259384000	-1.336883000	-1.618629000
C	-3.037022000	-1.969807000	-1.308512000
C	-2.871888000	-1.274870000	2.477829000
H	-3.482083000	-0.585970000	3.095039000
C	-2.305010000	-2.751629000	-2.379950000
H	-2.901090000	-2.802868000	-3.312582000
H	-2.964449000	-2.291506000	2.917456000
H	-2.087171000	-3.787648000	-2.046629000
H	3.482246000	-0.586367000	3.095520000
H	2.087627000	-3.787663000	-2.046585000
H	-2.171050000	-4.344114000	1.164670000
H	0.000014000	-5.481695000	1.699413000
H	2.171095000	-4.344049000	1.164946000
H	-5.122860000	-0.142577000	1.461124000
C	-6.304035000	0.051835000	-1.012943000
H	-4.635942000	-1.392941000	-2.654362000
H	-1.804975000	-0.992079000	2.582590000
H	-1.323981000	-2.295843000	-2.637405000
H	5.122859000	-0.142620000	1.461713000
C	6.304564000	0.051447000	-1.012014000
H	4.636467000	-1.393082000	-2.653812000
H	2.963051000	-2.291408000	2.917578000
H	1.324281000	-2.295805000	-2.637026000
H	-0.000263000	0.418282000	1.873704000
H	-1.349961000	3.189408000	1.980795000
H	-2.167851000	1.612396000	1.842076000
H	-2.179597000	2.493651000	4.221020000
H	-1.313938000	0.944513000	4.105118000
H	-0.001046000	3.743241000	4.204476000
H	-0.001095000	2.536695000	5.515324000
H	2.177778000	2.494120000	4.221683000
H	1.312488000	0.944797000	4.105480000
H	1.348668000	3.189769000	1.981249000
H	2.167000000	1.612951000	1.842754000
H	-0.000806000	3.675852000	0.430101000
H	-1.312004000	2.533325000	-2.100032000
H	-2.169709000	3.062630000	-0.631897000
H	-2.178257000	4.870119000	-2.412472000
H	-1.332283000	5.409553000	-0.943481000
H	0.001621000	4.363956000	-3.531708000

H	0.001099000	6.050352000	-2.958131000
H	2.180070000	4.870546000	-2.409913000
H	1.332274000	5.409903000	-0.941946000
H	1.313964000	2.533553000	-2.098301000
H	2.169775000	3.063207000	-0.629214000
H	-6.771620000	-0.388578000	-1.916899000
H	-7.043512000	0.009351000	-0.186972000
H	-6.122399000	1.127917000	-1.230122000
H	6.769544000	-0.385570000	-1.918940000
H	6.124052000	1.128785000	-1.223817000
H	7.045785000	0.004152000	-0.187845000

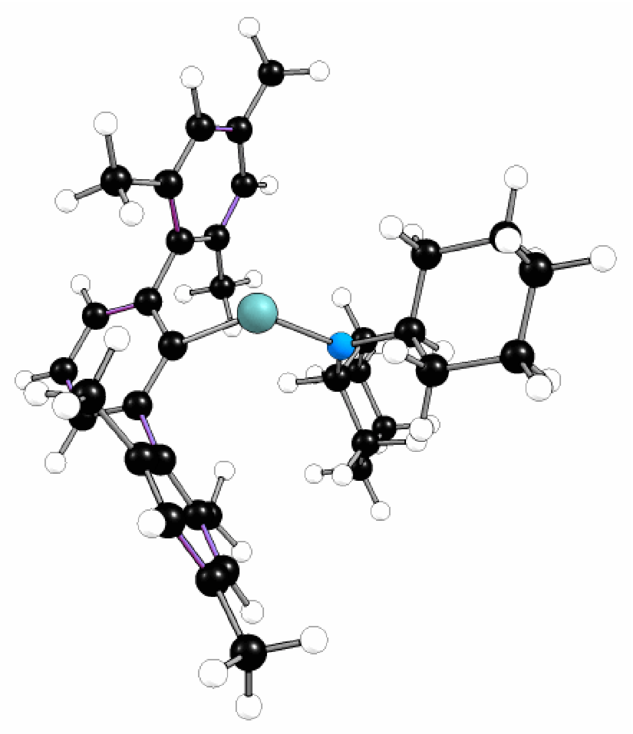

2c
SCF = -2052.68201112
H(0 K) = -2051.763178
H(298 K) = -2051.708964
G(298 K) = -2051.854353
SCF+D3BJ = -2052.94911382
PCM SCF (Benzene) = -2052.68490043
BS2 (def2-tzvp) = -2054.63658874
Low Freq. = 11.2925 cm^{-1}, 14.9219 cm^{-1}

108
2c

C	-4.330732000	2.645721000	1.924506000
C	-4.622517000	1.332150000	1.533379000
C	-3.694276000	0.583499000	0.772913000
C	-2.431141000	1.140665000	0.395890000
C	-2.176118000	2.497246000	0.750589000
C	-3.119529000	3.223536000	1.521605000
Sn	-1.201242000	-0.292370000	-0.953794000
N	0.322134000	-1.225461000	0.464897000

C	1.384962000	-0.664138000	-0.144155000
N	2.714960000	-1.103632000	0.058855000
C	3.006128000	-2.535465000	-0.186045000
C	-4.126179000	-0.800683000	0.350479000
C	-4.600161000	-1.024745000	-0.978397000
C	-5.024923000	-2.315153000	-1.350442000
C	-5.018800000	-3.396844000	-0.447970000
C	-4.608264000	-3.142445000	0.873520000
C	-4.181468000	-1.865342000	1.295127000
C	-4.686606000	0.103559000	-1.982111000
C	-3.861257000	-1.643705000	2.758523000
C	-0.981925000	3.302788000	0.305423000
C	-1.062274000	4.049225000	-0.903718000
C	-0.028592000	4.951827000	-1.226905000
C	1.087906000	5.141448000	-0.388958000
C	1.151544000	4.386475000	0.797537000
C	0.137582000	3.476611000	1.161665000
C	-2.261095000	3.919161000	-1.819667000
C	0.223819000	2.726709000	2.471451000
C	2.193234000	6.101952000	-0.769481000
C	-5.436188000	-4.782185000	-0.889290000
N	1.043562000	0.325845000	-0.999875000
C	1.879018000	0.697749000	-2.148070000
C	1.211206000	0.300160000	-3.479395000
C	0.362644000	-2.046312000	1.670339000
C	-0.023203000	-1.229445000	2.917890000
C	-0.528456000	-3.287735000	1.497594000
C	2.210479000	2.197833000	-2.138008000
H	1.409323000	-2.391748000	1.826417000
H	2.833449000	0.126645000	-2.081885000
H	-4.632166000	-3.963027000	1.611347000
H	-0.107190000	5.535092000	-2.160785000
H	-5.588320000	0.874738000	1.806401000
H	2.011311000	4.521277000	1.476727000
H	-5.385035000	-2.475970000	-2.381117000
H	-5.129762000	1.016617000	-1.534868000
H	-5.293351000	-0.191691000	-2.861192000
H	-3.678882000	0.392448000	-2.359076000
H	-3.097078000	-0.857224000	2.907209000
H	-3.509714000	-2.577506000	3.242350000
H	-4.765880000	-1.309617000	3.313222000
H	-2.893856000	4.269517000	1.789951000
H	-1.583698000	-2.995018000	1.312777000
H	-0.503121000	-3.927631000	2.404280000
H	-0.197308000	-3.898840000	0.632464000
H	-5.053879000	3.225388000	2.520908000
H	-0.594000000	3.022058000	3.163143000
H	1.188648000	2.915573000	2.983595000
H	0.115703000	1.634687000	2.315154000
H	0.701053000	-0.407017000	3.087088000
H	-0.035136000	-1.867371000	3.826996000
H	-1.027190000	-0.775007000	2.797558000

H	1.285525000	2.805865000	-2.206264000
H	2.868735000	2.463866000	-2.992187000
H	2.720903000	2.494213000	-1.199447000
H	-2.431032000	2.867063000	-2.130752000
H	-2.137624000	4.533508000	-2.733868000
H	-3.195873000	4.240024000	-1.312361000
H	2.921484000	5.624490000	-1.462465000
H	2.763009000	6.443822000	0.118522000
H	1.795907000	6.998737000	-1.288420000
H	-4.596075000	-5.313472000	-1.389293000
H	-6.272272000	-4.744956000	-1.617950000
H	-5.754989000	-5.407968000	-0.031150000
H	0.992421000	-0.787123000	-3.517934000
H	1.869235000	0.552057000	-4.338047000
H	0.251778000	0.842743000	-3.621125000
C	3.881540000	-3.234967000	0.877757000
H	2.011877000	-3.032434000	-0.147831000
C	3.989162000	-4.748375000	0.606310000
H	4.903537000	-2.795293000	0.875626000
H	3.463256000	-3.057105000	1.891253000
C	4.514249000	-5.043152000	-0.808573000
H	4.640877000	-5.222896000	1.371542000
H	2.982762000	-5.211764000	0.726505000
C	3.656286000	-4.340665000	-1.873752000
H	5.567134000	-4.686515000	-0.891197000
H	4.542401000	-6.139381000	-0.990455000
C	3.555212000	-2.826936000	-1.608964000
H	4.066919000	-4.523400000	-2.890325000
H	2.633228000	-4.783255000	-1.869356000
H	4.559568000	-2.366858000	-1.728120000
H	2.893137000	-2.346640000	-2.360316000
C	3.713805000	-0.084554000	0.457347000
C	5.045368000	-0.091497000	-0.327092000
H	3.221511000	0.885819000	0.229034000
C	5.935860000	1.101210000	0.074335000
H	5.598298000	-1.036405000	-0.127839000
H	4.845266000	-0.064716000	-1.418932000
C	6.198095000	1.141808000	1.588829000
H	6.894373000	1.060926000	-0.487056000
H	5.435447000	2.047423000	-0.235194000
C	4.878164000	1.135442000	2.377549000
H	6.804753000	0.253378000	1.881052000
H	6.806688000	2.033852000	1.852543000
C	3.993451000	-0.061757000	1.984086000
H	5.075480000	1.121432000	3.471362000
H	4.326024000	2.081878000	2.173687000
H	4.497696000	-1.003547000	2.290139000
H	3.026128000	-0.026538000	2.528629000

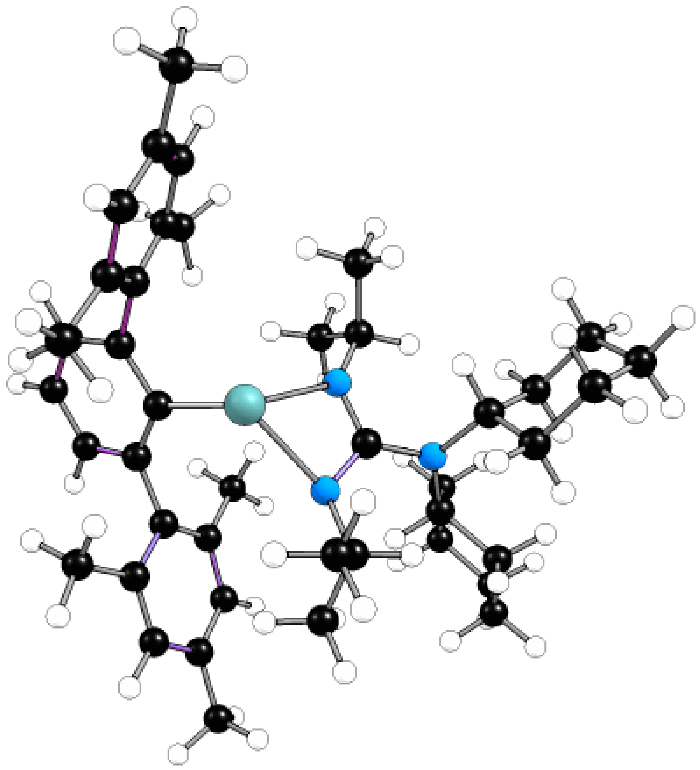

2c_tBu
SCF = -2131.21292992
H(0 K) = -2130.239445
H(298 K) = -2130.183394
G(298 K) = -2130.328730
SCF+D3BJ = -2131.50490946
PCM SCF (Benzene) = -2131.21576201
BS2 (def2-tzvp) = -2133.25057084
Low Freq. = 15.1207 cm^{-1}, 19.6456 cm^{-1}

114
2c_tBu

C	4.061788000	0.047623000	1.951269000
C	3.764326000	0.025859000	0.425908000
C	5.083612000	0.090603000	-0.379661000
C	5.946763000	1.299727000	0.033416000
C	6.224480000	1.322048000	1.544909000
C	4.911861000	1.271026000	2.342679000
N	2.801607000	-1.021640000	0.007412000
C	3.160796000	-2.454464000	-0.208757000
C	4.233832000	-3.042936000	0.738337000
C	4.405527000	-4.560259000	0.527608000
C	4.761039000	-4.901441000	-0.927065000
C	3.721590000	-4.307267000	-1.888169000
C	3.538761000	-2.792436000	-1.676086000
C	1.438054000	-0.639648000	-0.134587000
N	0.988722000	0.385503000	-0.918431000
C	1.562929000	1.052897000	-2.126940000
C	2.447955000	2.270410000	-1.758252000

N	0.406535000	-1.304753000	0.440715000
C	0.180498000	-2.010388000	1.725405000
C	-0.862440000	-3.112563000	1.434707000
Sn	-1.191054000	-0.371992000	-0.893520000
C	-2.502021000	1.031254000	0.460242000
C	-3.757538000	0.430534000	0.804957000
C	-4.693008000	1.111609000	1.619839000
C	-4.420485000	2.396718000	2.104612000
C	-3.229961000	3.026797000	1.720990000
C	-2.283369000	2.376576000	0.887568000
C	-4.202186000	-0.915268000	0.281143000
C	-4.620324000	-1.045761000	-1.078827000
C	-5.081644000	-2.292971000	-1.543485000
C	-5.168497000	-3.421548000	-0.705569000
C	-4.813306000	-3.258147000	0.645806000
C	-4.351845000	-2.028041000	1.159469000
C	-1.162809000	3.280291000	0.430968000
C	-1.362480000	4.059931000	-0.744211000
C	-0.416031000	5.046771000	-1.091258000
C	0.719182000	5.304928000	-0.300325000
C	0.892282000	4.531844000	0.864443000
C	-0.024394000	3.530075000	1.244381000
C	-4.612452000	0.134516000	-2.024676000
C	-5.623058000	-4.760096000	-1.243661000
C	-4.081439000	-1.912960000	2.645537000
C	-2.604829000	3.886485000	-1.592713000
C	1.730210000	6.357967000	-0.697184000
C	0.203306000	2.749194000	2.517109000
C	0.358629000	1.617749000	-2.919429000
C	-0.389179000	-0.990503000	2.743130000
C	1.394773000	-2.679080000	2.404020000
C	2.330713000	0.108363000	-3.072943000
H	-4.906855000	-4.116293000	1.333612000
H	-0.586636000	5.646809000	-2.001734000
H	-5.653244000	0.624296000	1.857470000
H	1.765089000	4.722633000	1.512733000
H	-5.397418000	-2.379562000	-2.597383000
H	-5.000390000	1.052932000	-1.539389000
H	-5.221909000	-0.075509000	-2.926180000
H	-3.580393000	0.369262000	-2.373577000
H	-3.238493000	-1.232597000	2.869834000
H	-3.863007000	-2.905172000	3.089646000
H	-4.966119000	-1.503712000	3.180947000
H	-3.027319000	4.061304000	2.046238000
H	-1.791372000	-2.690584000	0.998672000
H	-1.138416000	-3.653662000	2.363503000
H	-0.455843000	-3.847520000	0.709419000
H	-5.146585000	2.918713000	2.748722000
H	-0.690118000	2.762589000	3.175196000
H	1.063793000	3.149806000	3.089521000
H	0.405565000	1.682913000	2.289891000
H	0.398226000	-0.269409000	3.044362000

H	-0.746139000	-1.502236000	3.661594000
H	-1.228241000	-0.411046000	2.309329000
H	1.945053000	2.893655000	-0.992773000
H	2.618949000	2.898377000	-2.657377000
H	3.444436000	1.975847000	-1.381554000
H	-2.758857000	2.830534000	-1.895916000
H	-2.556042000	4.508585000	-2.508692000
H	-3.518016000	4.175476000	-1.029363000
H	2.564041000	5.914177000	-1.285702000
H	2.184555000	6.844543000	0.190455000
H	1.272983000	7.148711000	-1.326244000
H	-4.780930000	-5.305299000	-1.725022000
H	-6.413645000	-4.644712000	-2.013740000
H	-6.018494000	-5.413158000	-0.439377000
H	-0.298415000	0.801038000	-3.287701000
H	0.708723000	2.188125000	-3.804152000
H	-0.234734000	2.305408000	-2.284110000
H	2.220017000	-3.010205000	-0.016128000
H	5.213011000	-2.551972000	0.551078000
H	3.979282000	-2.842994000	1.796108000
H	5.182157000	-4.946266000	1.222919000
H	3.458639000	-5.079818000	0.804149000
H	5.766987000	-4.485282000	-1.166729000
H	4.838469000	-6.002047000	-1.062822000
H	4.001443000	-4.508349000	-2.944979000
H	2.743286000	-4.816725000	-1.726514000
H	4.475915000	-2.262031000	-1.957015000
H	2.745273000	-2.421070000	-2.351733000
H	3.229427000	0.976608000	0.229815000
H	5.674140000	-0.837360000	-0.222005000
H	4.862064000	0.137497000	-1.466171000
H	6.898897000	1.293242000	-0.540085000
H	5.422178000	2.240683000	-0.251903000
H	6.855339000	0.444456000	1.817943000
H	6.812995000	2.224915000	1.817173000
H	5.116838000	1.256865000	3.435076000
H	4.329670000	2.201223000	2.148134000
H	4.597519000	-0.876226000	2.255580000
H	3.099727000	0.060435000	2.506145000
H	1.081750000	-3.043386000	3.404322000
H	1.764078000	-3.557513000	1.842555000
H	2.226513000	-1.966982000	2.552179000
H	2.633900000	0.656732000	-3.989872000
H	3.244101000	-0.297193000	-2.604208000
H	1.689616000	-0.742772000	-3.383966000

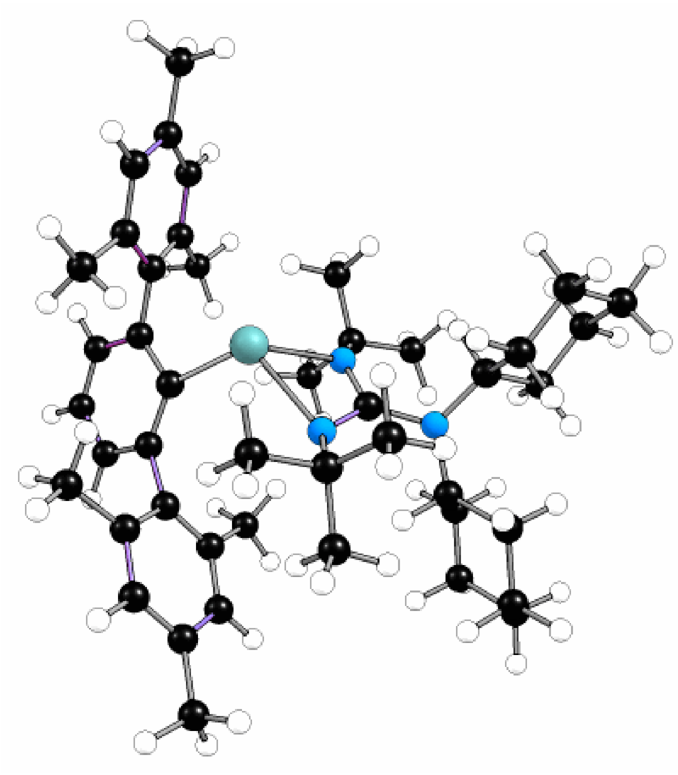

2c_TMS
SCF = -2634.04765706
H(0 K) = -2633.094700
H(298 K) = -2633.033959
G(298 K) = -2633.190960
SCF+D3BJ = -2634.35073772
PCM SCF (Benzene) = -2634.05038239
BS2 (def2-tzvp) = -2636.22553003
Low Freq. = 10.0944 cm^{-1}, 16.5191 cm^{-1}

114
2c_TMS

C	0.391272000	3.094623000	1.545014000
C	-0.807616000	3.192485000	0.790595000
C	-0.913394000	4.186971000	-0.224049000
C	0.176995000	5.047401000	-0.467158000
C	1.369815000	4.972403000	0.278099000
C	1.453296000	3.983093000	1.276801000
C	-2.047939000	2.409975000	1.151462000
C	-2.441649000	1.173381000	0.559750000
C	-3.769300000	0.700873000	0.819903000
C	-4.616779000	1.385694000	1.722219000
C	-4.177838000	2.547384000	2.371516000
C	-2.910942000	3.064323000	2.066984000
Sn	-1.280136000	-0.311091000	-0.792251000
N	0.310206000	-1.205099000	0.632471000
Si	-0.027856000	-2.178343000	2.075716000
C	1.444660000	-2.838075000	3.079184000
C	-4.359976000	-0.486299000	0.093120000
C	-4.704991000	-0.361383000	-1.288352000

C	-5.278122000	-1.457294000	-1.961304000
C	-5.539573000	-2.681625000	-1.316027000
C	-5.247898000	-2.767911000	0.056944000
C	-4.686080000	-1.692830000	0.778125000
C	-4.490487000	0.931631000	-2.044618000
C	-6.107869000	-3.858892000	-2.076678000
C	-4.504000000	-1.842573000	2.273728000
C	-2.199470000	4.374640000	-1.001840000
C	2.523713000	5.912091000	0.003033000
C	0.528534000	2.065062000	2.640564000
C	1.321198000	-0.646838000	-0.070042000
N	0.938043000	0.327760000	-0.932337000
Si	1.497385000	1.059708000	-2.454974000
C	2.175605000	-0.189850000	-3.717548000
N	2.657759000	-1.094695000	0.050750000
C	2.896382000	-2.559089000	-0.041131000
C	4.044950000	-3.132312000	0.817863000
C	4.097465000	-4.670300000	0.719626000
C	4.223303000	-5.154243000	-0.733892000
C	3.093239000	-4.578836000	-1.601744000
C	3.034745000	-3.043535000	-1.508363000
C	3.726566000	-0.075091000	0.170236000
C	4.207662000	0.099541000	1.636836000
C	5.214799000	1.254054000	1.778523000
C	6.420817000	1.054982000	0.846703000
C	5.962019000	0.903929000	-0.612355000
C	4.930254000	-0.229172000	-0.789799000
C	2.739358000	2.471243000	-2.215205000
C	-0.045555000	1.841402000	-3.234365000
C	-1.082305000	-3.643793000	1.495860000
C	-1.002852000	-1.115146000	3.308650000
H	-5.476459000	-3.703018000	0.596905000
H	0.078145000	5.818449000	-1.250725000
H	-5.636676000	1.005687000	1.898954000
H	2.370414000	3.909372000	1.886611000
H	-5.536921000	-1.345146000	-3.028239000
H	-4.789301000	1.814737000	-1.444381000
H	-5.065673000	0.935603000	-2.991959000
H	-3.419436000	1.079772000	-2.312501000
H	-3.651127000	-1.254692000	2.658333000
H	-4.356014000	-2.905057000	2.554776000
H	-5.405694000	-1.486230000	2.819321000
H	-2.579770000	4.011300000	2.525841000
H	-1.993897000	-3.303447000	0.960183000
H	-1.406289000	-4.265488000	2.357825000
H	-0.513350000	-4.296681000	0.800630000
H	-4.834847000	3.069752000	3.085741000
H	-0.320719000	2.109683000	3.353439000
H	1.468969000	2.203790000	3.210863000
H	0.526388000	1.040145000	2.215063000
H	-0.312731000	-0.498107000	3.920238000
H	-1.569903000	-1.768503000	4.005772000

H	-1.715916000	-0.426586000	2.812564000
H	2.398321000	3.138644000	-1.397031000
H	2.786693000	3.071217000	-3.149387000
H	3.768586000	2.130745000	-1.988847000
H	-2.583704000	3.416641000	-1.407115000
H	-2.060524000	5.081931000	-1.843848000
H	-3.007429000	4.774027000	-0.351376000
H	3.172728000	5.527410000	-0.815208000
H	3.167883000	6.041218000	0.896600000
H	2.168185000	6.914741000	-0.311902000
H	-5.306914000	-4.410444000	-2.617266000
H	-6.846627000	-3.535464000	-2.838974000
H	-6.605436000	-4.583241000	-1.400445000
H	-0.814276000	1.087148000	-3.507070000
H	0.237108000	2.374089000	-4.167704000
H	-0.503768000	2.585665000	-2.550697000
H	1.964004000	-3.025664000	0.341985000
H	5.021615000	-2.726746000	0.477439000
H	3.925332000	-2.826357000	1.875366000
H	4.940307000	-5.053754000	1.334415000
H	3.170518000	-5.099684000	1.166198000
H	5.206478000	-4.828182000	-1.145703000
H	4.222123000	-6.265083000	-0.774332000
H	3.212867000	-4.891463000	-2.661612000
H	2.119811000	-5.003318000	-1.262741000
H	3.955299000	-2.609937000	-1.957736000
H	2.179695000	-2.655889000	-2.099488000
H	3.230116000	0.881686000	-0.097696000
H	5.440946000	-1.198828000	-0.609336000
H	4.571460000	-0.256337000	-1.839917000
H	6.831552000	0.722288000	-1.280565000
H	5.514948000	1.867853000	-0.946551000
H	6.979383000	0.141720000	1.157816000
H	7.133609000	1.902732000	0.940328000
H	5.545278000	1.343150000	2.836059000
H	4.712541000	2.216553000	1.526707000
H	4.683535000	-0.838554000	1.992856000
H	3.320279000	0.272810000	2.281870000
H	1.044906000	-3.179667000	4.058918000
H	1.958485000	-3.702293000	2.614797000
H	2.195865000	-2.048643000	3.286496000
H	2.388241000	0.336714000	-4.673421000
H	3.112070000	-0.684802000	-3.392080000
H	1.428001000	-0.982851000	-3.930258000

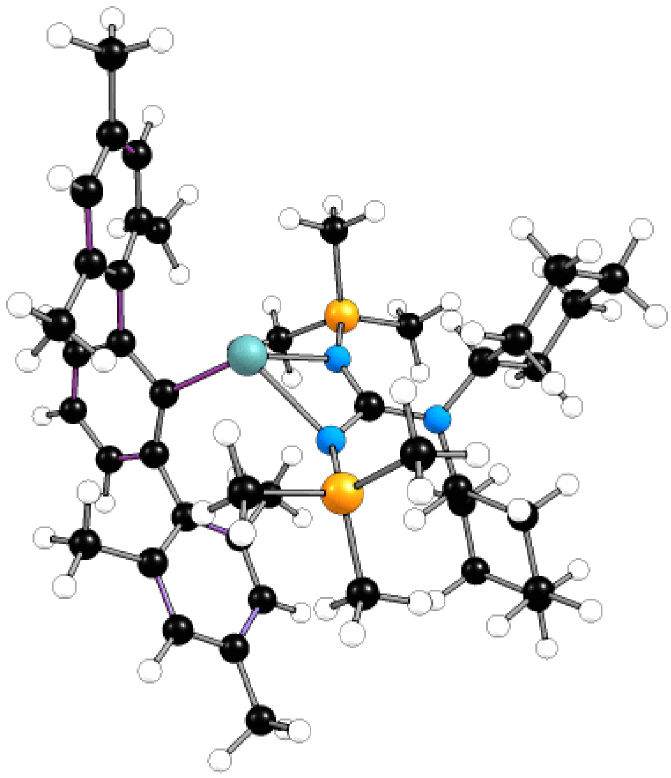

3a
SCF = -4802.32635554
H(0 K) = -4801.500346
H(298 K) = -4801.441216
G(298 K) = -4801.594662
SCF+D3BJ = -4802.60703393
PCM SCF (Benzene) = -4802.33133186
BS2 (def2-tzvp) = -4804.56048556
Low Freq. = 17.4073 cm^{-1}, 19.4820 cm^{-1}

101
3a

C	-2.843504000	3.229297000	0.623106000
C	-2.531368000	2.634970000	-0.634215000
C	-1.922471000	3.416075000	-1.657668000
C	-1.599617000	4.761645000	-1.394478000
C	-1.863291000	5.361971000	-0.147031000
C	-2.484495000	4.576823000	0.842005000
C	-3.052199000	1.264086000	-0.971111000
C	-2.420985000	0.028061000	-0.650639000
C	-3.080112000	-1.196817000	-0.955982000
C	-4.327300000	-1.163014000	-1.626923000
C	-4.934998000	0.048440000	-1.975219000
C	-4.300462000	1.249989000	-1.640582000
Sn	-0.581218000	0.011689000	0.605377000
N	1.220448000	-1.154244000	-0.075821000
C	1.982745000	-0.043355000	-0.159616000
N	3.395319000	-0.072295000	-0.261829000
Si	4.127118000	-0.133436000	-1.916599000

C	2.735651000	-0.029149000	-3.194957000
C	-2.593789000	-2.575420000	-0.599264000
C	-1.988464000	-3.380447000	-1.605800000
C	-1.708604000	-4.732740000	-1.327474000
C	-2.012794000	-5.316471000	-0.081413000
C	-2.625190000	-4.505646000	0.892534000
C	-2.938963000	-3.149571000	0.658866000
C	-1.675520000	-2.806681000	-2.971833000
H	-2.600652000	-2.521124000	-3.516356000
C	-3.655137000	-2.353062000	1.724743000
H	-4.499494000	-1.769979000	1.303036000
C	-1.650430000	2.824959000	-3.025306000
H	-2.593288000	2.574142000	-3.556452000
C	-3.575668000	2.466375000	1.702373000
H	-4.435593000	1.898773000	1.291215000
Se	-0.750270000	0.044800000	3.019257000
N	1.258586000	1.094182000	-0.095720000
C	1.829867000	2.440869000	0.003719000
C	1.198390000	3.210243000	1.174563000
C	5.322487000	1.322227000	-2.136581000
C	5.095318000	-1.734971000	-2.231027000
Si	4.375902000	0.024052000	1.268946000
C	3.338556000	-0.585235000	2.718912000
C	4.990625000	1.782221000	1.649859000
C	5.916042000	-1.068147000	1.088540000
C	1.753446000	-2.511108000	0.084015000
C	1.030512000	-3.247121000	1.222484000
H	-1.125303000	-3.536723000	-3.598302000
H	-4.052324000	-3.018943000	2.516330000
H	-1.080979000	3.530780000	-3.662282000
H	-3.953730000	3.153666000	2.485017000
C	1.696788000	-3.318815000	-1.224108000
C	1.724957000	3.230584000	-1.311637000
H	-4.825186000	-2.119196000	-1.856310000
H	-5.909342000	0.056352000	-2.489492000
H	-4.777384000	2.214216000	-1.880587000
H	-1.245797000	-5.352358000	-2.114596000
C	-1.675490000	-6.762515000	0.206238000
H	-2.893907000	-4.945740000	1.867834000
H	-1.064197000	-1.883189000	-2.896856000
H	-2.969937000	-1.623321000	2.216507000
H	-1.132708000	5.361993000	-2.194017000
C	-1.477318000	6.799068000	0.123939000
H	-2.726430000	5.032164000	1.817322000
H	-1.075058000	1.878325000	-2.955277000
H	-2.907381000	1.727166000	2.203509000
H	2.826688000	-2.400435000	0.361578000
H	0.647780000	-3.474980000	-1.540955000
H	2.160224000	-4.317990000	-1.081713000
H	2.237032000	-2.807163000	-2.044620000
H	1.057780000	-2.664665000	2.164744000
H	1.500745000	-4.237024000	1.398756000

H	-0.035317000	-3.421834000	0.967185000
H	2.911571000	2.309197000	0.217937000
H	2.250371000	2.714929000	-2.139703000
H	2.184378000	4.234581000	-1.193488000
H	0.667005000	3.375808000	-1.601249000
H	0.121012000	3.404866000	0.989180000
H	1.700085000	4.191821000	1.305179000
H	1.275330000	2.639649000	2.121950000
H	5.476698000	2.265547000	0.777781000
H	5.745313000	1.724564000	2.464461000
H	4.175526000	2.445936000	2.003918000
H	5.662151000	-2.118315000	0.838386000
H	6.447165000	-1.073385000	2.064951000
H	6.633867000	-0.691957000	0.330691000
H	2.328896000	-0.122108000	2.777911000
H	3.872636000	-0.339150000	3.661938000
H	3.199280000	-1.685160000	2.692927000
H	4.814455000	2.298536000	-1.996353000
H	5.751940000	1.304936000	-3.161378000
H	6.172729000	1.274425000	-1.424525000
H	6.025339000	-1.798751000	-1.632627000
H	5.386324000	-1.770305000	-3.303495000
H	4.489309000	-2.638937000	-2.018393000
H	2.034465000	-0.883655000	-3.108237000
H	3.177831000	-0.054169000	-4.213624000
H	2.140664000	0.900648000	-3.102372000
H	-2.143776000	7.266941000	0.876771000
H	-0.440281000	6.866090000	0.521427000
H	-1.512047000	7.414193000	-0.798484000
H	-2.386529000	-7.212714000	0.928573000
H	-1.684360000	-7.377539000	-0.716745000
H	-0.659569000	-6.854925000	0.650413000

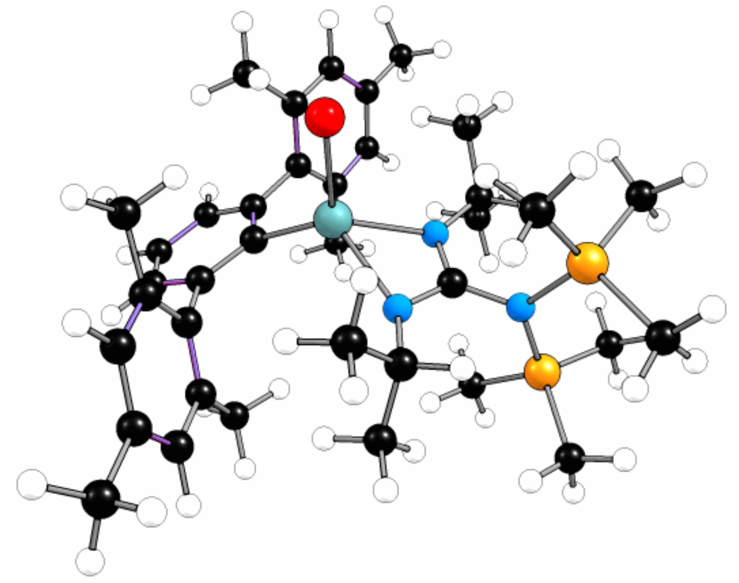

4a_one
SCF = -2475.89737004
H(0 K) = -2475.069368
H(298 K) = -2475.011273
G(298 K) = -2475.161451
SCF+D3 = -2476.16737442
PCM SCF (Benzene) = -2475.90322623
BS2 (def2-tzvp) = -2477.93463772
Low Freq. = 11.7692cm-1, 21.3457cm-1

101
SnSi2C37N3H57O

C	-2.097469000	3.511401000	-1.042025000
C	-2.095666000	3.004768000	0.290255000
C	-1.490654000	3.756022000	1.338728000
C	-0.906215000	5.001614000	1.040987000
C	-0.901142000	5.529836000	-0.266673000
C	-1.493396000	4.764493000	-1.287592000
C	-2.908488000	1.784324000	0.622766000
C	-2.500867000	0.443898000	0.387845000
C	-3.413057000	-0.628576000	0.595049000
C	-4.701161000	-0.339047000	1.106637000
C	-5.092242000	0.974924000	1.393098000
C	-4.203042000	2.027418000	1.143058000
C	-3.139446000	-2.070724000	0.266450000
C	-3.235922000	-2.533408000	-1.080212000
C	-3.112305000	-3.919247000	-1.325199000
C	-2.919295000	-4.856738000	-0.294253000
C	-2.852200000	-4.376030000	1.029040000
C	-2.967323000	-3.005273000	1.329616000
C	-3.485401000	-1.595802000	-2.238740000
H	-2.543823000	-1.071076000	-2.547654000
C	-2.942457000	-2.551629000	2.775775000
H	-2.567138000	-3.355807000	3.439924000
Sn	-0.592233000	0.063435000	-0.660234000
N	0.989314000	-1.262088000	0.193735000
C	1.236216000	-2.672019000	0.479311000
C	0.683231000	-3.079677000	1.855549000
C	-1.480026000	3.234835000	2.760204000
H	-0.884401000	3.892792000	3.423808000
C	-2.732419000	2.750284000	-2.185098000
H	-2.100637000	1.884147000	-2.502017000
N	1.407567000	0.929087000	-0.088868000
C	2.184652000	2.083817000	-0.550934000
C	2.263851000	3.177880000	0.526983000
O	-0.634979000	-0.044477000	-2.556555000
C	1.930214000	-0.298196000	0.118117000
N	3.319204000	-0.557910000	0.185974000
Si	4.114297000	-1.194771000	-1.322579000
C	5.832925000	-0.407206000	-1.486799000
Si	4.188572000	-0.264556000	1.743112000
C	5.386622000	1.203293000	1.656431000

C	2.900382000	0.074814000	3.085536000
C	5.185219000	-1.812475000	2.198581000
H	-2.504821000	3.163705000	3.182299000
H	-2.866321000	3.405736000	-3.068495000
H	-3.957108000	-2.272889000	3.133507000
H	-3.865120000	-2.153593000	-3.118039000
C	0.668550000	-3.556207000	-0.642894000
C	3.068310000	-0.717601000	-2.819128000
C	4.374288000	-3.075978000	-1.282315000
C	1.609886000	2.640327000	-1.865309000
H	-4.512782000	3.069943000	1.321724000
H	-6.101088000	1.181160000	1.785135000
H	-5.408042000	-1.171282000	1.255639000
H	-0.446421000	5.581583000	1.859573000
C	-0.253962000	6.864786000	-0.561071000
H	-1.506702000	5.160466000	-2.317242000
H	-1.052602000	2.211561000	2.813197000
H	-3.723749000	2.339314000	-1.903851000
H	-2.714664000	-5.091810000	1.857638000
C	-2.768387000	-6.331306000	-0.595500000
H	-3.191752000	-4.274489000	-2.366659000
H	-2.307549000	-1.653640000	2.920489000
H	-4.225310000	-0.811308000	-1.978655000
H	3.218871000	1.716219000	-0.745042000
H	1.253121000	3.571736000	0.756908000
H	2.889075000	4.026125000	0.175849000
H	2.706101000	2.794429000	1.468467000
H	1.435699000	1.835984000	-2.606741000
H	2.297248000	3.397621000	-2.297412000
H	0.635053000	3.138608000	-1.682809000
H	2.341093000	-2.800909000	0.507704000
H	1.126210000	-2.466070000	2.665734000
H	0.910224000	-4.145473000	2.068166000
H	-0.416539000	-2.955166000	1.885263000
H	-0.436820000	-3.471228000	-0.694611000
H	0.917874000	-4.623308000	-0.465326000
H	1.073179000	-3.258633000	-1.631082000
H	4.889772000	-3.409963000	-0.358599000
H	5.012380000	-3.366548000	-2.145268000
H	3.423776000	-3.639229000	-1.370319000
H	5.784690000	0.700830000	-1.505207000
H	6.275745000	-0.740062000	-2.450437000
H	6.535048000	-0.712952000	-0.683703000
H	1.972005000	-0.854866000	-2.689994000
H	3.385435000	-1.332795000	-3.688458000
H	3.232432000	0.345811000	-3.090056000
H	4.532006000	-2.704102000	2.298072000
H	5.695683000	-1.654707000	3.172985000
H	5.971616000	-2.045495000	1.450945000
H	6.220339000	1.027247000	0.948049000
H	5.831783000	1.368824000	2.661618000
H	4.876142000	2.140842000	1.356864000

H	2.237295000	0.921770000	2.815889000
H	3.415908000	0.331259000	4.035212000
H	2.258320000	-0.808988000	3.275546000
H	-0.620931000	7.296549000	-1.513870000
H	-0.446089000	7.599937000	0.247734000
H	0.850431000	6.763441000	-0.649431000
H	-3.277018000	-6.610008000	-1.540583000
H	-1.696778000	-6.608900000	-0.707963000
H	-3.182451000	-6.960871000	0.218726000

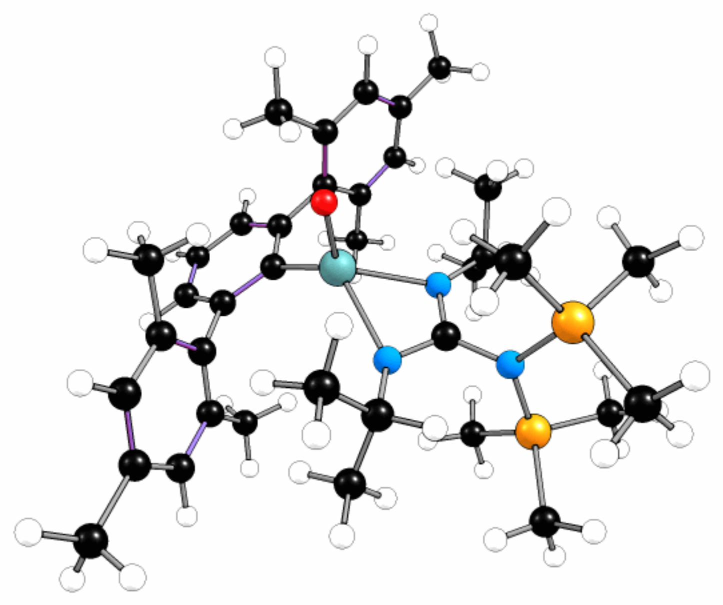

4a

SCF = -2475.94140267

H(0 K) = -2475.116092

H(298 K) = -2475.056347

G(298 K) = -2475.214210

SCF+D3 = -2476.19955298

PCM SCF (Benzene) = -2475.94477493

BS2 (def2-tzvp) = -2477.98201422

Low Freq. = 10.8406cm-1, 14.2866cm-1

101

SnSi2C37N3H57O

C	3.782925000	0.127535000	-1.802589000
C	3.538824000	0.793079000	-0.569418000
C	4.269002000	0.425538000	0.596190000
C	5.198374000	-0.628285000	0.508496000
C	5.427383000	-1.332667000	-0.691163000
C	4.710082000	-0.935003000	-1.834719000
C	2.642008000	1.999644000	-0.554982000
C	1.239044000	1.972298000	-0.312981000
C	0.468194000	3.165244000	-0.383066000
C	1.128408000	4.380852000	-0.687089000
C	2.509593000	4.423810000	-0.911469000
C	3.256225000	3.240449000	-0.846592000

Sn	0.283497000	0.067783000	0.172554000
N	-0.514298000	-1.316727000	-1.189416000
C	-0.178702000	-2.187493000	-2.306694000
C	1.181736000	-2.873771000	-2.114018000
C	-1.016741000	3.265154000	-0.169337000
C	-1.507729000	3.814630000	1.048716000
C	-2.885183000	4.085813000	1.169455000
C	-3.789702000	3.844323000	0.116824000
C	-3.280539000	3.292220000	-1.074819000
C	-1.913454000	2.994944000	-1.240843000
C	-0.573091000	4.138671000	2.196096000
H	0.077924000	5.008229000	1.961699000
C	-1.421837000	2.421602000	-2.552012000
H	-2.212044000	2.465531000	-3.327098000
C	4.062036000	1.150887000	1.905775000
H	4.808477000	0.833744000	2.660872000
C	3.123064000	0.589913000	-3.084226000
H	3.541844000	1.564025000	-3.418761000
O	1.349422000	-0.732659000	1.677517000
Si	1.423013000	-2.215567000	2.498430000
C	-0.216908000	-3.160649000	2.440413000
N	-1.738499000	-0.429395000	0.494843000
C	-1.747983000	-1.431022000	-0.492840000
N	-2.640171000	-2.310376000	-0.742168000
Si	-4.075277000	-3.243991000	-0.746792000
C	-3.675483000	-4.978588000	-1.417318000
C	1.853351000	-1.793590000	4.294878000
C	2.790686000	-3.292356000	1.755279000
C	-5.353201000	-2.436436000	-1.903273000
C	-4.866268000	-3.483201000	0.971911000
C	-2.699784000	-0.385205000	1.593693000
C	-4.033858000	0.245632000	1.155986000
H	-1.140648000	4.383729000	3.115654000
H	-1.129103000	1.353900000	-2.444788000
H	3.050700000	0.926870000	2.305928000
H	3.276654000	-0.140608000	-3.902731000
C	-2.101906000	0.364534000	2.791951000
C	-0.285518000	-1.467273000	-3.664094000
H	0.527111000	5.302045000	-0.749279000
H	3.004084000	5.380076000	-1.145597000
H	4.342244000	3.257363000	-1.032317000
H	-3.260119000	4.513373000	2.114945000
C	-5.265304000	4.135408000	0.272059000
H	-3.969282000	3.092938000	-1.913129000
H	0.110800000	3.295803000	2.427116000
H	-0.531528000	2.963771000	-2.932738000
H	5.770266000	-0.902894000	1.411352000
C	6.403237000	-2.486739000	-0.738472000
H	4.890994000	-1.452079000	-2.792280000
H	4.136995000	2.251680000	1.786608000
H	2.032447000	0.750258000	-2.963887000
H	2.814476000	-1.241440000	4.357124000

H	1.950905000	-2.715106000	4.908210000
H	1.068588000	-1.159593000	4.758543000
H	2.556121000	-3.578713000	0.708999000
H	2.920580000	-4.226330000	2.343388000
H	3.758587000	-2.750174000	1.743577000
H	-1.023461000	-2.619118000	2.976823000
H	-0.099279000	-4.151753000	2.930019000
H	-0.565295000	-3.339110000	1.401259000
H	-2.904064000	-1.437724000	1.906035000
H	-1.149675000	-0.096748000	3.125609000
H	-2.806212000	0.363268000	3.649047000
H	-1.903175000	1.423924000	2.522950000
H	-3.881385000	1.306061000	0.871404000
H	-4.781388000	0.200102000	1.976875000
H	-4.448200000	-0.289279000	0.279312000
H	-0.972504000	-2.971125000	-2.280425000
H	0.462480000	-0.650418000	-3.748817000
H	-1.294813000	-1.025681000	-3.789598000
H	-0.112010000	-2.170865000	-4.506441000
H	1.410103000	-3.551718000	-2.963274000
H	1.188142000	-3.477504000	-1.183844000
H	2.007703000	-2.133121000	-2.044070000
H	-5.673525000	-1.440103000	-1.531817000
H	-6.261468000	-3.070205000	-1.995845000
H	-4.929994000	-2.295915000	-2.920185000
H	-4.141206000	-3.931183000	1.684468000
H	-5.739790000	-4.168382000	0.908420000
H	-5.220825000	-2.525033000	1.405429000
H	-3.268885000	-4.921555000	-2.449328000
H	-4.577979000	-5.627102000	-1.440356000
H	-2.913311000	-5.481837000	-0.784823000
H	-5.726312000	4.436547000	-0.690654000
H	-5.813150000	3.234122000	0.626298000
H	-5.449095000	4.941085000	1.011360000
H	6.725988000	-2.709951000	-1.775218000
H	7.308952000	-2.281286000	-0.131121000
H	5.942886000	-3.412776000	-0.328734000

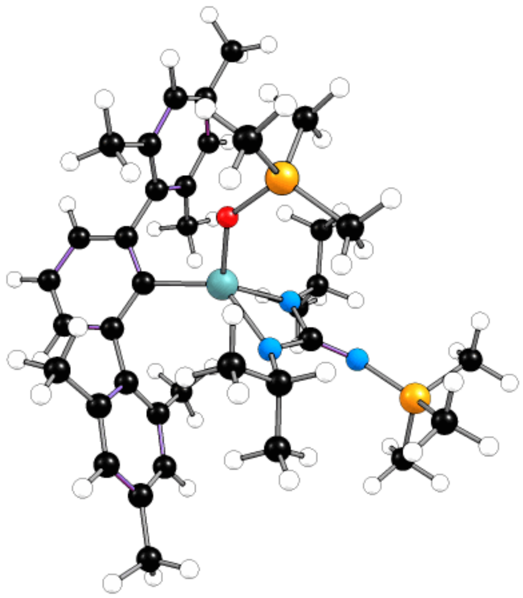

4b_one
SCF = -2711.56669075
H(0 K) = -2710.572125
H(298 K) = -2710.506658
G(298 K) = -2710.670975
SCF+D3 = -2711.88651425
PCM SCF (Benzene) = -2711.57161123
BS2 (def2-tzvp) = -2713.85407834
Low Freq. = 16.1987cm-1, 19.8845cm-1

119
SnSi2C43N3H69O

C	-2.311778000	-3.199363000	-1.029262000
C	-2.382014000	-2.567282000	0.250062000
C	-2.059250000	-3.288039000	1.440581000
C	-1.631227000	-4.627180000	1.324135000
C	-1.525977000	-5.246712000	0.070606000
C	-1.864333000	-4.537626000	-1.089806000
C	-2.972627000	-1.188134000	0.376230000
C	-2.248376000	0.029208000	0.233440000
C	-2.926894000	1.271821000	0.378180000
C	-4.310121000	1.275668000	0.681463000
C	-5.025793000	0.081512000	0.824479000
C	-4.357033000	-1.138261000	0.670889000
Sn	-0.210362000	0.002136000	-0.680533000
N	1.623389000	1.126179000	0.011014000
C	2.349390000	-0.012154000	0.054244000
N	3.765290000	-0.033212000	0.048755000
Si	4.630725000	0.119621000	1.628141000
C	3.359993000	-0.057193000	3.016806000
C	-2.288739000	2.627503000	0.231663000
C	-1.831353000	3.308046000	1.400704000
C	-1.370466000	4.635070000	1.272722000
C	-1.364483000	5.281752000	0.028050000
C	-1.829385000	4.610296000	-1.110630000

C	-2.310096000	3.284477000	-1.036071000
C	-1.908903000	2.665745000	2.791095000
C	-2.986288000	3.355859000	3.656541000
C	-2.929070000	2.640936000	-2.282673000
C	-4.261727000	3.342984000	-2.632078000
C	-2.232400000	-2.673190000	2.835553000
C	-3.360657000	-3.382441000	3.616464000
C	-2.836740000	-2.524636000	-2.302769000
C	-4.233156000	-3.094244000	-2.647711000
O	-0.122272000	-0.019232000	-2.577678000
N	1.590706000	-1.128229000	0.052606000
C	2.128129000	-2.486569000	-0.041534000
C	1.679083000	-3.164348000	-1.346764000
C	5.925727000	-1.257892000	1.775751000
C	5.518658000	1.784642000	1.820762000
Si	4.606706000	-0.242437000	-1.553176000
C	3.434098000	0.230687000	-2.953891000
C	5.242390000	-2.013103000	-1.822683000
C	6.126650000	0.893992000	-1.590982000
C	2.201982000	2.445029000	-0.269494000
C	1.552741000	3.078950000	-1.509915000
C	-0.550936000	2.631264000	3.519544000
C	-1.973810000	2.599151000	-3.493200000
C	-0.925889000	-2.648024000	3.654184000
C	-1.875982000	-2.615495000	-3.504099000
C	2.112484000	3.385122000	0.943419000
C	1.777984000	-3.339131000	1.186501000
H	-1.101581000	-2.180954000	4.646418000
H	-0.136045000	-2.065892000	3.139153000
H	-4.319325000	-3.359661000	3.058658000
H	-3.526273000	-2.891987000	4.599224000
H	-3.113436000	-4.447895000	3.811175000
H	-4.820755000	2.245309000	0.794067000
H	-6.103809000	0.101306000	1.050361000
H	-4.905268000	-2.087765000	0.777843000
H	-1.023206000	5.178388000	2.166655000
H	-1.003447000	6.319801000	-0.052452000
H	-1.836455000	5.131314000	-2.081424000
H	-2.228155000	1.612290000	2.650483000
H	0.209864000	2.083128000	2.928377000
H	-0.652229000	2.124686000	4.502586000
H	-0.156594000	3.651521000	3.713972000
H	-2.732807000	4.420788000	3.846199000
H	-3.081512000	2.852075000	4.641949000
H	-3.980134000	3.331810000	3.164255000
H	-3.172049000	1.587764000	-2.029389000
H	-2.514539000	2.210575000	-4.382338000
H	-1.122192000	1.911046000	-3.300112000
H	-1.591414000	3.608487000	-3.757925000
H	-4.097273000	4.402866000	-2.922547000
H	-4.968436000	3.336989000	-1.775979000
H	-4.755928000	2.834278000	-3.486619000

H	-1.382233000	-5.198689000	2.233143000
H	-1.189971000	-6.293801000	-0.000960000
H	-1.803758000	-5.040243000	-2.068114000
H	-2.545332000	-1.618016000	2.695876000
H	-2.967612000	-1.444371000	-2.082952000
H	-1.609205000	-3.664934000	-3.753771000
H	-0.958785000	-2.019212000	-3.312475000
H	-2.362611000	-2.180664000	-4.402598000
H	-4.661849000	-2.566669000	-3.525907000
H	-4.944561000	-2.984178000	-1.802842000
H	-4.175215000	-4.175416000	-2.898059000
H	3.281173000	2.274025000	-0.486969000
H	1.055920000	3.621429000	1.178338000
H	2.634700000	4.342046000	0.731146000
H	2.574212000	2.934284000	1.844937000
H	1.573781000	2.389206000	-2.375149000
H	2.076863000	4.019122000	-1.781983000
H	0.492130000	3.334661000	-1.310958000
H	3.234662000	-2.380155000	-0.066276000
H	2.142206000	-2.868624000	2.122230000
H	2.246175000	-4.342597000	1.104835000
H	0.683528000	-3.483845000	1.272049000
H	0.579624000	-3.311723000	-1.357851000
H	2.152884000	-4.163216000	-1.450497000
H	1.941394000	-2.550895000	-2.231159000
H	5.841222000	-2.387083000	-0.967497000
H	5.900225000	-2.018418000	-2.719191000
H	4.421885000	-2.733791000	-2.012542000
H	5.856961000	1.957629000	-1.427717000
H	6.595459000	0.816630000	-2.595777000
H	6.901252000	0.612834000	-0.847645000
H	2.371404000	-0.078308000	-2.819697000
H	3.804181000	-0.232879000	-3.893714000
H	3.436212000	1.329495000	-3.108461000
H	5.464050000	-2.264114000	1.699591000
H	6.428316000	-1.188751000	2.764503000
H	6.716693000	-1.183226000	1.000823000
H	6.321838000	1.925433000	1.070204000
H	5.989127000	1.832158000	2.826957000
H	4.819231000	2.640919000	1.736830000
H	2.557519000	0.704689000	2.940862000
H	3.863923000	0.075188000	3.997587000
H	2.877914000	-1.055437000	3.011572000
H	-0.528817000	-3.669640000	3.835581000

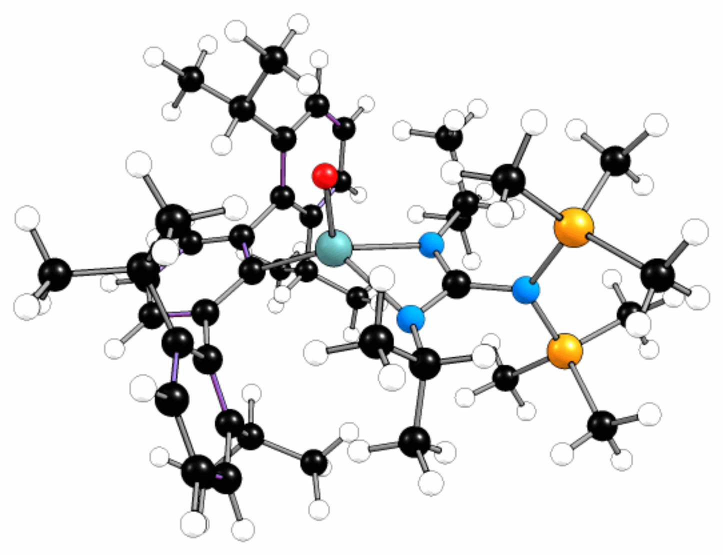

4b
SCF = -2711.60838275
H(0 K) = -2710.615795
H(298 K) = -2710.549302
G(298 K) = -2710.718638
SCF+D3 = -2711.92011769
PCM SCF (Benzene) = -2711.61174139
BS2 (def2-tzvp) = -2713.89967988
Low Freq. = 11.4971cm-1, 18.1889cm-1

119
SnSi2C43N3H69O

Sn	-0.034934000	-0.348853000	-0.165448000
Si	-0.886765000	-3.342851000	-1.651672000
Si	5.355692000	-1.668738000	-0.029691000
O	-1.088782000	-1.726757000	-1.170930000
N	1.903779000	-0.305886000	-0.980361000
N	1.471400000	-1.107346000	1.089579000
N	3.691261000	-1.381018000	0.266738000
C	-1.375893000	1.221522000	0.563190000
C	-0.836409000	2.513741000	0.841552000
C	-1.649707000	3.454659000	1.517596000
H	-1.229341000	4.448410000	1.737315000
C	-2.972260000	3.158815000	1.867186000
H	-3.591072000	3.908064000	2.385969000
C	-3.516352000	1.920399000	1.508147000
H	-4.576369000	1.703965000	1.712380000
C	-2.742920000	0.928173000	0.857648000
C	0.496872000	3.038445000	0.359345000
C	0.527614000	3.730509000	-0.893152000
C	1.725409000	4.361088000	-1.289252000
H	1.761454000	4.900715000	-2.248737000
C	2.867057000	4.325394000	-0.478211000
H	3.793495000	4.824099000	-0.805054000
C	2.825886000	3.656035000	0.750866000
H	3.724888000	3.639145000	1.385433000

C	1.651411000	3.014600000	1.197492000
C	-0.719358000	3.894398000	-1.772086000
H	-1.470442000	3.149549000	-1.434543000
C	-0.455008000	3.629834000	-3.267376000
H	0.222276000	4.388521000	-3.713077000
H	-1.405485000	3.668372000	-3.839502000
H	0.002753000	2.633882000	-3.434508000
C	-1.343270000	5.295236000	-1.577044000
H	-1.610782000	5.481919000	-0.517637000
H	-2.266107000	5.403777000	-2.185515000
H	-0.634420000	6.091389000	-1.889798000
C	1.623192000	2.393666000	2.596379000
H	0.789734000	1.658600000	2.613236000
C	2.917094000	1.638221000	2.955036000
H	3.778041000	2.329263000	3.075421000
H	3.183453000	0.885284000	2.186726000
H	2.795843000	1.112103000	3.924875000
C	1.315526000	3.468026000	3.665465000
H	1.268989000	3.011773000	4.677099000
H	0.348104000	3.975970000	3.478969000
H	2.107970000	4.246548000	3.680306000
C	-3.505557000	-0.309125000	0.443808000
C	-4.040158000	-0.369121000	-0.878869000
C	-4.876718000	-1.449552000	-1.225171000
H	-5.296125000	-1.502688000	-2.242512000
C	-5.198579000	-2.446564000	-0.295773000
H	-5.859686000	-3.279981000	-0.583090000
C	-4.681063000	-2.374978000	1.004006000
H	-4.944235000	-3.157966000	1.732116000
C	-3.836405000	-1.315979000	1.399353000
C	-3.801940000	0.735723000	-1.912687000
H	-3.011007000	1.404318000	-1.514271000
C	-3.295439000	0.189213000	-3.262016000
H	-2.399290000	-0.446177000	-3.116112000
H	-3.033874000	1.024231000	-3.945848000
H	-4.066058000	-0.423036000	-3.777014000
C	-5.071091000	1.597975000	-2.093438000
H	-4.887676000	2.424886000	-2.812186000
H	-5.399145000	2.046727000	-1.133084000
H	-5.915543000	0.991821000	-2.485579000
C	-3.346789000	-1.263156000	2.850928000
H	-2.489559000	-0.555793000	2.888583000
C	-2.848009000	-2.630295000	3.362518000
H	-3.681052000	-3.354919000	3.480132000
H	-2.372146000	-2.521437000	4.359059000
H	-2.106881000	-3.084116000	2.674103000
C	-4.439272000	-0.714132000	3.796820000
H	-4.765945000	0.304548000	3.508006000
H	-4.066513000	-0.666504000	4.842071000
H	-5.336018000	-1.369814000	3.785895000
C	-1.804714000	-3.533405000	-3.296412000
H	-2.876495000	-3.268620000	-3.184734000

H	-1.746807000	-4.582589000	-3.658195000
H	-1.371539000	-2.880089000	-4.082547000
C	-1.666703000	-4.485358000	-0.360631000
H	-1.103919000	-4.462913000	0.596210000
H	-1.677298000	-5.537710000	-0.717590000
H	-2.712013000	-4.178084000	-0.150275000
C	0.935825000	-3.803111000	-1.885276000
H	1.409240000	-3.222341000	-2.703820000
H	1.024800000	-4.880271000	-2.145199000
H	1.528207000	-3.630269000	-0.962939000
C	2.558433000	-0.136513000	-2.271783000
H	3.198805000	-1.036949000	-2.425896000
C	1.519437000	-0.100340000	-3.400584000
H	0.896982000	-1.017150000	-3.412407000
H	2.012135000	0.000506000	-4.389319000
H	0.841310000	0.772219000	-3.277542000
C	3.469691000	1.103117000	-2.308419000
H	2.875752000	2.030875000	-2.179586000
H	4.018358000	1.165365000	-3.272793000
H	4.213835000	1.063680000	-1.488634000
C	2.490375000	-0.965643000	0.117091000
C	1.689002000	-1.836878000	2.331988000
H	2.725979000	-1.579071000	2.652061000
C	0.710742000	-1.372578000	3.416363000
H	-0.336942000	-1.588547000	3.120129000
H	0.797515000	-0.283917000	3.602547000
H	0.899193000	-1.903447000	4.372232000
C	1.642296000	-3.365032000	2.142174000
H	1.885153000	-3.893706000	3.088665000
H	2.380607000	-3.676938000	1.377147000
H	0.632619000	-3.693687000	1.814855000
C	6.377609000	-0.222024000	0.665717000
H	6.145919000	0.729996000	0.142971000
H	7.466673000	-0.413288000	0.553810000
H	6.166483000	-0.073113000	1.745525000
C	5.827334000	-1.914679000	-1.862238000
H	5.232398000	-2.730863000	-2.325070000
H	6.900156000	-2.194275000	-1.949564000
H	5.671351000	-0.994715000	-2.463403000
C	5.863680000	-3.256750000	0.884528000
H	5.656042000	-3.172167000	1.972051000
H	6.946611000	-3.472371000	0.757163000
H	5.298332000	-4.133779000	0.503052000

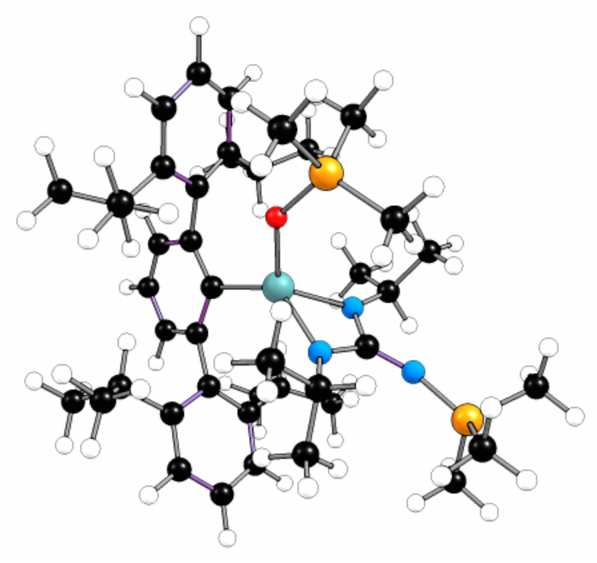

10mono
SCF = -2127.81383333
H(0 K) = -2126.890301
H(298 K) = -2126.835750
G(298 K) = -2126.979241
SCF+D3 = -2128.08642727
PCM SCF (Benzene) = -2127.82052631
BS2 (def2-tzvp) = -2129.86773859
Low Freq. = 13.5831cm-1, 18.2668cm-1

109
SnC43N3H61O

C	-5.353395000	0.959143000	1.439426000
C	-4.959847000	-0.365325000	1.210695000
C	-3.683813000	-0.675962000	0.681239000
C	-2.785918000	0.390940000	0.388643000
C	-3.198035000	1.738697000	0.568406000
C	-4.478294000	2.002402000	1.113434000
Sn	-0.912928000	0.022388000	-0.736708000
N	0.749087000	-1.183629000	0.114257000
C	1.651455000	-0.182703000	-0.007895000
N	3.031661000	-0.392759000	0.130796000
C	3.633969000	-1.477945000	-0.689699000
C	-3.403309000	-2.134350000	0.439160000
C	-3.482486000	-2.676901000	-0.879778000
C	-3.313736000	-4.069652000	-1.045007000
C	-3.103128000	-4.942225000	0.038280000
C	-3.076886000	-4.387943000	1.333475000
C	-3.235863000	-3.006303000	1.555762000
C	-3.784373000	-1.821059000	-2.087521000
C	-3.283450000	-2.490615000	2.981132000
C	-2.400775000	2.945759000	0.156246000
C	-2.472541000	3.411306000	-1.189236000
C	-1.882694000	4.655815000	-1.504044000
C	-1.243256000	5.453562000	-0.538016000
C	-1.177604000	4.964473000	0.783472000

C	-1.740636000	3.727076000	1.147928000
C	-3.166764000	2.615694000	-2.272284000
C	-1.651898000	3.245944000	2.580518000
C	-0.615973000	6.779893000	-0.905117000
C	-2.899417000	-6.425088000	-0.180039000
N	1.091685000	1.008324000	-0.322385000
C	1.812888000	2.046938000	-1.081286000
C	1.142232000	2.308753000	-2.442076000
C	0.951864000	-2.399886000	0.902472000
C	0.308444000	-2.264257000	2.292822000
C	0.438820000	-3.626382000	0.135152000
C	1.942092000	3.351284000	-0.277830000
H	2.047060000	-2.514872000	1.060113000
H	2.835026000	1.650264000	-1.279866000
H	-2.939415000	-5.053517000	2.203045000
H	-1.950128000	5.020398000	-2.543161000
H	-5.656342000	-1.192141000	1.424022000
H	-0.676977000	5.569095000	1.559177000
H	-3.376746000	-4.485017000	-2.065181000
H	-4.557821000	-1.058453000	-1.861209000
H	-4.150050000	-2.447648000	-2.925571000
H	-2.875899000	-1.267758000	-2.445532000
H	-2.815399000	-1.491777000	3.088163000
H	-2.778350000	-3.191920000	3.675567000
H	-4.332329000	-2.380829000	3.334067000
H	-4.788150000	3.051145000	1.250897000
H	-0.652037000	-3.549934000	-0.051699000
H	0.620454000	-4.556850000	0.711903000
H	0.943849000	-3.719946000	-0.848124000
H	-6.352779000	1.178852000	1.848079000
H	-2.654604000	3.154720000	3.049035000
H	-1.047706000	3.938651000	3.199548000
H	-1.189806000	2.238073000	2.639198000
H	0.735313000	-1.401176000	2.844537000
H	0.474013000	-3.179128000	2.899859000
H	-0.784102000	-2.110161000	2.200749000
H	0.940062000	3.766938000	-0.050459000
H	2.506495000	4.110098000	-0.859760000
H	2.471888000	3.200563000	0.685265000
H	-2.555981000	1.731832000	-2.583654000
H	-3.338090000	3.240225000	-3.171552000
H	-4.146486000	2.224193000	-1.929093000
H	0.482644000	6.676503000	-1.047776000
H	-0.767416000	7.538574000	-0.109481000
H	-1.032861000	7.182866000	-1.849968000
H	-1.819453000	-6.668555000	-0.292062000
H	-3.407921000	-6.776581000	-1.100758000
H	-3.279799000	-7.021928000	0.674230000
H	0.923835000	1.367516000	-2.985654000
H	1.791684000	2.958548000	-3.065761000
H	0.174703000	2.835049000	-2.303164000
C	4.654347000	-2.382785000	0.033589000

H	2.777101000	-2.129016000	-0.965109000
C	5.097655000	-3.550053000	-0.870645000
H	5.551191000	-1.791191000	0.322037000
H	4.217241000	-2.775784000	0.976134000
C	5.660553000	-3.056138000	-2.213597000
H	5.847404000	-4.173432000	-0.337237000
H	4.223220000	-4.213496000	-1.062598000
C	4.653289000	-2.143717000	-2.932407000
H	6.604327000	-2.491627000	-2.031067000
H	5.934529000	-3.919150000	-2.858125000
C	4.212866000	-0.970920000	-2.036747000
H	5.082876000	-1.755520000	-3.880839000
H	3.757548000	-2.740407000	-3.220838000
H	5.082753000	-0.305313000	-1.849260000
H	3.444305000	-0.359547000	-2.555165000
C	3.783032000	0.504634000	1.042100000
C	5.078149000	1.123955000	0.472654000
H	3.087590000	1.349923000	1.229410000
C	5.690852000	2.138776000	1.458598000
H	5.824012000	0.324050000	0.269258000
H	4.868090000	1.617461000	-0.499314000
C	5.941575000	1.520936000	2.844007000
H	6.633837000	2.548567000	1.036818000
H	4.998724000	3.005450000	1.565282000
C	4.655569000	0.896705000	3.410227000
H	6.725733000	0.733205000	2.759710000
H	6.344057000	2.286604000	3.541914000
C	4.052013000	-0.128934000	2.433081000
H	4.852606000	0.415714000	4.392605000
H	3.911229000	1.703465000	3.604018000
H	4.749733000	-0.987143000	2.330488000
H	3.101059000	-0.538095000	2.836043000
O	-1.113202000	-0.141849000	-2.617645000

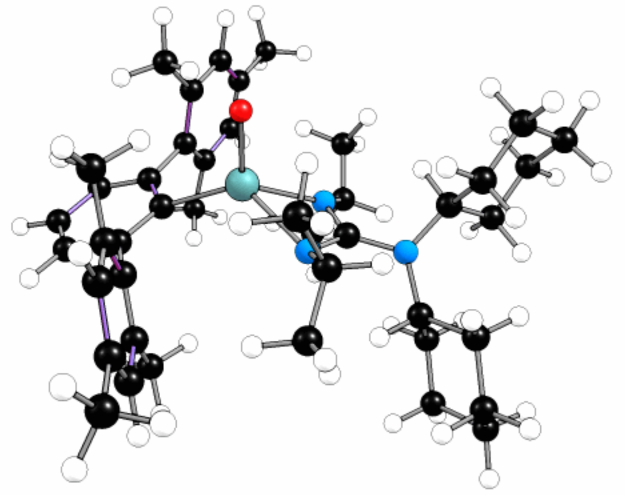

10cis

SCF = -4255.68784695

H(0 K) = -4253.838160

H(298 K) = -4253.729116

G(298 K) = -4253.987807

SCF+D3 = -4256.31165400
PCM SCF (Benzene) = -4255.69240927
BS2 (def2-tzvp) = -4259.78009891
Low Freq. = 6.6644cm-1, 16.1569cm-1

218
Compound 10 cis

C	2.740344	-2.505403	0.751557
N	3.045984	0.406662	-1.366807
O	-0.006320	-0.779813	1.397110
Sn	1.561117	-0.754644	-0.007096
O	-0.012622	-0.910399	-1.312240
Sn	-1.583728	-0.728517	0.088944
N	2.224540	1.300697	0.527131
C	3.179824	-2.502769	2.110870
N	3.817444	2.636559	-0.787793
C	3.562321	-3.721615	2.724846
H	3.899111	-3.696465	3.774100
N	-2.514663	1.097101	-0.797234
C	3.546058	-4.931307	2.021634
H	3.825336	-5.873355	2.520349
N	-2.738447	0.803346	1.425112
C	3.228314	-4.913938	0.659401
H	3.290940	-5.839707	0.064126
N	-3.728951	2.804737	0.416232
C	2.848564	-3.716766	0.002303
C	3.461224	-1.292180	2.968317
C	2.631389	-0.947619	4.071264
C	3.086209	0.017572	4.997216
H	2.435814	0.272243	5.851512
C	4.342477	0.638147	4.881303
C	5.145201	0.288840	3.776658
H	6.139512	0.754585	3.663105
C	4.730399	-0.657015	2.819487
C	1.284521	-1.597267	4.263205
H	1.378818	-2.699828	4.341998
H	0.624091	-1.389278	3.389551
H	0.788284	-1.228680	5.182737
C	4.820259	1.653888	5.895281
H	4.157588	1.689205	6.783167
H	4.850946	2.677326	5.460324
H	5.849309	1.426219	6.245670
C	5.657247	-1.016705	1.678190
H	6.621486	-0.477033	1.762967
H	5.206863	-0.766429	0.694028
H	5.872011	-2.105678	1.653675
C	2.745399	-3.853426	-1.496903
C	3.948934	-3.747062	-2.252539
C	3.924809	-4.035671	-3.632075
H	4.863047	-3.950385	-4.206905
C	2.749145	-4.446142	-4.289107
C	1.582518	-4.585348	-3.514482

H	0.656917	-4.943020	-3.995319
C	1.559374	-4.309086	-2.131712
C	5.258865	-3.385056	-1.586219
H	5.570155	-4.166355	-0.860122
H	5.177784	-2.441391	-1.010151
H	6.069827	-3.269598	-2.332656
C	2.739408	-4.720416	-5.776769
H	1.980700	-5.482898	-6.047810
H	3.727687	-5.074596	-6.135360
H	2.495092	-3.799984	-6.352382
C	0.301327	-4.536144	-1.333070
H	-0.482319	-5.033500	-1.935912
H	-0.113648	-3.571930	-0.972294
H	0.500408	-5.161573	-0.439577
C	3.041935	1.481143	-0.537379
C	3.286048	0.528282	-2.812434
H	3.536890	1.593010	-3.017638
C	4.457780	-0.342710	-3.294285
H	5.380412	-0.169218	-2.703967
H	4.687952	-0.129030	-4.359485
H	4.197502	-1.415949	-3.216594
C	2.017689	0.200460	-3.620389
H	1.676472	-0.836502	-3.426668
H	2.219444	0.307130	-4.707555
H	1.181099	0.873022	-3.351077
C	2.105404	1.930596	1.852425
H	2.079878	1.072411	2.557324
C	0.747036	2.640362	2.003916
H	-0.078150	1.945135	1.752787
H	0.679966	3.537474	1.351251
H	0.611216	2.975359	3.054002
C	3.253855	2.831033	2.324441
H	3.257405	3.816755	1.823419
H	4.239752	2.351661	2.183840
H	3.126882	3.015882	3.410671
C	5.246010	2.432426	-1.156227
H	5.328839	1.338632	-1.321785
C	5.737602	3.106265	-2.460438
H	5.068743	2.842425	-3.305450
H	5.697496	4.212721	-2.358411
C	7.185737	2.695426	-2.794633
H	7.212625	1.604889	-3.020573
H	7.515063	3.210199	-3.723097
C	8.151983	2.994453	-1.637732
H	8.217784	4.097061	-1.487324
H	9.178986	2.653070	-1.891491
C	7.667330	2.332932	-0.338102
H	8.345332	2.583848	0.506350
H	7.713273	1.225290	-0.453583
C	6.224942	2.747938	0.006353
H	5.882673	2.219407	0.919675
H	6.204582	3.833641	0.240420

C	3.130806	3.952119	-0.846682
H	2.243475	3.843632	-0.185229
C	3.937095	5.158596	-0.313401
H	4.329955	4.948291	0.701376
H	4.821880	5.337558	-0.961737
C	3.078319	6.438136	-0.292753
H	3.683330	7.287946	0.091047
H	2.239728	6.304276	0.429115
C	2.505151	6.765821	-1.680834
H	3.341798	7.019688	-2.372205
H	1.857815	7.668184	-1.631388
C	1.723109	5.569462	-2.245885
H	1.354516	5.790046	-3.271168
H	0.816511	5.399489	-1.621156
C	2.575576	4.287176	-2.257931
H	1.976499	3.429259	-2.626879
H	3.419477	4.416355	-2.968975
C	-2.790234	-2.603375	-0.243757
C	-2.902370	-3.630211	0.748663
C	-3.268086	-4.943731	0.362869
H	-3.325751	-5.718740	1.144663
C	-3.583541	-5.258518	-0.962268
H	-3.855579	-6.287656	-1.246841
C	-3.608869	-4.227400	-1.905540
H	-3.948200	-4.428815	-2.934572
C	-3.230637	-2.901313	-1.572278
C	-2.832369	-3.456737	2.246566
C	-1.644870	-3.715588	2.982516
C	-1.692454	-3.691659	4.391672
H	-0.764928	-3.894428	4.952618
C	-2.883435	-3.443542	5.099935
C	-4.060830	-3.251601	4.352558
H	-5.017683	-3.099678	4.881134
C	-4.063298	-3.276602	2.943306
C	-0.363355	-4.056496	2.268299
H	0.030093	-3.163120	1.737730
H	-0.527428	-4.850433	1.512419
H	0.416877	-4.405272	2.971585
C	-2.896623	-3.382908	6.611304
H	-3.882998	-3.676245	7.025589
H	-2.684492	-2.352096	6.972704
H	-2.125308	-4.045756	7.054429
C	-5.384597	-3.210524	2.204475
H	-5.683421	-4.218808	1.842615
H	-5.336924	-2.566123	1.304629
H	-6.195107	-2.839001	2.862947
C	-3.517275	-1.931723	-2.694598
C	-2.738363	-1.942467	-3.885850
C	-3.223343	-1.280298	-5.031222
H	-2.613266	-1.304075	-5.950473
C	-4.451908	-0.592365	-5.037756
C	-5.192630	-0.567167	-3.840329

H	-6.163335	-0.042276	-3.817782
C	-4.754661	-1.227941	-2.673389
C	-1.402367	-2.642501	-3.927330
H	-1.506196	-3.730520	-3.735484
H	-0.726925	-2.238468	-3.140094
H	-0.912183	-2.514848	-4.912716
C	-4.938069	0.124909	-6.277725
H	-4.679952	-0.434320	-7.200749
H	-4.471395	1.130927	-6.369751
H	-6.036995	0.273741	-6.261811
C	-5.640260	-1.236481	-1.446810
H	-5.918640	-2.271945	-1.156725
H	-6.575358	-0.669395	-1.625577
H	-5.127884	-0.790093	-0.569484
C	-3.009767	1.591221	0.375834
C	-2.001859	1.996531	-1.846566
H	-2.701634	2.857355	-1.922644
C	-0.620360	2.561061	-1.485796
H	-0.635373	3.085408	-0.510369
H	-0.285171	3.280854	-2.260950
H	0.119567	1.738380	-1.421693
C	-1.965847	1.294089	-3.202331
H	-1.277302	0.426454	-3.157765
H	-1.601568	1.989371	-3.987415
H	-2.969418	0.932664	-3.492303
C	-3.334621	0.856191	2.762806
H	-3.387815	1.917092	3.093296
C	-4.767982	0.301177	2.800460
H	-5.180314	0.347294	3.830520
H	-4.781579	-0.755737	2.476444
H	-5.441189	0.879638	2.137237
C	-2.410045	0.132830	3.752288
H	-2.806276	0.220766	4.785045
H	-1.389502	0.560984	3.719527
H	-2.325880	-0.944518	3.506727
C	-4.815264	2.985453	-0.585536
H	-4.589336	2.240182	-1.377959
C	-4.869732	4.365837	-1.277073
H	-5.130156	5.151156	-0.533229
H	-3.871421	4.630189	-1.684157
C	-5.923255	4.386929	-2.401430
H	-5.945639	5.392121	-2.875280
H	-5.618175	3.672968	-3.200453
C	-7.317911	4.001973	-1.882724
H	-8.052840	3.986731	-2.716540
H	-7.672580	4.780960	-1.168534
C	-7.279296	2.639821	-1.172130
H	-8.278551	2.383561	-0.758297
H	-7.039823	1.848518	-1.919078
C	-6.222870	2.608862	-0.051652
H	-6.180949	1.596292	0.400419
H	-6.523709	3.309016	0.757093

C	-3.372447	3.806639	1.448859
H	-2.650119	3.277266	2.105119
C	-2.583450	5.034527	0.917538
H	-1.748650	4.685340	0.275272
H	-3.233752	5.669033	0.280849
C	-2.050220	5.887077	2.084285
H	-1.317666	5.288704	2.673490
H	-1.489143	6.761606	1.689381
C	-3.189052	6.350398	3.008075
H	-2.784996	6.930205	3.866083
H	-3.854207	7.047216	2.447103
C	-4.016751	5.156070	3.511447
H	-4.870445	5.506114	4.131252
H	-3.384430	4.529721	4.182444
C	-4.534017	4.281992	2.351813
H	-5.258056	4.873386	1.747861
H	-5.091150	3.407033	2.747005

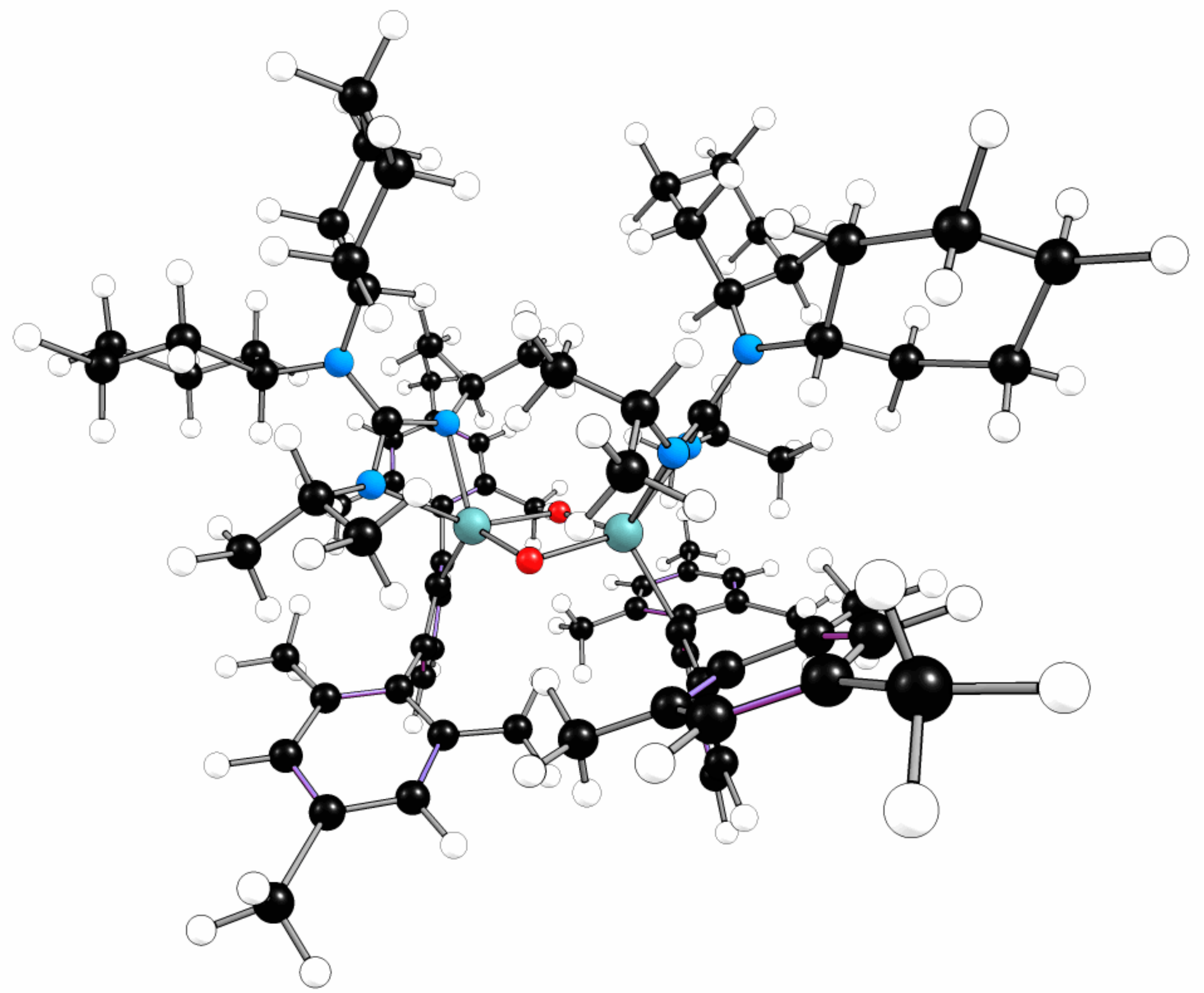

10trans

SCF = -4255.68696877

H(0 K) = -4253.837272

H(298 K) = -4253.728287

G(298 K) = -4253.984972

SCF+D3 = -4256.30905726

PCM SCF (Benzene) = -4255.69174793

BS2 (def2-tzvp) = -4259.77901153
Low Freq. = 13.5832cm-1, 17.5504cm-1

218
Compound 10 trans

C	-1.253473	2.236161	2.064524
N	-3.409290	1.081695	-0.524347
O	-0.045512	0.659541	-1.056237
Sn	-1.360323	0.617918	0.518539
O	0.082970	-0.693837	1.301357
Sn	1.404874	-0.642228	-0.294703
N	-2.898456	-0.950245	0.292413
C	-1.436972	1.843797	3.424473
N	-5.104706	-0.645361	-0.751758
C	-0.974759	2.686194	4.465296
H	-1.131209	2.369476	5.509406
N	3.252684	-0.111296	0.857385
C	-0.349448	3.906925	4.185967
H	0.036548	4.537855	5.002763
N	3.108487	0.597219	-1.276972
C	-0.285980	4.346757	2.858359
H	0.112450	5.348598	2.627432
N	5.139543	1.127696	-0.012567
C	-0.764551	3.551847	1.786801
C	-2.246460	0.658209	3.890959
C	-1.633758	-0.459910	4.523569
C	-2.448848	-1.423955	5.155067
H	-1.961306	-2.284422	5.644455
C	-3.849356	-1.307107	5.207566
C	-4.436752	-0.192601	4.576305
H	-5.532842	-0.067795	4.611397
C	-3.667653	0.786130	3.916080
C	-0.135453	-0.625968	4.520174
H	0.378696	0.254516	4.957796
H	0.221215	-0.722503	3.469186
H	0.172299	-1.523975	5.091499
C	-4.702654	-2.350176	5.893867
H	-4.108250	-2.968725	6.596201
H	-5.163777	-3.041815	5.154101
H	-5.535782	-1.887362	6.462916
C	-4.362170	1.977633	3.292420
H	-5.456232	1.933188	3.463987
H	-4.185773	2.026235	2.196576
H	-3.986726	2.933199	3.715473
C	-0.880339	4.294822	0.473987
C	-2.005224	5.160952	0.321806
C	-2.109759	5.967068	-0.829563
H	-2.989075	6.625532	-0.934828
C	-1.125755	5.966004	-1.836097
C	0.001017	5.147701	-1.640809
H	0.809094	5.154512	-2.392143
C	0.151774	4.328634	-0.501511

C	-3.063735	5.274838	1.399289
H	-2.660092	5.769927	2.308578
H	-3.430738	4.282484	1.727670
H	-3.929558	5.870480	1.047107
C	-1.279012	6.807427	-3.083583
H	-0.295161	7.105628	-3.500192
H	-1.864561	7.729146	-2.887900
H	-1.813076	6.245709	-3.881939
C	1.412841	3.525528	-0.330031
H	2.190842	3.856312	-1.046620
H	1.214852	2.447658	-0.519375
H	1.817287	3.624443	0.697320
C	-3.833333	-0.189872	-0.334477
C	-3.864960	1.911810	-1.648215
H	-4.561791	1.293937	-2.261279
C	-4.617058	3.176123	-1.196599
H	-5.480325	2.946685	-0.540862
H	-4.998629	3.735018	-2.077025
H	-3.934812	3.848471	-0.644388
C	-2.673651	2.303393	-2.542036
H	-1.962753	2.952192	-1.989999
H	-3.027544	2.866815	-3.431209
H	-2.110094	1.415175	-2.883686
C	-2.955154	-2.220078	1.033916
H	-2.157032	-2.096187	1.798052
C	-2.515218	-3.425743	0.179872
H	-1.547971	-3.225418	-0.321115
H	-3.262406	-3.693484	-0.595636
H	-2.385755	-4.317499	0.826908
C	-4.254355	-2.500762	1.799377
H	-5.104190	-2.721581	1.126926
H	-4.526049	-1.643929	2.445326
H	-4.106596	-3.382557	2.456881
C	-6.285545	0.175583	-0.367787
H	-5.847301	1.115087	0.025974
C	-7.243309	0.583464	-1.511565
H	-6.673995	1.062212	-2.334956
H	-7.725708	-0.321849	-1.941649
C	-8.346832	1.537905	-1.013093
H	-7.882210	2.498445	-0.692471
H	-9.028315	1.792109	-1.853625
C	-9.135857	0.943451	0.163820
H	-9.699304	0.046910	-0.184753
H	-9.896987	1.667912	0.526540
C	-8.188598	0.535893	1.302903
H	-8.755910	0.070409	2.137882
H	-7.711501	1.450686	1.724971
C	-7.092606	-0.428518	0.812879
H	-6.396499	-0.669431	1.640678
H	-7.564373	-1.385431	0.503402
C	-5.178146	-1.815431	-1.662684
H	-4.213885	-2.343226	-1.514095

C	-6.295223	-2.841934	-1.364885
H	-6.267698	-3.147881	-0.299359
H	-7.291243	-2.376796	-1.535088
C	-6.167706	-4.085009	-2.267823
H	-6.988565	-4.798858	-2.039488
H	-5.219401	-4.617043	-2.023565
C	-6.169845	-3.717403	-3.760194
H	-7.167227	-3.301774	-4.034249
H	-6.029392	-4.626632	-4.384055
C	-5.083272	-2.675280	-4.069748
H	-5.122820	-2.374064	-5.138935
H	-4.079595	-3.132698	-3.914767
C	-5.220224	-1.434120	-3.168839
H	-4.409639	-0.707918	-3.385424
H	-6.176681	-0.921631	-3.403659
C	1.357516	-2.754001	-1.096826
C	0.852196	-3.084911	-2.395955
C	0.406939	-4.404135	-2.662569
H	0.002032	-4.623502	-3.664150
C	0.501493	-5.419179	-1.706077
H	0.139203	-6.436169	-1.926363
C	1.125091	-5.131896	-0.488711
H	1.302584	-5.935870	0.243893
C	1.567566	-3.822948	-0.168331
C	0.870164	-2.217648	-3.631631
C	-0.259669	-1.459667	-4.037195
C	-0.238509	-0.803556	-5.285205
H	-1.120612	-0.211994	-5.583950
C	0.859600	-0.893361	-6.160915
C	1.947697	-1.694272	-5.764718
H	2.807018	-1.813188	-6.446917
C	1.966898	-2.372575	-4.529282
C	-1.474281	-1.367006	-3.154681
H	-1.250048	-0.723562	-2.275595
H	-1.780438	-2.362173	-2.772745
H	-2.326230	-0.921043	-3.702516
C	0.874479	-0.146616	-7.476181
H	1.507903	-0.656185	-8.230881
H	1.281654	0.881002	-7.348110
H	-0.146021	-0.039255	-7.897792
C	3.112161	-3.314350	-4.218444
H	2.797223	-4.374041	-4.338662
H	3.465933	-3.218477	-3.172806
H	3.970055	-3.141226	-4.898552
C	2.375442	-3.792762	1.107595
C	1.749128	-3.970318	2.372718
C	2.544731	-4.258527	3.500154
H	2.045958	-4.399065	4.474434
C	3.944221	-4.385416	3.419224
C	4.546344	-4.191047	2.160884
H	5.641298	-4.292610	2.065424
C	3.792339	-3.898526	1.006019

C	0.251063	-3.851976	2.512384
H	-0.276943	-4.628346	1.920551
H	-0.086609	-2.861362	2.132380
H	-0.063259	-3.955999	3.569761
C	4.775658	-4.683348	4.647176
H	4.216961	-5.301088	5.379830
H	5.069012	-3.746075	5.170620
H	5.712390	-5.218458	4.388676
C	4.494893	-3.767863	-0.327582
H	4.113506	-4.508667	-1.061958
H	5.586780	-3.923869	-0.220357
H	4.332502	-2.765829	-0.776628
C	3.856647	0.561080	-0.162257
C	3.451429	0.295992	2.262847
H	4.475122	0.722980	2.334211
C	2.475889	1.408701	2.680629
H	2.608092	2.311455	2.051918
H	2.647682	1.703996	3.737037
H	1.427701	1.065750	2.576532
C	3.375861	-0.904636	3.205280
H	2.373177	-1.370656	3.161806
H	3.563904	-0.589047	4.252626
H	4.117502	-1.679474	2.930978
C	3.531948	0.996765	-2.623099
H	4.111469	1.944003	-2.546385
C	4.446835	-0.036205	-3.301909
H	4.732804	0.303555	-4.319698
H	3.926351	-1.006720	-3.403251
H	5.376421	-0.194558	-2.721281
C	2.292452	1.309754	-3.472450
H	2.596897	1.701202	-4.464968
H	1.644952	2.054891	-2.972409
H	1.681031	0.401264	-3.635005
C	6.198036	0.266971	0.586739
H	5.640502	-0.542974	1.103458
C	7.090893	0.937976	1.654451
H	7.697845	1.745335	1.188075
H	6.462243	1.420249	2.431781
C	8.045884	-0.080377	2.307138
H	8.680131	0.432515	3.062132
H	7.446963	-0.835652	2.865830
C	8.917730	-0.796241	1.263519
H	9.570842	-1.550658	1.753340
H	9.600386	-0.056575	0.784441
C	8.044762	-1.456211	0.184839
H	8.676572	-1.936537	-0.593354
H	7.449820	-2.275082	0.650876
C	7.083081	-0.446632	-0.469269
H	6.428947	-0.968385	-1.197979
H	7.668944	0.302977	-1.043643
C	5.345859	2.535712	-0.426127
H	4.420210	2.798668	-0.979168

C	5.397087	3.554606	0.744853
H	4.532730	3.381113	1.419563
H	6.313285	3.403568	1.351967
C	5.388543	5.000593	0.213676
H	4.422571	5.197528	-0.305536
H	5.432984	5.716145	1.062921
C	6.550223	5.248508	-0.763300
H	6.508073	6.284192	-1.164729
H	7.516144	5.167078	-0.212718
C	6.535677	4.227827	-1.913225
H	7.407449	4.383159	-2.585122
H	5.629395	4.396313	-2.539910
C	6.524774	2.776003	-1.395896
H	7.486608	2.570477	-0.875114
H	6.465973	2.064407	-2.245861

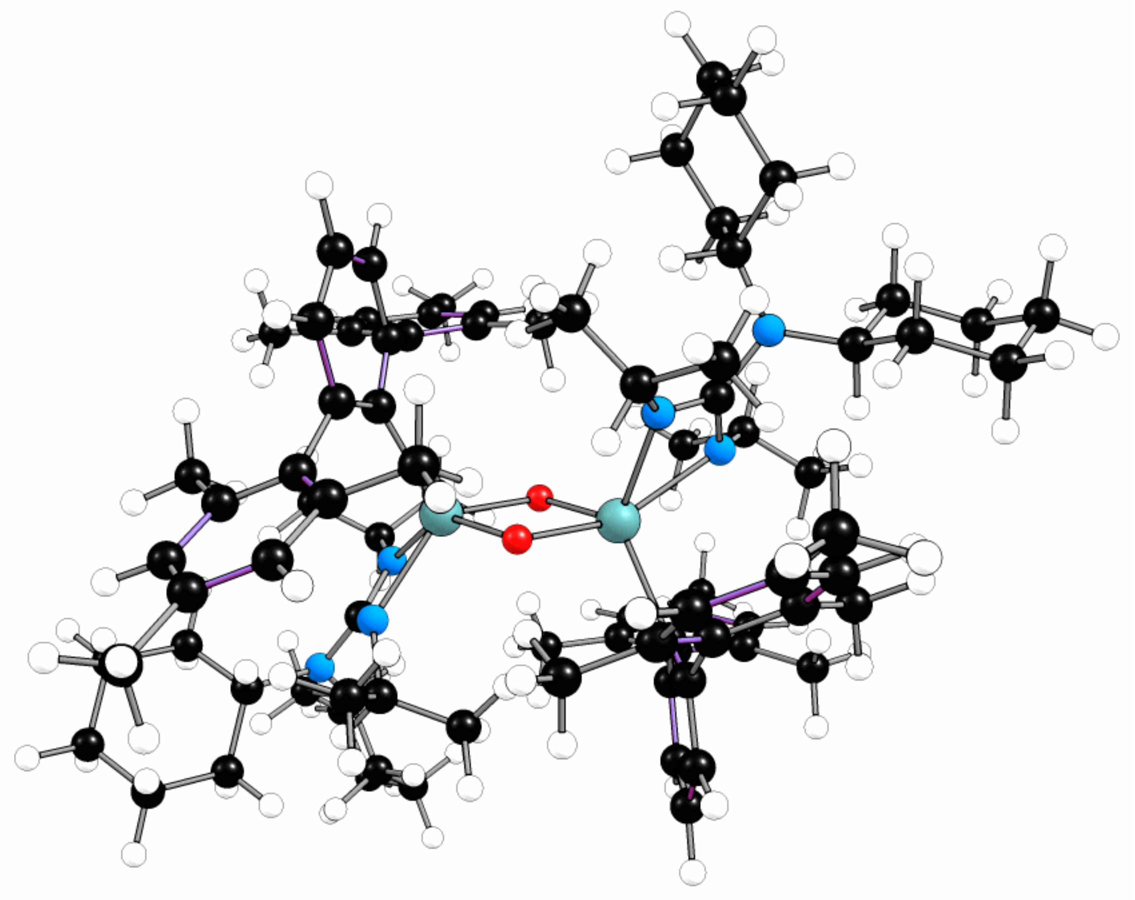

References

[S1] Fischer, M.; Roy, M. M. D.; Wales, L. L.; Ellwanger, M. A.; Heilmann, A.; Aldridge, S. Structural Snapshots in Reversible Phosphinidene Transfer: Synthetic, Structural, and Reaction Chemistry of a Sn=P Double Bond. *J. Am. Chem. Soc.* **2022**, *144*, 8908-8913.

[S2] a) Schulz, A.; Thomas, M.; Villinger, A. Tetrazastannoles *versus* distannadiazanes – a question of the tin(II) source. *Dalton Trans.* **2019**, *48*, 125-132; b) Simons, R. S.; Pu, L.; Olmstead, M. M.; Power, P. P. Synthesis and Characterization of the Monomeric Diaryls M{C_6H_3-2,6-Mes_2}$_2$ (M = Ge, Sn, or Pb; Mes = 2,4,6-$Me_3C_6H_2$-) and Dimeric Aryl-Metal Chlorides [M(Cl){C_6H_3-2,6-Mes_2}]$_2$ (M = Ge or Sn). *Organometallics* **1997**, *16*, 1920-1925.

[S3] Spek, A. L. PLATOn SQUEEZE: a tool for the calculation of the disordered solvent contribution to the calculated structure factors. *Acta Crystallogr.* **2015**, *C71*, 9-18.

[S4] Bruker AXS Inc., in *Bruker Apex CCD, SAINT v8.40B* (Ed.: Bruker AXS Inst. Inc.), WI, USA, Madison, **2019**.

[S5] Krause, L.; Herbst-Irmer, R.; Sheldrick, G. M.; Stalke, D. Comparison of silver and molybdenum microfocus X-ray sources for single-crystal structure determination. *J. Appl. Crystallogr.* **2015**, *48*, 3-10.

[S6] Sheldrick, G. M. SHELXT – Integrated space-group and crystal-structure determination. *Acta Crystallogr.* **2015**, *A71*, 3-8.

[S7] Sheldrick, G. M. Crystal structure refinement with SHELXL. *Acta Crystallogr.* **2015**, *C71*, 3-8.

[S8] Dolomanov, O. V.; Bourhis, L. J.; Gildea, R. J.; Howard, J. A.; Puschmann, H. OLEX2: a complete structure solution, refinement and analysis program. *J. Appl. Crystallogr.* **2009**, *42*, 339-341.

[S9] Hübschle, C. B.; Sheldrick, G. M.; Dittrich, B. ShelXle: a Qt graphical user interface for SHELXL. *J. Appl. Crystallogr.* **2011**, *44*, 1281-1284.

[S10] Parsons, S.; Flack, H.; Wagner, T. Use of intensity quotients and differences in absolute structure refinement. *Acta Crystallogr.* **2013**, *B69*, 249-259.

[S11] Groom, C. R.; Bruno, I. J.; Lightfoot, M. P.; Ward, S. C. The Cambridge Structural Database. *Acta Crystallogr.* **2016**, *B72*, 171-179.

[S12] Gaussian 16, Revision A.03, Frisch, M. J.; Trucks, G. W.; Schlegel, H. B.; Scuseria, G. E.; Robb, M. A.; Cheeseman, J. R.; Scalmani, G.; Barone, V.; Petersson, G. A.; Nakatsuji, H.; Li, X.; Caricato, M.; Marenich, A. V.; Bloino, J.; Janesko, B. G.; Gomperts, R.; Mennucci, B.; Hratchian, H. P.; Ortiz, J. V.; Izmaylov, A. F.; Sonnenberg, J. L.; Williams-Young, D.; Ding, F.; Lipparini, F.; Egidi, F.; Goings, J.; Peng, B.; Petrone, A.; Henderson, T.; Ranasinghe, D.; Zakrzewski, V. G.; Gao, J.; Rega, N.; Zheng, G.; Liang, W.; Hada, M.; Ehara, M.; Toyota, K.; Fukuda, R.; Hasegawa, J.; Ishida, M.; Nakajima, T.; Honda, Y.; Kitao, O.; Nakai, H.; Vreven, T.; Throssell, K.; Montgomery, J. A., Jr.; Peralta, J. E.; Ogliaro, F.; Bearpark, M. J.; Heyd, J. J.; Brothers, E. N.; Kudin, K. N.; Staroverov, V. N.; Keith, T. A.; Kobayashi, R.; Normand, J.; Raghavachari, K.; Rendell, A. P.; Burant, J. C.; Iyengar, S. S.; Tomasi, J.; Cossi, M.; Millam, J. M.; Klene, M.; Adamo, C.; Cammi, R.; Ochterski, J. W.; Martin, R. L.; Morokuma, K.; Farkas, O.; Foresman, J. B.; Fox, D. J. Gaussian, Inc., Wallingford CT, **2016**.

[S13] Becke, A. D. Density-functional exchange-energy approximation with correct asymptotic behavior. *Phys. Rev. A* **1998**, *38*, 3098-3100.

[S14] Perdew, J. P. Density-functional approximation for the correlation energy of the inhomogeneous electron gas. *Phys. Rev. B* **1986**, *33*, 8822-8824.

[S15] Weigend, F.; Ahlrichs, R. Balanced basis sets of split valence, triple zeta valence and quadruple zeta valence quality for H to Rn: Design and assessment of accuracy. *Phys. Chem. Chem. Phys.* **2005**, *7*, 3297-3305.

[S16] Grimme, S.; Antony, J.; Ehrlich, S.; Krieg, H. A consistent and accurate *ab initio* parametrization of density functional dispersion correction (DFT-D) for the 94 elements H-Pu. *J. Chem. Phys.* **2010**, *132*, 154104.

[S17] Grimme, S.; Ehrlich, S.; Goerigk, L. Effect of the damping function in dispersion corrected density functional theory. *J. Comp. Chem.* **2011**, *32*, 1456-1465.

[S18] NBO 7.0, Glendening, E. D.; Badenhoop, J. K.; Reed, A. E.; Carpenter, J. E.; Bohmann, J. A.; Morales, C. M.; Karafiloglou, P.; Landis, C. R.; Weinhold, F. Theoretical Chemistry Institute, University of Wisconsin, Madison, WI, **2018**.
